# *LMNA*-Related Dilated Cardiomyopathy: Single-Cell Transcriptomics during Patient-Derived iPSC Differentiation Support Cell Type and Lineage-Specific Dysregulation of Gene Expression and Development for Cardiomyocytes and Epicardium-Derived Cells with Lamin A/C Haploinsufficiency

**DOI:** 10.3390/cells13171479

**Published:** 2024-09-03

**Authors:** Michael V. Zaragoza, Thuy-Anh Bui, Halida P. Widyastuti, Mehrsa Mehrabi, Zixuan Cang, Yutong Sha, Anna Grosberg, Qing Nie

**Affiliations:** 1 UCI Cardiogenomics Program, Pediatrics and Biological Chemistry, UC Irvine School of Medicine, Irvine, CA 92697, USA; 2 Sue & Bill Gross Stem Cell Research Center, University of California, Irvine, Irvine, CA 92697, USA; 3 Biomedical Engineering and Edwards Lifesciences Foundation Cardiovascular Innovation and Research Center, University of California, Irvine, Irvine, CA 92697, USA; 4 Mathematics and NSF-Simons Center for Multiscale Cell Fate Research, University of California, Irvine, Irvine, CA 92697, USA

**Keywords:** nuclear lamina, disease modeling, stem cells, single-cell RNA-seq, differentially expressed genes, epigenetics, X-inactivation, genomic imprinting, pluripotency, cell fate

## Abstract

*LMNA*-related dilated cardiomyopathy (DCM) is an autosomal-dominant genetic condition with cardiomyocyte and conduction system dysfunction often resulting in heart failure or sudden death. The condition is caused by mutation in the Lamin A/C (*LMNA*) gene encoding Type-A nuclear lamin proteins involved in nuclear integrity, epigenetic regulation of gene expression, and differentiation. The molecular mechanisms of the disease are not completely understood, and there are no definitive treatments to reverse progression or prevent mortality. We investigated possible mechanisms of *LMNA*-related DCM using induced pluripotent stem cells derived from a family with a heterozygous *LMNA* c.357-2A>G splice-site mutation. We differentiated one *LMNA*-mutant iPSC line derived from an affected female (Patient) and two non-mutant iPSC lines derived from her unaffected sister (Control) and conducted single-cell RNA sequencing for 12 samples (four from Patients and eight from Controls) across seven time points: Day 0, 2, 4, 9, 16, 19, and 30. Our bioinformatics workflow identified 125,554 cells in raw data and 110,521 (88%) high-quality cells in sequentially processed data. Unsupervised clustering, cell annotation, and trajectory inference found complex heterogeneity: ten main cell types; many possible subtypes; and lineage bifurcation for cardiac progenitors to cardiomyocytes (CMs) and epicardium-derived cells (EPDCs). Data integration and comparative analyses of Patient and Control cells found cell type and lineage-specific differentially expressed genes (DEGs) with enrichment, supporting pathway dysregulation. Top DEGs and enriched pathways included 10 *ZNF* genes and RNA polymerase II transcription in pluripotent cells (PP); *BMP4* and TGF Beta/BMP signaling, sarcomere gene subsets and cardiogenesis, *CDH2* and EMT in CMs; *LMNA* and epigenetic regulation, as well as *DDIT4* and mTORC1 signaling in EPDCs. Top DEGs also included *XIST* and other X-linked genes, six imprinted genes (*SNRPN*, *PWAR6*, *NDN*, *PEG10*, *MEG3*, *MEG8*), and enriched gene sets related to metabolism, proliferation, and homeostasis. We confirmed Lamin A/C haploinsufficiency by allelic expression and Western blot. Our complex Patient-derived iPSC model for Lamin A/C haploinsufficiency in PP, CM, and EPDC provided support for dysregulation of genes and pathways, many previously associated with Lamin A/C defects, such as epigenetic gene expression, signaling, and differentiation. Our findings support disruption of epigenomic developmental programs, as proposed in other *LMNA* disease models. We recognized other factors influencing epigenetics and differentiation; thus, our approach needs improvement to further investigate this mechanism in an iPSC-derived model.

## 1. Introduction

Lamins are intermediate filament proteins of the nuclear lamina that interact with chromatin and the inner nuclear membrane and are distributed throughout the nucleoplasm [1,2]. Lamins have diverse roles in both nuclear structure and function, including gene expression, chromatin regulation, cell proliferation, and differentiation [1,2]. Epigenomic roles involve heterochromatin binding and genome organization through lamina-associated domains (LADs) [3,4]. In humans, nuclear lamins are A-type lamins (Lamin A and C isoforms encoded by the *LMNA* gene) and B-type lamins (lamin B1, encoded by *LMNB1*, and lamin B2 and B3, encoded by *LMNB2*) [1,2]. B-type lamins are constitutively expressed, and A-type lamins are expressed temporally during development in most differentiated cells [5,6].

Despite *LMNA* expression in most differentiated cells, *LMNA* mutations primarily affect mesoderm-derived lineages in diseases collectively called laminopathies [1,7]. *LMNA*-related dilated cardiomyopathy (DCM), the heart-specific laminopathy, is an autosomal-dominant condition with ventricular enlargement, systolic dysfunction, and conduction system disease often resulting in heart failure or sudden death [8]. Despite clinical severity, the molecular mechanisms of *LMNA*-related DCM are not completely understood, and there are no definitive treatments to reverse progression or prevent mortality.

For heart and striated muscle laminopathies, two interconnected mechanisms are proposed to explain cell type-specific effects of lamin mutations: the ‘mechanical stress’ hypothesis, based on nuclear structure, and the ‘gene expression’ hypothesis, based on nuclear function [1,7,9]. In the ‘mechanical stress’ hypothesis, *LMNA* mutations alter the lamina structure, leading to the mechanical fragility of nuclei and cell dysfunction. In the ‘gene expression’ hypothesis, *LMNA* mutations dysregulate nuclear chromatin organization and gene transcription to alter cell function [1,7,9]. The ‘gene expression’ hypothesis involves epigenomic defects, affecting normal cell differentiation, in which *LMNA* mutations uncouple the LAD from peripheral heterochromatin to alter genome organization and unlock aberrant genes and pathways [10,11,12].

For *LMNA*-related DCM, the ‘gene expression’ hypothesis is supported by comparisons of *LMNA*-mutant and control cells across three main types of experimental models: mouse models, patient heart tissue, and induced pluripotent stem cell (iPSC) models [1,7,9,11,12]. From *LMNA*-mutant mouse models, evidence supports cell-signaling dysregulation, including the hyperactivation of the TGF Beta [13,14] and AKT-mTORC1 signaling pathways [15], as well as dysregulated differentiation pathways, including epithelial–mesenchymal transition (EMT) [16]. From *LMNA* patient heart tissue, evidence also supports dysregulation of signal transduction and gene expression including the TGF Beta/BMP signaling pathway and *BMP4* overexpression [17,18].

While evidence from numerous mouse models and limited patient heart tissue supports the ‘gene expression’ hypothesis for *LMNA*-related DCM, another valuable approach involves Patient-derived iPSC models, where somatic cells from patients are reprogramed into iPSCs [19] that can be directly differentiated in vitro into cardiomyocytes (iPSC-CMs) for analyses [20]. From *LMNA* Patient-derived iPSCs, evidence supports the dysregulation of PDGF signaling associated with open chromatin [21], non-cardiac lineage expression with chromatin compartment changes [22], and non-myocyte lineage expression with LAD changes [23]. Furthermore, the analysis of *LMNA* knockdown iPSC-CM during differentiation found a premature increase in cardiogenesis genes [24]. These studies [13,14,15,16,17,18,21,22,23,24] do provide much support for the ‘gene expression’ hypothesis; however, the results obtained using traditional techniques (e.g., bulk RNA-sequencing) may be inconsistent. For samples with ‘previously unappreciated levels of heterogeneity,’ such techniques, which average measurements across all cells in a sample, may obscure cell type-specific findings [25].

More recently, measurements at single-cell resolution are used in cardiovascular studies to evaluate the cell heterogeneity of cell samples and heart tissues and to dissect cell type-specific mechanisms in normal and disease processes [26]. To study normal processes, many recent studies used single-cell RNA-sequencing (scRNA-seq) to evaluate normal gene expression and developmental pathways in human iPSC [27,28] and during differentiation to cardiomyocytes [29,30,31,32,33,34,35,36]. In contrast, single-cell analyses to study disease processes such as *LMNA*-related DCM are limited and include our initial study of patient iPSC-CM [37], a large DCM single nuclei RNA-seq (snRNA-seq) study with heart tissue from 12 *LMNA* patients [38], and scRNA-seq studies in *Lmna* Q353R mutant mouse model and heart tissue from one *LMNA* Q353R patient [39].

To evaluate cell heterogeneity and cell type and lineage-specific mechanisms for *LMNA*-related DCM, we analyzed a Patient-derived iPSC disease model using scRNA-seq at multiple time points during CM differentiation. This extends our studies focused on a unique family with DCM with a heterozygous *LMNA* c.357-2A>G splice-site mutation [37,40,41] by testing the ‘gene expression’ hypothesis that lamin A/C haploinsufficiency may be associated with the dysregulation of epigenomic developmental pathways and the expression of genes with important roles in the nuclear function. Here, we describe our scRNA-seq bioinformatic workflow with serial, cell type-specific data processing, report our results from extensive multi-level analyses of the differentiation of Control iPSCs and *LMNA* Patient iPSCs, and discuss our evidence to support the ‘gene expression’ hypothesis, along with the challenges of *LMNA* disease modeling, using Patient-derived iPSCs.

## 2. Materials and Methods

### 2.1. Generation and Validation of Control and Patient iPSC Lines

Using standard methods, we generated and validated eight total iPSC lines (Supplementary Text, Table S1), each derived from dermal fibroblasts, as reported [41]. Of the eight iPSC lines, we generated seven iPSC lines from the study family [40]: three *LMNA*-mutant iPSC lines from three affected members heterozygous for the *LMNA* c.357-2A>G splice-site mutation (Patient) and four non-mutant iPSC lines from three unaffected, *LMNA* mutation-negative members to serve as sex and age-matched controls (Control). For Control A1, we generated two iPSC lines, CA1-A [41] and CA1-B [37], from independent clones (A and B), as reported. For Control A2, revival of cryopreserved cells had low viability; therefore, we generated Unrelated Control 2 iPSCs using purchased dermal fibroblasts from a healthy male (CC-2511, Lot No. 0000293971, Lonza, Basel, Switzerland). We conducted fibroblast collection and culturing, DNA extraction, *LMNA* genotyping, and Whole Exome Sequencing (WES), as previously described [40,42].

### 2.2. In Vitro iPSC-CM Differentiation and Cell Collection

**Figure 1:**
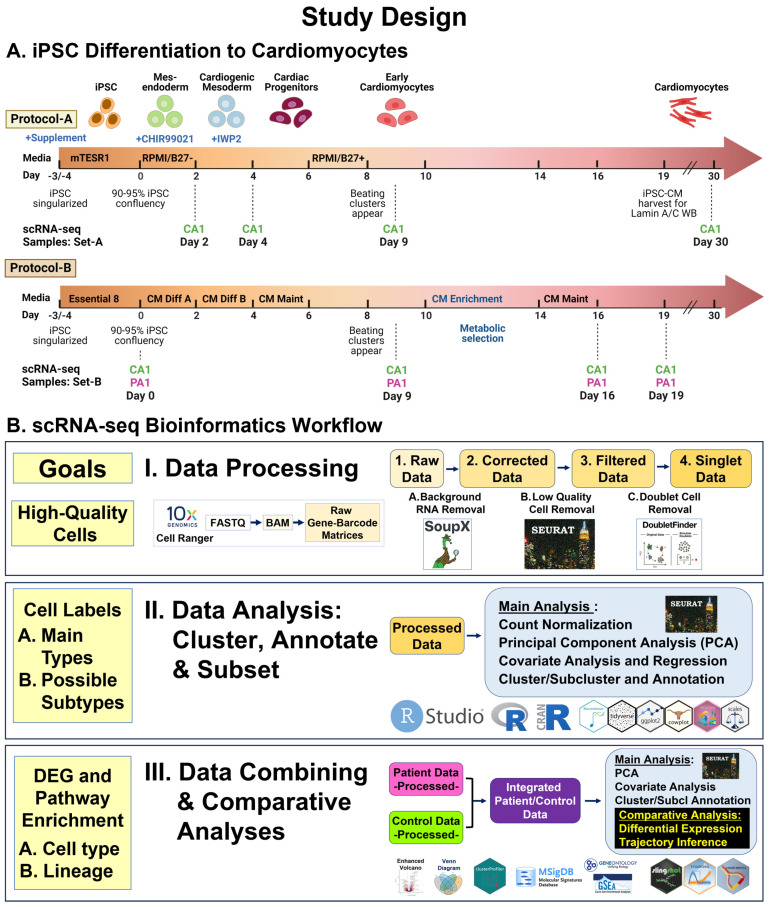
**Study design.** (**A**) iPSC differentiation into cardiomyocytes. Using standard protocols, Protocol-A and Protocol-B, we differentiated three iPSC lines (CA1-A, CA1-B, PA1) and collected 12 cell samples (8 from CA1 and 4 from PA1), across seven time points, for scRNA-seq. Using Protocol-A, we differentiated six iPSC lines (CA1-B, U2, CA3, PA1, PA2, PA3) and collected six cell samples (3 from controls and 3 from patients) at Day 19 for Western blot analysis. (**B**) scRNA-seq bioinformatics workflow. We used standard pipelines and software packages for data processing and analyses. Three main steps were involved: I. data processing for initial read processing/mapping and sequential quality control processing of raw data sets; II. data analysis for identification of main cell types and possible cell subtypes by unsupervised clustering, annotation, and subset analyses; III. data combining and comparative analyses of samples (Patient vs. Control) for cell type and lineage differentially expressed genes (DEGs) and gene set enrichment.

Using two standard protocols (A and B) (Figure 1A, Supplementary Text), we differentiated three iPSC lines (CA1-A, CA1-B, PA1) for serial scRNA-seq and six iPSC lines (CA1-B, U2, CA3, PA1, PA2, PA3) for Western blot. Protocol-A used Cardiomyocyte (CM) Differentiation Methods (Version 1.0) from the Allen Institute of Cell Science [43] based on the modulation of Wnt/beta-catenin signaling using the GSK3 inhibitor CHIR99201 and the Wnt inhibitor IWP2 [44]. Protocol-B used a Gibco PSC CM Differentiation Kit (A2921201, TFS), as described [37]. To generate iPSC-CMs, we used both protocols for scRNA-seq and only Protocol-A for Lamin A/C Western blots (WBs).

### 2.3. Serial scRNA-seq Studies Using 10× Genomics Platform

During differentiation of three iPSC lines (CA1-A, CA1-B, PA1), we collected 12 total cell samples (8 CA1 and 4 PA1 samples) across seven time points (Day 0, 2, 4, 9, 16, 19, and 30) (Figure 1A, Supplementary Text). Sample Set-A consisted of four unpaired Control samples derived from the iPSC line CA1-A, collected at Day 2, 4, 9, and 30. Sample Set-B consisted of four paired samples derived from the iPSC lines CA1-B (Control) and PA1 (Patient), differentiated in parallel, and collected at Day 0, 9, 16, and 19. Immediately after collection, we transferred cell samples to the UCI Genomics High-Throughput Facility for processing using the droplet-based chromium system [45] and Chromium Next GEM Single Cell 3ʹ Kit v3 (10× Genomics, Pleasanton, CA, USA). The facility conducted multiplex sequencing using NovaSeq 6000, with the goal of obtaining at least 50,000 raw reads per cell.

### 2.4. scRNA-seq Bioinformatics Workflow

Our workflow (Figure 1B) used standard pipelines [46,47,48] and bioinformatics software packages (Table S3) involving three steps: Step-I, data processing; Step-II, data analysis; and Step-III, data combining and comparative analyses. These steps used two general types of data: 1. single-sample data: individual data for each of the 12 samples; 2. combined data: merged (non-integrated) and integrated data from combining multiple sets or subsets of data (Supplementary Text). Since we used two different protocols—A and B (Figure 1A)—we first determined what cell types and possible subtypes are generated across both protocols in individual and merged data for all 12 samples (Sample Set-A and Set-B). To determine differential expression between Patient and Control cells, we then identified shared cell types in integrated data from paired samples (Sample Set-B) generated from Protocol-B. For two samples, CA1-B and PA1 at Day 19, we reported results for sarcomeric α-actinin expression by immunocytochemistry (ICC) staining and initial scRNA-seq analysis [37].

### 2.5. Lamin A/C Western Blot

To compare lamin A and C protein levels in Control and Patient cells, we conducted WB, as described [49], and quantitative analyses [50]. Using Protocol-A (Figure 1A), we differentiated three iPSC pairs as biological replicates (BRs): three Patient-derived iPSCs (PA1, PA2, PA3) and three Control iPSCs (CA1-B, U2, CA3) to Day 19 (Supplementary Text).

## 3. Results

### 3.1. Validated iPSC and Confirmed Identification of Cell Samples for scRNA-seq

We used eight validated iPSC lines, including three *LMNA* Patient (PA1, PA2, PA3) and five Control (CA1-A, CA1-B, CA2, CA3, U2) iPSC lines (Table S1). Validation results included normal karyotype at passage number 9 or above, normal pluripotency by ICC staining for pluripotent stem cell markers [41], and normal ICC staining for germ layer markers in EB for all eight iPSC lines (Figure S1). We differentiated seven iPSC lines and excluded one line (CA2) due to poor cell viability. Prior to scRNA-seq, each cell sample collected had cell viability of 78% or greater. Results for allelic expression of 22 coding SNV (10 autosomal and 12 XL SNV), compared to fibroblast genotype, confirmed the identification of the 12 cell samples collected for scRNA-seq (Figure S2).

### 3.2. Processed Data: 110,521 (88%) High-Quality Cells of 125,554 Cells Collected (Workflow Step-I)

To identify high-quality cells, our bioinformatics workflow (Figure 1B) began with read processing, followed by sequential quality control (QC) processing for each of the 12 cell samples. For single-sample data and combined data, the parameters and results are provided in (Supplementary Text, Tables S4–S6, Figure S3).

### 3.3. Complex Heterogeneity with Ten Main Cell Types in Control Samples, Eight Shared Cell Types between Paired Samples, and Multiple Possible Cell Subtypes (Workflow Step-II)

**Figure 2:**
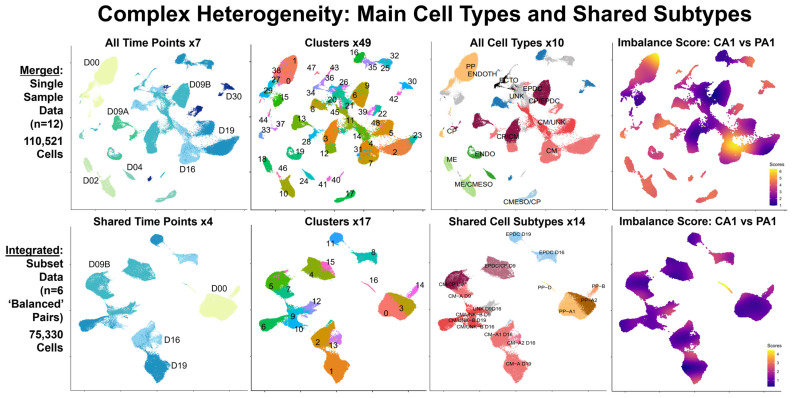
**Complex heterogeneity: main cell types and shared subtypes.** Our scRNA-seq bioinformatics workflow included analyses of combined data: merged data (**top panel**) and integrated data (**bottom panel**). Each panel consists of four UMAP plots from left to right, with clusters marked and colored by time point, pre-annotation cluster number, main cell type or shared subtype, and imbalance score. To summarize results from our individual analyses, we created merged (non-integrated) data (**top panel**) by combining processed single-sample data for all 12 samples (110,521 total cells) across all seven time points: D00, D02, D04, D09A/B, D16, D19, and D30 (**first plot**). To compare Patient and Control samples, we created integrated data (**bottom panel**) by combining processed subset data for six pairs of ‘balanced’ cell types/subtypes (75,330 total cells) at four time points: D00, D09B, D16, and D19 (**1st plot**). Unsupervised clustering (**2nd plots**) found 49 clusters in merged data and 17 clusters in integrated data. Using known markers (**3rd plots**), we identified 10 major cell types: pluripotent cells (PP), mesendoderm (ME), cardiogenic mesoderm (CMESO), endoderm (ENDO), ectoderm (ECTO), endothelium (ENDOTH), cardiac progenitors (CPs), cardiomyocytes (CMs), epicardium-derived cells (EPDCs), and unknown (UNK) cells in merged data, as well as multiple possible shared subtypes in integrated data. Using condiments [51], we calculated the “Imbalance Score” of each cell to assess if nearby cells had the same condition (**last plots**). We defined ‘balanced’ cell types/subtypes, as shared cells with low imbalance scores, which were then selected for comparative analyses between Patient and Control cells.

To identify main cell types and possible subtypes generated across both protocols and conditions, our workflow (Figure 1B) involved clustering, annotation, and subsetting using marker panels (88 total genes, Table S8) of processed data for 12 samples (8 Control and 4 Patient: 110,521 total cells). For single-sample data (Table S7, Figure S4A) and combined data (Figure 2 and Figure S4B), the results are provided as two stages: individual analyses of singlet data for the main cell types (Figure S5) and subcluster analyses of subset data for possible cell subtypes (Supplementary Text, Figure S6).

From our individual analyses, we identified ten main cell types in the Control samples and eight conserved cell types between paired samples (Figure 2, Figures S4A and S5, Table S7). In the Control samples (Figure S5B), we identified ten main cell types: 1. pluripotent cells (PP), 2. mesendoderm (ME), 3. cardiogenic mesoderm (CMESO), 4. endoderm (ENDO), 5. ectoderm (ECTO), 6. endothelium (ENDOTH), 7. cardiac progenitors (CPs), 8. cardiomyocytes (CMs), 9. epicardium-derived cells (EPDCs), and 10. unknown (UNK) cells. Of these, we found eight (PP, ENDO, ECTO, ENDOTH, CP, CM, EPDC, UNK) conserved in four Patient samples (D00, D09B, D16, D19) (Figure S5C) and two cell types (ME and CMESO) identified only in the Control samples (D02 and D04). For all 12 samples, we found clusters with unclear results and assigned these cells to the “Unknown” category (UNK). At this initial stage, we found CM consisting of two possible subtypes: clusters with high expression of multiple known CM markers (CM-A) and clusters with lower expression of fewer CM markers, except for *TTN*, and higher levels of %Mt (CM/UNK-B).

### 3.4. Four Shared Main Cell Types: PP, CP, CM, EPDC with Primarily ‘Balanced’ Cell Subtypes in Integrated Patient and Control Data (Workflow Step-III)

The final step in our workflow (Figure 1B) focused on the creation and evaluation of two types of combined data to summarize and evaluate our results: integrated data of paired samples for differential expression analysis and merged data (non-integrated). For the combined data of the paired-sample data (Figure S7A, Table S9), the results are provided for individual analyses (n = 4 pairs: 89,269 total cells) in Figure S8 and subcluster analyses (n = 11 pairs: 88,420 total cells) in Figure S9.

To identify ‘balanced’ cell types/subtypes for differential expression analysis between conditions, we created combined data: integrated and merged data for each pair of the Patient and Control samples (D00, D09B, D16, D19) and conducted both individual and subcluster analyses for the shared cell types and subtypes (Figure S7A,B). Overall, our analyses found that the Patient and Control cells clustered separately by condition in the merged data and clustered together by shared cell type/subtype in the integrated data (Figures S8A and S9A). From our individual analyses of integrated data (Figures S7A,B and S8B, Table S9, Excel Table S1-3, S1-5 and S1-6), we identified eight shared cell types (PP, ENDO, ECTO, ENDOTH, CP, CM, EPDC, UNK), the same cell types conserved in single sample data (Figure S5, Table S7, Excel Table S1-1). From our subcluster analyses (Figure S9B,C, Table S9, Excel Table S1-4), we also found the same 19 possible subtypes in six cell types (PP, ECTO, CP, CM, EPDC, UNK) in both the single-sample data (Figure S6, Table S7, Excel Table S1-2) and the integrated data. However, of eight shared cell types, imbalance scoring identified one cell type (CP) with all ‘balanced’ subtypes, four cell types (PP, ECTO, CM, EPDC) with both ‘balanced’ and ‘imbalanced’ subtypes, and three cell types (ENDO, ENDOTH, UNK) with all ‘imbalanced’ subtypes (Table S9B). Overall, four shared cell types (PP, CP, CM, EPDC) in the integrated data had mostly ‘balanced’ cell subtypes.

### 3.5. Cell Type-Specific Differential Expression: LMNA DCM-Related Gene, X-Linked Genes Including the Non-Coding XIST RNA, and Multiple Imprinted Genes (Workflow Step-IIIA)

From four shared main cell types (PP, CP, CM, EPDC) in the integrated data, we selected 14 primarily ‘balanced’ subtypes (71,541 total cells) (Table S9B) for differential expression and enrichment analyses (Table 1, Figure S10A–C). Across these 14 subtypes, we found similar average proportions of Patient (52%) and Control (48%) cells and total proportions of overexpressed DEGs (25,551 of 53,548: 48%) and underexpressed DEGs (27,997 of 53,548: 52%) in the Patient cells compared to the Control cells.

**Figure 3:**
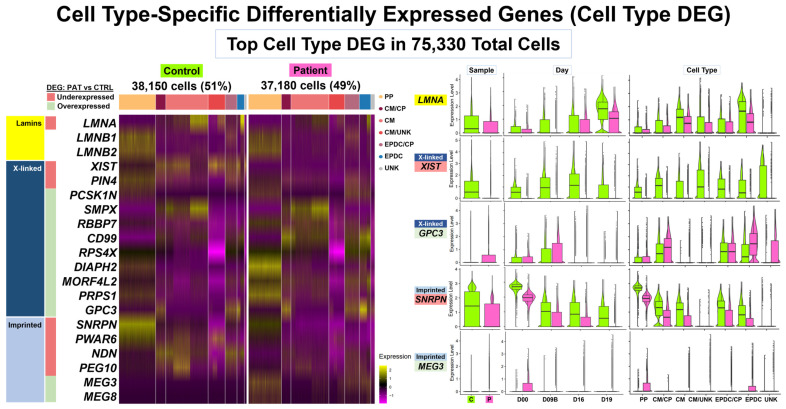
**Cell type-specific differentially expressed genes (cell type DEGs): top DEGs.** Our comparative analyses (Patient vs. Control cells) included cell type-specific differential expression and enrichment using integrated data at four time points: D00, D09B, D16, and D19. We identified cell type DEGs in six pairs of ‘balanced’ cell types/subtypes, with 75,330 total cells. Left panel: heat map for top DEGs in main cell types for 38,150 (51%) Control cells and 37,180 (49%) Patient cells. Top DEGs included *LMNA*, 11 X-linked (XL) genes, and six imprinted genes. Right panel: violin plots with boxplot by sample, day, and cell type for *LMNA*, XL genes, *XIST* and *GPC3*, and imprinted genes, *SNRPN* and *MEG3*. Overall, these results show Patient compared to Control cells had *LMNA* underexpressed in both CMs and EPDCs at Day 19, limited *XIST* expression for all time points and shared cell types, XL gene *GPC3* overexpressed in CM/CP and EPDCs, *SNRPN* underexpressed for all time points and most shared cell types, and *MEG3* overexpressed in PPs and EPDCs.

Among the top cell type DEGs, we found that our DCM study gene *LMNA* underexpressed in Patient cells compared to Control cells (Figure 3 and Figure S10D, Excel Table S2-1 and S2-2). We found that *LMNA* was underexpressed in 12 of the 14 shared cell subtypes and as a top-ten threshold DEG in two subtypes: D19 CM-A1 and D19 EPDC. Across all 14 subtypes from Days 0 to 19, *LMNA* expression increased, with the highest expression levels in CMs and EPDCs. At D19, both the Patient CMs and EPDCs had lower *LMNA* expression compared to the Control cells. In addition, the Patient cells showed primarily monoallelic expression from only the normal allele when we examined the heterozygous *LMNA* coding SNV (C>T, rs4641) (Table S1, Figure S2A, Excel Table S4-1). In contrast, we did not identify B-type lamin genes, *LMNB1* and *LMNB2*, as threshold DEGs. Expression was limited to PPs and EPDC/CP cells for *LMNB1* and to PPs for *LMNB2*, with no differences between Patient and Control cells (Figure S10D), and with biallelic *LMNB2* expression in Patient cells (Figure S2A).

The top cell type DEGs also included underexpressed X-linked (XL) genes, including *XIST*, the non-coding RNA gene that initiates X chromosome inactivation (XCI) [52], and *PIN4*, an isomerase-encoding gene with roles in cell cycle progression and chromatin remodeling [53] (Figure 3 and Figure S10D, Excel Table S2-1 and S2-2). Of the 47 genes identified as top ten underexpressed threshold DEGs, we found two (4%) XL genes: *XIST* and *PIN4*. We found *PIN4* underexpressed in 11 of the 14 shared cell subtypes and as a top ten threshold DEG in only D00 PP-A1A2, the cell subtype with the highest expression levels (Figure S10D). In contrast, we found *XIST* as the only gene ranked in the top ten threshold DEGs shared in all 14 subtypes. For all time points and shared cell types, we detected no expression or lower expression levels of *XIST* in the Patient cells compared to the Control cells (Figure 3 and Figure S10D). In addition, despite limited *XIST* expression in Patient cells, we detected a primarily monoallelic expression using heterozygous coding SNV in seven XL genes known to be subject to XCI (Figure S2B, Excel Table S4-2).

Top cell type DEGs also included multiple overexpressed XL genes, including *GPC3*, encoding Glypican Proteoglycan 3, a cell-surface glycoprotein involved in growth regulation [54]. Of the 71 genes found as top ten overexpressed threshold DEGs, we found nine (13%) XL genes: *PCSK1N*, *SMPX*, *RBBP7 (TXLNG)*, *CD99*, *RPS4X*, *DIAPH2*, *MORF4L2*, *PRPS1*, and *GPC3* across seven of the 14 shared cell subtypes (Excel Table S2-2). For overexpressed XL DEGs, the expression patterns varied across seven cell subtypes. For example, we found as a threshold DEG, *GPC3*, in two cell subtypes: D09B CM-A and D19 EPDC (Figure 3 and Figure S10D); four genes, *RBBP7 (TXLNG)*, *DIAPH2*, *MORF4L2*, and *PRPS1*, in only one subtype: D00 PP-A1A2; and both *CD99* and *RPS4X* in three subtypes: D09B CP-A, D09A CM-A, and D19 EPDC (Excel Table S2-1 and S2-2). In addition, we detected biallelic expression of *CD99* in Patient cells using a heterozygous coding SNV (C>T, rs1136447) (Figure S2B). Since *CD99* is known to escape XCI [55], we might expect this result; however, the proportion of allelic expression differed. In Patients cells, we detected equal total expression of each allele (total read count = 3528, C 51% and T 49%) compared to skewed expression of one allele (total read count = 3342, C: 81% and T:19%) in the Control cells (Excel Table S4-2). We also found similar results for another gene known to escape XCI, *EIF2S3*, and for two genes with variable XCI escape, *PLS3* and *TMEM187* (Figure S2B, Excel Table S4-2).

Top cell type DEGs included five imprinted genes, including *SNRPN* at chr15q11.2 and *MEG3* at chr14q32.2 (Figure 3, Excel Table S2-1 and S2-2) [54]. Of the 47 genes found as top ten underexpressed threshold DEGs, we found four (9%) paternally expressed imprinted genes: *SNRPN*, *PWAR6*, *NDN*, and *PEG10*, with *SNRPN* shared in nine subtypes (Excel Table S2-2). Of the 71 genes found as top ten overexpressed threshold DEGs, we found one (1%) maternally expressed imprinted gene, *MEG3*, in two subtypes: D00 PP-A1A2 and D19 EPDC (Excel Table S2-2). Across all 14 subtypes from Days 0 to 19, PP cells had the highest *SNRPN* expression, which then decreased in other shared cell types (Figure S10D). Similar to the *XIST* expression, for all time points and shared cell types, we detected lower expression levels of *SNRPN* in the Patient, compared to the Control cells, except for the CM/UNK cells (Figure 3). Likewise, for two other imprinted genes at chr15q11.2, we found *PWAR6* as an underexpressed DEG in 13 of 14 subtypes, a threshold DEG in six subtypes, a top ten DEG in four cell subtypes, an *NDN* as an underexpressed DEG in 11 of 14 subtypes, a threshold DEG in seven subtypes, and a top ten DEG in four cell subtypes (Excel Table S2-2). For *PEG10* at 7q21, we found variable results across 14 subtypes, with underexpression in seven and overexpression in four subtypes, as well as identification as a top ten threshold underexpressed DEG in only D16 CM-A1A2 (Excel Table S2-2). For *MEG3*, despite limited expression at all time points (Figure 3 and Figure S10D), we found *MEG3* as a top ten overexpressed threshold DEG in two subtypes: D00 PP-A1A2 and D19 EPDC (Excel Table S2-2). For these imprinted genes, we could not determine allelic expression due to the lack of informative coding SNV.

### 3.6. Cell Type-Specific Pathway Enrichment: Dysregulation of Gene Expression and Development of Cardiac Progenitors and Cardiomyocytes (Workflow Step-IIIA)

Using enrichment analysis of top cell type DEGs (Table 1, Figure S10A,C), we found evidence for the dysregulation of gene expression and developmental pathways by ORA (Excel Table S2-3) and GSEA (Excel Table S2-4). For most shared cell types (Table 1, Figure S10A,C), our results by ORA supported differences in gene regulation (Excel Table S2-3), involving dosage compensation, epigenetic regulation, and heterochromatin formation, attributed to underexpression of three DEGs: *LMNA*, *XIST*, and *NDN* in Patient cells. Specifically, D00 PP-B and D09B EPDC, were enriched for dosage compensation by the inactivation of the X chromosome (GO:0009048, GO:0007549), as expected, attributed to *XIST* underexpression. Similarly, D09B CP-B and D19 EPDCs were enriched for epigenetic regulation of gene expression (GO:0040029), attributed to the underexpression of up to three DEGs: *XIST*, *NDN*, and *LMNA*. For D19 EPDCs, the underexpression of *NDN* and *LMNA* also resulted in enrichment for heterochromatin formation (GO:0031507, GO:0070828).

Using the enrichment analysis of top DEGs, we also found evidence for differences in developmental pathways between Patient and Control CPs and CMs (Table 1, Figure S10A,C), attributed to DEG subsets encoding transcription factors, signaling proteins, and structural components of cardiac muscle cells and sarcomeres [54,56]. First, we found differences in CP subtypes at Day 9 by ORA (Excel Table S2-3). D09B CP-A was enriched for ventricular cardiac muscle cell development or differentiation (GO:0055015, GO:0055012), attributed to the underexpression of two DEGs, our DCM-study genes *LMNA* and *NKX2-5*. In contrast, for D09B CP-B, overexpression of two DEGs, *KRT19*, encoding a keratin intermediate filament protein, and *MYL9*, encoding a myosin light chain, resulted in enrichment for myofibril assembly (GO:0030239) and striated muscle cell development (GO:0055002).

**Figure 4:**
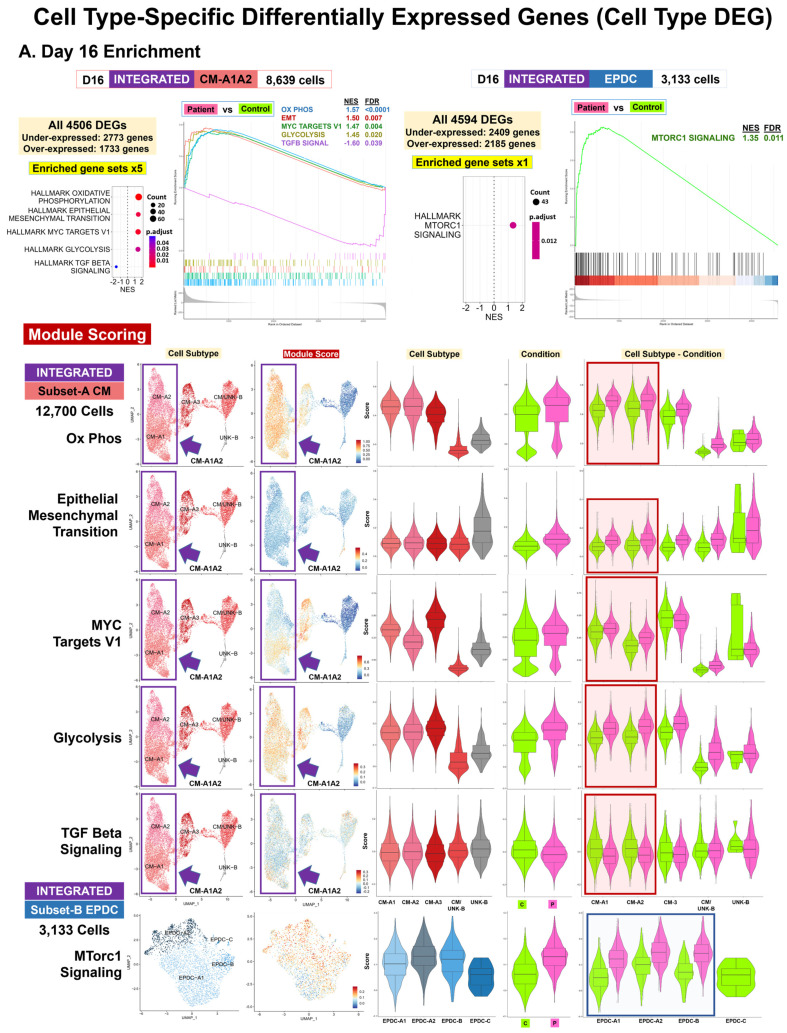

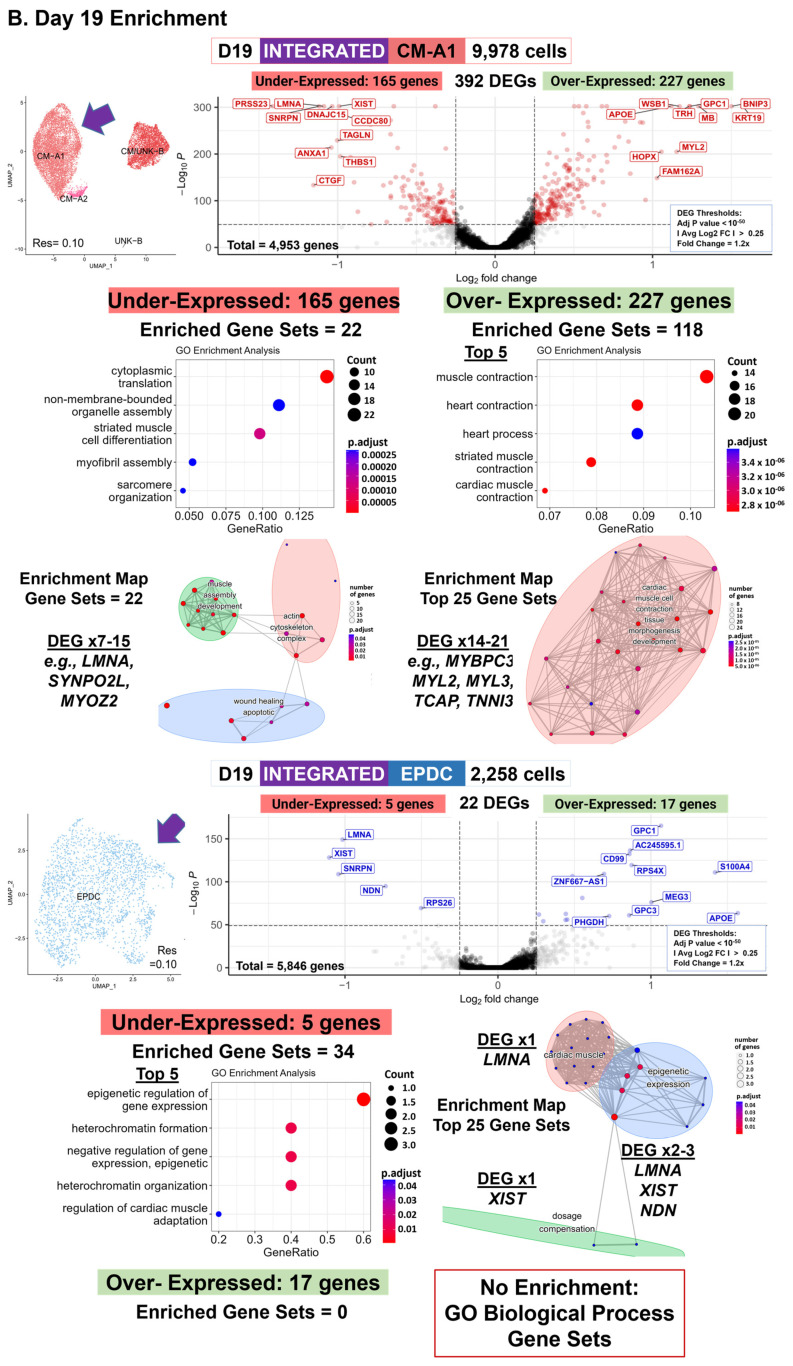
**Cell type-specific differentially expressed genes (cell type DEGs): enrichment.** Our comparative analyses (Patient vs. Control cells) included cell type-specific differential expression and enrichment using integrated data at four time points: D00, D09B, D16, and D19. We identified cell type DEGs in 14 pairs of ‘balanced’ cell types/subtypes, with 71,541 total cells. (**A**) Day 16 enrichment. Shown are GSEA [61] results using Cluster Profiler [62] (**top panel**) for 8639 CM-A1A2 cells and 3133 EPDC, with numbers of DEG and enriched MSigDB Hallmark gene sets, dot plots with the normalized enrichment score (NES) and adjusted *p*-values, and GSEA enrichment plots. We used module scoring in Seurat (**bottom panel**) to compare the expression patterns of key DEGs in enriched gene sets for Subset-A data (12,700 CM) and Subset-B data (3133 EPDC). For each gene set, shown are UMAP plots with cell subtype and module scores, as well as violin plots with boxplot by cell subtype, condition, and cell subtype–condition. Overall, these results at Day 16 support the dysregulation of TGF Beta signaling, EMT developmental pathway, OXPHOS and glycolysis metabolism, and cell proliferation (via MYC target genes) in Patient CMs and mTORC1 signaling in Patient EPDCs. (**B**) Day 19 enrichment. Shown are cell type DEG results using Seurat and EnhancedVolcano [63] (**top panels**) and ORA [64] results using Cluster Profiler [62] (**bottom panels**) for 9978 CM-A1 cells and 2258 EPDC with the UMAP plot of cell subtypes, number of threshold DEGs, and volcano plot [63] with top ten DEGs labeled, number of under/overexpressed DEGs and enriched GO biological processes gene sets, dot plots with gene ratio and adjusted *p*-values, and enrichment maps. Overall, these results at Day 19 support the dysregulation of cardiogenesis, with both underexpressed DEGs, including *LMNA*, and overexpressed DEGs, including sarcomere genes, in Patient CMs, as well as epigenetic/heterochromatin dysregulation, with underexpression of *XIST*, *NDN*, and *LMNA*, in Patient EPDCs.

For CMs at Days 16 and 19, our results supported differences in developmental and differentiation pathways (Table 1, Figure 4 and Figure S10A,C). For D16 CM-A1A2, among top enriched gene sets by ORA (Excel Table S2-3), we found muscle organ development (GO:0007517), attributed to underexpression of 13 DEG, including *NKX2-5* and *WNT2*, encoding a highly expressed WNT-signaling protein during cardiac differentiation [57] and cardiac muscle contraction (GO:0060048, GO:0006941) and actin filament-based movement (GO:0030048), attributed to the underexpression of 8 to 11 DEGs including *CACNA1D* and *CACNA1G*, encoding voltage-sensitive calcium channels [54]. For D16 CM-A1A2, we found muscle cell development and differentiation (GO:0055001, GO:0042692, GO:0051146) by ORA, attributed to overexpression of 23 to 31 genes, including four sarcomeric genes, *ACTC1*, *ACTN2*, *CSRP3*, and *MYH7*, as well as Hallmark EMT by GSEA (NES = 1.49, FDR = 0.007) (Excel Table S2-4), attributed to the upregulation of 23 DEGs, including the EMT genes *CDH2* and XL *TIMP1* [58]. For all CM-A subtypes, Patient cells had a higher median module score for EMT compared to the Control cells at Day 16 (Figure 4A and Figure S10E).

For D19 CM-A1 (Figure 4B), among the top enriched gene sets, we found striated muscle cell differentiation (GO:0051146) and myofibril assembly/sarcomere organization (GO:0030239, GO:0045214) by ORA (Excel Table S2-3), attributed to the underexpression of 7 to 15 DEGs, including *LMNA* and two genes, *SYNPO2L* and *MYOZ2*, encoding actin binding proteins. For D19 CM-A1, we found cardiac muscle and heart contraction (GO:0060048, GO:0006941, GO:0003015, GO:0060047, GO:0006936) by ORA, attributed to the overexpression of 14 to 21 DEGs including five sarcomere genes: *MYBPC3*, *MYL2*, *MYL3*, *TCAP*, and *TNNI3* and Hallmark myogenesis by GSEA (NES = 1.59, FDR = 0.0074) (Excel Table S2-4), attributed to the upregulation of 37 DEGs, including the XL *DMD* Dystrophin gene and seven sarcomere protein-encoding genes: *LDB3*, *MYBPC3*, *MYL2*, *MYL3*, *TCAP*, *TNNC1*, and *TNNT2*. For all CM-A subtypes, Patient cells had a higher median module score for myogenesis compared to the Control cells at Day 19 (Figure S10E).

### 3.7. Cell Type-Specific Pathway Enrichment: Dysregulation of Cell Signaling, Metabolism, and Proliferation (Workflow Step-IIIA)

Using enrichment analysis of top Cell Type DEG (Table 1, Figure S10A,C), we found evidence for dysregulation of signaling, metabolism, and cell cycle control by ORA (Excel Table S2-3) and GSEA (Excel Table S2-4). For signaling, our results included enriched pathways involving TGF Beta signaling for CM and mTORC1 signaling for EPDC at Day 16 (Figure 4A), attributed to DEG subsets encoding secreted proteins, regulatory factors, and downstream targets [54]. Among the top enriched gene sets in D16 CM-A1A2, we found the transmembrane receptor serine/threonine kinase signaling pathway (GO:0007178) by ORA (Excel Table S2-3) and Hallmark TGF Beta signaling by GSEA (NES = −1.60, FDR = 0.039) (Excel Table S2-4), attributed to the underexpression or downregulation of 7 to 13 DEGs, including *SMAD6*, *ID1*, *ID2*, and *ID3*, encoding negative regulatory factors [54]. For CM-A1 and A2, the Patient cells had a lower median module score for TGF Beta signaling compared to the Control cells at Day 16 (Figure 4A and Figure S10E). In contrast, D16 EPDC was enriched for Hallmark Mtorc1 signaling by GSEA (NES = 1.35, FDR = 0.011), attributed to the upregulation of 43 DEG, including the mTORC1 regulation gene *DDIT4* (*REDD1*) and the metabolic genes *HK2*, *PGK1*, *ENO1*, and *GAPDH* for glucose; *LDHA* for lactate; and *PHGDH* and *SHMT2* for amino acid metabolism. For EPDC-A1, A2, and B, Patient cells had a higher median module score for Mtorc1 signaling compared to Control cells (Figure 4A and Figure S10E).

Our enrichment results supported dysregulation in cell metabolism involving oxidative phosphorylation (OXPHOS), glucose, and nucleotides for different cell types and time points (Table 1, Figure S10A,C). At Days 0 and 9, our results supported decreased OXPHOS for Patient PP and CP and increased glycolysis for Patient EPDC (Figure S10C) compared to the Control cells, attributed to DEG subsets encoding key enzymes, components, and regulators of the electron transport chain (ETC) and glycolysis [54]. For D00 PP-A1A2, we found mitochondrial oxidative metabolism (GO:0019646, GO:0042773, GO:0042775, GO:0006120, GO:0022904) among the top enriched gene sets by ORA (Excel Table S2-3), attributed to three underexpressed DEGs: *DNAJC15*, *NDUFA3*, and *NDUFA6*. Similarly, D09B CP-A was enriched for Hallmark oxidative metabolism by GSEA (NES = −1.74, FDR = 0.00016) (Excel Table S2-4), attributed to the downregulation of 46 DEGs including multiple ETC-encoding genes. For D09B CP-A, the Patient cells had a lower median module score for oxidative metabolism compared to the Control cells (Figure S10E). In contrast, for D09B EPDC, we found glycolytic metabolism (GO:0061621, GO:0061718, GO:0006735) by ORA, attributed to the overexpression of *PFKP*, encoding phosphofructokinase in the first committing step of glycolysis.

At Day 16, our results for CMs supported (Figure 4A) metabolic differences, consistent with increased glucose metabolism and OXPHOS. For D16 CM-A1A2 using GSEA (Excel Table S2-4), we found Hallmark glycolysis (NES = 1.45, FDR = 0.020), attributed to the upregulation of 27 DEG, including *HK2* and *ENO2*, encoding enzymes for glucose metabolism and Hallmark oxidative metabolism (NES = 1.57, FDR = 3.86E−05), attributed to the upregulation of 72 DEGs, including multiple ETC-encoding genes and three DEGs overlapping glycolysis. For all CM-A subtypes, Patient cells had a higher median module score for both glycolysis and oxidative metabolism compared to the Control cells at Day 16 (Figure 4A and Figure S10E). Likewise, for D16 CM-A1A2 by ORA (Excel Table S2-3), we found mitochondrial oxidative metabolism (GO:0019646, GO:0042773), attributed to 16 overexpressed ETC-encoding genes, including *COX6A2*, *COX7A1*, and *CYCS*, not found by GSEA.

In contrast to CM at Day 16, our results for EPDCs supported increased glucose and nucleotide metabolism for the Patient, compared to the Control EPDCs, consistent with metabolic selection by lactate enrichment [59]. For D16 EPDCs (Figure S10C), we found purine ribonucleotide metabolism (GO:0009150, GO:0006163, GO:0072521, GO:0019693) and generation of precursor metabolites and energy (GO:0006091) by ORA (Excel Table S2-3), attributed to the upregulation of 11 to 12 DEGs, including *PFKP*, *TPI1*, *PKM*, and *GAPDH*, encoding enzymes for glycolysis and gluconeogenesis, and *TKT*, *HINT1*, and *PAICS*, encoding enzymes for nucleotide metabolism.

In addition, for CM at Day 16, we found differences in cell proliferation (Figure 4A). D16 CM-A1A2 was enriched for Hallmark Myc targets V1 by GSEA (NES = 1.47, FDR = 0.004) (Excel Table S2-4), attributed to the upregulation of 36 DEGs encoding targets of the MYC transcription factor that promote cell growth and proliferation, including ten spliceosome genes: *HNRNPA2B1*, *HNRNPA3*, *HNRNPD*, *HNRNPR*, *LSM7*, *SNRPD2*, *SNRPG*, *SRSF2*, *SRSF3*, and *TXNL4A* [54,60]. For CM-A1 and A2, the Patient cells had a higher median module score for Myc targets V1 compared to Control cells at Day 16 (Figure 4A and Figure S10E).

### 3.8. Single Lineage for Pluripotent Cells and Lineage Bifurcation for Cardiac Progenitors into Cardiomyocytes and EPDCs in Patient and Control Data (Workflow Step-IIIB)

**Figure 5:**
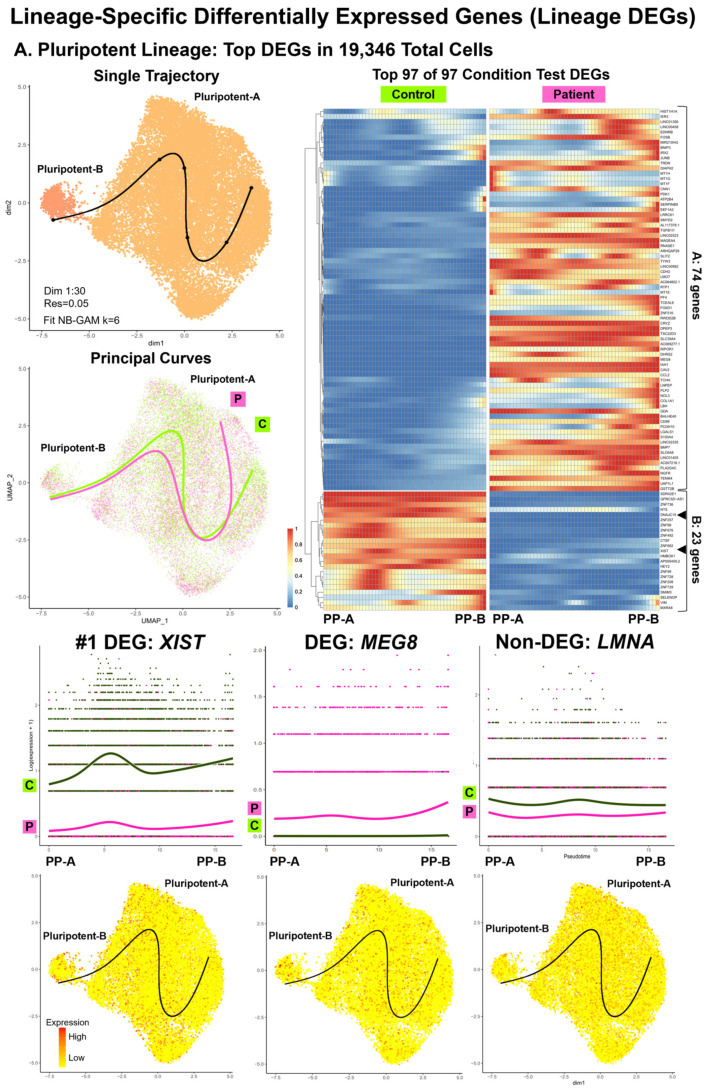

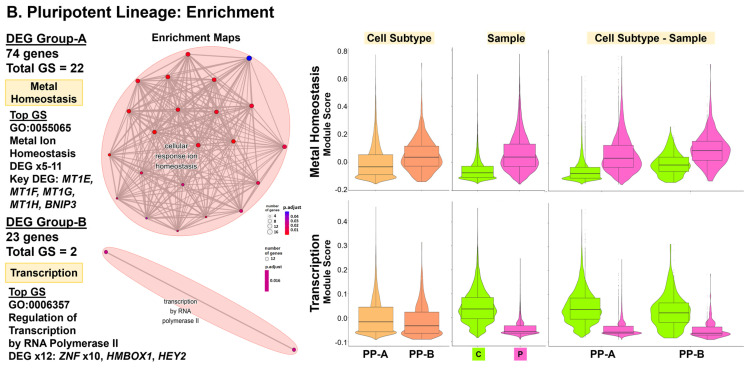
**Lineage-specific differentially expressed genes (lineage DEGs): top DEGs and enrichment for pluripotent lineage.** Our comparative analyses (Patient vs. Control) included lineage-specific differential expression and enrichment using integrated data of 18 shared subtypes (62,488 total cells). This included D00 pluripotent (PP) subtypes for the PP lineage (19,346 total cells), a single trajectory from PP-A to PP-B. Shown are UMAP plots with trajectory and principal curves using Slingshot [65] (**top left**), a heat map by condition, along pseudotime points using pheatmap and condiments [51] (**top right**), and smoother plots and gene count plots using TradeSeq [66] (**bottom**). (**A**) PP Lineage: top DEGs in 19,346 total cells. We identified 97 conditional test DEGs that clustered into two groups: A (74 genes) and B (23 genes). Top DEGs included *XIST* (#1), underexpressed, and the imprinted gene *MEG8*, overexpressed in Patient PPs, along all pseudotime points. *LMNA* was not found as a lineage DEGs; however, *LMNA* expression was lower in Patient compared to Control PPs at all pseudotime points. (**B**) PP lineage: enrichment. Shown are ORA enrichment results using Cluster Profiler [62] (**left panel**) with DEG numbers by group and enriched GO BP gene sets (GSs), top GSs, key DEGs, and enrichment maps. To compare expression of key DEGs in enriched GSs, we used Seurat Module Scoring (**right panel**) and violin plots with boxplots. Results support dysregulation of metal homeostasis, with overexpression of 5 to 11 genes from DEG Group-A, including four metallothionein protein (*MT*) genes and RNA pol II transcription, with underexpression of 12 DEGs from DEG Group-B, including 10 Zinc-finger protein (*ZNF*) genes in Patient PPs.

**Figure 6:**
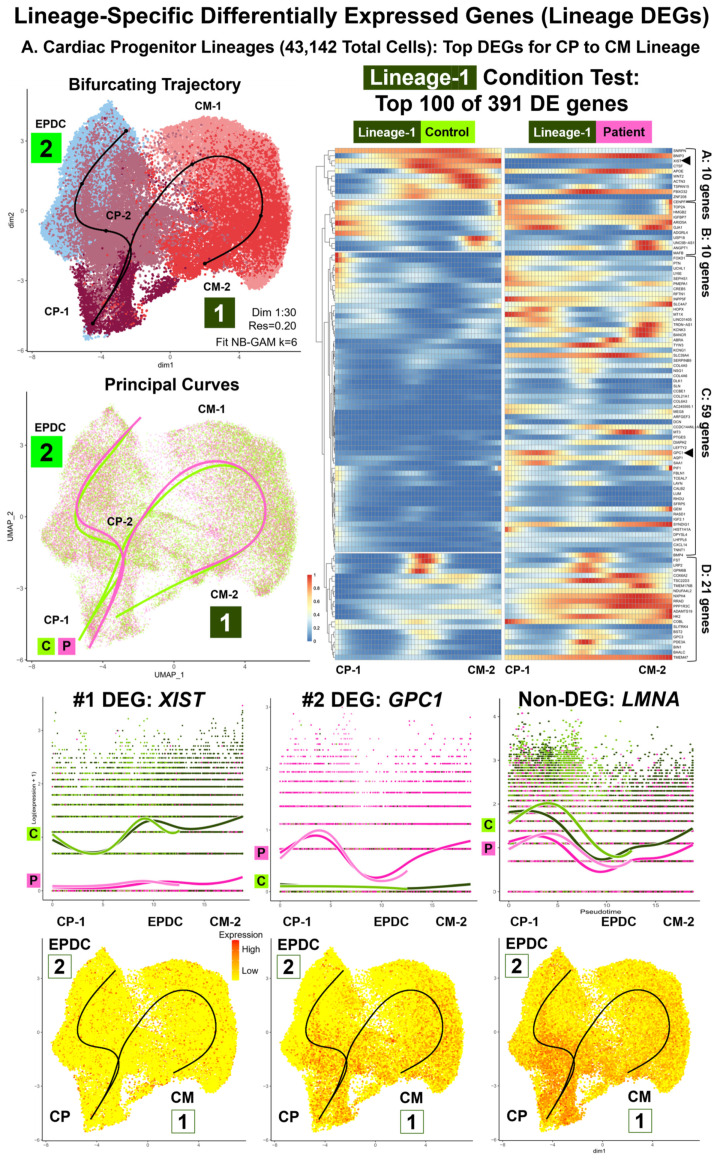

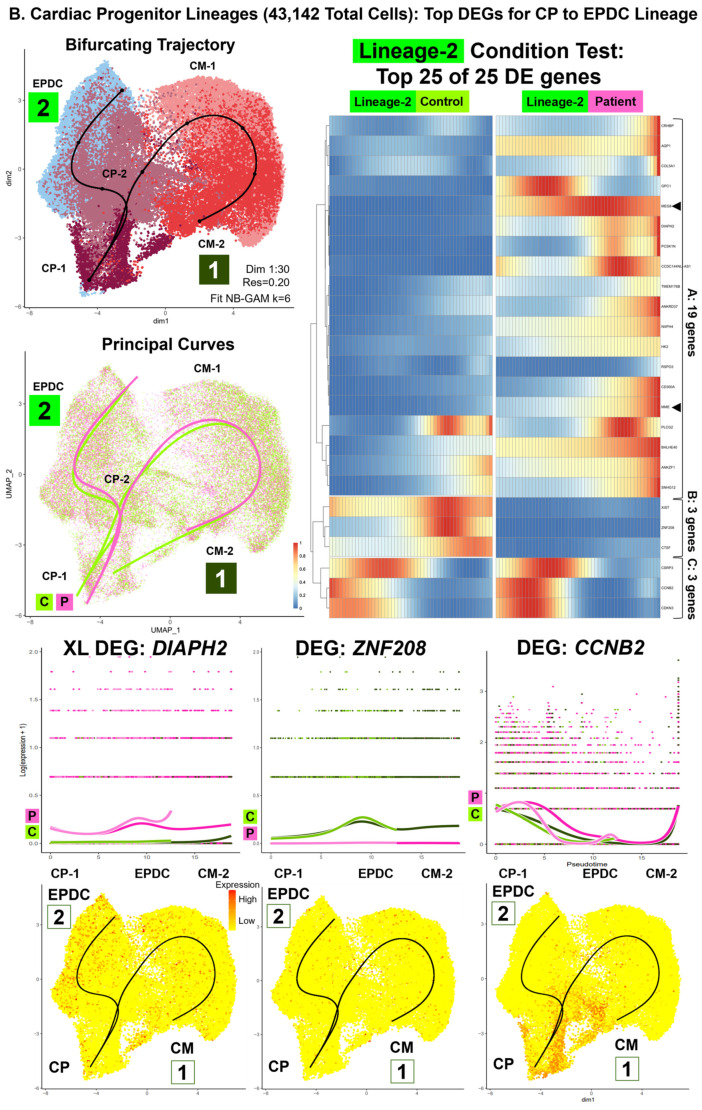
**Lineage-specific differentially expressed genes (lineage DEGs): cardiac progenitor lineages—top DEGs.** Our comparative analyses (Patient vs. Control cells) included lineage-specific differential expression and enrichment using integrated data of 18 shared subtypes (62,488 total cells). This included D09B, D16, and D19 cardiac progenitor (CP), CM, and EPDC subtypes for the CP lineages (43,142 total cells), a bifurcating trajectory starting at CP-1 and ending at CM-2 (Lineage-1: CPs to CMs) and EPDCs (Lineage-2: CPs to EPDCs). Shown are UMAP plots with trajectory and principal curves using Slingshot [65] (**top left**), heat map by condition, along pseudotime points, using pheatmap and condiments [51] (**top right**), and smoother plots and gene count plots, using TradeSeq [66] (**bottom**). (**A**) CP Lineage-1: CPs to CMs—Top DEG. We identified 391 total conditional test DEGs, with the top 100 DEGs clustered into four groups: A (10 genes), B (10 genes), C (59 genes), and D (21 genes). The top DEGs included *XIST* (#1), underexpressed, and *GPC1* (#2), overexpressed in Patient cells, along all pseudotime points. (**B**) CP Lineage-2: CPs to EPDCs—top DEGs. We identified 25 total conditional test DEGs that clustered into three groups: A (19 genes), B (3 genes), and C (3 genes). Top DEGs included XL *DIAPH2*, overexpressed, and *ZNF208*, underexpressed in Patient cells, along all pseudotime points and cell cycle gene *CCNB2*, overexpressed in Patient CP cells. Similar to the PP lineage, *LMNA* was not identified as a DEG in both CP lineages with lower expression in Patient compared to Control cells at all pseudotime points.

**Figure 7:**
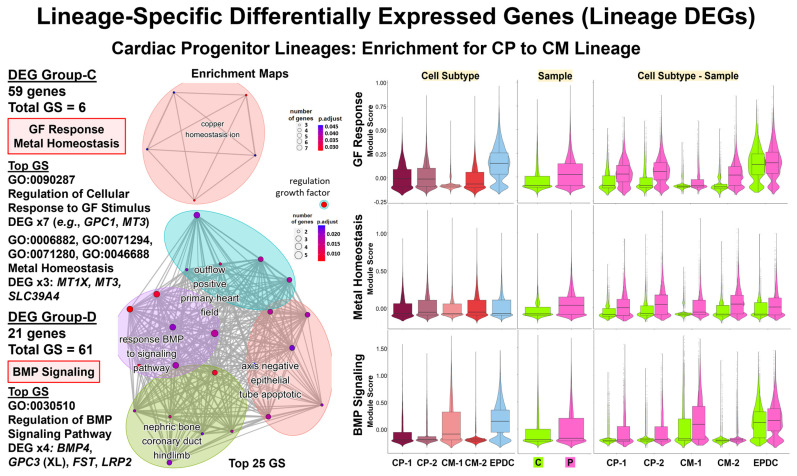
**Lineage-specific differentially expressed genes (lineage DEGs): cardiac progenitor lineage-enrichment.** For Lineage-1: CP into CM, shown are ORA enrichment results using Cluster Profiler [62] (**left panel**) with numbers of DEGs by group and enriched GO BP gene sets (GSs), top GSs, key DEGs, and enrichment maps. To compare the expression of key DEGs in enriched GSs, we used Module Scoring in Seurat (**right panel**) and violin plots with boxplots. For Lineage-1: CPs into CMs, these results support dysregulation of Growth Factor (GF) Response with overexpression of seven Group-C DEGs, including *GPC1* and *MT3*, metal homeostasis with overexpression of three Group-C DEGs, including two metallothionein proteins (*MT1X* and *MT3*) genes, and BMP signaling pathway, with overexpression of four Group-D DEGs: *BMP4*, XL *GPC3*, *FST*, and *LRP2*.

For the single subset data (n = 7: 22,326 total cells) for selected cell subtypes of one condition (Table S10) and for the integrated subset data (n = 2: 62,488 total cells) of 18 paired subtypes (Figure S7A, Table S11), we conducted trajectory inference, testing for lineage DEGs, and enrichment analyses (Table 1, Figure 5, Figure 6, Figure 7, Figures S11 and S12, Excel Table S3). The results for the control single subset data included three single trajectories (PP-A to PP-B, CMESO to CP, CP-A to CM-A) and one bifurcating trajectory (ME to CMESO and ENDO) (Figure S11A). The results for the integrated subset data consisted of two parts: D00 PP cells (19,346 total cells) with a single trajectory from PP-A to PP-B (PP lineage) (Figure 5 and Figure S12B,C), and D09B, D16, and D19 CP, CM, and EPDC cells (43,142 total cells) with a bifurcating trajectory starting at CP-1 and ending at CM-2 (CP Lineage-1) and EPDC (CP Lineage-2) (Figure 6, Figure 7 and Figure S12B,D). Between conditions, we found differences in topology and progression for the PP and CP lineages and in the rate of differentiation between the CP Lineage-1 for CMs and the CP Lineage-2 for EPDCs (Figure S12A).

### 3.9. Lineage-Specific Differential Expression: Non-Coding RNA XIST, Sixth Imprinted Gene MEG8, and Cell-Surface Glycoprotein Encoding Gene GPC1 (Workflow Step-IIIB)

Across three lineages, we found equal proportions of Patient (50%) and Control (50%) cells (Table S11) and variable numbers of lineage DEGs between conditions (Figure S12A). For example, we identified fewer total DEGs between conditions (CT lineage DEGs) for the PP lineage (924 DEGs) compared to two CP lineages (2335 global DEGs). Between the two CP lineages, we found fewer total DEGs between conditions (CT lineage DEGs) for CP Lineage-1 into CMs (1795 DEG) compared to CP Lineage-2 into EPDCs (2216 DEG).

For all three lineages, consistent with our cell type DEG results, we found the *XIST* non-coding RNA gene as the highest ranked or among top 25 CT lineage DEGs (Excel Table S3-2). For all three lineages, we found underexpression of *XIST* in Patient compared to Control PP cells along all pseudotime points (Figure 5A and Figure 6A). In addition, we found a sixth imprinted gene, *MEG8*, at 14q32.2 differentially expressed between the Patient and Control cells [54]. For the PP lineage, we found *MEG8* among the top CT lineage DEGs and for the CP to EPDC lineage, as the highest ranked CT lineage DEGs (Excel Table S3-2), with overexpression of *MEG8* in the Patient, compared to the Control PP cells, along all pseudotime points (Figure 5A). For both CP lineages (Excel Table S3-3), top CT lineage DEGs included *GPC1* encoding Glypican Proteoglycan 1, a cell-surface glycoprotein for growth regulation [54], with overexpression of *GPC1* in the Patient, compared to Control cells, along all pseudotime points (Figure 6A). For all three lineages, *LMNA* expression was lower in the Patient, compared to the Control cells, at most pseudotime points (Figure 5A and Figure 6A); however, the top CT lineage DEGs did not include *LMNA*.

### 3.10. Lineage-Specific Pathway Enrichment: Regulation of Gene Expression, Cell Signaling, Proliferation, and Homeostasis (Workflow Step-III: Lineage Enrichment)

Using enrichment analysis of the top Lineage DEGs (Table 1, Figure 5, Figure 6, Figure 7 and Figure S12A), we found evidence for the dysregulation of pathways consistent with our analyses of the top cell type DEGs. For the PP lineage, we found four enriched gene sets, compared to none for the CP lineages by ORA and GSEA. For the CP lineages, we found 19 enriched gene sets for Lineage-1 for CMs, compared to none for Lineage-2 for EPDCs by ORA and GSEA. Furthermore, we found additional enriched gene sets after clustering the top Lineage DEGs into smaller groups by hierarchical cluster analysis.

Consistent with our cell type DEG results, we also found evidence for differences in gene regulation for two lineages: the PP lineage and the CP to EPDC lineage (Table 1, Excel Table S3-4). The PP lineage (Figure 5B and Figure S12C) was enriched for regulation of transcription by RNA polymerase II (GO:0006357, GO:0006366) using CT lineage DEG Group-B, attributed to the underexpression of 12 genes encoding DNA-binding proteins: 10 Zinc-finger proteins (ZNFs), *HMBOX1*, and *HEY2* [54]. For PP-A and PP-B, the Patient cells had a lower median module score, based on expression of the 12 genes compared to the Control cells (Figure 5B). For the CP to EPDC lineage, the top DEGs included *ZNF208*, underexpressed in the Patient cells along all pseudotime points (Figure 6B). For the CP to EPDC lineage, we also found enrichment for dosage compensation (GO:0007549, GO:0009048) using the CT Lineage-2 DEG Group-B, attributed to *XIST* underexpression (Figure S12D).

In each CP lineage, we also found evidence for differences in cell signaling and proliferation by ORA (Table 1, Excel Table S3-4). The CP-to-CM lineage (Figure 7 and Figure S12D) was enriched for regulation of the BMP signaling pathway (GO:0030510, GO:0030509, GO:0071773, GO:0071772) using the CT Lineage-1 DEG Group-D, attributed to overexpression of four DEGs: *BMP4*, XL *GPC3*, *FST*, and *LRP2*, encoding four regulatory proteins [54]. For CM-1, the Patient cells had a greater median module score based on *BMP4*, *GPC3*, *FST*, and *LRP2* expression, compared to the Control cells (Figure 7), consistent with our cell type DEG results for TGF Beta signaling in D16 CM-A1A2 (Figure 4A). The CP-to-CM lineage was also enriched for regulation of cellular response to growth factor stimulus (GO:0090287) using the CT Lineage-1 DEG Group-C, attributed to the overexpression of seven DEGs such as *GPC1* and *MT3*, encoding Metallothionein 3, a growth inhibitory factor [54]. For CP-1, CP-2, and CM-2 cells, the Patient, compared to the Control cells, had a greater median module score based on the expression of these seven DEGs (Figure S12D). For the CP to EPDC lineage (Figure S12C), we found enrichment for the regulation of cyclin-dependent protein serine/threonine kinase activity (GO:0000079, GO:0071900, GO:1904029) using the CT Lineage-2 DEG Group-C, attributed to overexpression, primarily in CP cells, of two DEGs: *CDKN3* and *CCNB2*, encoding Cyclin B2 in TGF Beta-mediated cell cycle control [54].

Finally, for two lineages, the PP lineage and the CP Lineage-1, our results included evidence for differences in homeostasis by ORA (Table 1, Excel Table S3-4). The PP lineage (Figure 5B and Figure S12C,D) was enriched for zinc/metal ion homeostasis (GO:0055065, GO:0055069, GO:0072507, GO:0006875, GO:0072503) using the CT lineage DEG Group-A, attributed to the overexpression of 5 to 11 DEGs, including *MT1E*, *MT1F*, *MT1G*, and *MT1H*, encoding four metallothionein proteins that bind heavy metals, and *BNIP3*, encoding BCL2 Interacting Protein 3, a pro-apoptotic factor [54]. For PP-A and PP-B, the Patient, compared to the Control cells, had a higher median module score, based on the expression of 11 genes (Figure 5B). Likewise, the CP-to-CM lineage (Figure 7 and Figure S12D) was enriched for metal homeostasis and response to zinc (GO:0006882, GO:0071294) and copper (GO:0071280, GO:0046688) ions using the CT Lineage-1 DEG Group-C, attributed to the overexpression of three DEGs, such as MT1X and MT3, also encoding metallothionein proteins [54]. Similar to the PP lineage, the Patient, compared to the Control cells, had a higher median module score based on expression of the five genes (Figure 7).

### 3.11. Decreased Lamin A/C Protein Levels for Patient Cells at Day 19

**Figure 8:**
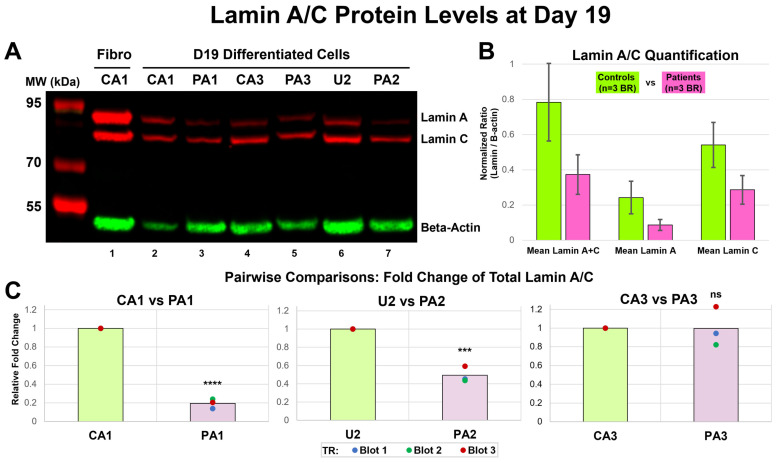
**Lamin A/C protein levels at Day 19.** (**A**) Western blot (WB). We performed immunoblotting on lysates from differentiated cells with three biological replicates (BRs) at Day 19 for Patient (PA1, PA2, and PA3) and Control (CA1-B, U2, and CA3) iPSC lines. The image (Blot 3) shown was taken using the Azure c600 imaging system with fluorescent detection in red for Lamin A (74 kDa) and Lamin C (62 kDa) and in green for Beta-Actin (42 kDa) from Control fibroblast (CA1) (lane 1, positive control) and the three age- and sex-matched pairs: CA1 and PA1 (lane 2 and 3), CA3 and PA3 (lane 4 and 5), and U2 and PA2 (lane 6 and 7). Positions and sizes of molecular weight (MW) marker proteins are given on the left margin. (**B**) Lamin A/C quantification: Controls vs. Patients. We measured the band volume (BV) for Lamin A, Lamin C, and Beta-Actin using AzureSpot software (v2.2.167) and calculated normalized ratio (NR = lamin BV/Beta-Actin BV) of each sample (CA1-B, PA1, U2, PA2, CA3, and PA3) for three WB experiments (Blot 1–3) as technical replicates (TRs). Comparison of the Control (n = 3 BRs: CA1-B, U2, CA3) vs. Patient (n = 3 BRs: PA1, PA2, PA3) data showed a decrease in mean protein levels for Lamin A, Lamin C, Lamin A + C in the Patient, compared to Control cells; these differences were not statistically significant (*p* > 0.05). Error bars denote SD of the mean protein levels of BRs. (**C**) Pairwise comparisons: fold change of total lamin A/C. For each matched pair, we calculated fold change for Lamin A + C by dividing the Control and Patient protein levels by the Control protein level. Each chart shows the fold change from three TRs (Blot 1–3) as individual data points and mean fold change shown as column. Statistical analysis of the fold change revealed a significant decreases in protein level for Lamin A + C of PA1 (**** *p* < 0.0001) and PA2 (*** *p* < 0.001) but no significant difference for PA3 (*p* = 0.98) compared to matched Control.

To compare Lamin A/C protein levels, we differentiated three iPSC pairs as biological replicates (BRs), three Patient-derived iPSCs (PA1, PA2, and PA3) and three Control iPSCs (CA1-B, U2, and CA3); obtained protein lysates from differentiated cells at Day 19; repeated Western blot three times (TR blots x3) (Figure 8A and Figure S13); and quantified the protein levels for Lamin A, Lamin C, and total Lamin A + C as normalized ratios (NRs) (Table S12A). A comparison of the Control (n = 3: CA1-B, U2, CA3) vs. Patient (n = 3: PA1, PA2, PA3) data showed a decrease in mean protein levels for Lamin A, Lamin C, and total Lamin A + C in the Patient, compared to the Control cells (Figure 8B, Table S12B); however, these differences were not statistically significant (Lamin A + C: t = 1.66, *p* = 0.172, Lamin A: t = 1.59, *p* = 0.187, Lamin C: t = 1.68, *p* = 0.168). In our pairwise comparisons, statistical analysis of fold change revealed a significant decrease in the protein levels for Lamin A + C for PA1 (−81%), compared to CA1 (t = 27.1, *p* < 0.0001), and PA2 (−51%), compared to U2 (t = 10.2, *p* < 0.001). However, there was no significant difference for PA3 (−0.34%) compared to CA3 (t = 0.028, *p* = 0.98) (Figure 8C, Table S12C). Our analyses also showed a similar, acceptable CV for all the pairs (mean CV = 21.6% ± 4.5%) and % changes twice as large the CV for PA1 and PA2, but not for PA3 (Table S12C).

**Table 1:** Enrichment results for top cell type and lineage DEGs.

Cell Type/Subtype	Process/Pathway ^a^	Method	Expr	#DEGs ^b^	Key Cell Type DEGs
D00 PP-A1A2	OXPHOS	ORA	UNDER	3	*DNAJC15*, *NDUFA3*, *NDUFA6*
D00 PP-B	Dosage compensation	ORA	UNDER	1	*XIST* (XL)
D09B CP-A	OXPHOS	GSEA	UNDER	46	Electron Transport Chain genes
	Muscle dev/diff	ORA	UNDER	2	*LMNA*, *NKX2-5*
D09B CP-B	Epigenetic gene expr	ORA	UNDER	2	*XIST* (XL), *NDN*
	Muscle/myofibril dev	ORA	OVER	2	*KRT19*, *MYL9*
D09B EPDC	Dosage compensation	ORA	UNDER	1	*XIST* (XL)
	Glycolysis	ORA	OVER	1	*PFKP*
D16 CM-A1A2	TGF Beta signaling	GSEA	UNDER	7	*SMAD6*, *ID1*, *ID2*, *ID3*
	RSTK signaling	ORA	UNDER	13	*ID1*
	Muscle contraction	ORA	UNDER	8–11	*CACNA1D*, *CACNA1G*
	Muscle development	ORA	UNDER	13	*NKX2-5*, *WNT2*
	Glycolysis	GSEA	OVER	27	*HK2*, *ENO2*
	OXPHOS	GSEA	OVER	72	Electron Transport Chain genes
	OXPHOS	ORA	OVER	16	Electron Transport Chain genes
	Myc targets	GSEA	OVER	36	Spliceosome genes x10, *TYMS*
	Muscle dev/diff	ORA	OVER	23–31	*ACTC1*, *ACTN2*, *CSRP3*, *MYH7*
	EMT	GSEA	OVER	23	*CDH2*, *TIMP1* (XL)
D16 EPDC	mTORC1 signaling	GSEA	OVER	43	*HK2*, *PGK1*, *ENO1*, *GAPDH*, *LDHA*, *PHGDH*, *SHMT2*
	Nucleotide metabolism	ORA	OVER	11–12	*PFKP*, *TPI1*, *PKM*, *GAPDH*, *TKT*, *HINT1*, *PAICS*
	Precursor Metabolites	ORA	OVER	12	*PFKP*, *TPI1*, *PKM*, *GAPDH*, *TKT*
D19 CM-A1	Muscle differentiation	ORA	UNDER	7–15	*LMNA*, *SYNPO2L*, *MYOZ2*
	Myogenesis	GSEA	OVER	37	*LDB3*, *MYBPC3*, *MYL2*, *MYL3*, *TCAP*, *TNNC1*, *TNNT2*, *DMD* (XL)
	Muscle contraction	ORA	OVER	14–21	*MYBPC3*, *MYL2*, *MYL3*, *TCAP*, *TNNI3*
D19 EPDC	Epigenetic gene express	ORA	UNDER	3	*LMNA*, *XIST* (XL), *NDN*
	Heterochromatin form	ORA	UNDER	2	*LMNA*, *NDN*
**Lineage**	**Process/Pathway ^a^**	**Method**	**Expr**	**#DEGs ^b^**	**Key Condition Test Lineage DEGs**
PP Lineage: PP-A to PP-B	Transcription	ORA	UNDER	12	Zinc-finger protein (*ZNF*) genes x10, *HMBOX1*, *HEY2*
	Metal homeostasis	ORA	OVER	5–11	*MT1E*, *MT1F*, *MT1G*, *MT1H*, *BNIP3*
CP Lineage-1: CP to CM	Growth Factor response	ORA	OVER	7	*GPC1*, *MT3*, *FOXD1*, *PMEPA1*, *CCBE1*, *DCN*, *SFRP5*
	BMP signaling pathway	ORA	OVER	4	*BMP4*, *GPC3* (XL), *FST*, *LRP2*
	Metal homeostasis	ORA	OVER	3	*MT1X*, *MT3*, *SLC39A4*
CP Lineage-2: CP to EPDC	Dosage compensation	ORA	UNDER	1	*XIST* (XL)
	Cyclin kinase activity	ORA	OVER	2	*CCNB2*, *CDKN3*

^a^ Enriched gene set using Cluster Profiler [62]: gene ontology biological process by ORA [64] or molecular signatures database Hallmark gene set by GSEA [61]. ^b^ Number (#) of DEG in enriched gene set by ORA or in leading edge subset by GSEA. Abbreviations: DEG, differentially expressed genes; expr, expression; PP, pluripotent; CP, cardiac progenitors; EPDC, epicardium-derived cells; CM, cardiomyocyte; OXPHOS, oxidative phosphorylation; dev, development; diff, differentiation; EMT, epithelial-mesenchymal transition; RSTK, receptor serine/threonine kinase; form, formation; ORA, over-representation analysis; GSEA, gene set enrichment analysis; UNDER, underexpressed; OVER, overexpressed; XL, X-linked.

## 4. Discussion

To investigate possible molecular mechanisms of *LMNA*-related DCM due to a *c.357-2A>G* splice-site mutation, we used a Patient-derived iPSC model of CM differentiation, serial scRNA-seq studies, and a comprehensive bioinformatics workflow. Our main findings include: 1. model complexity with heterogenous cell types, possible subtypes, and lineages after sequential QC data processing and multi-level analyses; 2. differential expression of genes, including *LMNA* and enriched gene sets for cell signaling, cardiogenesis, and EMT involved in gene regulation and cell development in specific *LMNA*-mutant cell types and lineages; 3. differential expression of XL genes, including *XIST*, six imprinted genes, and gene sets for regulation of metabolism, homeostasis, and proliferation. In our iPSC-derived model for Lamin A/C haploinsufficiency, our findings support a possible molecular mechanism involving cell type and lineage-specific dysregulation of gene expression in epigenomic developmental programs for PPs, CMs, and EPDCs. We also discuss the challenges of data interpretation in our iPSC-derived model.

**Complexity of iPSC-derived model and comparisons to previous serial scRNA-seq studies of normal CM differentiation.** After iPSC validation, we differentiated one *LMNA*-mutant line derived from an affected female (Patient) and two non-mutant lines derived from her unaffected sister (Control) for scRNA-seq. After sequential QC data processing, we identified 110,521 total high-quality cells. We conducted multi-level analyses of high-quality cells by unsupervised clustering and annotation using known markers (88 total genes) to identify main cell types and possible cell subtypes in single-sample, merged, and integrated data, as well as subset data. Here, we compared our results to previous scRNA-seq studies [27,28,29,30,31,32,33,34,35,36,67].

For our Control samples (65,324 high-quality cells), collected across all seven time points (D00 to D30), we identified ten main cell types: PP, ME, CMESO, ENDO, ECTO, ENDOTH, CP, CM, EPDC, and UNK. These main cell types are consistent with seven serial scRNA-seq studies on control human iPSCs [29,30,32,33,35,36] and human ESCs [31], differentiated into CMs using the Wnt small molecule method. One exception was ECTO, identified as a main cell type in only two [30,33] of seven studies. The absence of ECTO in five studies [29,32,33,35,36] may be due to the small proportion of ECTO cells (2% for D09B CA1), the lack of cell annotation markers, such as *PAX6* or *SOX1* [68], and/or biological variability of different control iPSC lines.

By subclustering, we identified many possible cell subtypes, including four PP subtypes, two CP subtypes, two CM subtypes, and six further differentiated CM and EPDC subtypes at Day 30. Among our PP subtypes at Day 0, the predominant PP-A subtype (88%) represents a combined cluster, after regression of CC scores of cells, of two reported main PP subpopulations: Core and Proliferative PPs [27,28]. For our three smaller subtypes, PP-B (3%) and PP-C (6%) might be similar to the reported smaller PP subpopulations: early and late primed for differentiation [27], while PP-D (3%) comprises partially differentiated cells with lower expression of a core PP marker *POU5F1* [69] and the undifferentiated cell marker *CMND* [70]. Within our CP cell type at Day 9A and 9B, CP cells subclustered into subtype CP-A, with a higher expression of *HAND1* and *HAPLN1* and a similar expression pattern to early CMs, and into subtype CP-B, with a higher expression of *GATA4* and *HAND2* and a similar expression pattern to early EPDCs. Thus, these two subtypes comprise distinct progenitor cells for CMs (CP-A) and EPDCs (CP-B), similar to two reported progenitor subpopulations at Day 5: the CM precursor (D5:S1) and the cardiovascular progenitor (D5:S3) [29].

We also identified possible cell subtypes for CMs and EPDCs. Within our CM cell type at Days 9B, 16, and 19, cells subclustered into subtype CM-A (70%), with a higher expression of multiple CM markers and Ribosomal Protein Genes, and subtype CM/UNK-B (30%), with a lower expression of most CM markers, except for *TTN*, and greater levels of %Mt. The predominant CM subtype, CM-A, is consistent with reported single CM populations, committed CMs (D15:S2) [29], and D11-15 early CMs [36]. In contrast, subtype CM/UNK-B is unique to our study, likely due to cell type-specific QC processing; we used a high %Mt threshold level for low-quality cells only for non-CM cells because of the higher Mt content in heart tissues [71,72]. Thus, our approach allowed the identification of the subtype CM/UNK-B, cells typically discarded prior to annotation. The subtype CM/UNK-B will need to be evaluated further as either low-quality/dying CM or a possible unique CM subtype characterized by a high mitochondrial content. Finally, at Day 30, we identified later-differentiated CM subtypes: ATR-CM and VENTR-CM. One of three similar scRNA-seq studies at Day 30 reported six distinct cell populations as atrial and ventricular cells using the Fluidigm C1 system for 43 *TNNT2*+ *ACTC1*+ subclustered cells [30]. Without subclustering at Day 30, two other studies reported a single CM cell type as late CMs [36] and as definitive CMs (D30:S2) [29]. Our results demonstrate the importance of subcluster analyses for CMs and later time points for identification and further analysis of differentiated subtypes [34].

Subcluster analyses for the EPDCs at Day 30, without metabolic enrichment, identified four possible subtypes: EPI, CFIBRO, VSM, and an unspecified EPDC subtype. These subtypes were not observed for EPDC at earlier time points, at Day 16 and 19, with metabolic enrichment. The three similar scRNA-seq studies at Day 30 did not report these EPDC subtypes after metabolic enrichment [29,30,36]. Similar to CMs, two studies that did not perform subcluster analyses reported a single EPDC cell type, identified as epicardial cells [36] and non-contractile cells (D30:S1) [29]. Taken together, our results provide, first, additional evidence for the continued presence of EPDCs [67] despite metabolic enrichment for CMs by glucose deprivation/lactate enrichment [59] and second, at later time points, four possible EPDC subtypes requiring further analysis.

After the identification of cell types and subtypes, we analyzed single and paired subset data using Slingshot and found multiple lineages with single trajectories and two bifurcating trajectories. For the Control single-subset data, we identified three single trajectories (PP-A to PP-B, CMESO to CP, CP-A to CM-A) and one bifurcating trajectory (ME to CMESO and ENDO). For the paired subset data of primarily “balanced” subtypes (62,488 total cells), we also identified a single trajectory at Day 0 (PP-A to PP-B) for both the Control and Patient cells and a second bifurcating trajectory (CPs into CMs and EPDCs). Our first lineage bifurcation at Day 2 (ME into CMESO and ENDO) is similar to the reported lineages using different trajectory methods, including Scdiff [29], Monocle 2 [31], and STREAM [36]. Our second lineage bifurcation of CPs at Day 9 (CPs into CMs and EPDCs) is consistent with previous reported lineages: cardiac precursor cells (D5:S1 and D5:S3) into committed CMs (D15:S2) and non-contractile cells (D15:S1) [29], D5 CPs into D7-D15 CMs and CFIBRO [35], and D5-D7 late CPs into D11-D15 early CMs and D5-D15 epicardial progenitors/epicardial cells [36]. Overall, our results for main cell types, several subtypes, and lineages with at least two bifurcations are consistent with previous scRNA-seq studies, supporting the validity of our complex iPSC-derived model.

**Differential expression of *LMNA* and gene sets for cell signaling, cardiogenesis, and EMT involved in epigenetic gene regulation and cell development for CMs and EPDCs with Lamin A/C haploinsufficiency.** Using integrated data, we conducted comparative analyses that identified two types of DEGs for the Patient, compared to the Control cells: cell type DEGs and lineage DEGs. We identified the cell type DEGs using four shared cell types (PPs, CPs, CMs, EPDCs) in 14 primarily ‘balanced’ subtypes (71,541 total cells). We identified the lineage DEGs using 18 paired subtypes (62,488 total cells) that derived three lineages: a single trajectory from PP-A into PP-B at Day 0, and a bifurcating trajectory from CPs into CMs (CP Lineage-1) and EPDCs (CP Lineage-2) across Days 9, 16, and 19. Using the top cell type and lineage DEGs, pathway enrichment by ORA and GSEA provided evidence for the dysregulation of multiple biological processes and important insights into *LMNA*-related DCM pathogenesis.

Among top cell type DEGs, we found that our DCM study gene, *LMNA*, was underexpressed in the Patient cells compared to the Control cells for shared cell types and lineages. In the Control PPs at Day 0, we detected limited *LMNA* expression, whcih increased along normal CM differentiation, as reported [22,23,24]. In the Patient PPs, we observed a similar pattern but at lower levels, with the greatest differences in *LMNA* expression at Day 19 CM-A1 and EPDC. We confirmed decreased Lamin A/C protein expression at Day 19 by WB, using two additional affected patients from the family, with matched controls. In addition, for all shared cell types detected, we found evidence for primarily monoallelic expression of the normal *LMNA* allele in Patient cells, similar to our previous results on *LMNA*-mutant dermal fibroblasts derived from the study family [49]. Our results support a molecular mechanism of *LMNA* haploinsufficiency for both cell types, CMs and EPDCs, in our iPSC-derived model.

To evaluate possible effects of *LMNA* haploinsufficiency and other top cell type DEGs, we used pathway enrichment, which provided evidence for differences in the regulation of epigenetic gene expression and cell development between conditions. First, the EPDC progenitor cells at Day 9 (CP-B) were enriched for epigenetic gene expression, attributed to the decreased expression of the non-coding *XIST* RNA gene that initiates XCI [52] and *NDN*, the paternally expressed imprinted gene [73], and for muscle development, attributed to the increased expression of keratin and myosin light chain protein-encoding genes, *KRT19* and *MYL9.* EPDC at Day 19 was also enriched for epigenetic expression and heterochromatin formation, attributed to the decreased expression of *LMNA*, *NDN*, and/or *XIST*.

Using the top lineage DEG pathway enrichment, we found evidence for differences in the regulation of transcription in all three lineages between conditions. The PP-A to PP-B lineage was enriched for the regulation of transcription by RNA polymerase II, with underexpression of 12 genes, including ten Zinc-finger protein (ZNF) genes, such as *ZNF208* [54]. For both CP lineages, our analysis also identified *ZNF208* as the top global lineage DEGs with limited expression in the Patient, compared to the Control CPs, along CM and EPDC lineages. Zinc-finger proteins bind DNA, regulate gene transcription, and play roles in many cellular processes, including cell proliferation, differentiation, and cancer progression [74]. For example, ZNF208 may serve as a tumor suppressor, with an epigenetic mechanism of differentiation to adenocarcinoma from *ZNF208* gene silencing due to promoter hypermethylation and *ZNF208* underexpression [75]. Thus, it is possible that *LMNA* haploinsufficiency results in the epigenomic dysregulation of *ZNF* gene expression during the differentiation of CPs into EPDCs, a similar pathogenic mechanism proposed for defective myogenesis in patients with Emery–Dreifuss muscular dystrophy associated with the *LMNA* R453W missense mutation [11,76].

For the CM progenitor cells and CMs, our enrichment results provided evidence for differences between conditions in developmental pathways, including cardiogenesis and EMT. The CP-A cells were enriched for muscle cell differentiation, attributed to the decreased expression of *LMNA* and *NKX2-5*, which encode a key transcription factor in cardiogenesis and myocardial regeneration [77,78]. The predominant CM subtype at Day 16 was then enriched for muscle contraction and development, attributed to the underexpression of 20 different DEGs, including *NKX2-5* and WNT-signaling protein-encoding gene *WNT2*, as well as for muscle development and differentiation, attributed to the overexpression of 31 different DEGs, including the sarcomere protein-encoding gene *ACTC1.* At Day 16, the CMs were enriched for EMT, attributed to the upregulation of 23 DEGs, including the mesenchymal marker *CDH2* and the known EMT and XL gene *TIMP1* [58]. At Day 19, CMs continued to show enrichment for muscle differentiation, attributed to the underexpression of 15 different genes, including three DEGs encoding key components and regulators for sarcomere development (*LMNA* [79], *MYOZ2* [80], and *SYNPO2L* [81]) as well as for myogenesis and muscle contraction, attributed to the overexpression of 49 different DEGs, including the XL *DMD* gene and sarcomere protein-encoding genes: *MYBPC3*, *MYL2*, *MYL3*, and *TNNT2*.

These results for *LMNA* haploinsufficiency in our iPSC-derived model suggest cardiomyocyte dysfunction due to the disruption of gene expression in EPDCs and CMs, as reported in two other iPSC-derived models using bulk RNA-seq for *LMNA* K117fs [21] at Day 40 and *LMNA* R225X at Day 14 [22]. Our results are also similar to those from studies of Lamin A/C loss-of-function that reported gene expression changes, including the upregulation for EMT in Lmna −/− mouse heart tissue, using bulk RNA-seq [16]. Another recent study reported precocious CM development with the upregulation of *Nkx2-5* and sarcomere genes in *Lmna* −/− mouse ESC-derived CPs and CMs and in *LMNA* knockdown iPSC-derived CMs, using bulk RNA-seq and qPCR [24]. Our results for *LMNA* haploinsufficiency in our iPSC-derived model support cell type-specific disruption of epigenomic developmental pathways for cardiogenesis and EMT in *LMNA*-mutant CP differentiation into EPDCs and CMs.

Using enrichment analysis, we also found evidence for differences in cell signaling pathways involving the TGF Beta superfamily for CMs and mTORC1 for EPDCs at Day 16. The predominant CM cell type was enriched for RSTK and TGF Beta signaling pathways, attributed to the underexpression of multiple DEG, including *SMAD6*, which encodes a signaling inhibitor [82], and *ID1*, *ID2*, and *ID3*, which encode three Inhibitor-of-DNA-Binding proteins, which regulate signaling in heart development [83]. The CP-to-CM lineage was also enriched for the regulation of BMP signaling pathway, attributed to the overexpression of four DEGs encoding regulatory proteins involved in heart growth and development: *BMP4* [84], XL gene *GPC3* [85], *FST* [86], and *LRP2* [87], as well as the regulation of response to growth factor, attributed to the overexpression of seven DEGs, such as *GPC1*, encoding Glypican Proteoglycan 1, a modulator of signaling pathways that regulates cell growth, motility, and differentiation [88]. In contrast to the CP-to-CM lineage, the EPDCs at Day 16 were enriched for the mTORC1 signaling pathway, attributed to the upregulation of genes involved in the metabolism of glucose, lactate, and amino acid metabolism.

These results for *LMNA* haploinsufficiency in our iPSC-derived model suggest that both CM and EPDC dysfunction due to the dysregulation of TGF Beta and mTORC1 signaling, as reported in previous studies using *LMNA*-mutant mouse models and the Patient samples. These studies reported hyperactivation of TGF Beta signaling in *Lmna* −/− MEF [13] and in *Lmna* H222P/H222P mouse cardiomyocytes and heart tissue, with myocardial fibrosis and increased collagen expression [14]. Another study reported the hyperactivation of AKT-mTORC1 in heart tissue from *Lmna* H222P/H222P mice and from three patients with the *LMNA* mutation [15]. A recent study also provided evidence for the dysregulation of the TGF Beta/BMP pathway and *BMP4* overexpression using LAD analysis in *LMNA*-related DCM heart tissue [17,18]. In addition, the snRNA-seq study of *LMNA*-related DCM tissue showed cell type-specific dysregulation of multiple signaling pathways, including BMP signaling [38]. Similarly, our iPSC-derived model for *LMNA* haploinsufficiency supports altered cell-specific responses to signaling pathways involving TGF Beta/BMP in mutant CMs and mTORC1 in mutant EPDCs.

**Differential expression of X-linked genes, including *XIST*, six imprinted genes, and gene sets involved in the regulation of metabolism, homeostasis, and proliferation.** Among the top cell type and lineage DEGs, we found the non-coding *XIST* RNA gene that initiates XCI [52]. For XIST, we detected no expression, or lower expression levels of *XIST* in the Patient, compared to the Control cells, at all time points and shared cell types. Despite the limited *XIST* expression in the Patient cells, we detected primarily monoallelic expression for seven XL genes (*PRICKLE3*, *MAGED2*, *MAGEH1*, *KIF4A*, *ARMCX4*, *HTATSF1*, and *IDS*), known to be subject to XCI [55], consistent with the reported retention of clonal X-inactivation during fibroblast reprogramming and iPSC differentiation [89]. In contrast, for four of five XL genes (*CD99*, *EIF2S3*, *PLS3*, *TMEM187*) with consistent or variable XCI escape [55], we detected the biallelic expression of equal proportions across the Patient cells, compared to the skewed expression across the Control cells, during iPSC differentiation into CMs and EPDCs, suggesting possible partial X chromosome reactivation or XCI erosion. Since the nuclear lamina plays an important role in *XIST*-mediated X chromosome gene silencing [90,91], we might speculate that our findings for altered *XIST* and XL gene expression may be evidence for the disruption of epigenomic developmental pathways due to Lamin A/C haploinsufficiency.

In addition to *XIST*, among the top cell type and lineage DEGs, we found six imprinted genes: *SNRPN*, *PWAR6*, *NDN*, *PEG10*, *MEG3*, and *MEG8*, located at known imprinted regions. In the Patient, compared to the Control cells, we found decreased expression for four paternally expressed genes: *SNRPN*, *PWAR6*, *NDN*, at chr15q11.2, and *PEG10*, at 7q21 and increased expression for two maternally expressed genes: *MEG3* and *MEG8*, at chr14q32.2. Since these imprinted genes lacked informative coding SNV, we could not determine biallelic vs. monoallelic expression. In a genomic imprinting model using mouse ESCs, the biallelic expression of maternally expressed *Meg3* occurred further away from the nuclear periphery [92]. Similarly, we might speculate that our findings in human cells with Lamin A/C haploinsufficiency is due to the disruption of epigenomic developmental pathways, altered genome organization, and subsequent altered or loss of imprinting. Further studies on a possible role of Lamin A/C in the maintenance of XCI and genomic imprinting are needed.

Using top DEGs, we also found metabolic gene signatures consistent with both cell type and metabolic selection, as previously reported [59,93], but also differences in cell metabolism between the Patient and Control cells by cell type and lineage. First, the predominant PP subtype at Day 0 was enriched for OXPHOS, with the ETC genes *DNAJC15*, *NDUFA3*, and *NDUFA6* underexpressed in the Patient cells. For the CP subtypes at Day 9, the CM progenitors were enriched for oxidative metabolism with 46 DEG downregulated in the Patient cells, while the EPDC progenitors were enriched for glycolytic metabolism with the phosphofructokinase-encoding gene *PFKP*, which was overexpressed in the Patient cells. For both the Control and Patient CMs and EPDCs, we also found unique metabolic gene signatures, consistent metabolic selection for CMs using lactate oxidation, as previously reported [59,93], but also EPDC survival, using alternative metabolic pathways. For CMs and EPDCs at Day 16 (post-metabolic selection), CMs were enriched for glucose metabolism and OXPHOS, with an overexpression of 98 DEGs, while EPDCs were enriched for glucose and nucleotide metabolism, with an overexpression of 18 DEGs. Only three DEGs (*ATP5ME*, *COX4I1*, *P4HA2*) were overexpressed in both CMs and EPDCs.

These results in our iPSC-derived model demonstrated the ability to detect gene signatures associated with specific cell type, metabolic selection, and condition (Patient vs. Control). Two previous studies reported gene expression changes using bulk RNA-seq with downregulation of OXPHOS in Lmna −/− mouse heart tissue [16] and mitochondrial dysfunction in *LMNA* E342K iPSC-CM [94]. Using snRNA-seq, heart tissues from *LMNA* patients showed CM-specific alterations of mitochondrial metabolism and glycolysis through the upregulation of *FNIP2* and *CPEB4* [38]. Using scRNA-seq to distinguish between CP, CM, and EPDC, our results in our iPSC-derived model with Lamin A/C haploinsufficiency might also suggest an association with metabolic activity dependent on mutant cell type and lineage. For example, based on our findings, metabolic activity might decrease in CM progenitors and increase in EPDC progenitors with Lamin A/C haploinsufficiency.

In addition, our results provided evidence for differences in cell homeostasis and response between Patient and Control cells for two lineages. The PP-A to PP-B and CP to CM lineages were enriched for metal homeostasis, attributed to the overexpression of *BNIP3*, which encodes a pro-apoptotic mitochondrial protein, and metallothionein protein genes (*MT*): *MT1E*, *MT1F*, *MT1G*, *MT1H*, *MT1X*, and *MT3*, which encode various heavy metal binding proteins [54]. The BNIP3 and MT proteins play important roles in the regulation of normal cell processes, including cell growth, differentiation, mitochondrial function, and responses to oxidative stress [95,96]. A study on *Bnip3* overexpression in mouse ESCs and iPSCs reported decreased ROS levels and DNA damage and increased mitochondrial respiration and DNA repair [97]. Another study reported *LMNA* S143P iPSC-CM had increased sarcomere disorganization with increased cellular stress due to hypoxia [98]. Similarly, our enrichment results in our iPSC-derived model for *LMNA* haploinsufficiency may suggest the dysregulation of cellular homeostasis and responses to oxidative stress or hypoxia in *LMNA*-mutant PPs and CPs to CM lineages.

Finally, our results provided evidence for differences in cell proliferation for CMs at Day 16 and the CP to EPDC lineage. The predominant CM subtype was enriched for Hallmark Myc targets V1, with upregulation of 36 DEGs, including ten spliceosome genes, *TYMS*, a canonical S-phase marker [99], and other proto-oncogene targets for the activation of growth-related genes [54,60]. The CP to EPDC lineage was enriched for cyclin-dependent kinase activity, attributed to the overexpression, primarily in CP cells, of two CC genes, *CDKN3*, encoding a cyclin-dependent kinase inhibitor, and *CCNB2*, encoding Cyclin B2, required for control at the G2/M transition [54]. Our results, supporting increased proliferation of CM and EPDC progenitors, agree with the reported increased proliferation and altered CC progression in *Lmna* −/− MEF [13] and increased *CCNB2* expression in cancer cell lines with *LMNA* knockdown [100]. However, our results disagree with the reported restricted proliferation and delayed CC activity of *Lmna* −/− mouse CMs [101] and the downregulation of CC genes in *Lmna* −/− mouse ESC-derived CPs and CMs and in *LMNA* knockdown iPSC-CMs [24]. These discrepancies may involve *LMNA* genotype (haploinsufficiency vs. loss of function), species (human vs. mouse), growth conditions, including metabolic selection, and possible biological and technical variables in our iPSC-derived model. Further evaluations using single-cell analyses are required to define cell type-specific changes in cell proliferation and CC control.

## 5. Conclusions and Challenges

In our on-going studies, focused on a multi-generation family with a heterozygous *LMNA* c.357-2A>G splice-site mutation to identify the possible molecular mechanism of *LMNA*-related DCMs [37,40,41], we generated and differentiated iPSC lines, derived from an affected female (Patient) and her unaffected sister (Control) and conducted scRNA-seq across multiple time points. To evaluate cell heterogeneity, we used a comprehensive scRNA-seq bioinformatic workflow with serial, cell type-specific data processing. After extensive multi-level analyses of 110,521 high-quality cells, we found substantial complexity in our iPSC-derived model: ten main cell types, many possible subtypes, and several lineages, including the bifurcation of CPs into CMs and EPDCs lineages, consistent with previous scRNA-seq studies of normal iPSC differentiation. To identify cell type and lineage-specific mechanisms, we used comparative analyses of Patient and Control cells. In our analyses of the top cell type and lineage DEGs, we found evidence for the ‘gene expression’ hypothesis, as proposed in other *LMNA* disease models. Overall, using our Patient-derived iPSC model and single-cell transcriptomics, our study supports an association with Lamin A/C haploinsufficiency and cell type and lineage-specific disruption of epigenomic developmental programs such as gene transcription, cell signaling, and cardiogenesis and EMT differentiation pathways.

Although our evidence supports the ‘gene expression’ hypothesis and pathway dysregulation, as proposed in other *LMNA* disease models, we acknowledge the limit of our sample size and the challenges of data interpretation in an iPSC-derived model due to variability from biological and technical factors, which can influence gene expression, epigenetic regulation, and cell differentiation. These confounding factors include reported variability between different iPSC lines and clones [102,103] and variability from the susceptibility for epigenomic aberrations of human iPSC [104,105,106].

Thus, we advise caution for our interpretation of the results and emphasize the need for model improvement and validation in future studies. For our Patient-derived iPSC disease model, we will first need to resolve or reduce the confounding factors. To address the variability between iPSC lines and clones, we will need to conduct a larger scRNA-seq study with both biological replicates and isogenic Control iPSC lines. Biological replicates, multiple *LMNA* iPSCs derived from different family members, are needed to determine the levels of variability in CM differentiation and for gene expression between different *LMNA*-mutant iPSC lines and clones [102]. In addition, to minimize variability due to differences in the genomic background [103], we will need to create a matched set of isogenic Control iPSC lines by repairing the *LMNA* mutation in each Patient iPSC line using genome editing tools such as CRISPR-Cas9 or Base Editing [107,108].

To improve our Patient-derived iPSC disease model, we will also need to address the susceptibility of normal iPSC lines for epigenomic aberrations, which may influence multiple biological processes. Reported epigenomic aberrations include altered *XIST* expression, XCI erosion, and upregulation of XL genes [106], altered DNA methylation, and loss of imprinting for many genes, such as *SNRPN*, *NDN*, *MEG3*, *PEG10* [104], associated with somatic reprogramming, molecular memory of cell origin, and cell culturing [106]. A recent study showed the effects of molecular memory in human iPSCs of fibroblast origin, with retained expression for fibroblast-specific genes and increased expression for mesoderm progenitor markers such as *BMP4*, compared to human ESCs [109]. To address the biological effects of molecular memory, we may try to wipe away the fibroblast epigenomic memory in our *LMNA* Patient and Control fibroblast or iPSC lines to create cells in the naïve pluripotency state (naïve iPSC), which more closely resemble human ESCs [110], using a new method called transient-naïve-treatment [109].

By improving our Patient-derived iPSC model using biological replicates, isogenic Controls, and naïve iPSCs, it might be possible to separate the consequences of *LMNA* haploinsufficiency from confounding factors and to continue testing the ‘gene expression’ hypothesis for *LMNA*-related DCM. Finally, with the newly described role of Lamin A/C in naïve pluripotency and cardiovascular cell fate [12,24], successfully addressing the limitations may enable new investigations to evaluate other possible mechanisms, including epigenomic dysregulation, using naïve iPSC-based models for *LMNA*-related DCM.

## Data Availability

The raw and processed data for single-cell RNA-sequencing are available as NCBI BioProject PRJNA1120081.

## Supplementary Materials

The following supporting information can be downloaded at: https://www.mdpi.com/article/10.3390/cells13171479/s1, Text: Methods and Results; Figure S1: iPSC Validation Results: Differentiation Capability by ICC Staining; Figure S2: Allelic Expression Using Coding Single Nucleotide Variants (SNVs); Figure S3: Workflow Step-I: Data Processing Results; Figure S4: Workflow Step-II: Clustering and Annotation; Figure S5: Workflow Step-II: Single Sample Data Results- Individual Analyses of Singlet Data for Main Cell Types; Figure S6: Workflow Step-II: Single Sample Data Results- Subcluster Analyses of Subset Data for Possible Cell Subtypes; Figure S7: Workflow Step-III: Data Combining & Comparative Analyses; Figure S8: Workflow Step-III: Paired Sample Data Results- Individual Analyses of Combined Singlet Data; Figure S9: Workflow Step-III: Paired Sample Data Results- Individual Subcluster Analyses of Combined Subset Data for Possible Shared Subtypes; Figure S10: Workflow Step-III: Paired Sample Data Results- Comparative Analyses for Cell Type-Specific DE; Figure S11: Workflow Step-III: Single Subset Data Results- Trajectory Analyses for Lineage-Specific DE and Enrichment; Figure S12: Workflow Step-III: Paired Subset Data Results- Trajectory Analyses for Lineage-Specific DE and Enrichment; Figure S13: Western blots (n = 3); Table S1: Human subjects and results for fibroblast *LMNA* sequencing and iPSC validation; Table S2: Methods: Primary and secondary antibodies; Table S3: Methods: Main software packages; Table S4: scRNA-seq Summary Metrics using Cell Ranger; Table S5: Single Sample Data: QC data processing using SoupX, Seurat, and DoubletFinder; Table S6: Single Sample and Combined Data: QC processing of Raw to Singlet Data Matrices; Table S7: Single Sample Data: Individual and Subcluster Analyses for Cell Annotation; Table S8: Gene marker panels for cell annotation; Table S9: Paired Sample Data: Individual and Subcluster Analyses; Table S10: Single Subset Data: Trajectory Inference, Lineage DEG, and Enrichment; Table S11: Paired Subset Data: Integration, Trajectory Inference, Lineage DEG, and Enrichment; Table S12: Lamin A/C Western Blot Quantification Data and Statistical Analyses; Excel Table S1: Cluster. Subcluster. DEG; Excel Table S2: Cell Type. DEG; Excel Table S3: Lineage. DEG; Excel Table S4: Allelic Expression.
