# Supplementary material for: LMNA-Related Dilated Cardiomyopathy: Single-Cell Transcriptomics during Patient-Derived iPSC Differentiation Support Cell Type and Lineage-Specific Dysregulation of Gene Expression and Development for Cardiomyocytes and Epicardium-Derived Cells with Lamin A/C Haploinsufficiency
Source: Cells. 2024 Sep 3;13(17):1479. doi: 10.3390/cells13171479 (PMC11394257; doi:10.3390/cells13171479)

LMNA-Related Dilated Cardiomyopathy: Single-Cell Transcriptomics during Patient-derived iPSC Differentiation Support Cell type and Lineage-specific Dysregulation of Gene Expression and Development for Cardiomyocytes and Epicardium-Derived Cells with Lamin A/C Haploinsufficiency. M. Zaragoza, T-A. Bui, H. Widyastuti, M. Mehrabi, Z. Cang, Y. Sha, A. Grosberg, Q. Nie; Univ. of California, Irvine, Irvine, CA

**Supplemental Figures**		**Page**
**Figure S1.**	**iPSC Validation Results: Differentiation Capability by ICC Staining**	**3**
**Figure S2.**	**Allelic Expression Using Coding Single Nucleotide Variants (SNVs)**	**4 - 8**
	**A. Autosomal Genes including _LMNA_**	
	**B. X-Linked Genes with X-Chromosome Inactivation (XCI) Status and Results Summary**	
**Figure S3.**	**Workflow Step-I: Data Processing Results**	**9 - 16**
	**A. Summary: Sequential QC Processing of Raw-Corrected-Filtered-Singlet Data**	
	**B. Merged Data for All Single Sample Samples: QC Covariate Plots**	
	**C. Merged Data for All Single Sample Samples: QC Covariate Plots- Control vs. Patient**	
	**D. Merged Data for All Single Sample Samples: QC Covariate Plots by Cluster**	
	**E. Background RNA: Removal Using SoupX**	
	**F. Cell Quality: Removal of Low-Quality Cells Using Seurat**	
	**G. Doublets: Identification and Removal Using DoubletFinder (DF)**	
**Figure S4.**	**Workflow Step-II: Clustering and Annotation**	**17 - 18**
	**A. Summary: Single Sample Data for Cell Annotation**	
	**B. Summary: Merged Single Sample Data for Cell Annotation (110,521 Total Cells)**	
**Figure S5.**	**Workflow Step-II: Single Sample Data Results- Individual Analyses of Singlet Data for Main Cell Types**	**19 - 31**
	**A. Summary: Raw Data to Annotated Clusters**	
	**B. Single Sample Data Analyses: Control Samples (n=8)**	
	**C. Single Sample Data Analyses: Patient Samples (n=4)**	
**Figure S6.**	**Workflow Step-II: Single Sample Data Results- Subcluster Analyses of Subset Data for Possible Cell Subtypes**	**32 - 44**
	**A. Summary: Annotated Clusters to Annotated Subsets**	
	**B. Subcluster Analyses: Control Samples (n=8) to Annotated Subset Data (n=21)**	
	**C. Subcluster Analyses: Patient Samples (n=4) to Annotated Subset Data (n=11)**	

Figure S7. **Workflow Step-III: Data Combining & Comparative Analyses** **45 - 46**
A. Summary: Combined Data for Paired Sample Data
B. Summary: Combined Data for 'Balanced' Paired Subsets (n=6 Prs: 75,330 Total Cells)

Figure S8. **Workflow Step-III: Paired Sample Data Results- Individual Analyses of Combined Singlet Data for Shared Cell Types** **47 - 52**
A. Summary: Merged vs. Integrated Singlet Data- Clusters and Imbalance
B. Summary: Cell Annotation of Integrated Singlet Data (n=4 Prs: 89,269 Total Cells)
C. Paired Sample Data Analyses: Integrated Singlet Data- Patient vs. Control (n= 4 Prs)

Figure S9. **Workflow Step-III: Paired Sample Data Results- Individual Subcluster Analyses of Combined Subset Data for Possible Shared Subtypes** **53 - 68**
A. Summary: Merged vs. Integrated Subset Data- Clusters and Imbalance
B. Summary: Cell Annotation of Integrated Subset Data (n=11 Prs: 88,420 Total Cells)
C. Paired Sample Data Analyses: Integrated Subset Data- Patient vs. Control (n= 11 Prs)

Figure S10. **Workflow Step-III: Paired Sample Data Results- Comparative Analyses for Cell Type-Specific DE** **69 - 93**
A. Summary: Cell Type Differentially Expressed Genes (Cell Type DEG)
B. Summary: Volcano Plots and Cell Type DEG (n=14 Cell Subtypes: 71,541 Total Cells)
C. Individual Analyses of Integrated Subsets: Cell Type DEG and Enrichment (n=14)
D. Cell Type DEG: *LMNA*, X-Linked Genes, and Imprinted Genes Across 14 Subtypes
E. Cell Type DEG Enrichment: Module Scoring of GSEA Significant Gene Sets

Figure S11. **Workflow Step-III: Single Subset Data Results- Trajectory Analyses for Lineage-Specific DE and Enrichment** **94 - 108**
A. Summary: Annotated Subset Data to Cell Lineages
B. Single Subset Data: Trajectory, Lineage DEG, and Enrichment Analyses (n=7)

Figure S12. **Workflow Step-III: Paired Subset Data Results- Trajectory Analyses for Lineage-Specific DE and Enrichment** **109 - 120**
A. Summary: Lineage Differentially Expressed Genes (Lineage DEG)
B. Summary: UMAP Plots and Cell Lineages (n=2: 62,488 Total Cells)
C. Paired Subset Data Analyses: Pluripotent Cell Lineage (19,346 Total Cells)
D. Paired Subset Data Analyses: Cardiac Progenitor Lineages (43,142 Total Cells)

Figure S13. **Western blots (n=3)** **121 - 123**

Fig. S1 EB ICC

iPSC Validation Results: Differentiation Capability by Embryoid Body (EB) Immunocytochemistry (ICC) Staining

Control iPSCs

Control A1 Clone A (CA1-A)

Control A1 Clone B (CA1-B)

Control A2 (CA2)

Control A3 (CA3)

Unrelated Control (U2)

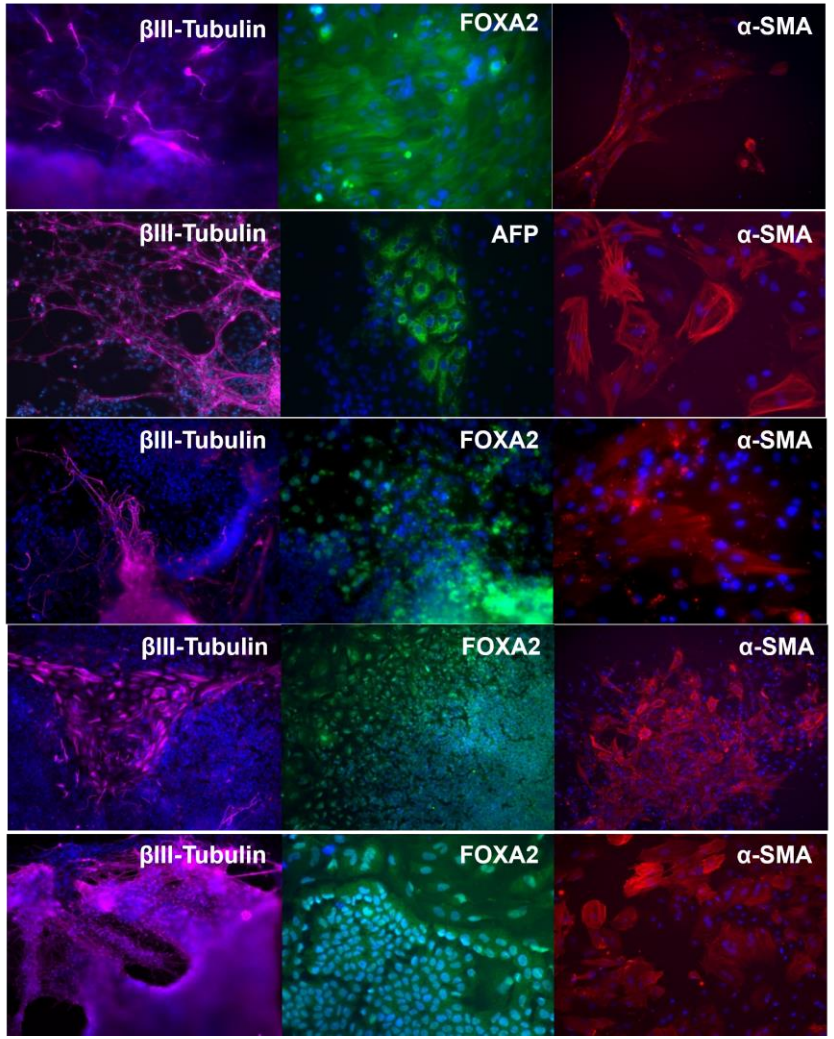

Patient iPSCs

Patient A1 (PA1)

Patient A2 (PA2)

Patient A3 (PA3)

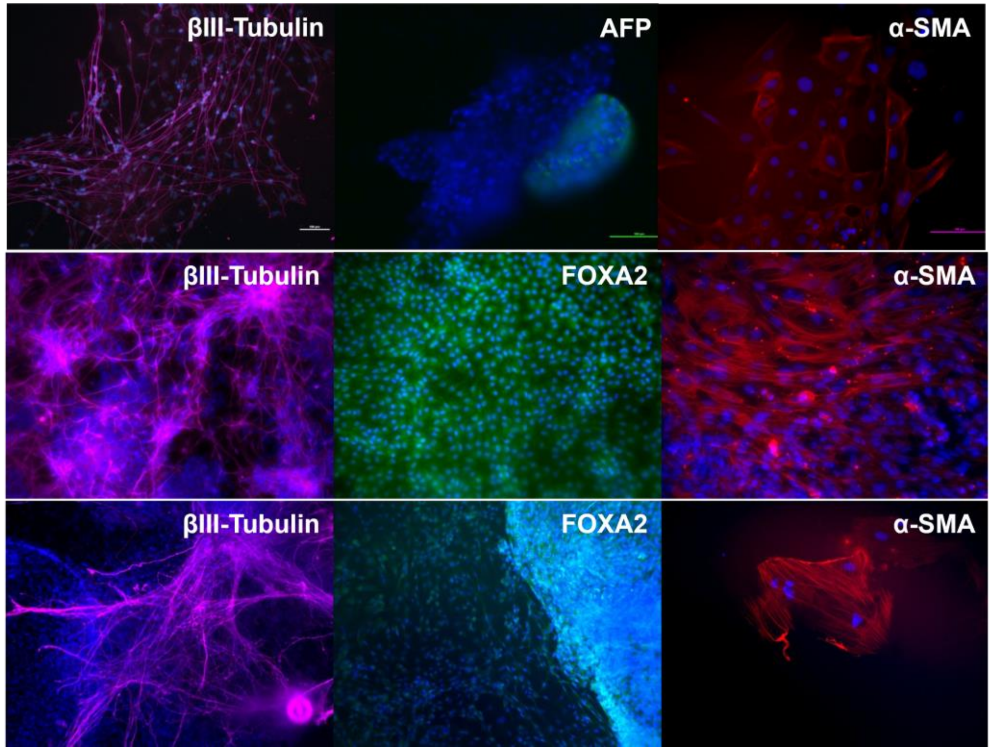

Germ Layer Markers

Endoderm: Forkhead Box A2 (FOXA2)
Alpha-fetoprotein (AFP)

Ectoderm: Beta-III Tubulin

Mesoderm: Smooth Muscle Actin (α-SMA)

Fig. S2 Coding SNV

Allelic Expression Using Coding Single Nucleotide Variants (SNVs)

A. Autosomal Genes including *LMNA*

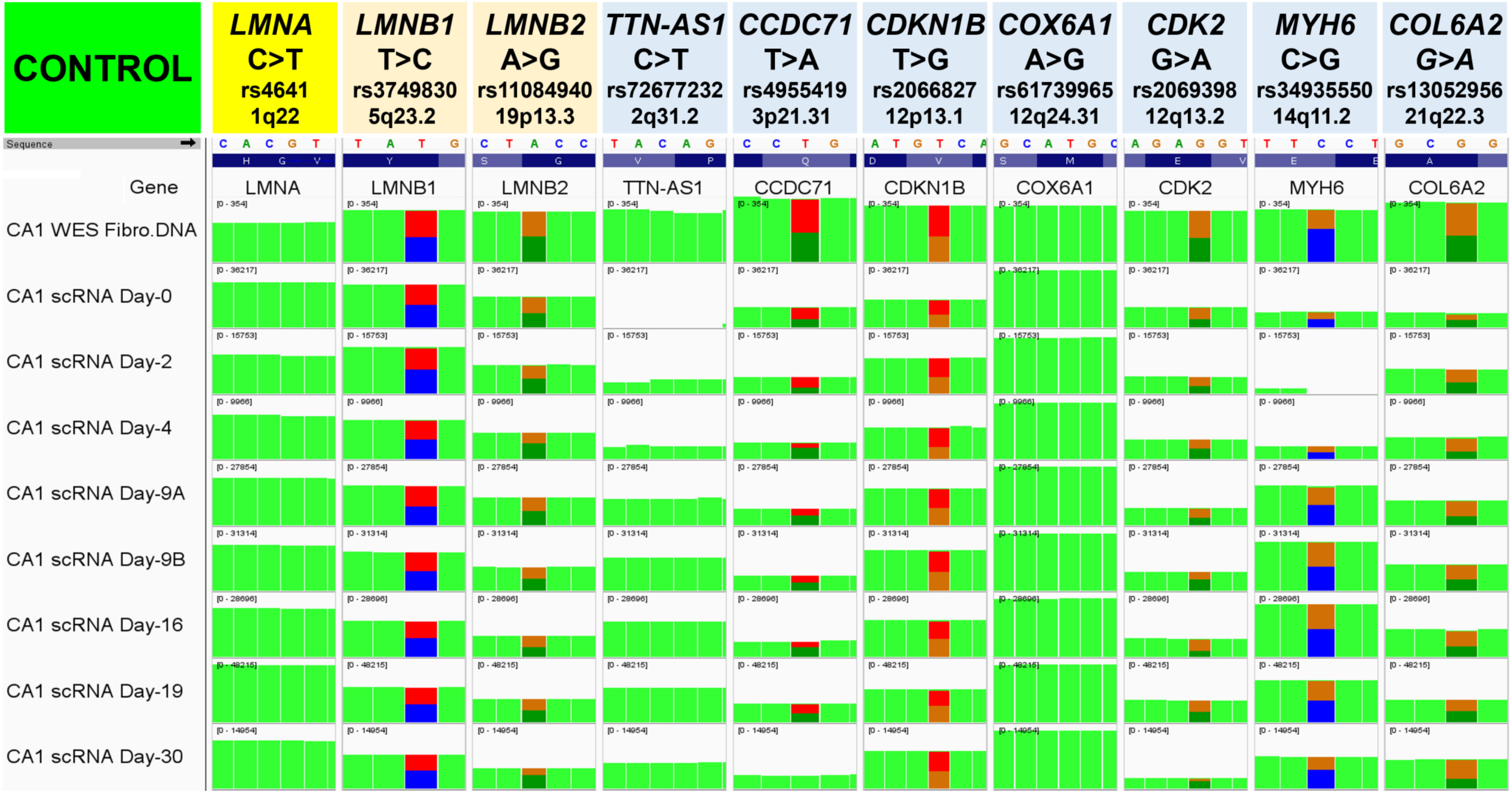

Integrative genomics viewer images of aligned reads to the human reference genome

Fig. S2 Coding SNV

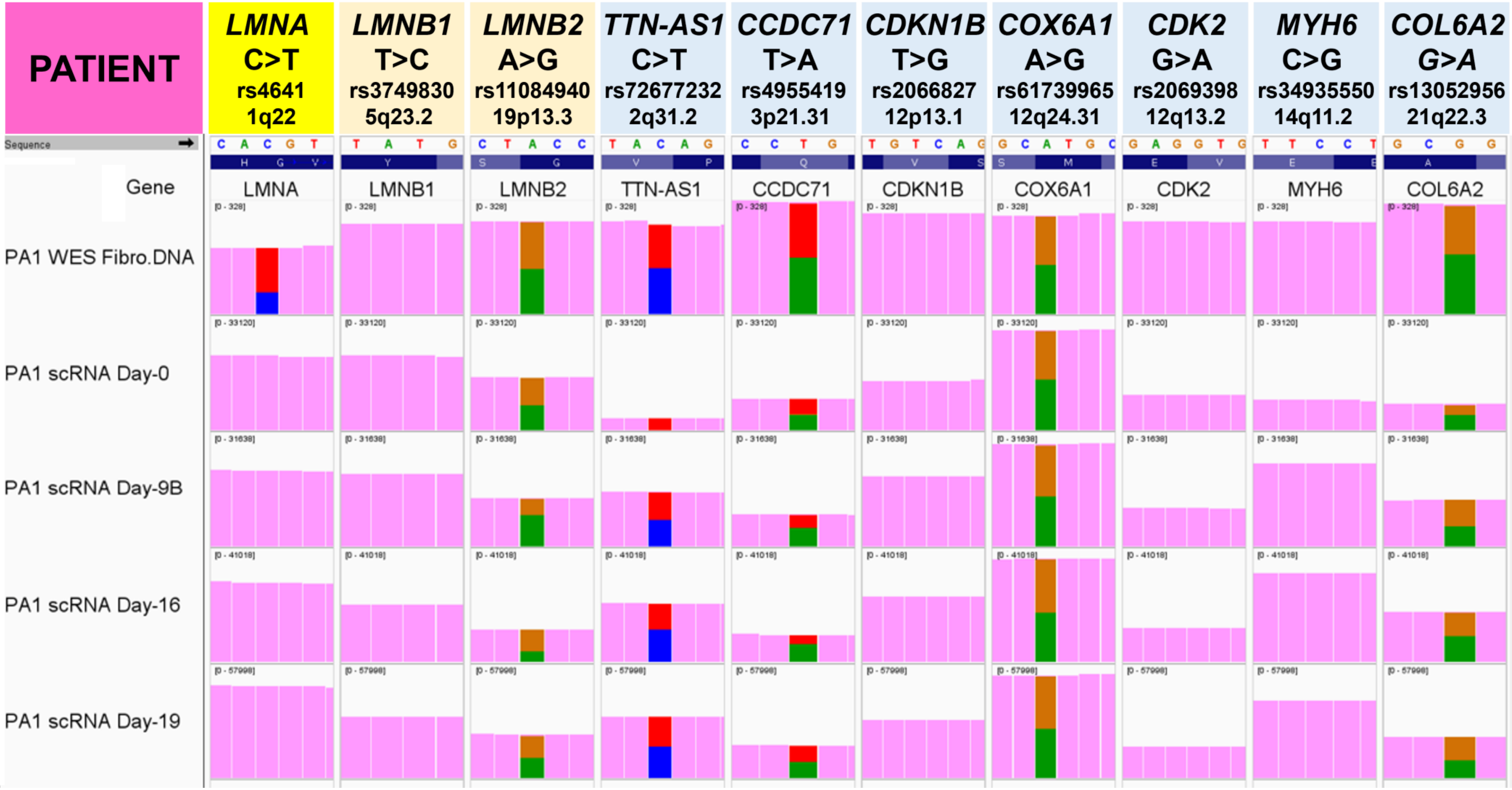

Fig. S2 Coding SNV

B. X-Linked Genes with X-Chromosome Inactivation (XCI) Status*

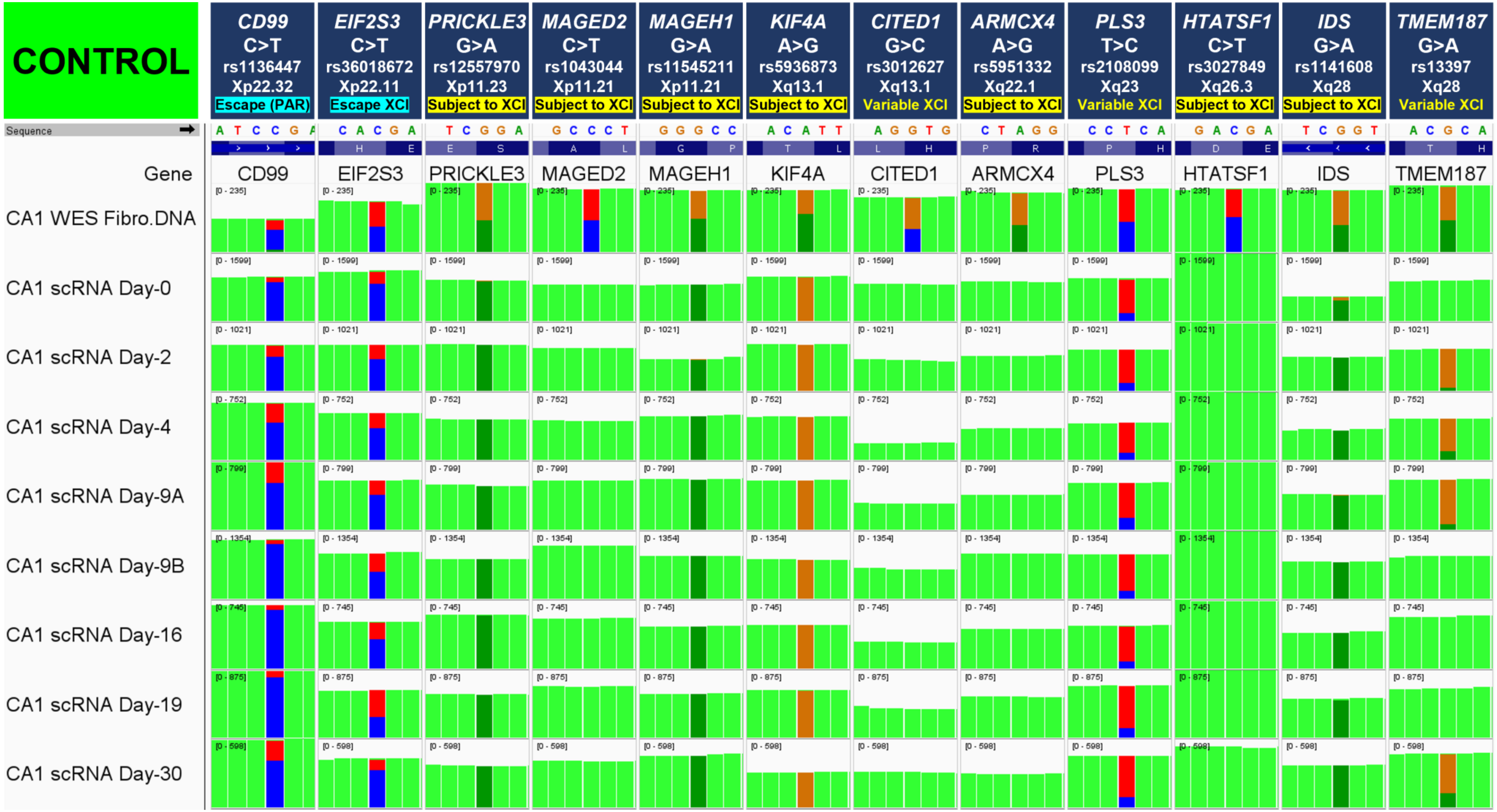

Fig. S2 Coding SNV

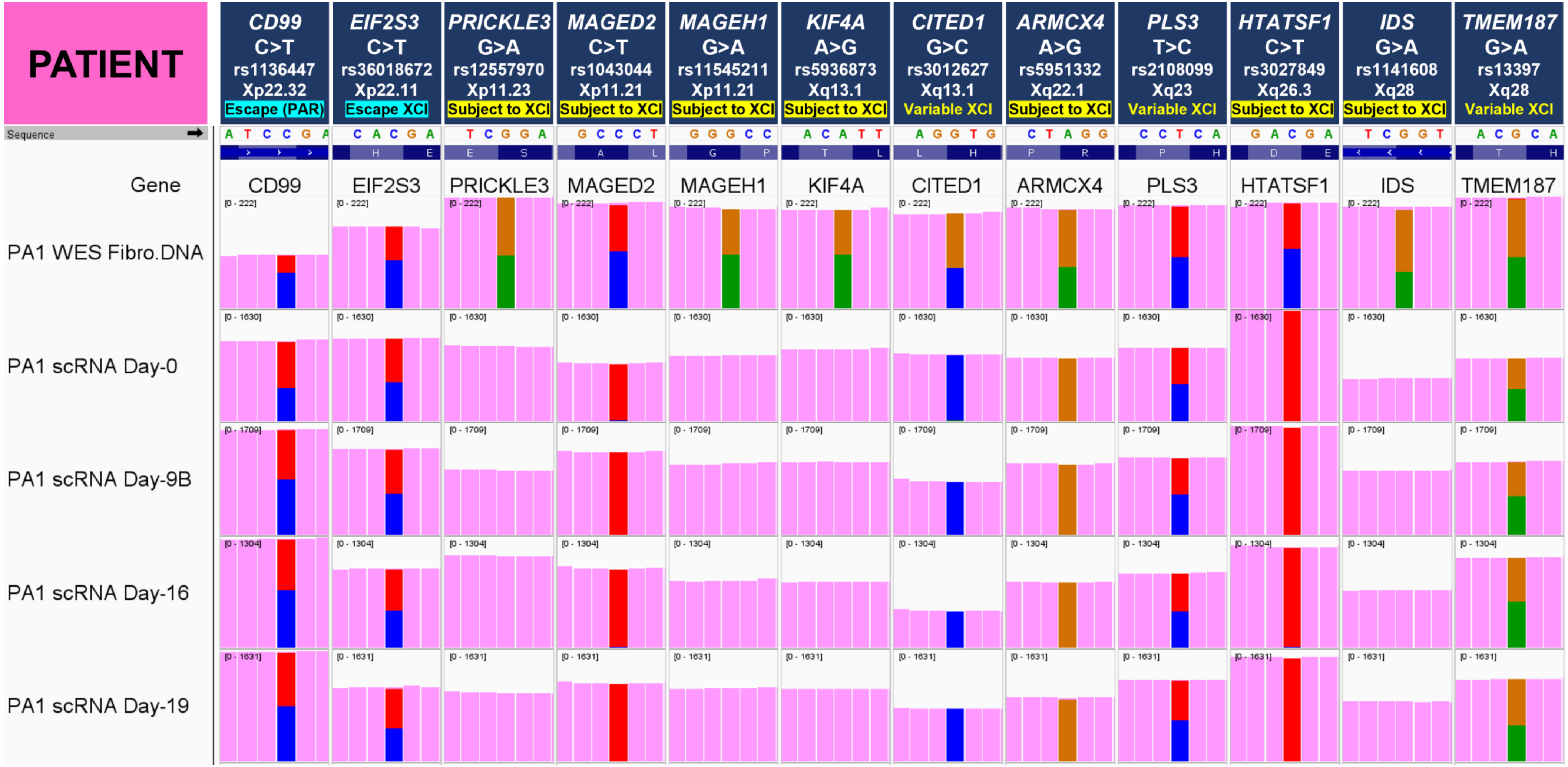

*XCI Status from Balaton *et al.* 2015

Fig. S2 Coding SNV

Allelic Expression Using Coding Single Nucleotide Variants (SNVs)

RESULTS SUMMARY: X-Linked Genes with X-Chromosome Inactivation (XCI) Status

Total Read Count by Allele for 12 scRNA-seq Samples

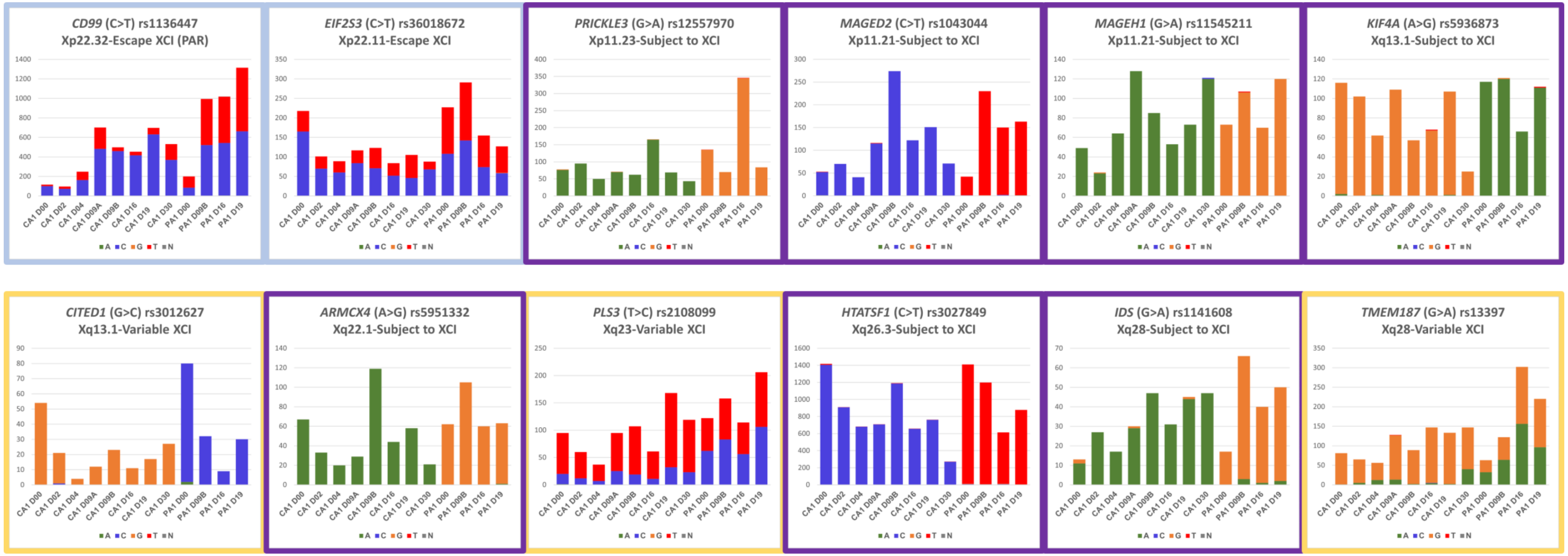

XCI Status of Gene (Balaton *et al.* 2015): Escape XCI Subject to XCI Variable XCI

Fig. S3 Processing

Workflow Step-I: Data Processing Results

A. Sequential QC Processing Summary: Raw-Corrected-Filtered-Singlet Data

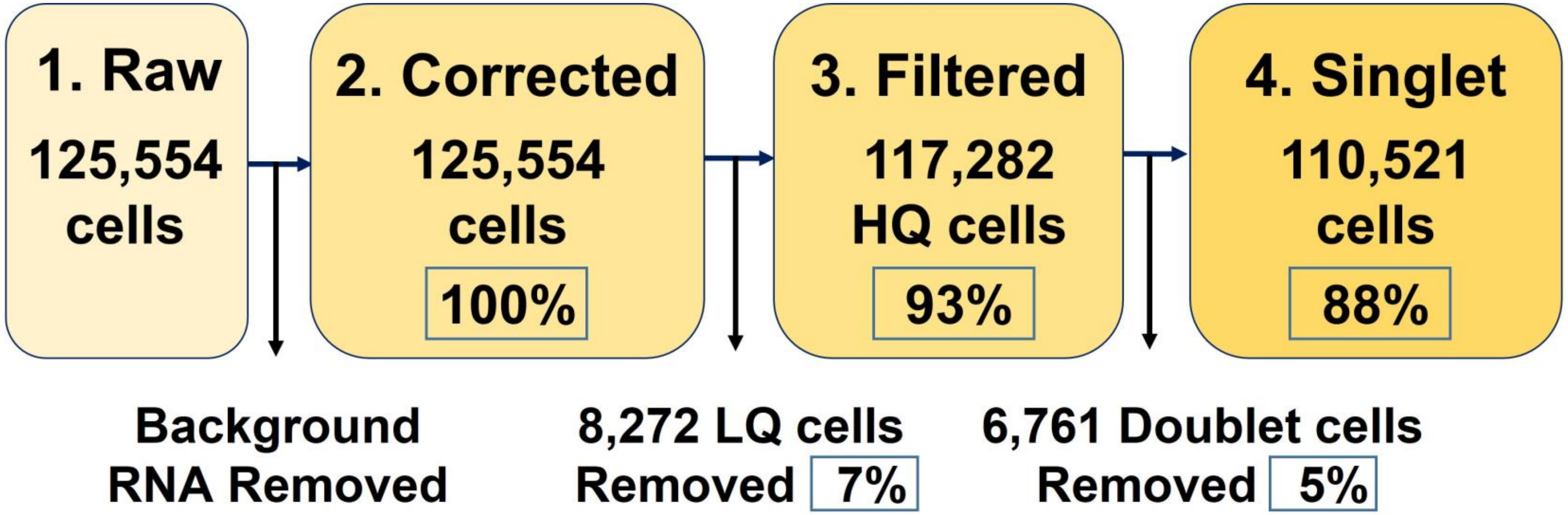

	No. Samples	No. Cells (% Raw Total)			
		1. Raw	2. Corrected	3. Filtered	4. Singlet
Control	8	74,097	74,097 (100%)	69,084 (93%)	65,324 (88%)
Patient	4	51,457	51,457 (100%)	48,198 (94%)	45,197 (88%)
All	12	125,554	125,554 (100%)	117,282 (93%)	110,521 (88%)

Fig. S3 Processing

B. Merged Data for All Single Sample Samples: QC Covariate Plots

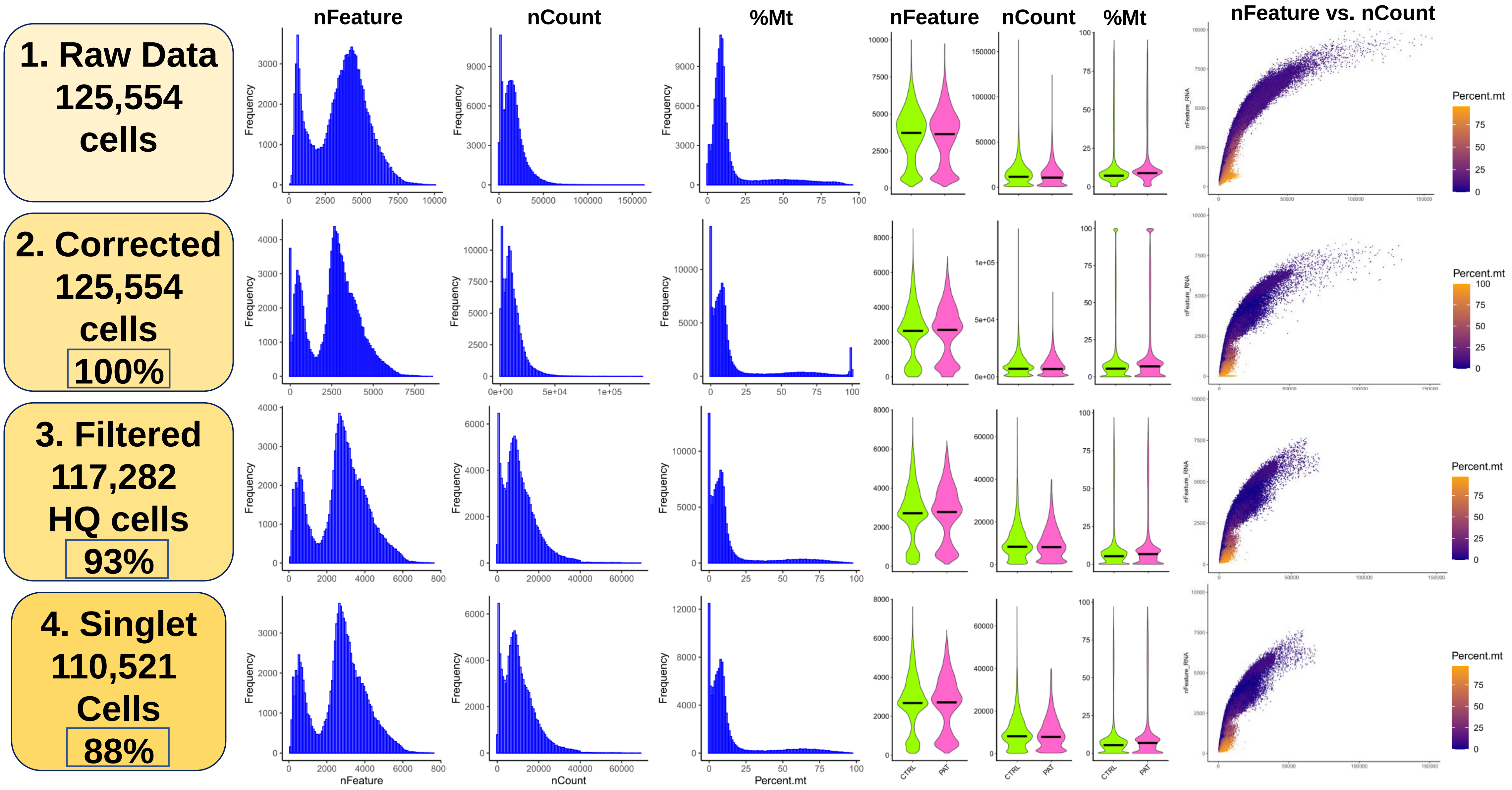

Fig. S3 Processing

C. Merged Data for All Single Sample Samples: QC Covariate Plots- Control vs. Patient

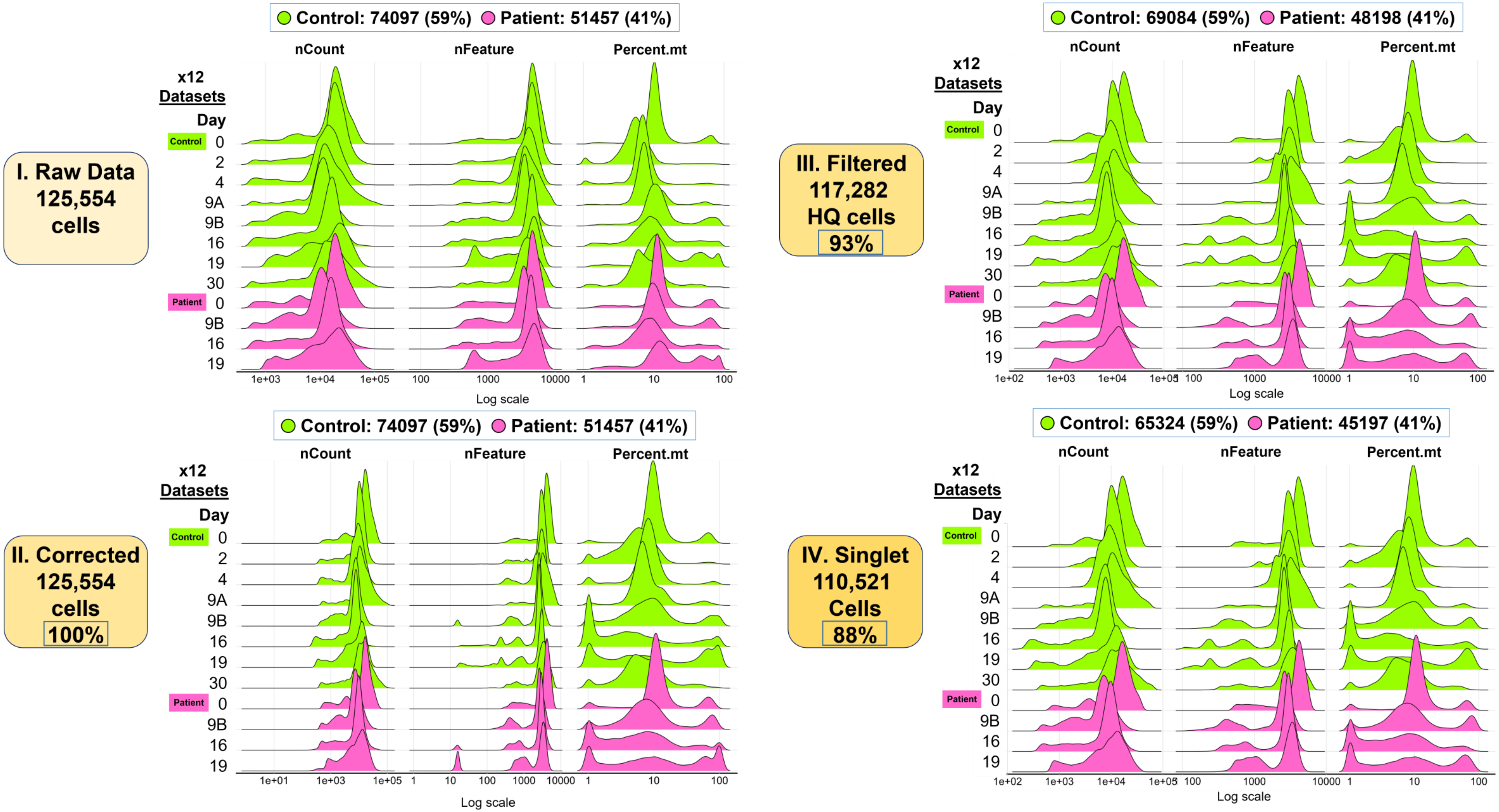

Fig. S3 Processing

D. Merged Data for All Single Sample Samples: QC Covariate Plots by Cluster

I. Raw Data 125,554 cells

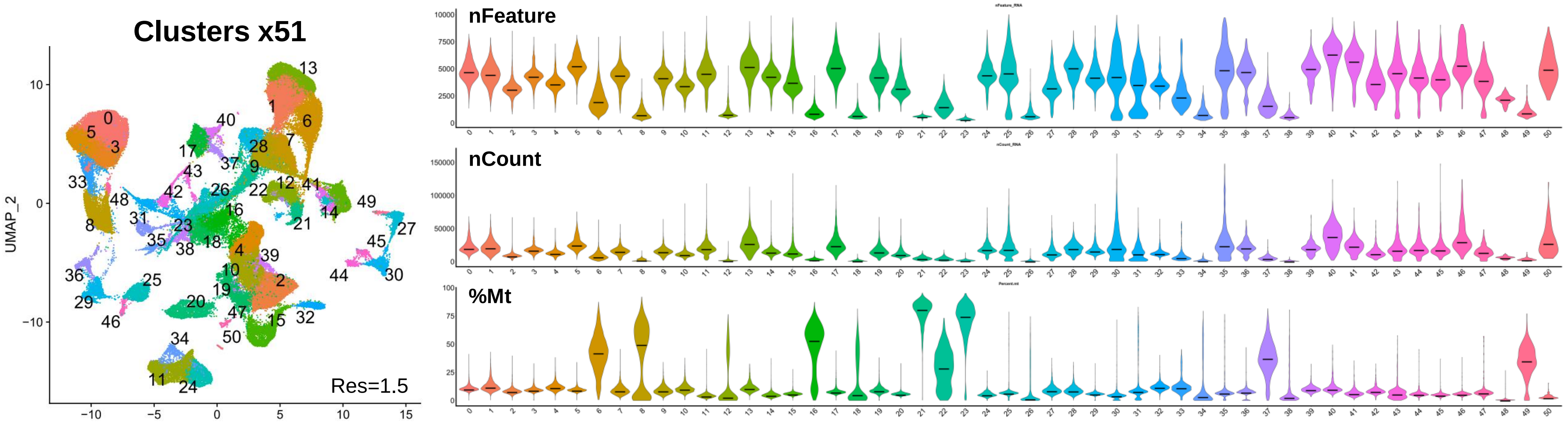

II. Corrected 125,554 cells 100%

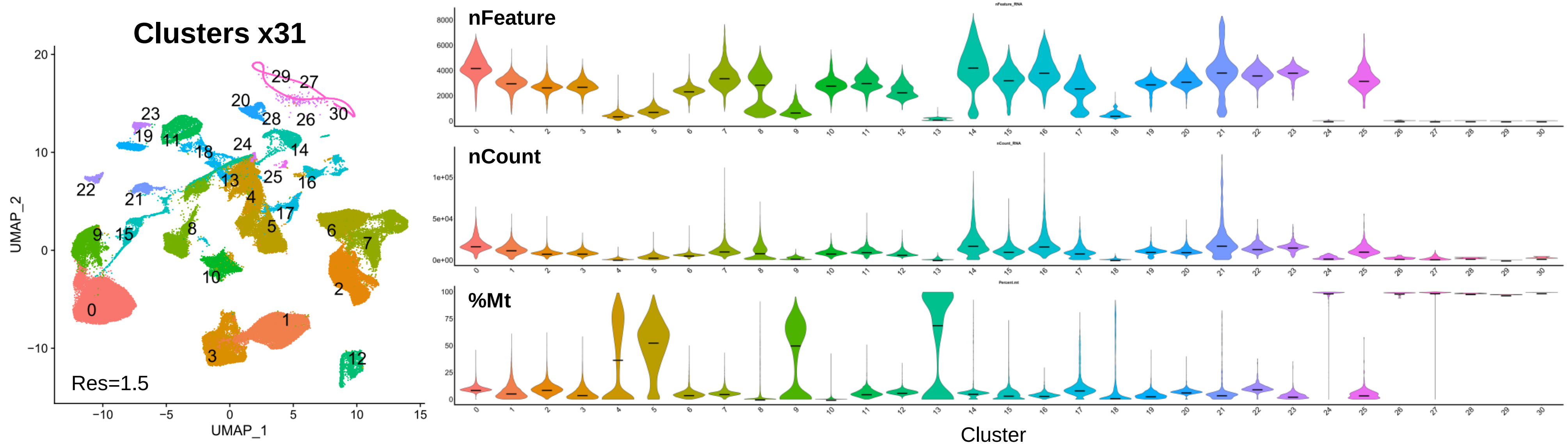

nfeatures = 3000, dims = 1:50

Fig. S3 Processing

III. Filtered
117,282
HQ cells
93%

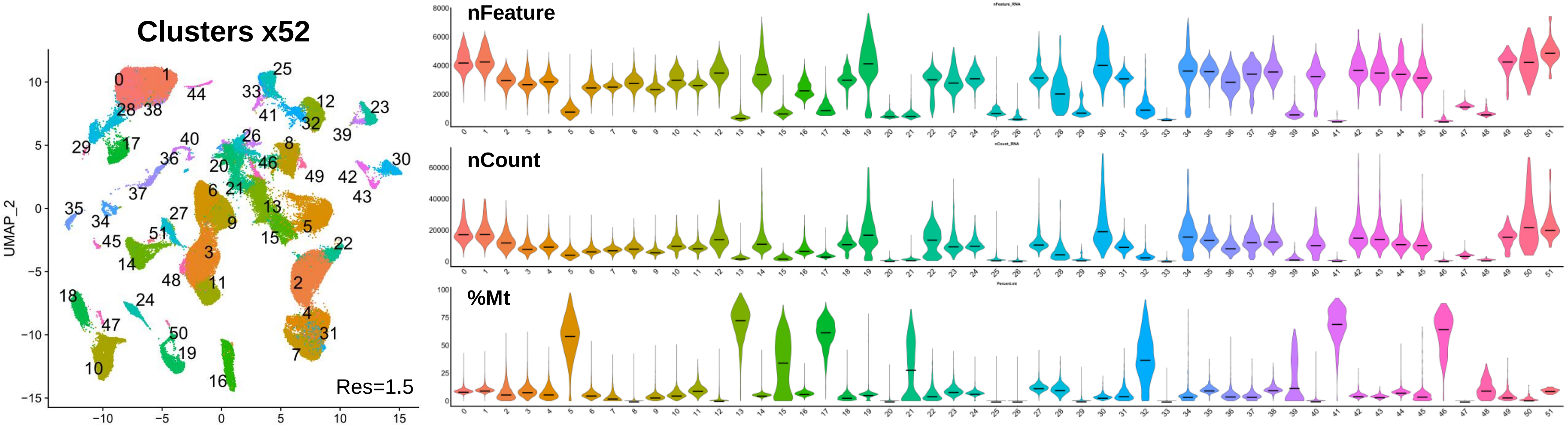

IV. Singlet
110,521
Cells
88%

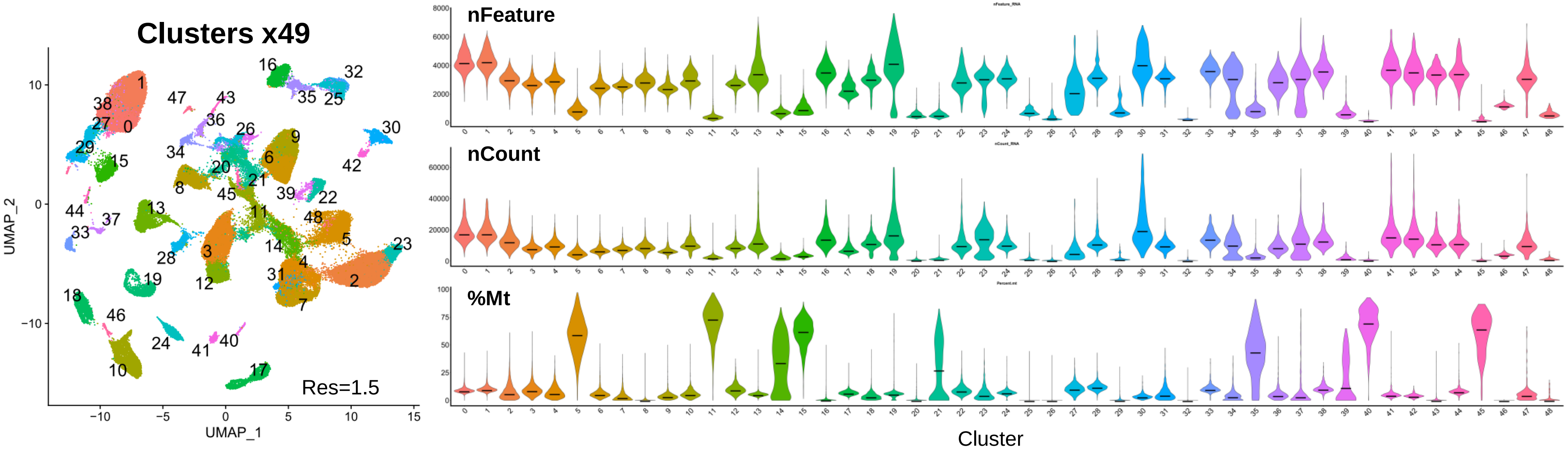

nfeatures = 3000, dims = 1:50

Fig. S3 Processing

E. Background RNA: Removal Using SoupX

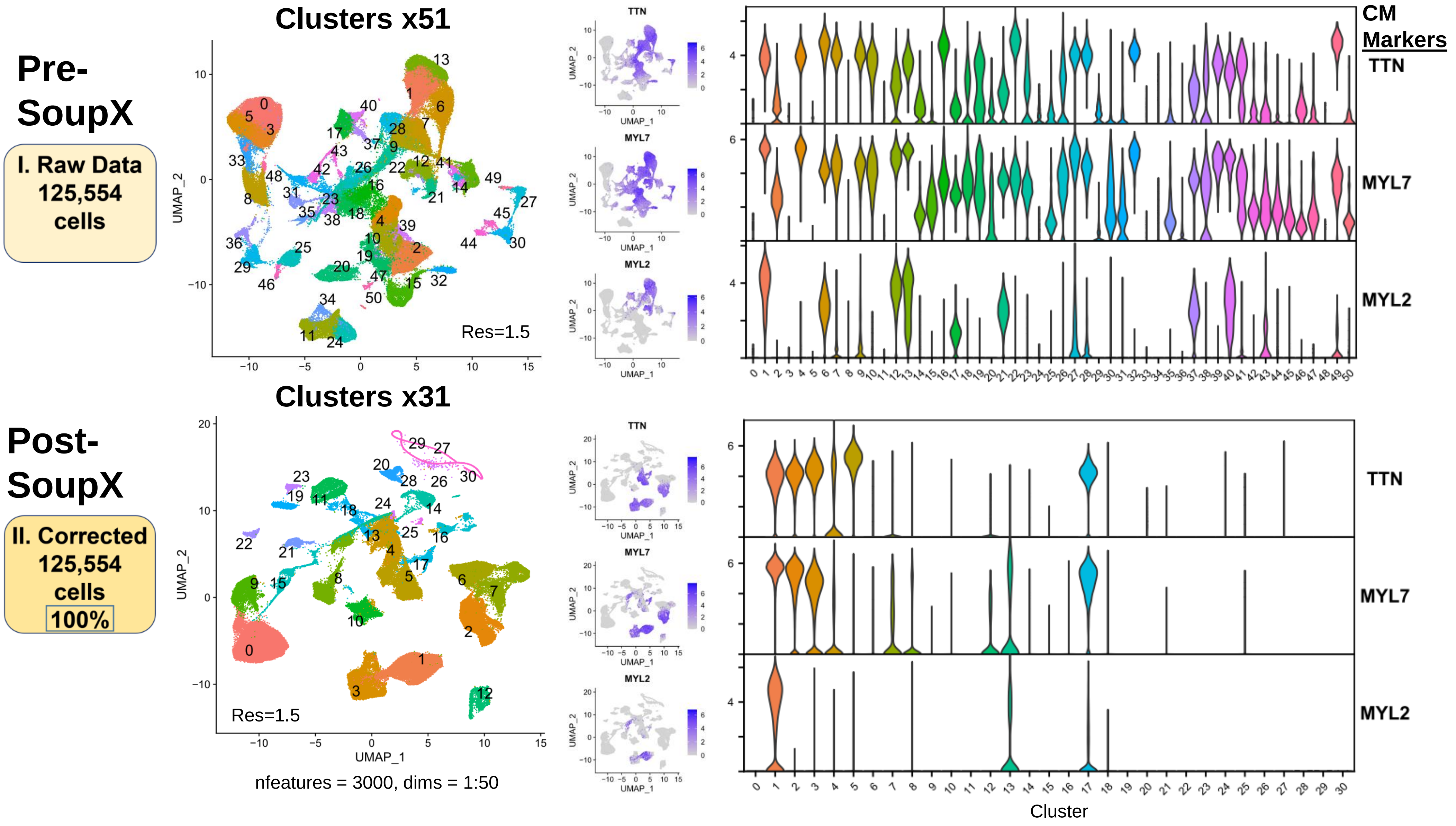

Fig. S3 Processing

F. Cell Quality: Removal of Low-Quality Cells Using Seurat

II. Corrected
125,554 cells
100%

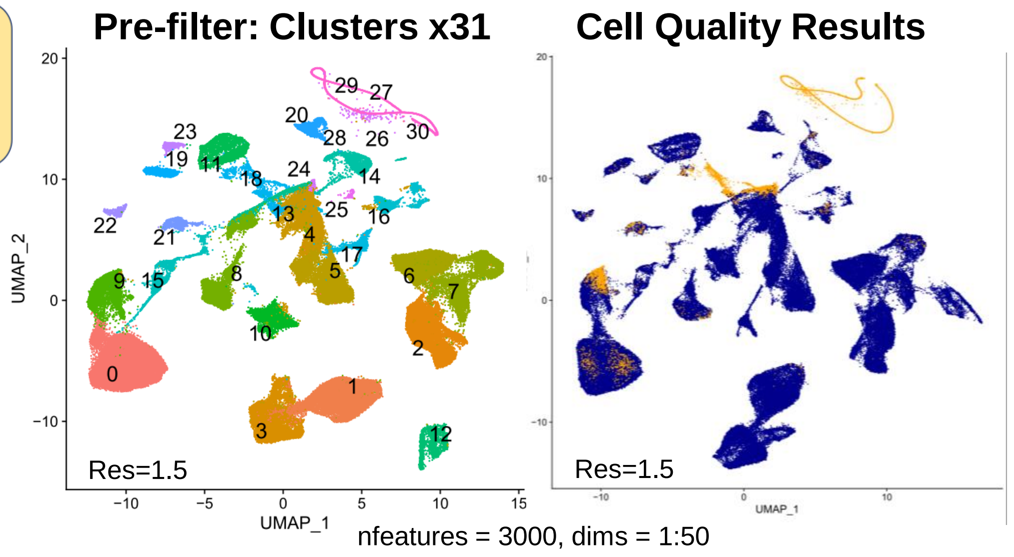

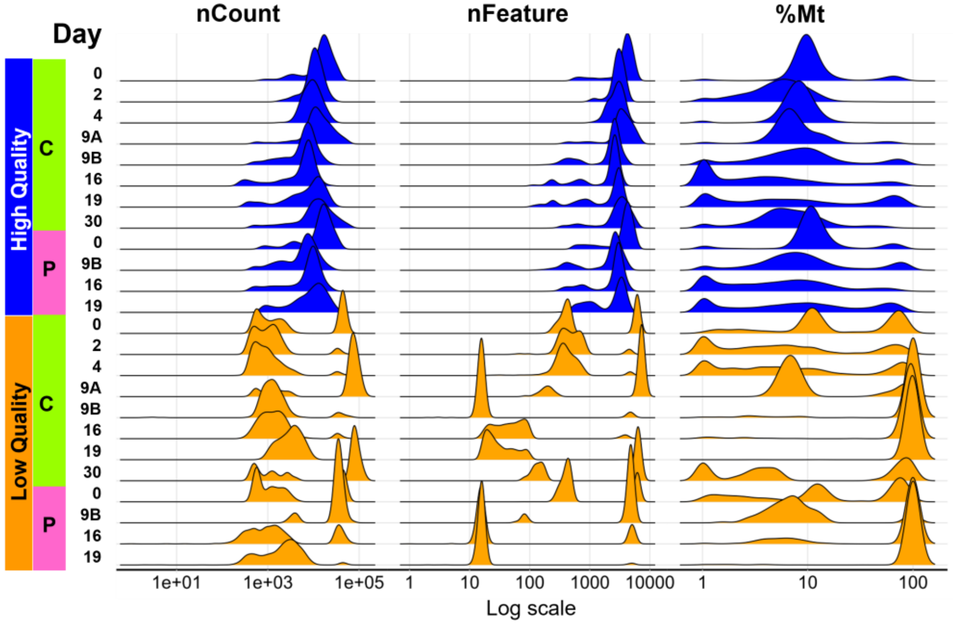

Cell Quality Results

High: 117,282 (93%) **Low: 8,272 (7%)**

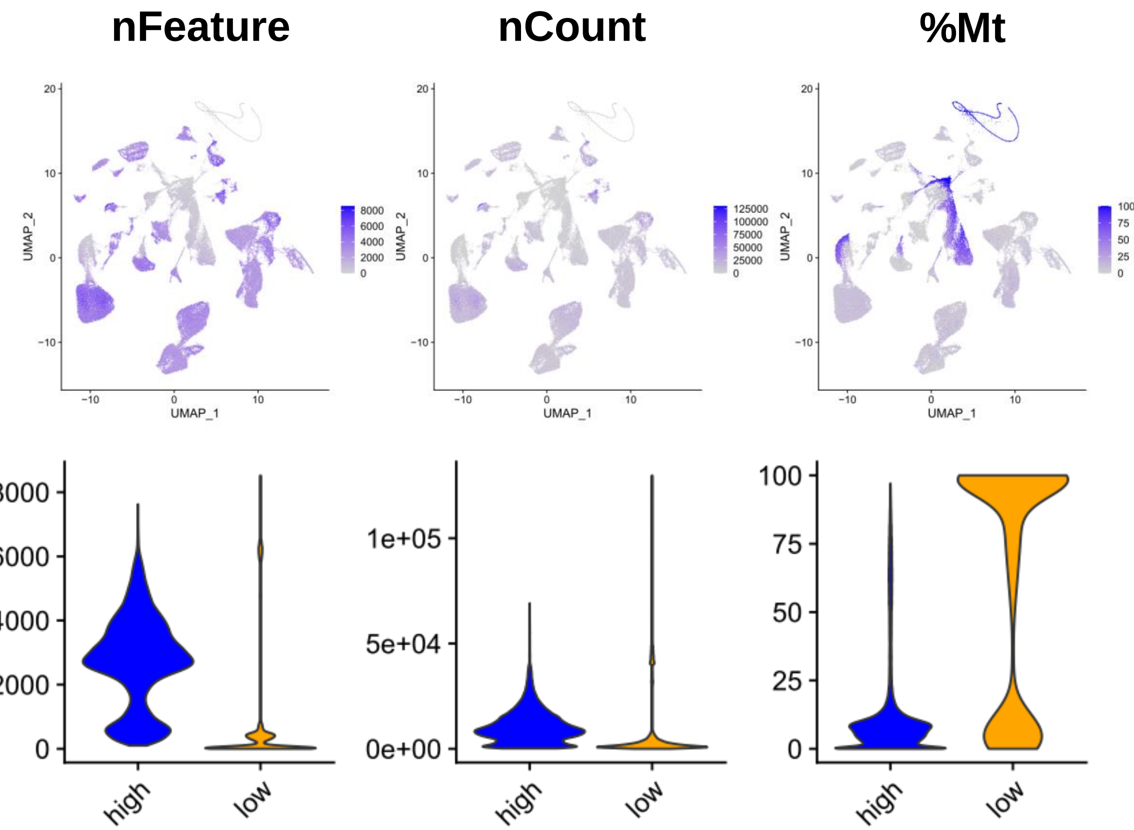

Seurat Filtering Parameters:
nFeature < 100-900, nCount > 30K-70K, %Mt N/A

Fig. S3 Processing

G. Doublets: Identification and Removal Using DoubletFinder (DF)

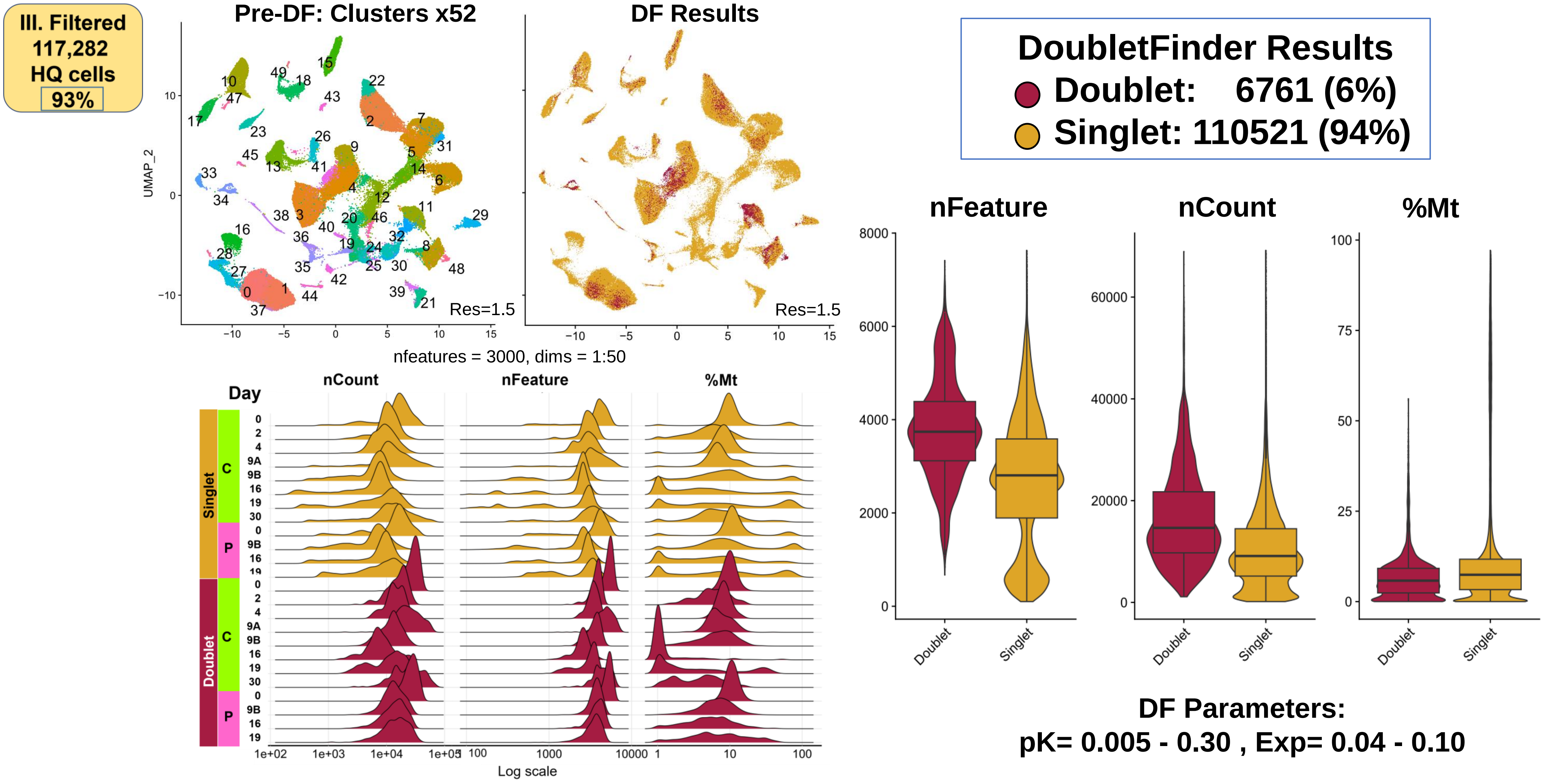

Fig. S4 Annotation

Workflow Step-II: Clustering and Annotation

A. Summary: Single Sample Data for Cell Annotation

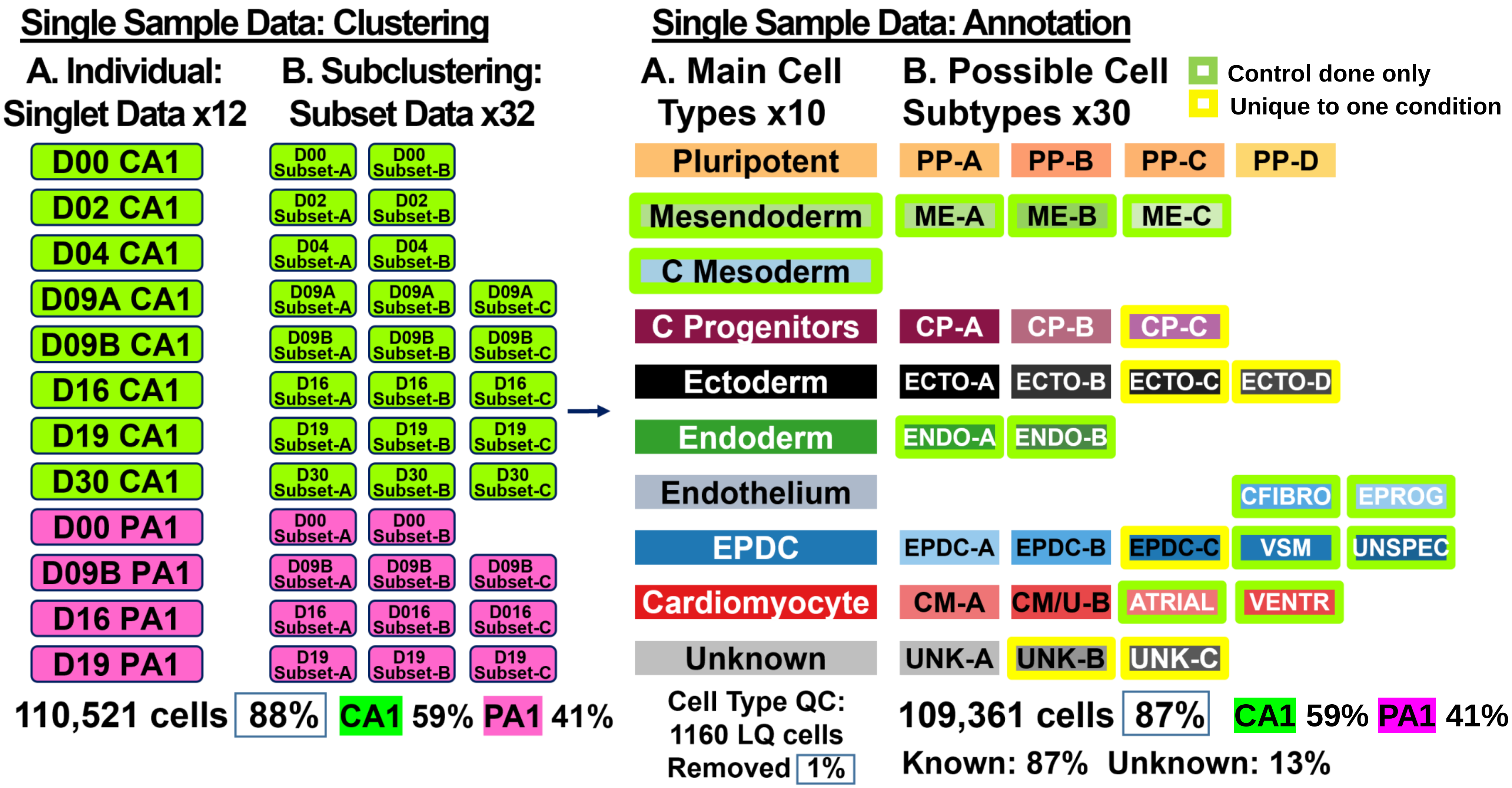

Fig. S4 Annotation

B. Summary: Merged Single Sample Data for Cell Annotation (110,521 Total Cells)

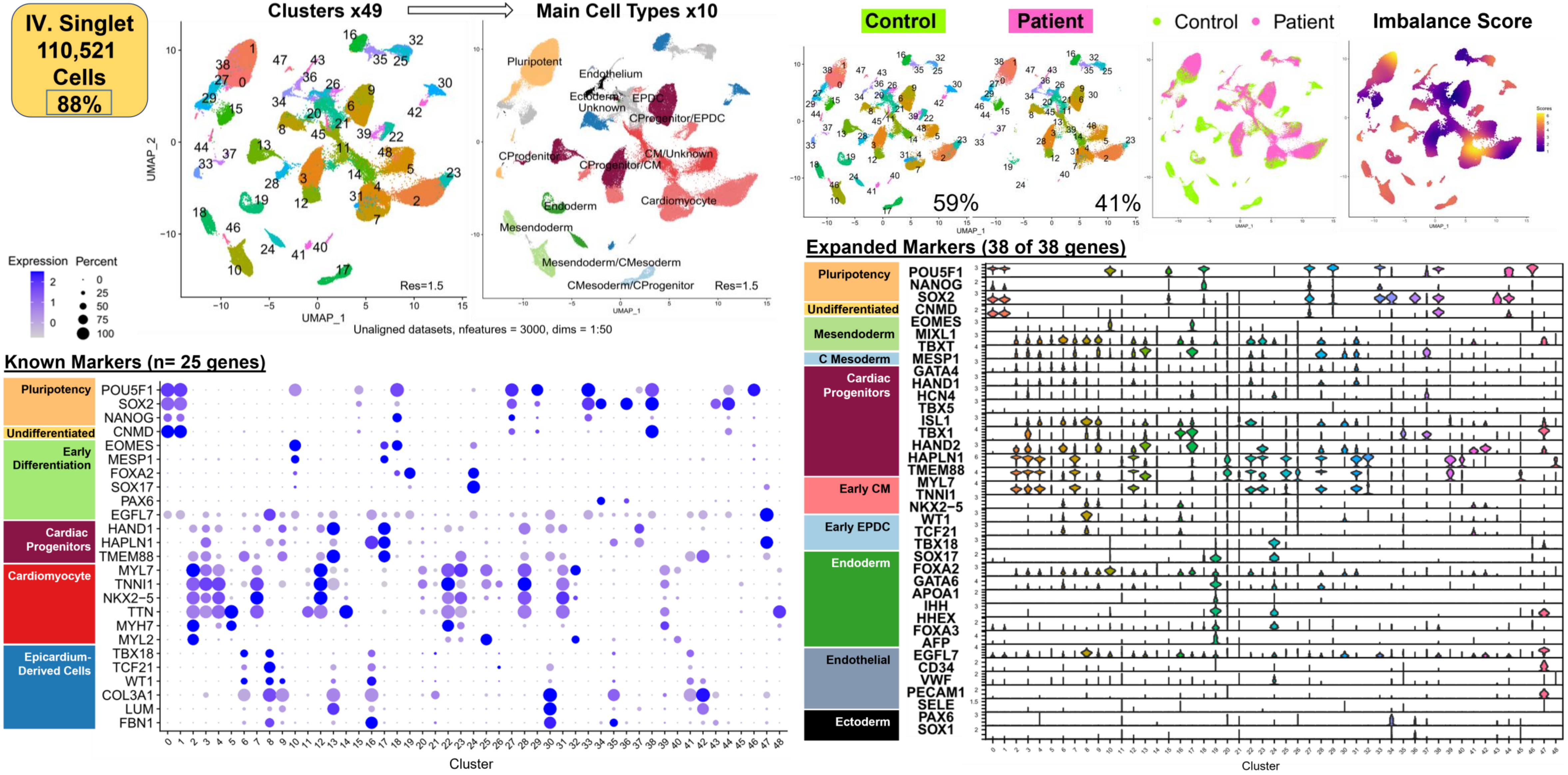

Fig. S5 Clustering

Workflow Step-II: Single Sample Data Results

Individual Analyses of Singlet Data for Main Cell Types

A. Summary: Raw Data to Annotated Clusters

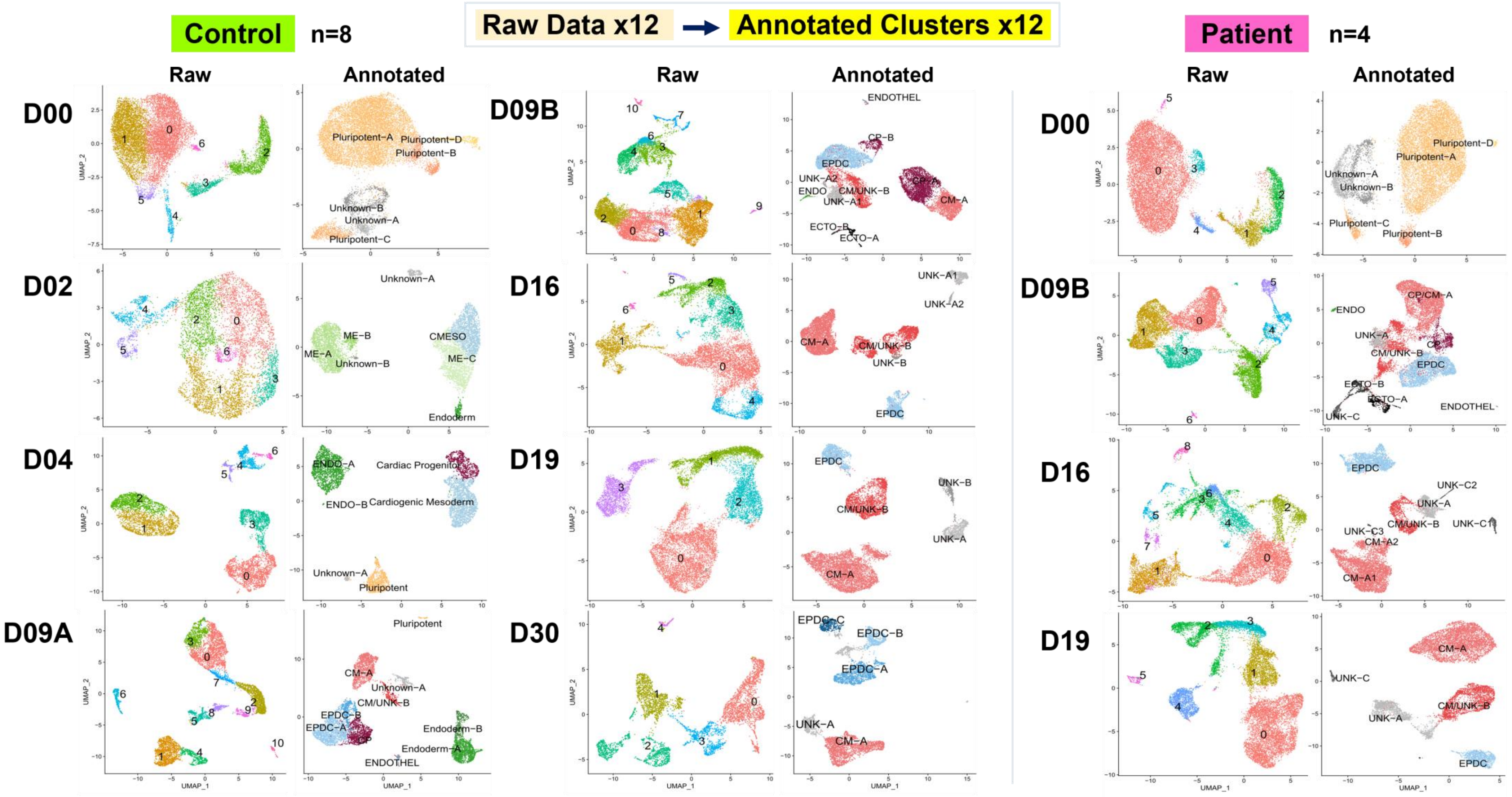

Fig. S5 Clustering

B. Single Sample Data Analyses: Control Samples (n=8)

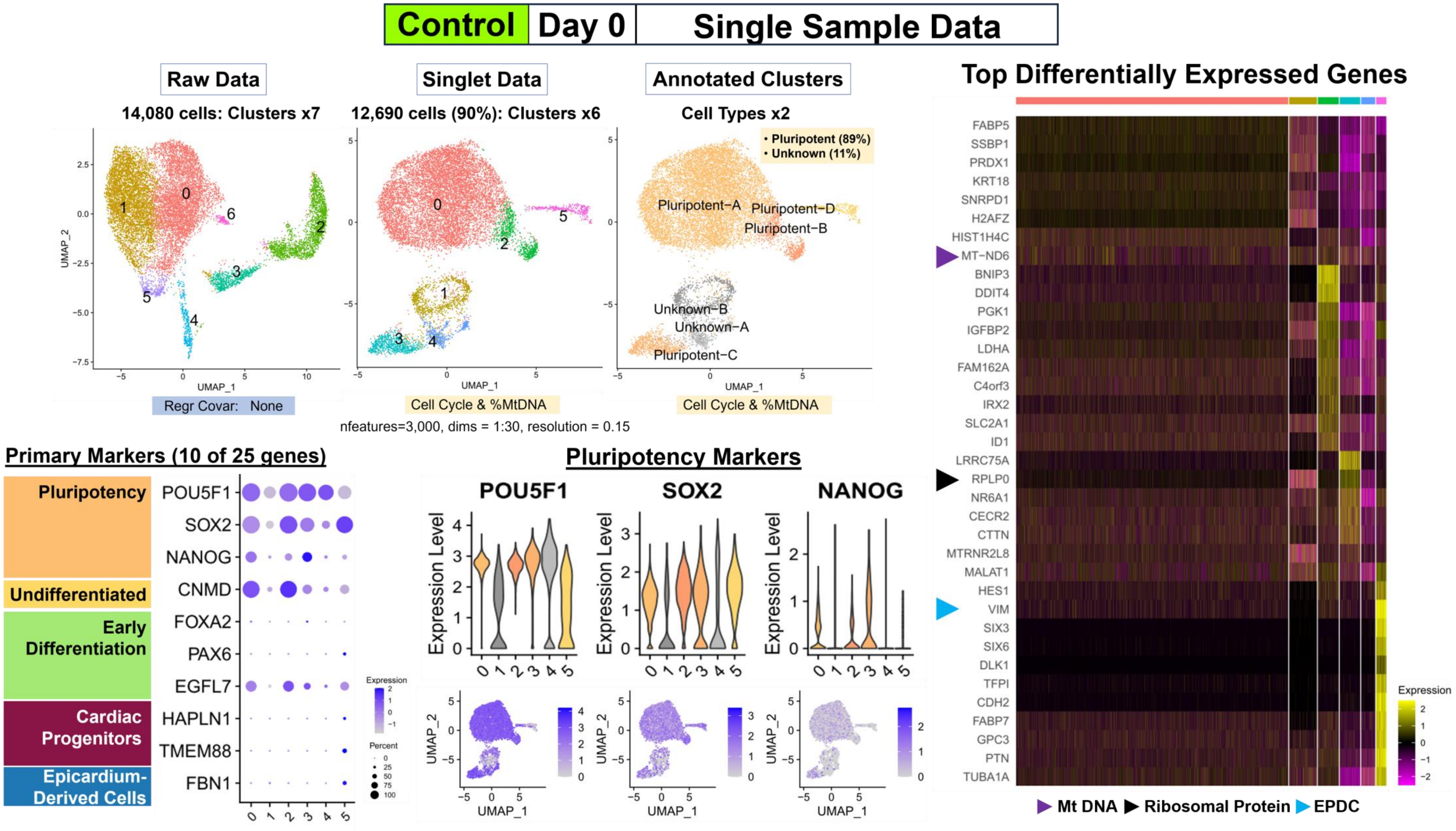

Fig. S5 Clustering

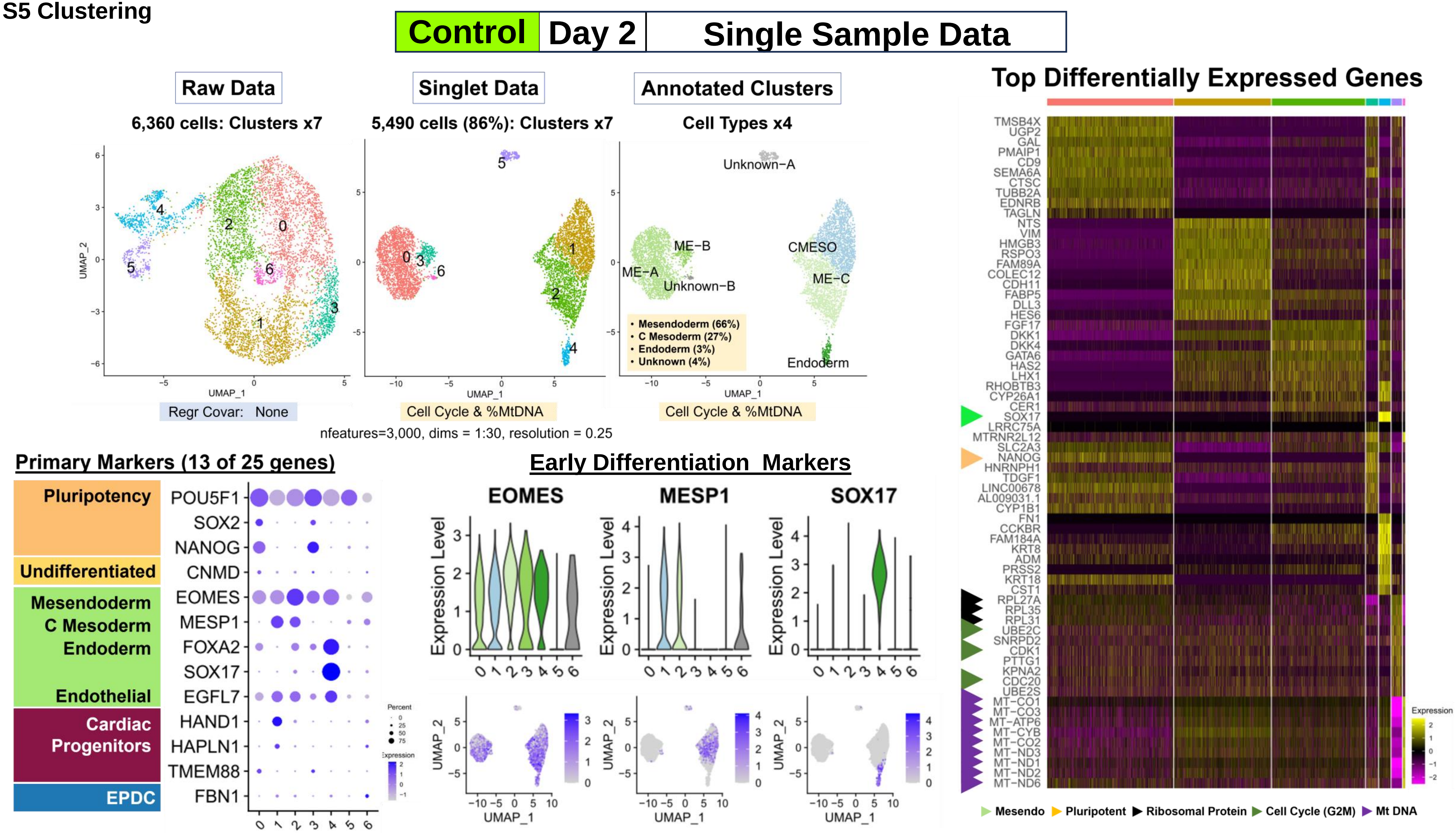

Fig. S5 Clustering

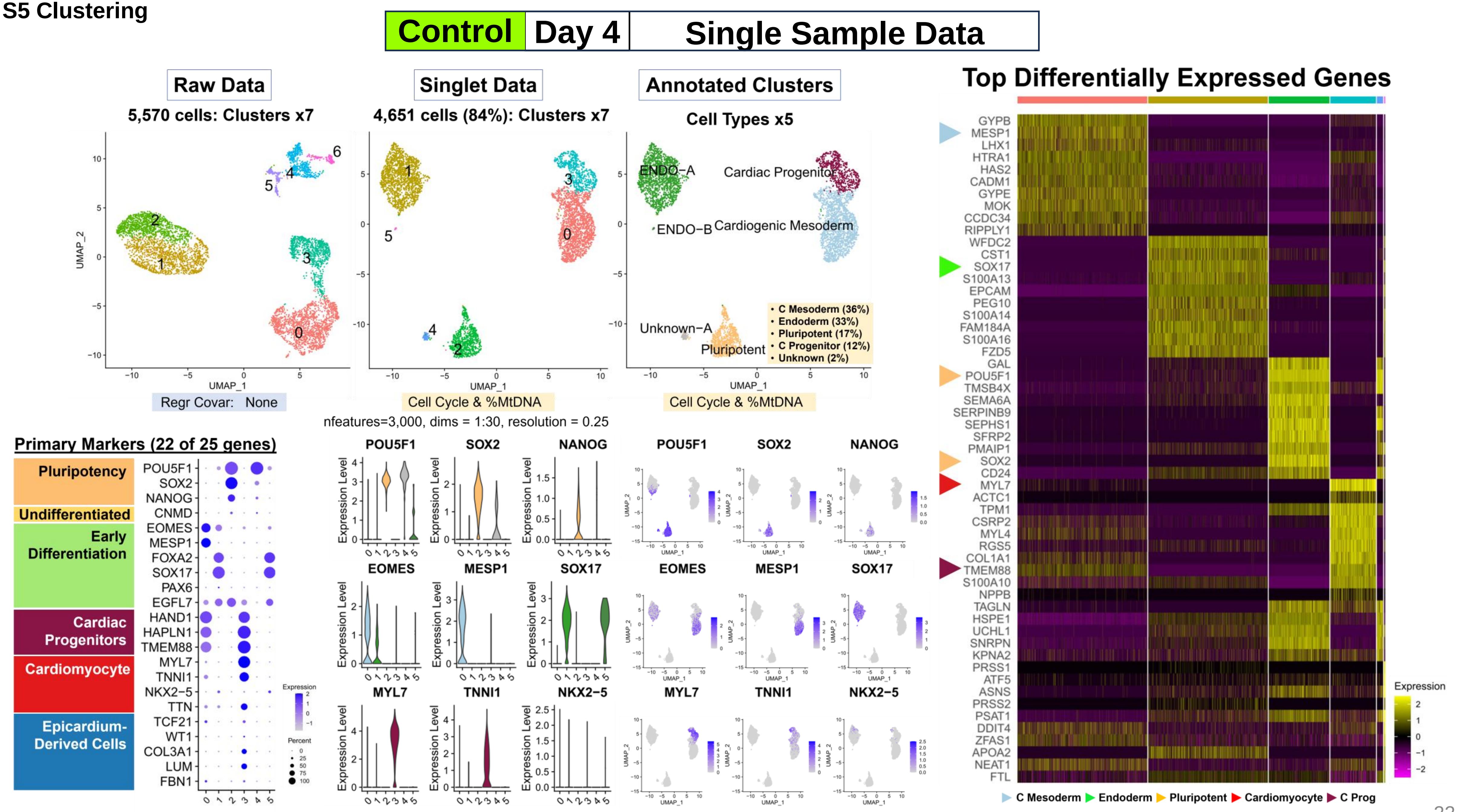

Fig. S5 Clustering

Control | Day 9A | Single Sample Data

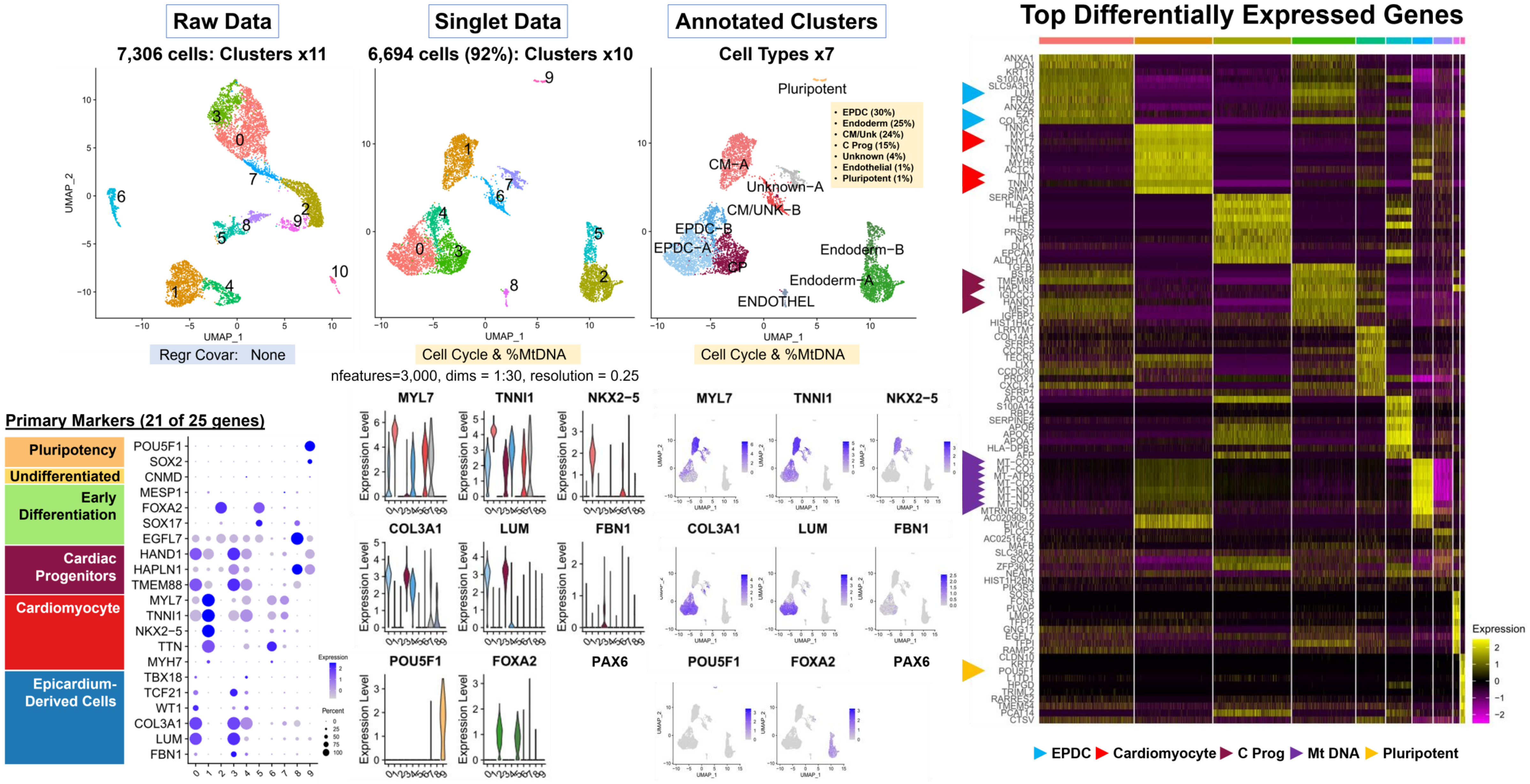

Fig. S5 Clustering

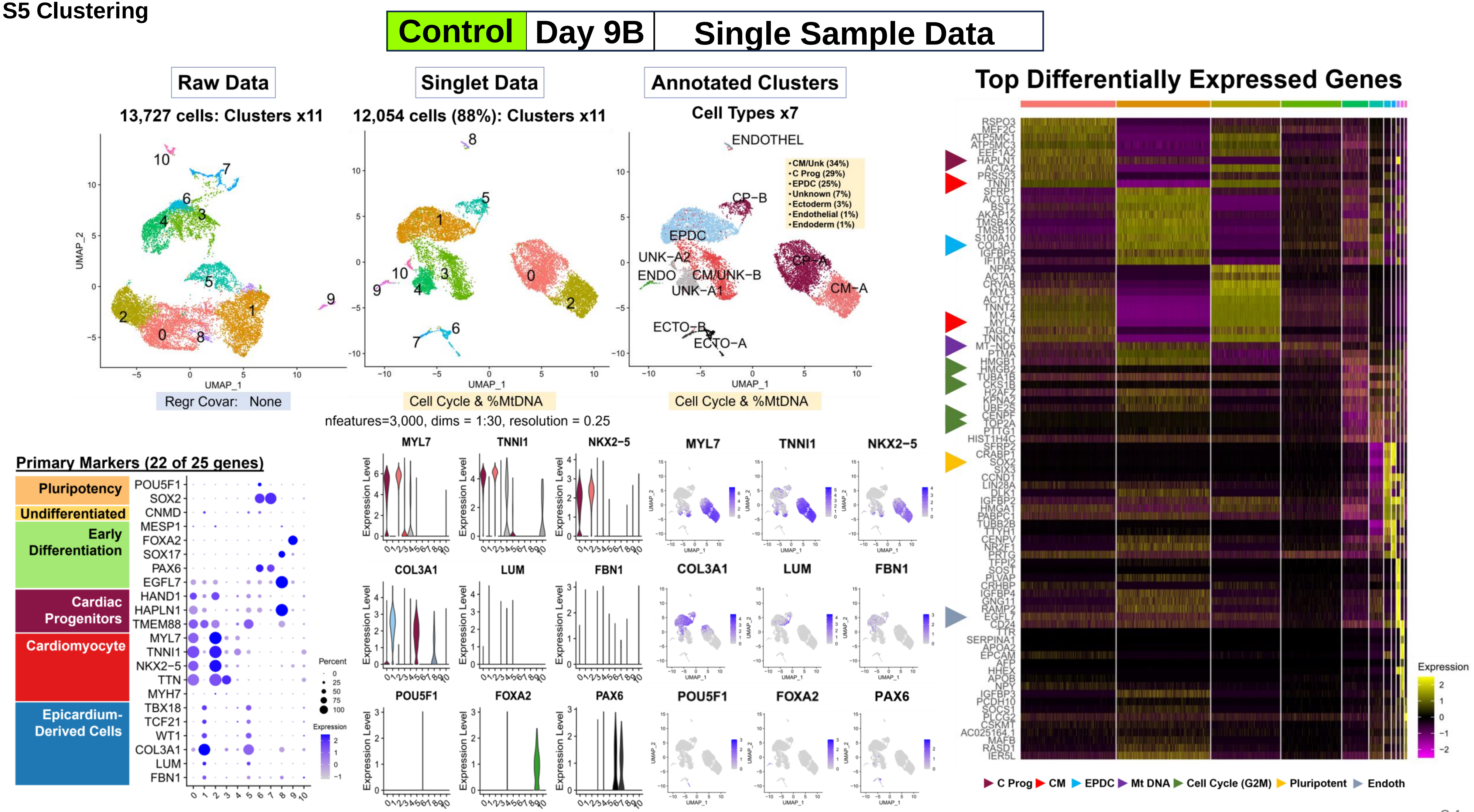

Fig. S5 Clustering

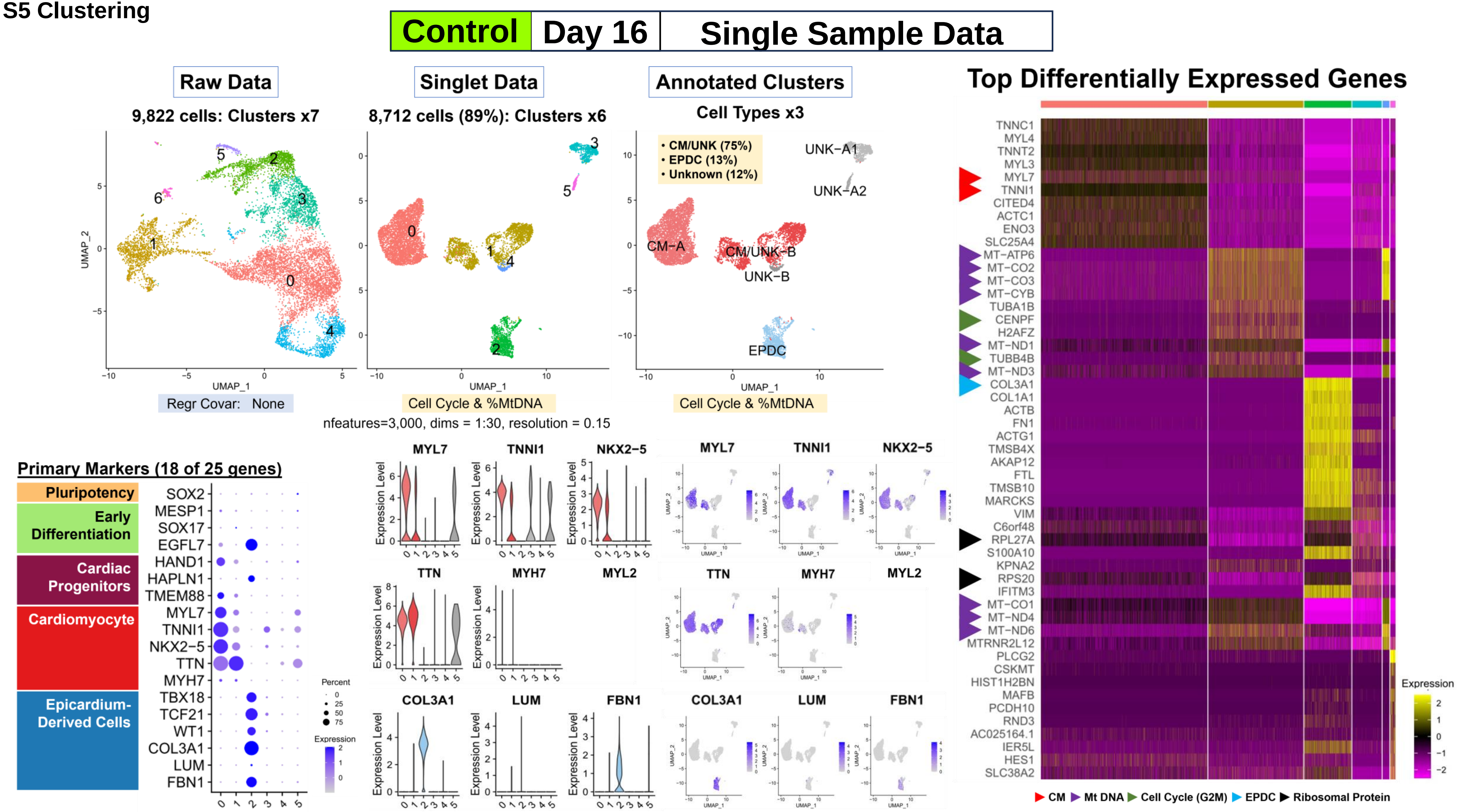

Fig. S5 Clustering

Control | Day 19 | Single Sample Data

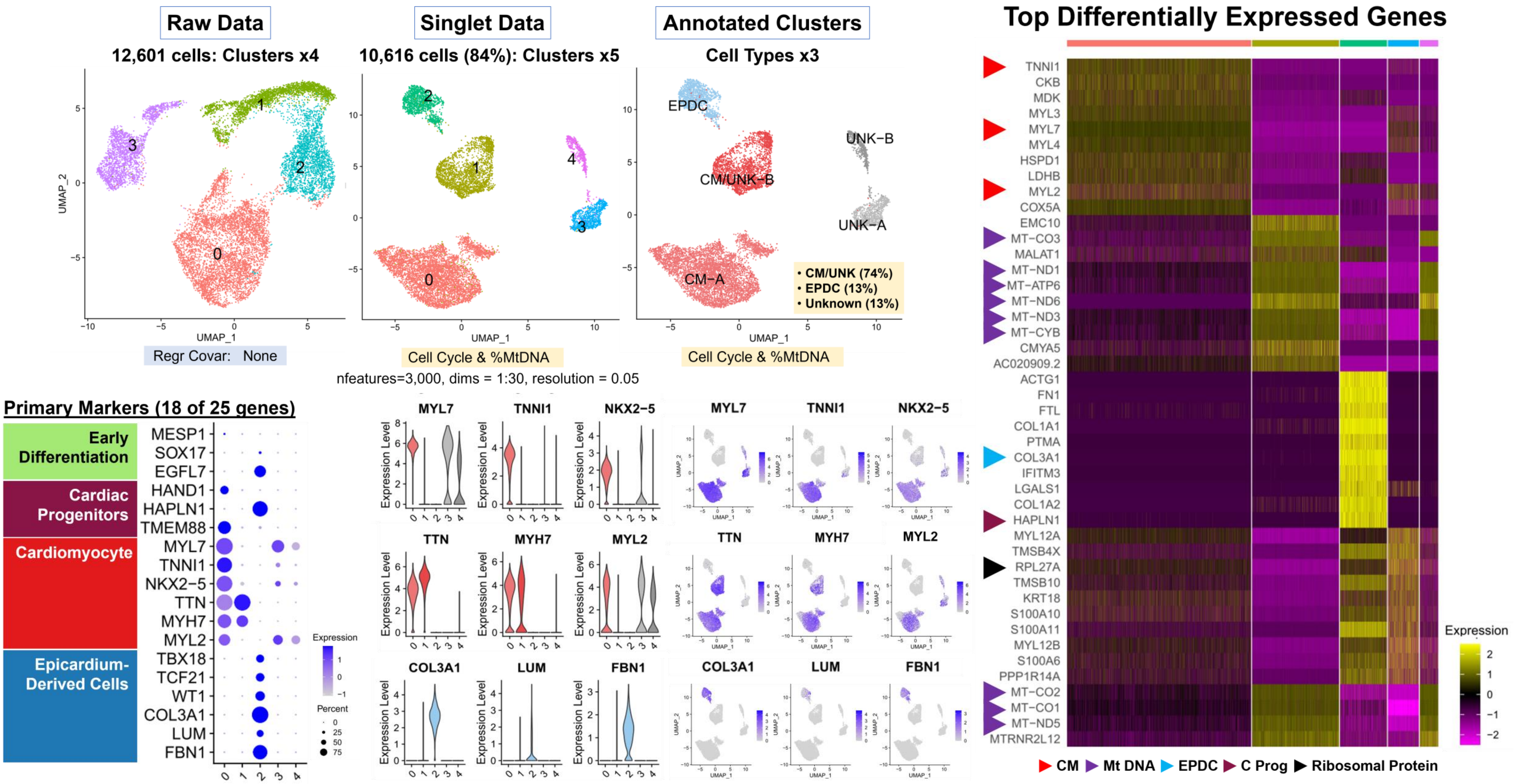

Fig. S5 Clustering

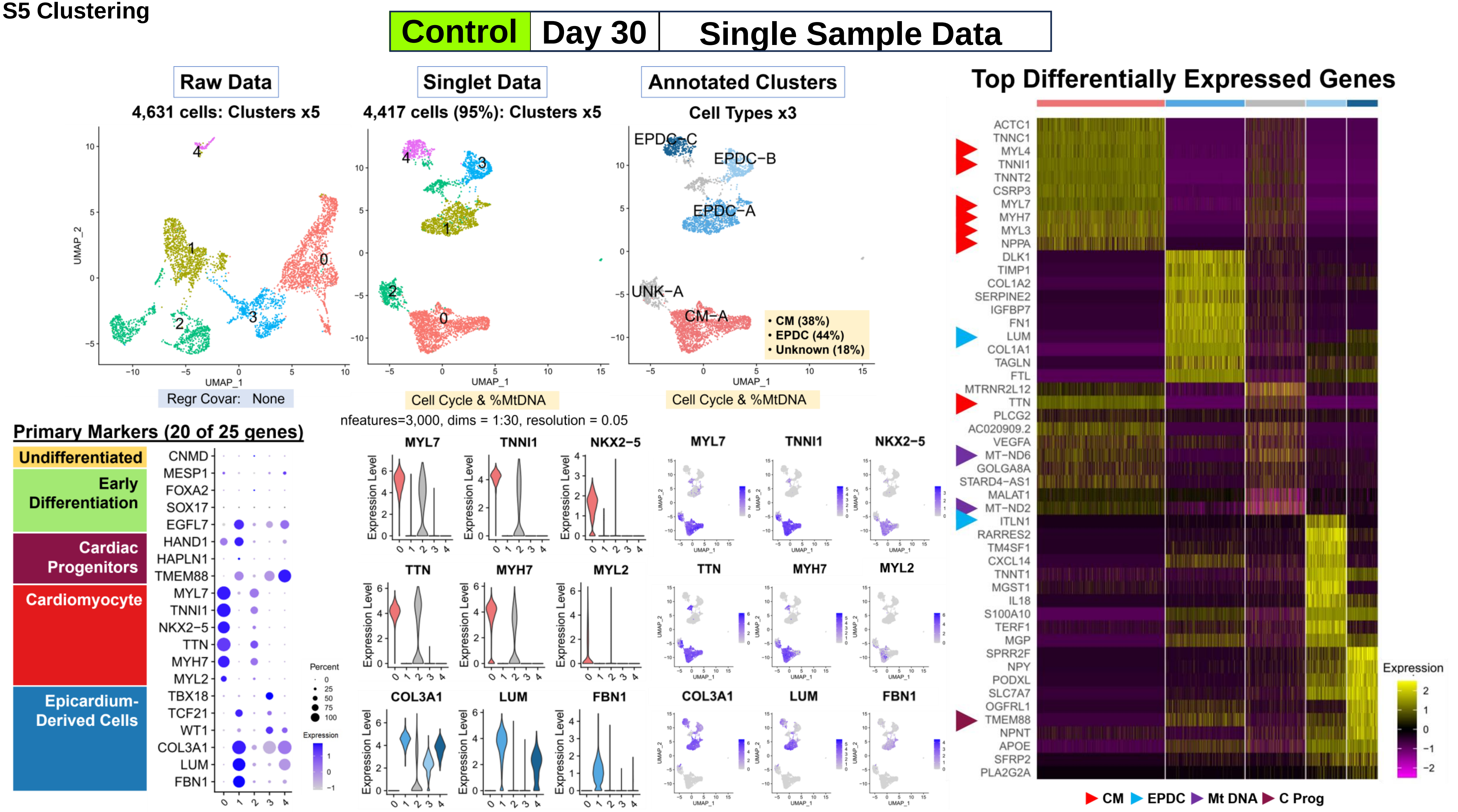

Fig. S5 Clustering

C. Single Sample Data Analyses: Patient Samples (n=4)

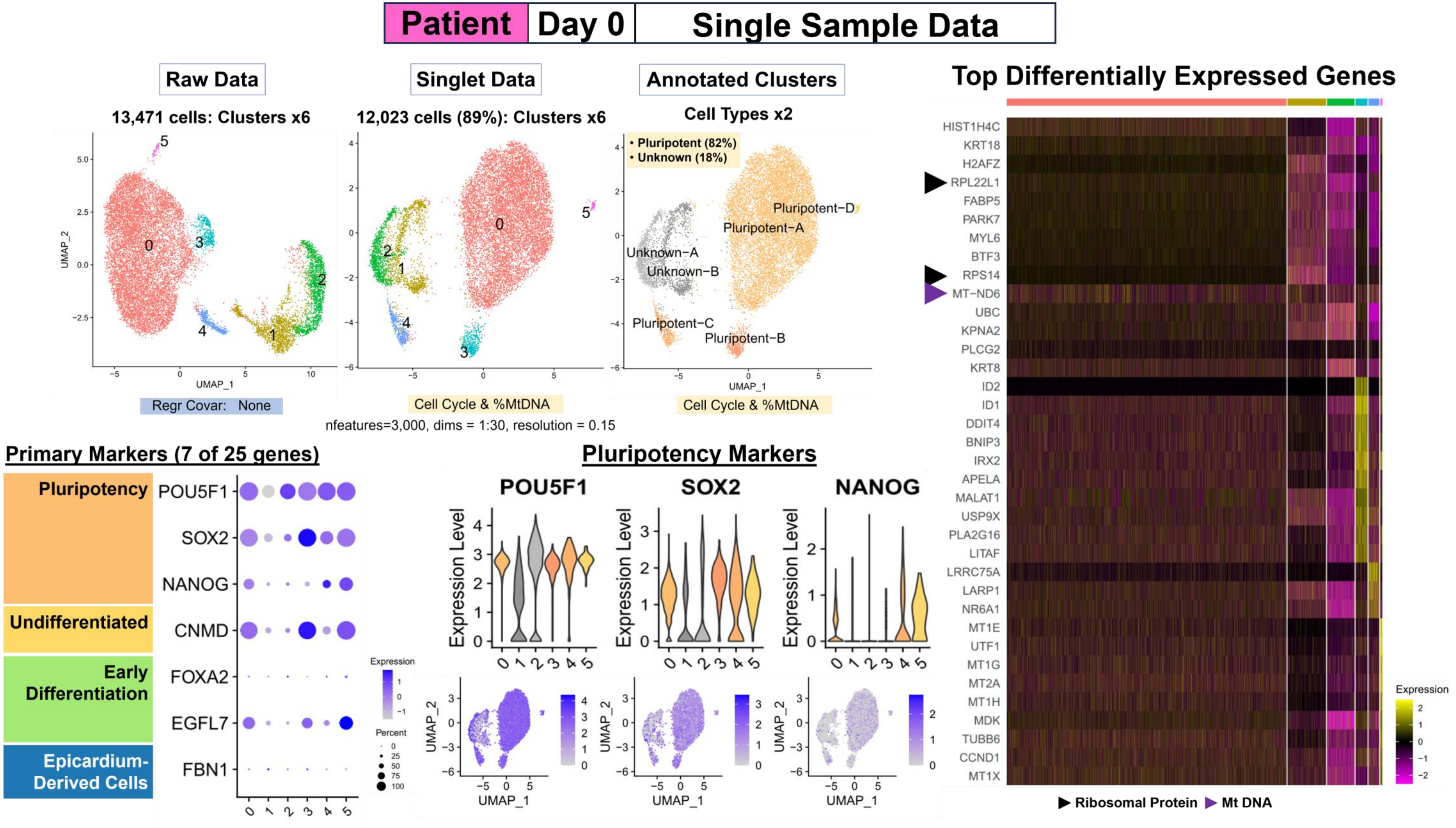

Fig. S5 Clustering

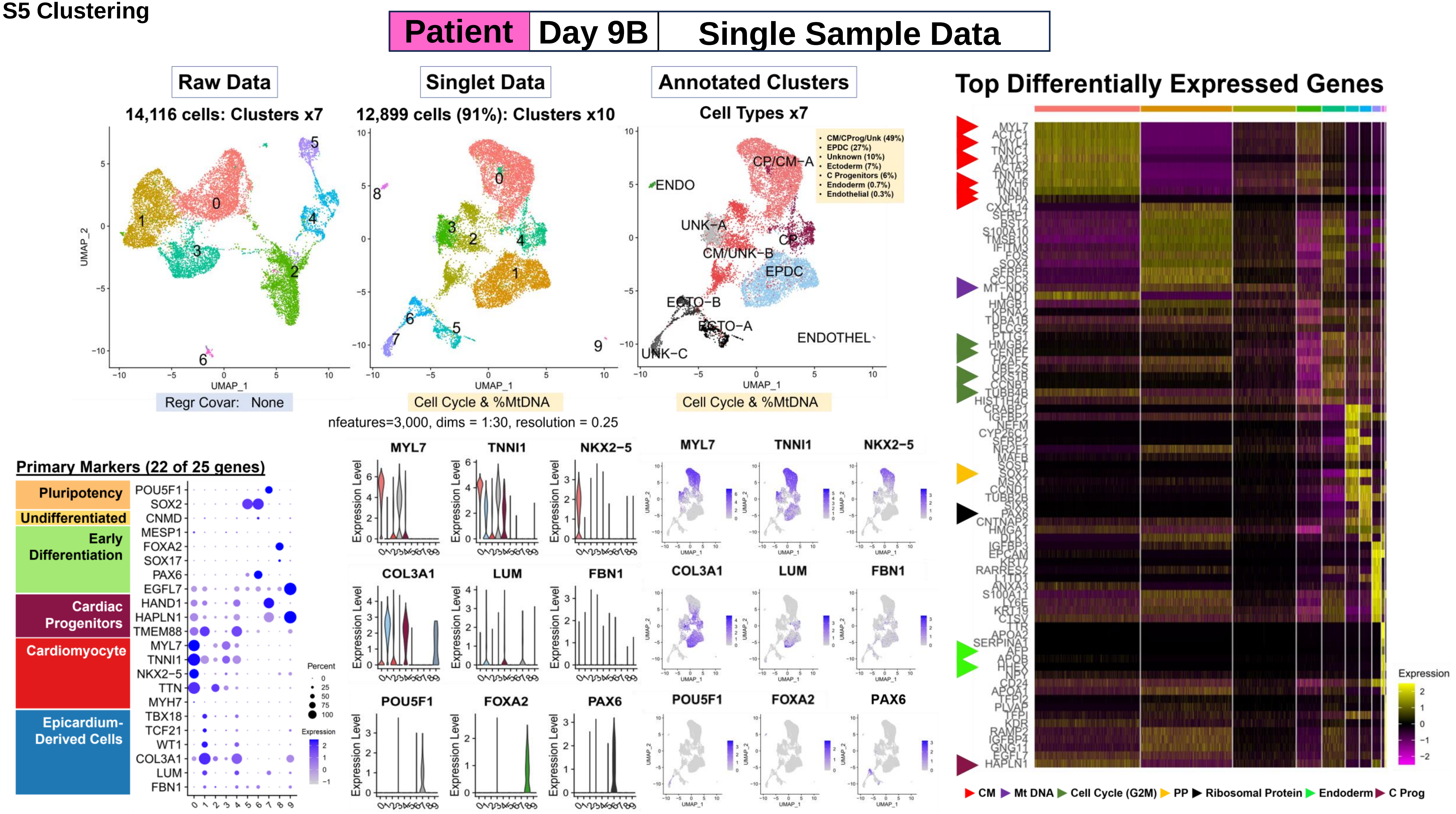

Fig. S5 Clustering

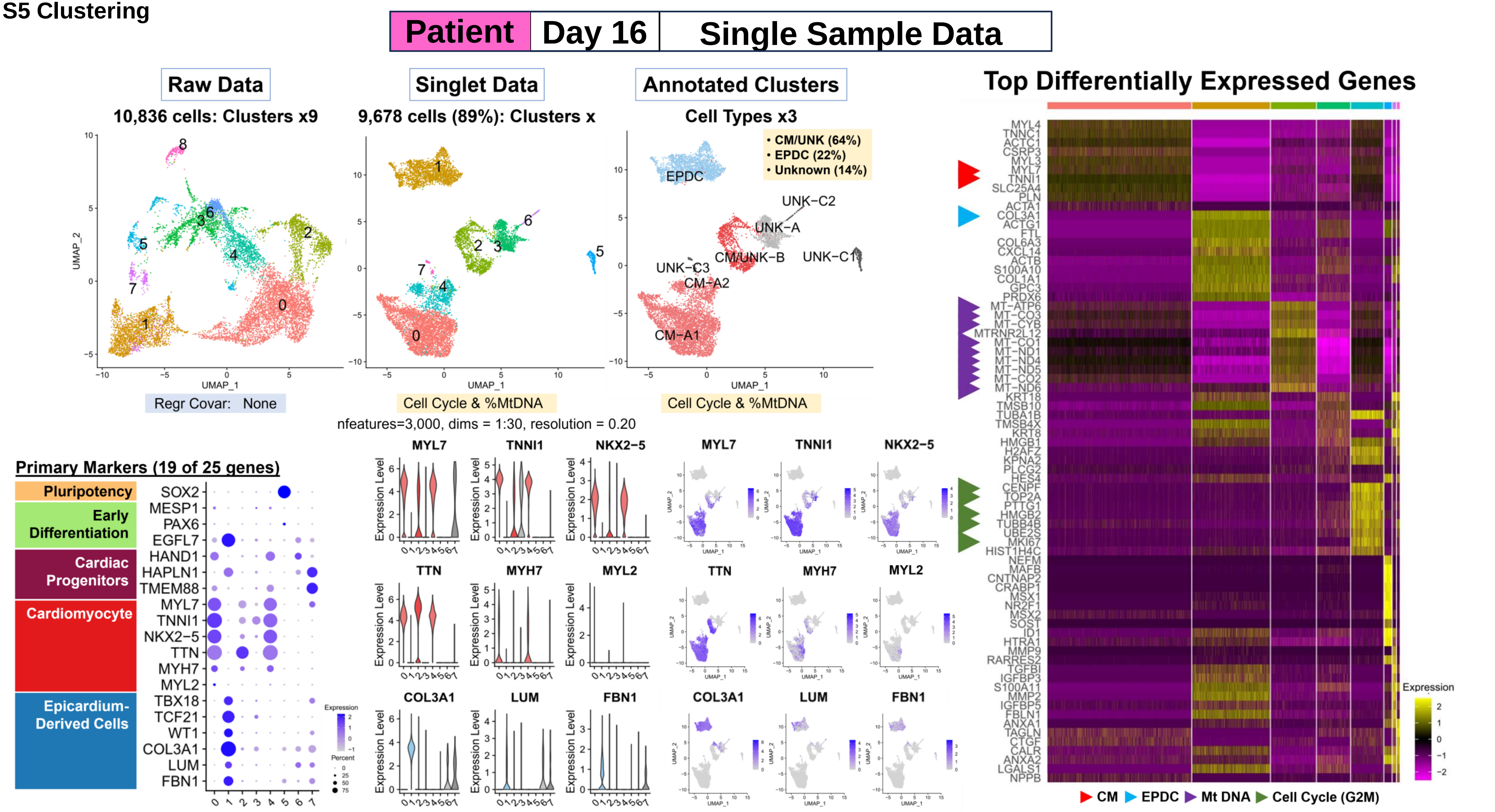

Fig. S5 Clustering

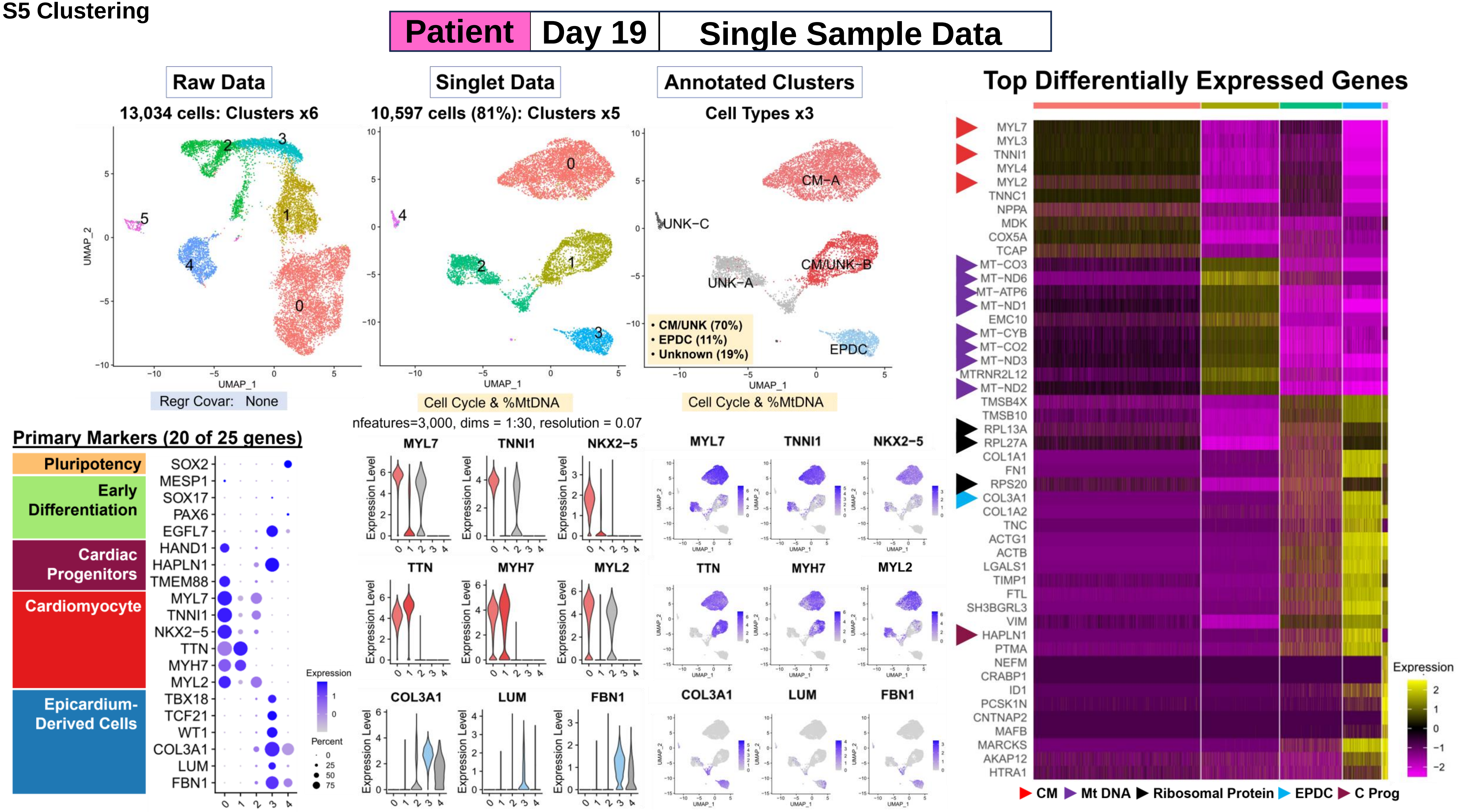

Fig. S6 Subcluster

Workflow Step-II: Single Sample Data Results

Subcluster Analyses of Subset Data for Possible Cell Subtypes

A. Summary: Annotated Clusters to Annotated Subsets

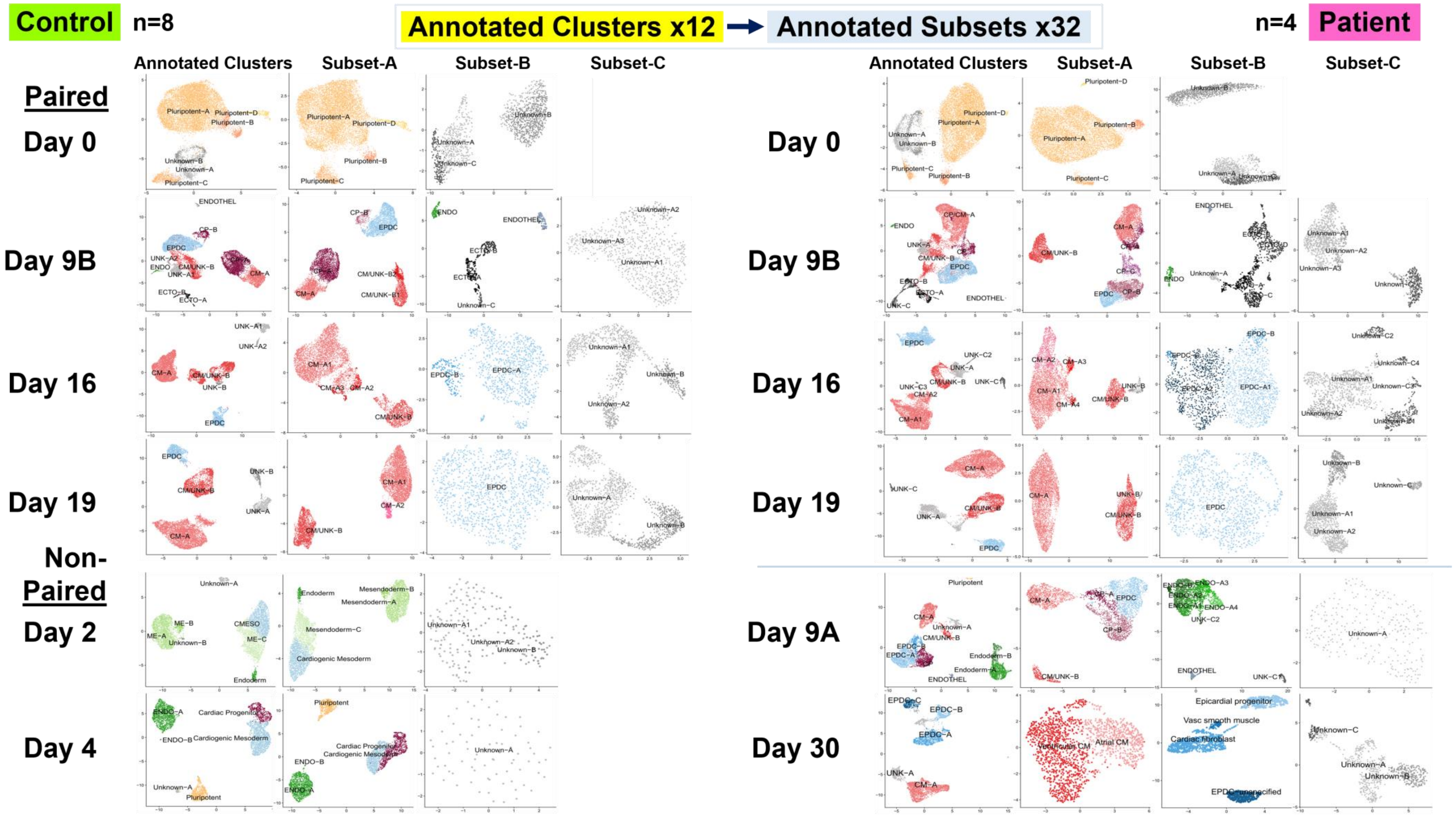

Fig. S6 Subcluster

B. Subcluster Analyses: Control Samples (n=8) to Annotated Subset Data (n=21)

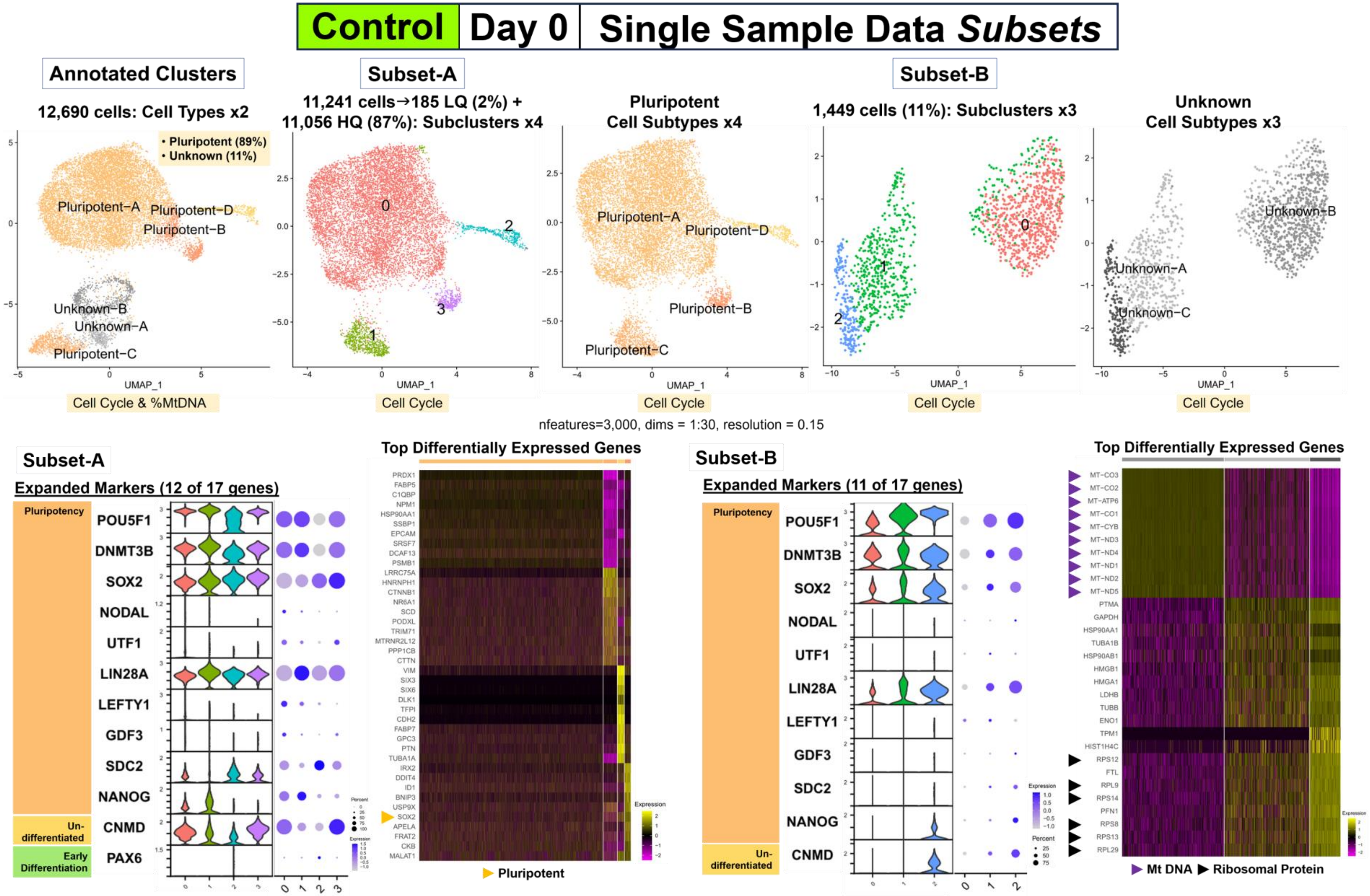

Fig. S6 Subcluster

Control | Day 2 | Single Sample Data *Subsets*

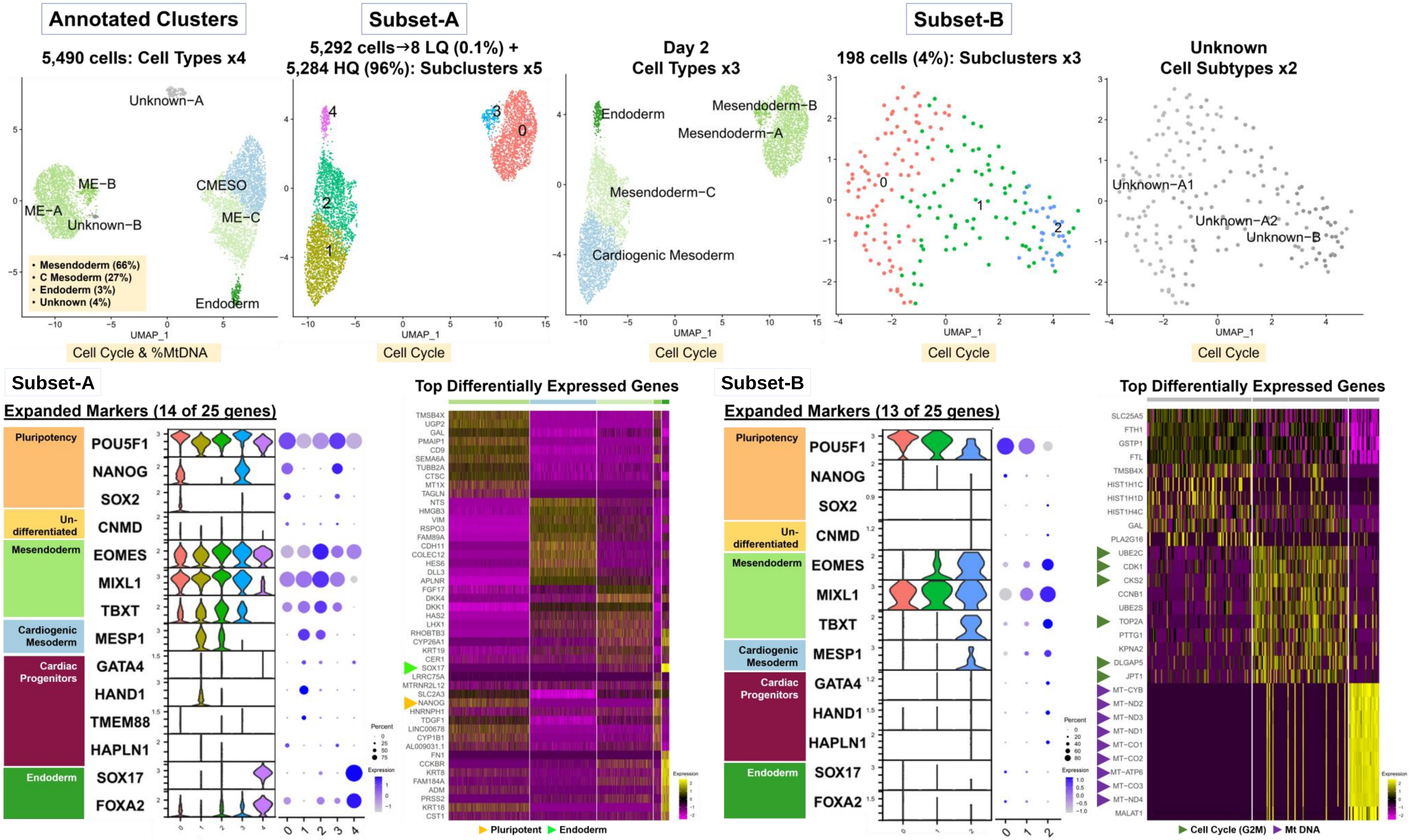

Fig. S6 Subcluster

Control | Day 4 | Single Sample Data *Subsets*

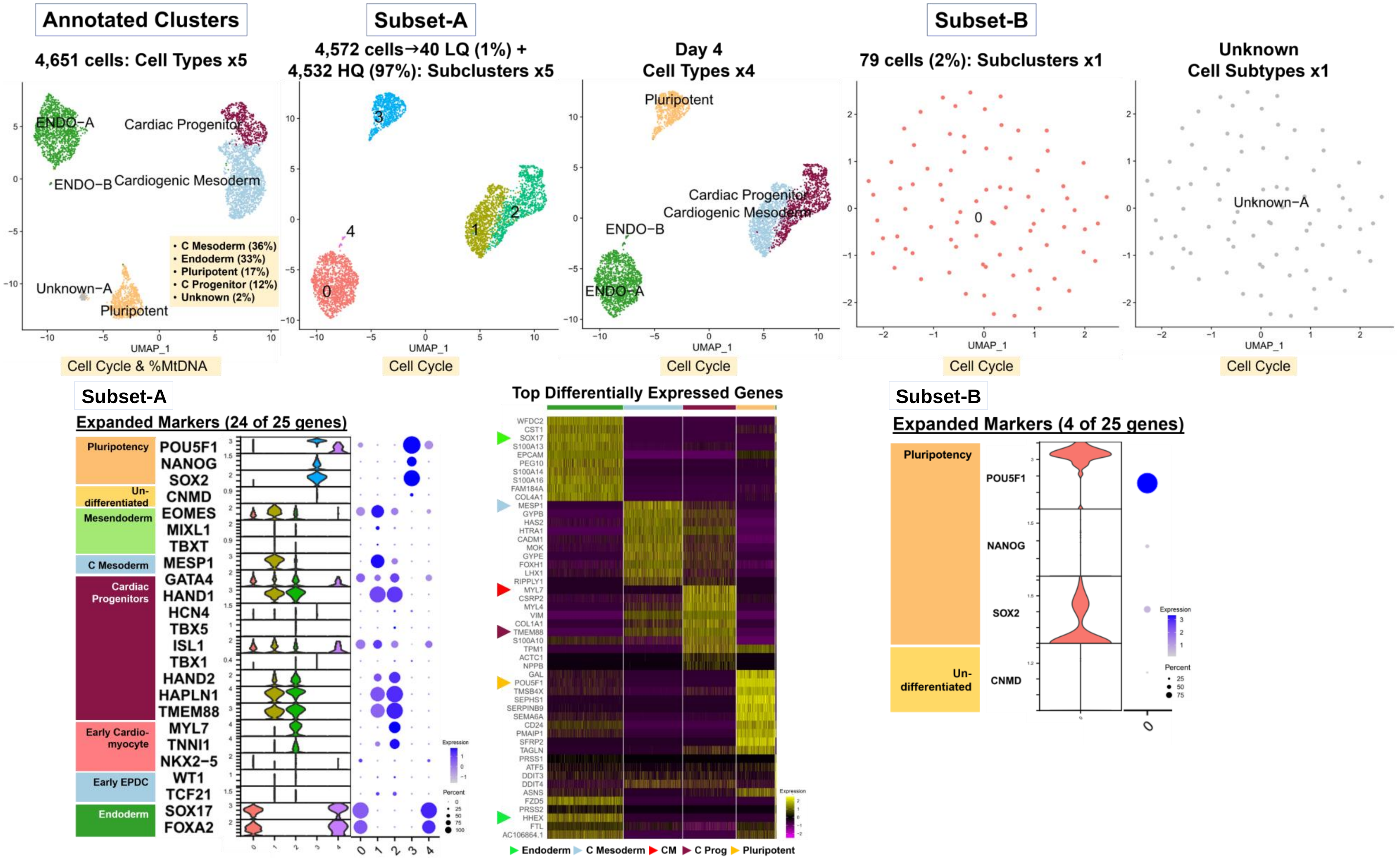

Fig. S6 Subcluster

Control | Day 9A | Single Sample Data *Subsets*

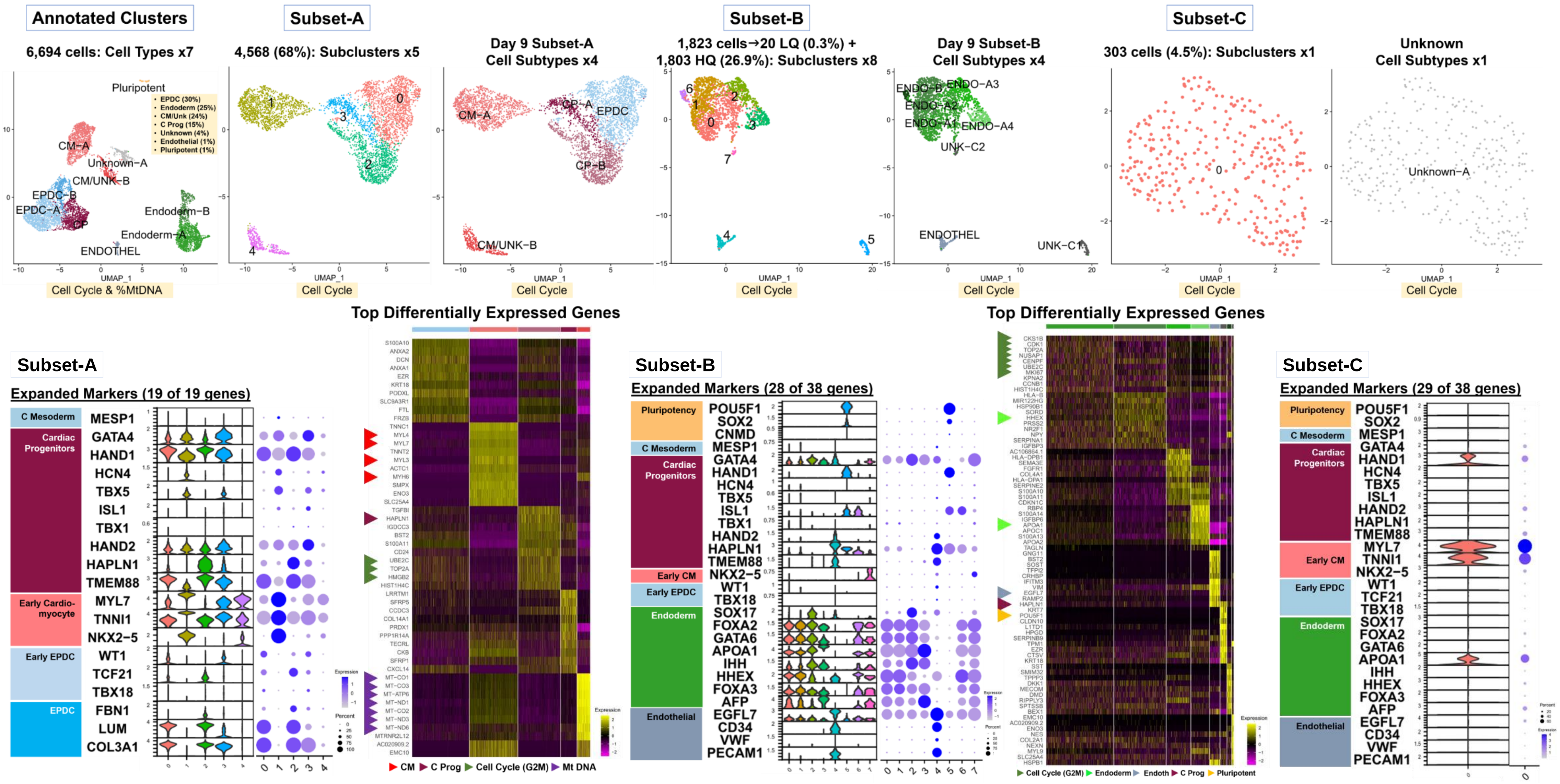

Fig. S6 Subcluster

Control | Day 9B | Single Sample Data *Subsets*

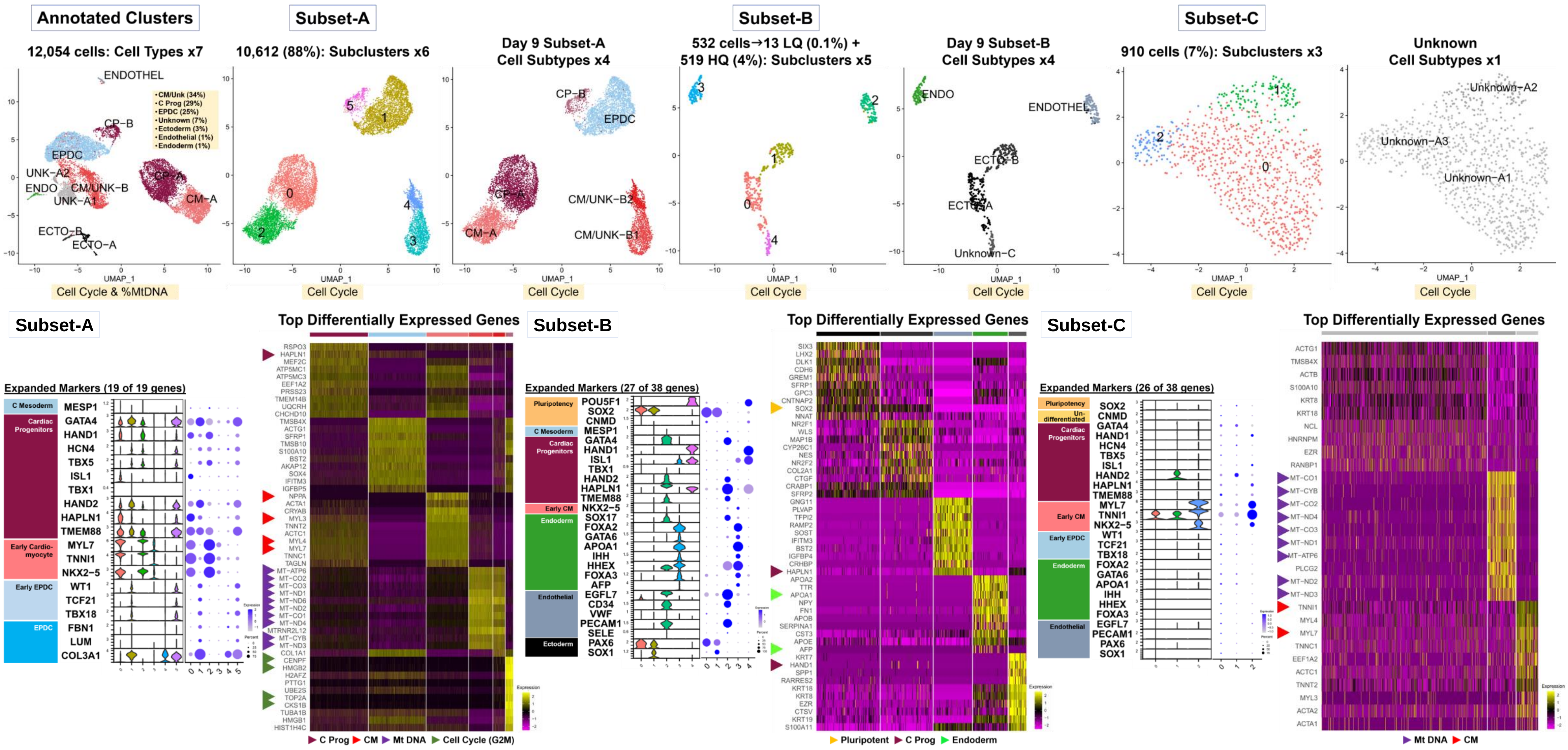

Fig. S6 Subcluster

Control | Day 16 | Single Sample Data *Subsets*

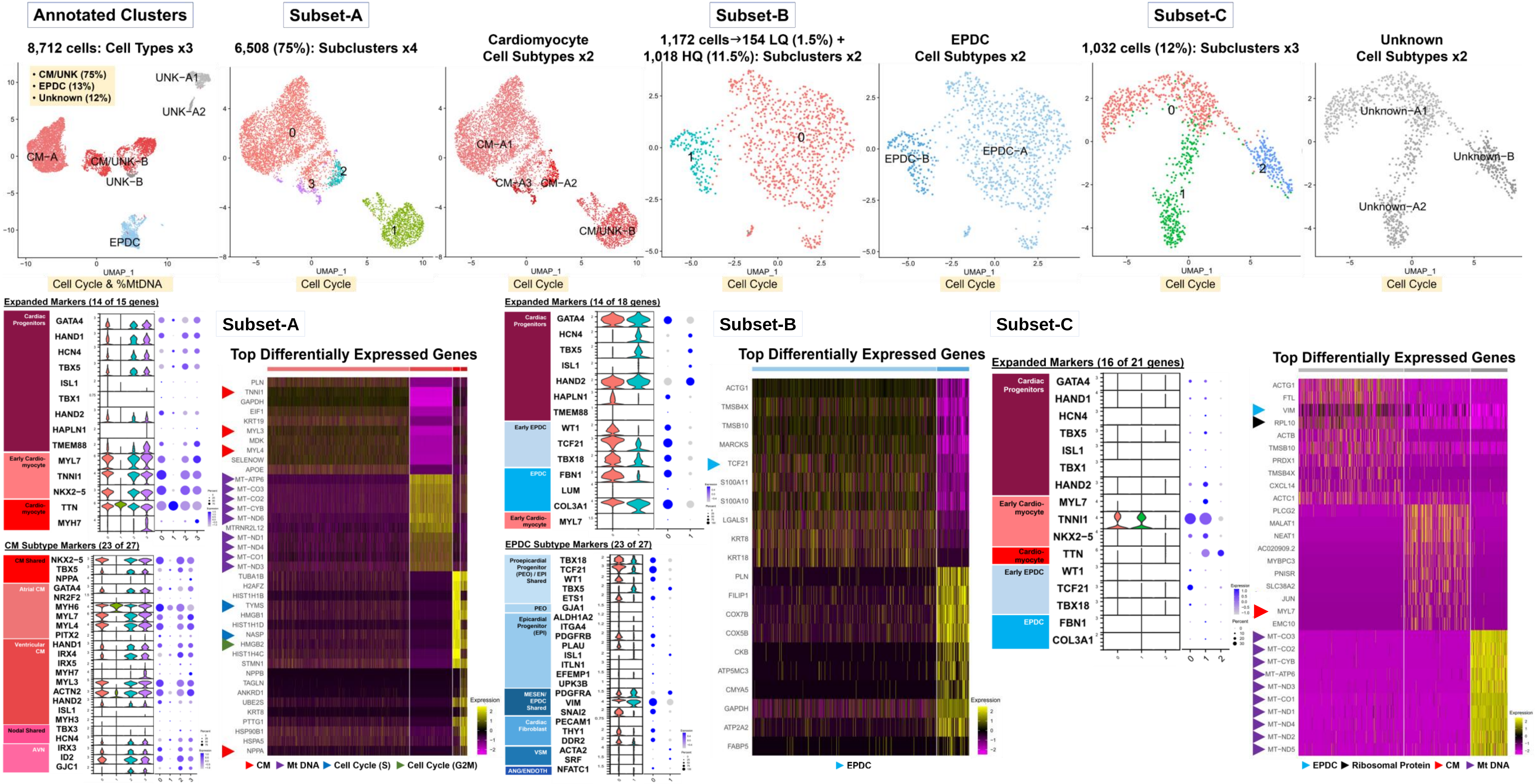

Fig. S6 Subcluster

Control | Day 19 | Single Sample Data *Subsets*

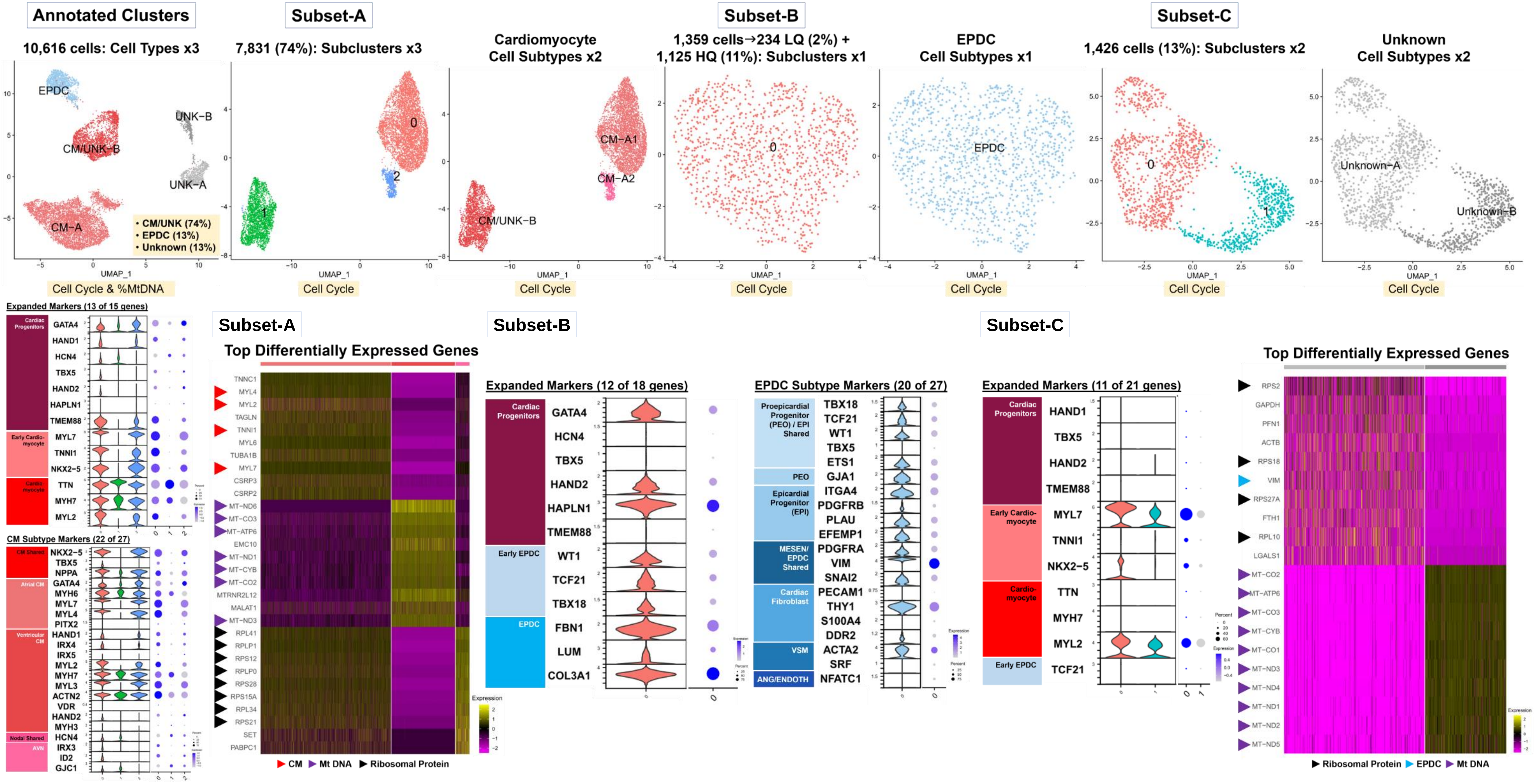

Fig. S6 Subcluster

Control | Day 30 | Single Sample Data *Subsets*

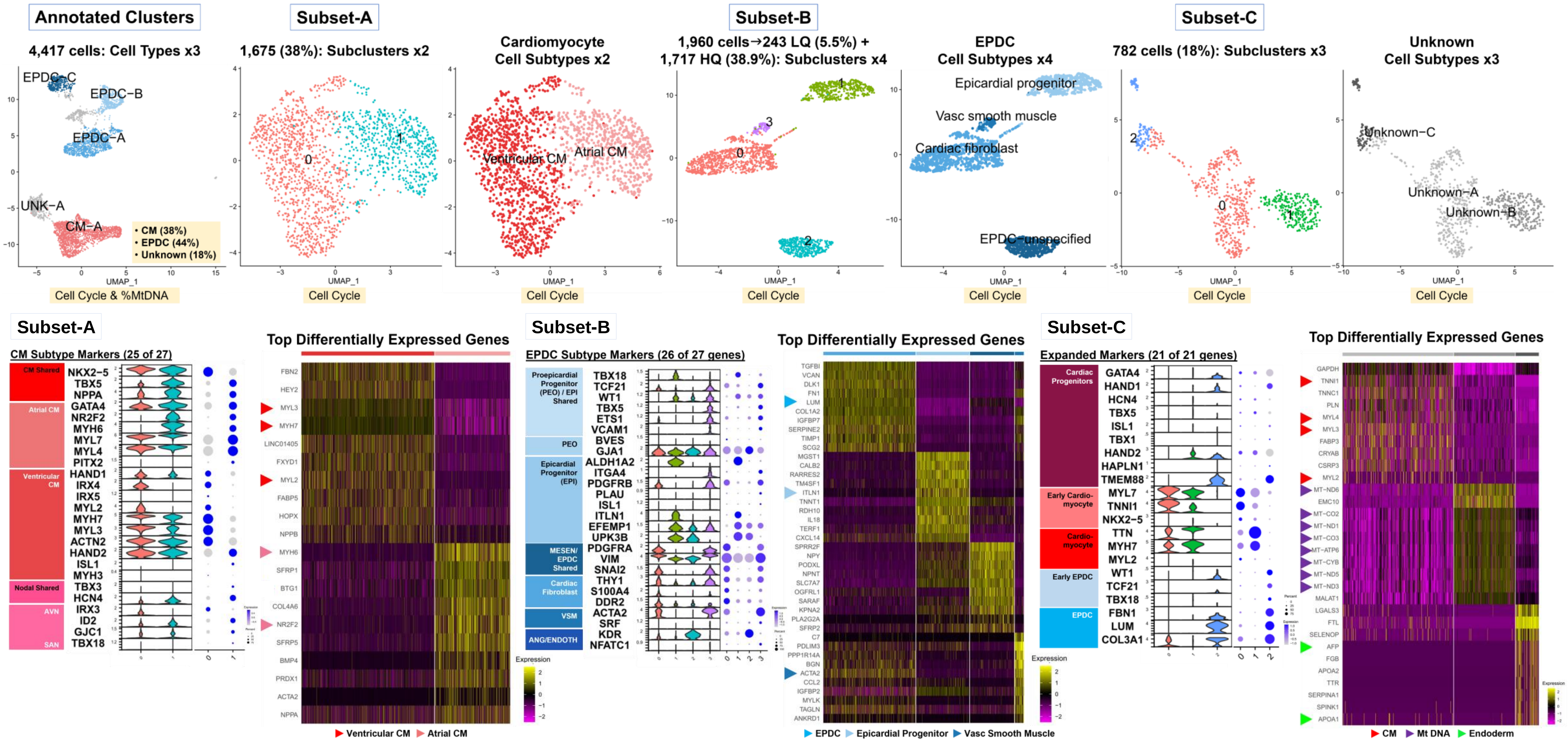

Fig. S6 Subcluster

C. Subcluster Analyses: Patient Samples (n=4) to Annotated Subset Data (n=11)

Patient | Day 0 | Single Sample Data *Subsets*

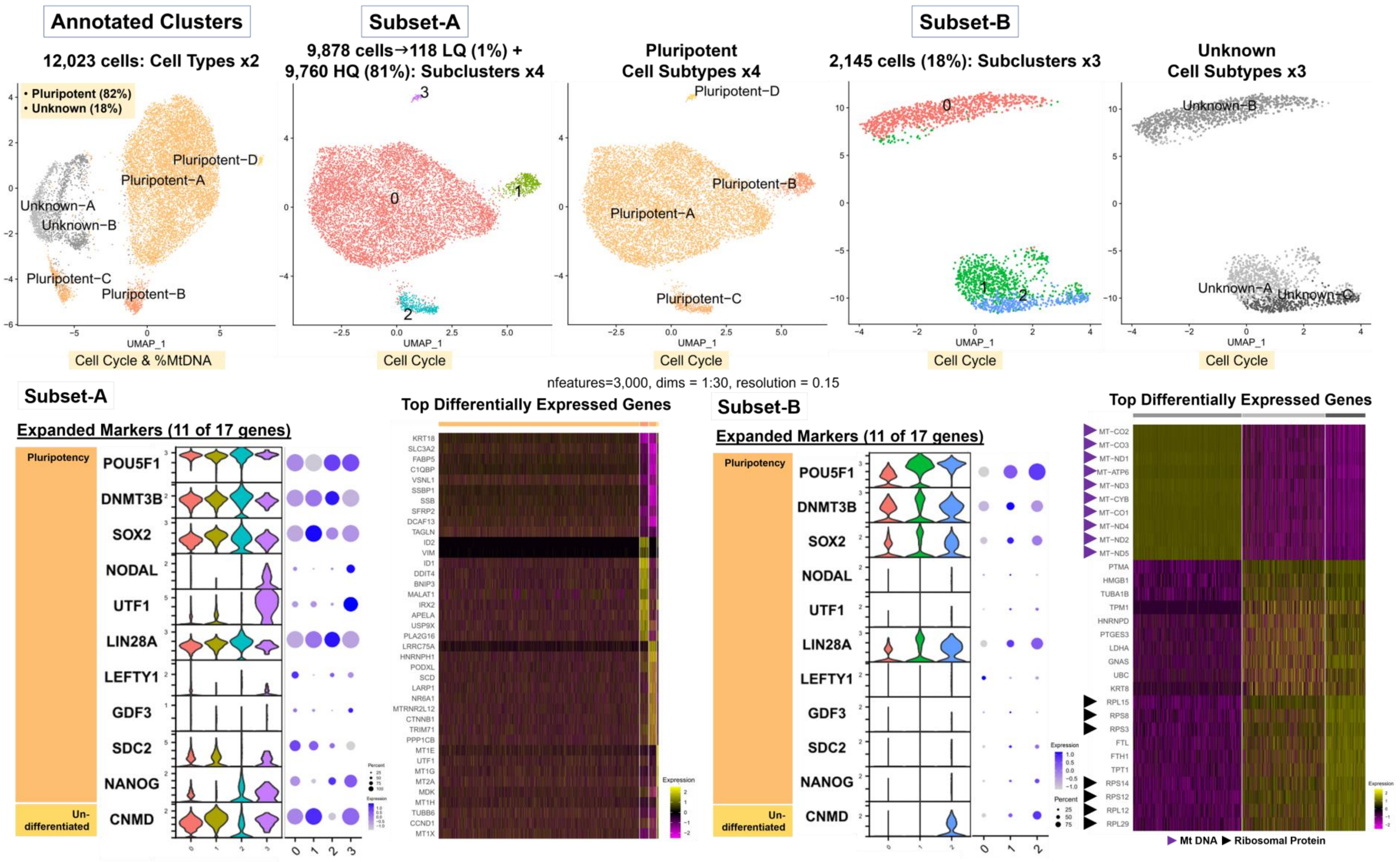

Fig. S6 Subcluster

Patient	Day 9B	Single Sample Data *Subsets*

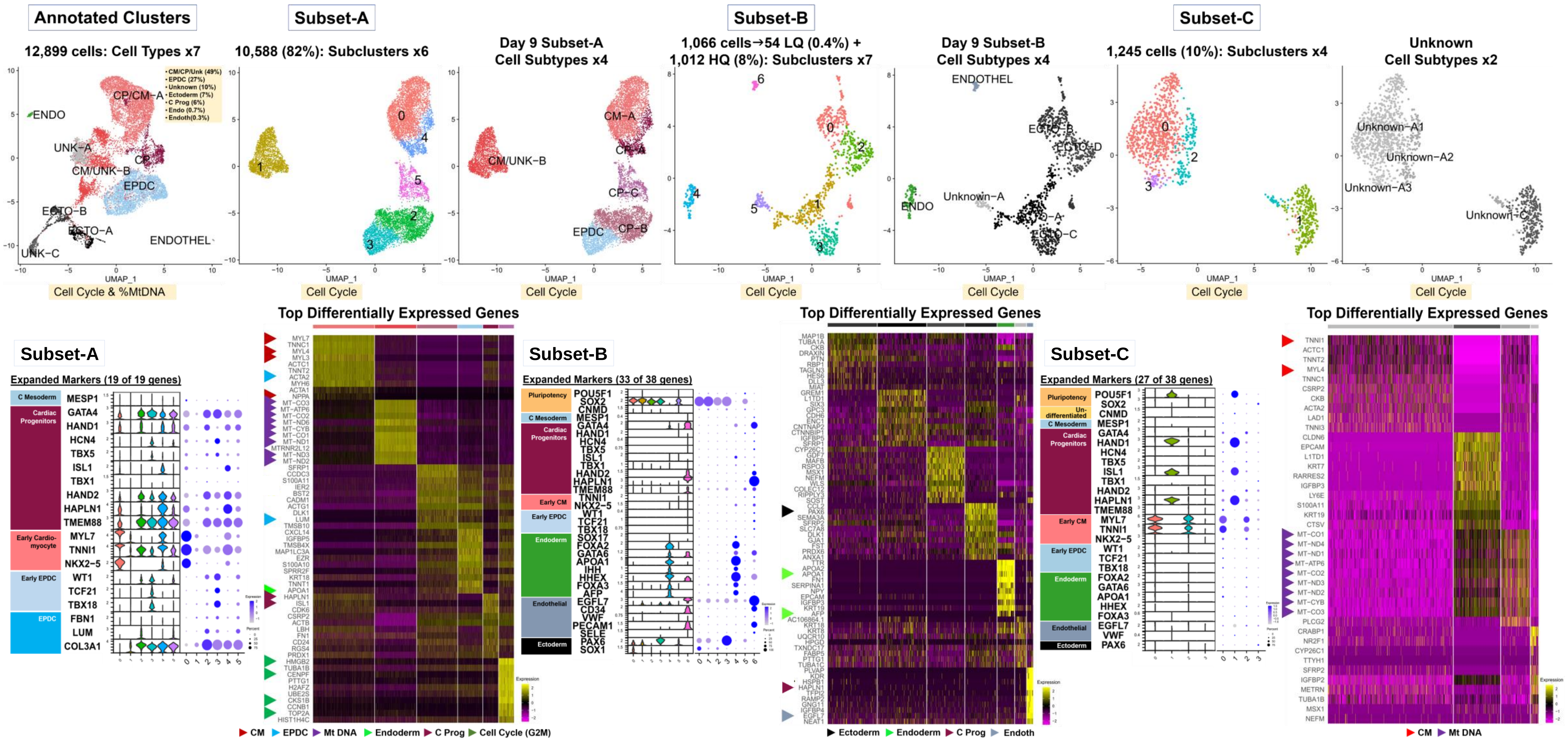

Fig. S6 Subcluster

Patient | Day 16 | Single Sample Data *Subsets*

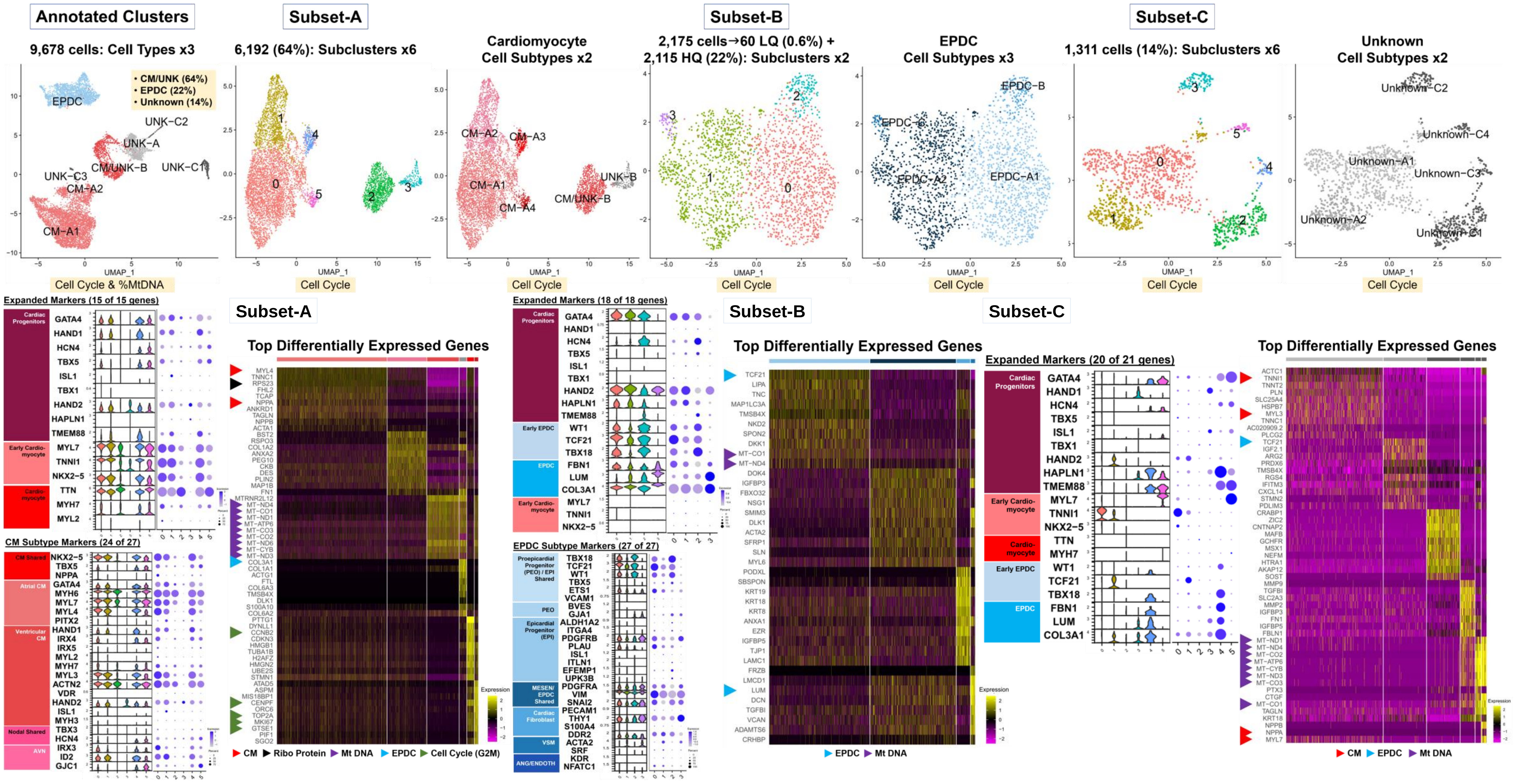

Fig. S6 Subcluster

Patient	Day 19	Single Sample Data *Subsets*

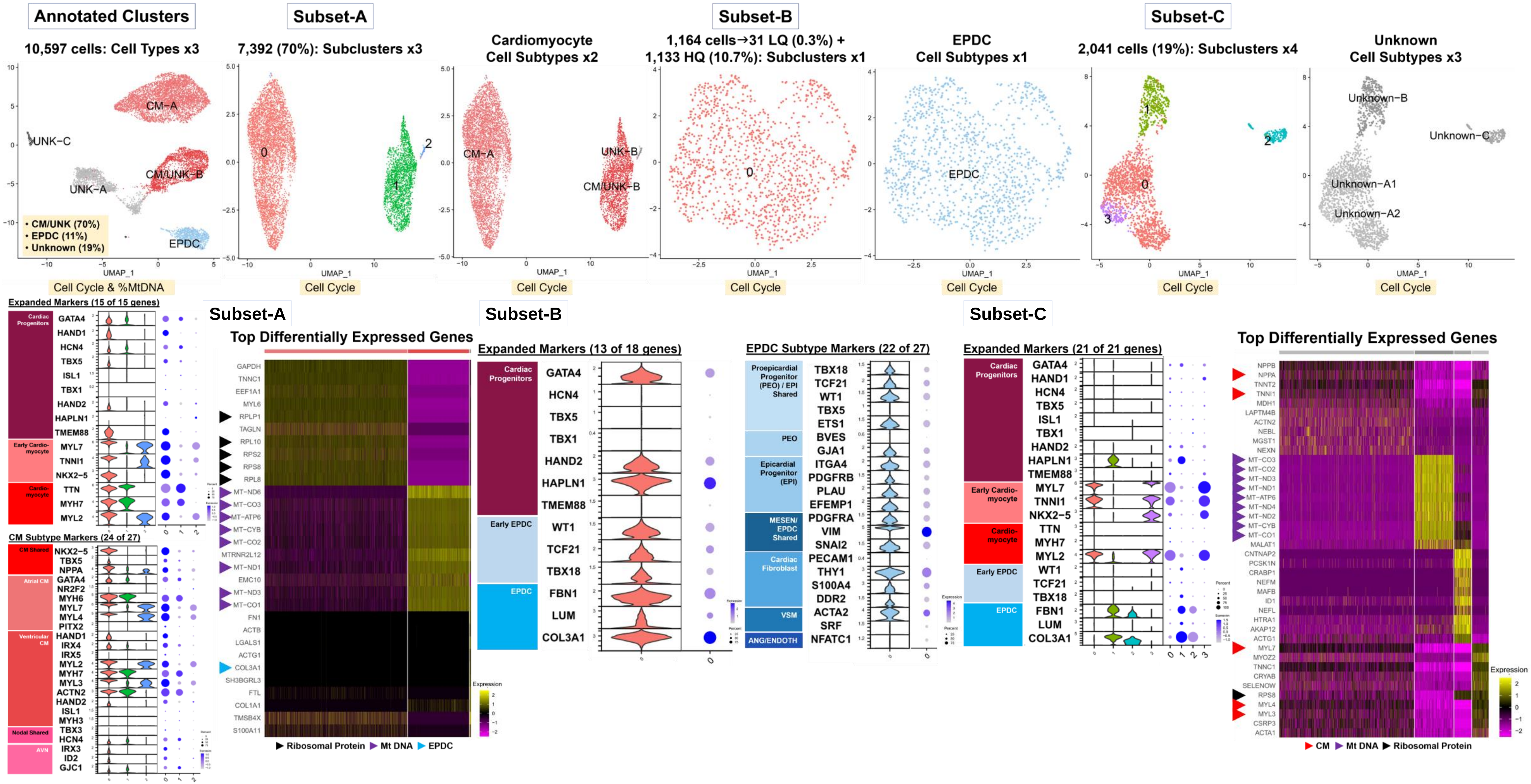

Fig. S7 Combo

Workflow Step-III: Data Combining & Comparative Analyses

A. Summary: Combined Data for Paired Sample Data

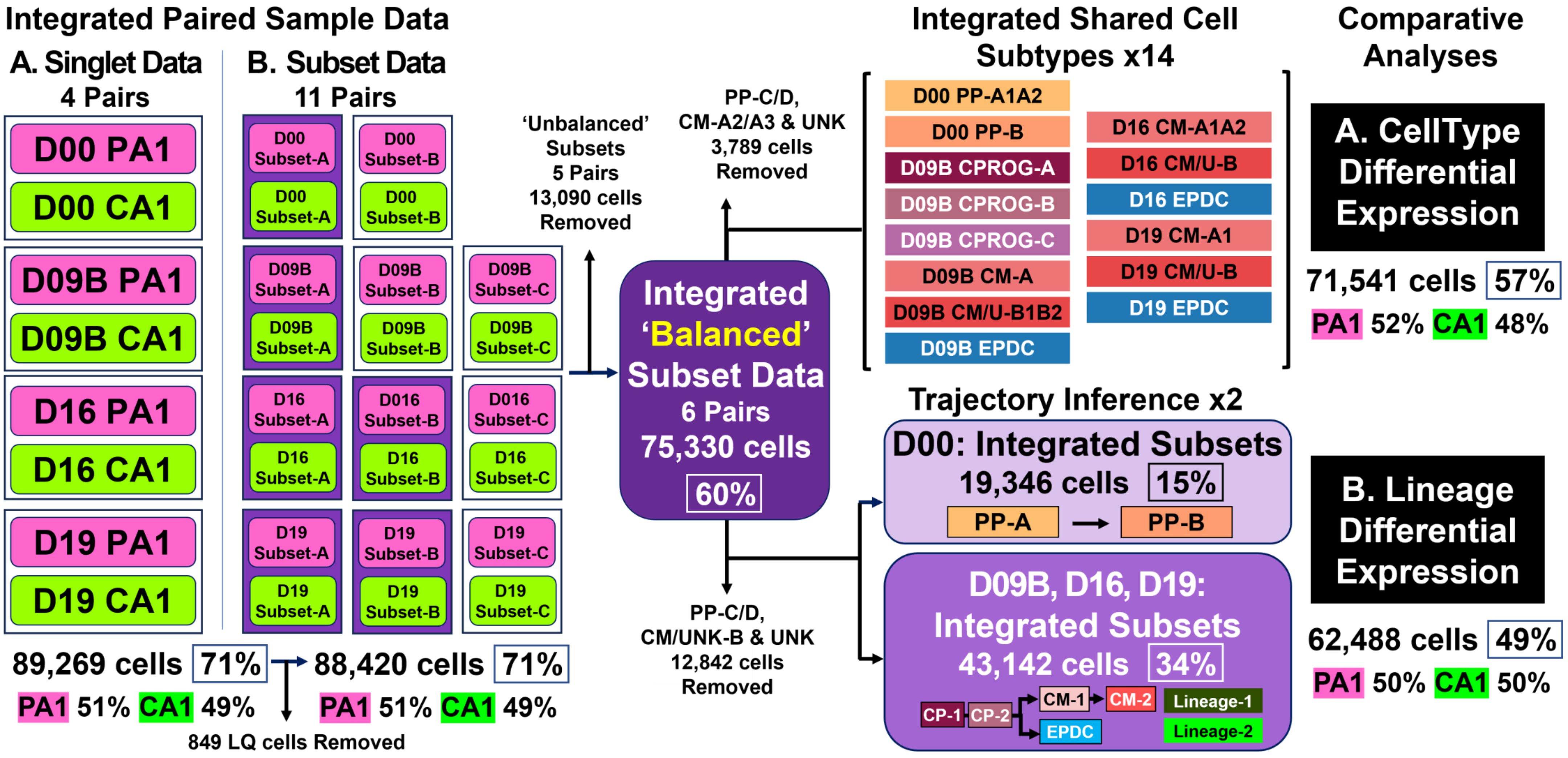

Fig. S7 Combo

B. Summary: Combined Data for ‘Balanced’ Paired Subsets (n=6 Prs: 75,330 Total Cells)

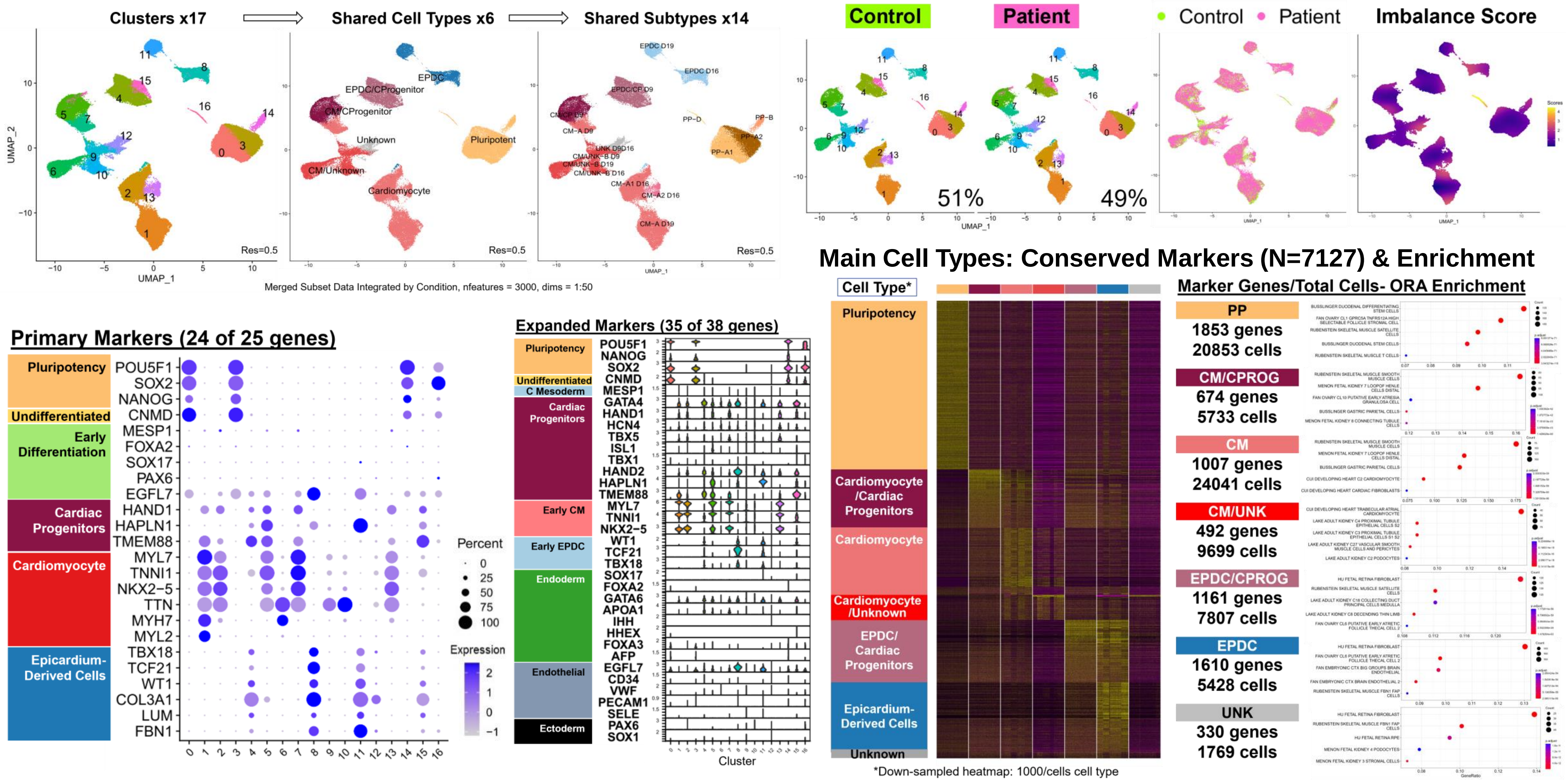

Fig. S8 Pr Cluster

Workflow Step-III: Paired Sample Data Results

Individual Analyses of Combined Singlet Data for Shared Cell Types

A. Summary: Merged vs. Integrated Singlet Data- Clusters and Imbalance

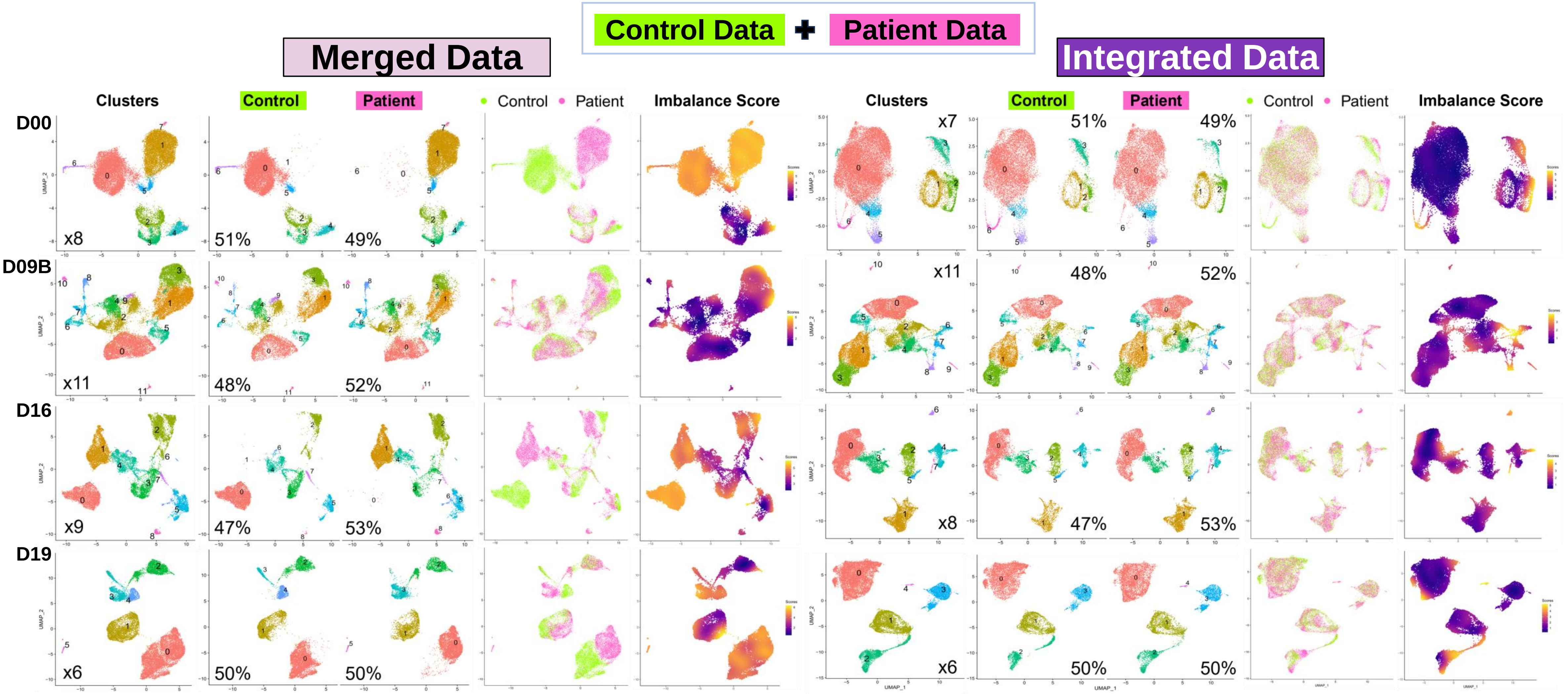

Fig. S8 Pr Cluster

B. Summary: Cell Annotation of Integrated Singlet Data (n=4 Prs: 89,269 Total Cells)

Control Data + Patient Data → Integrated Data

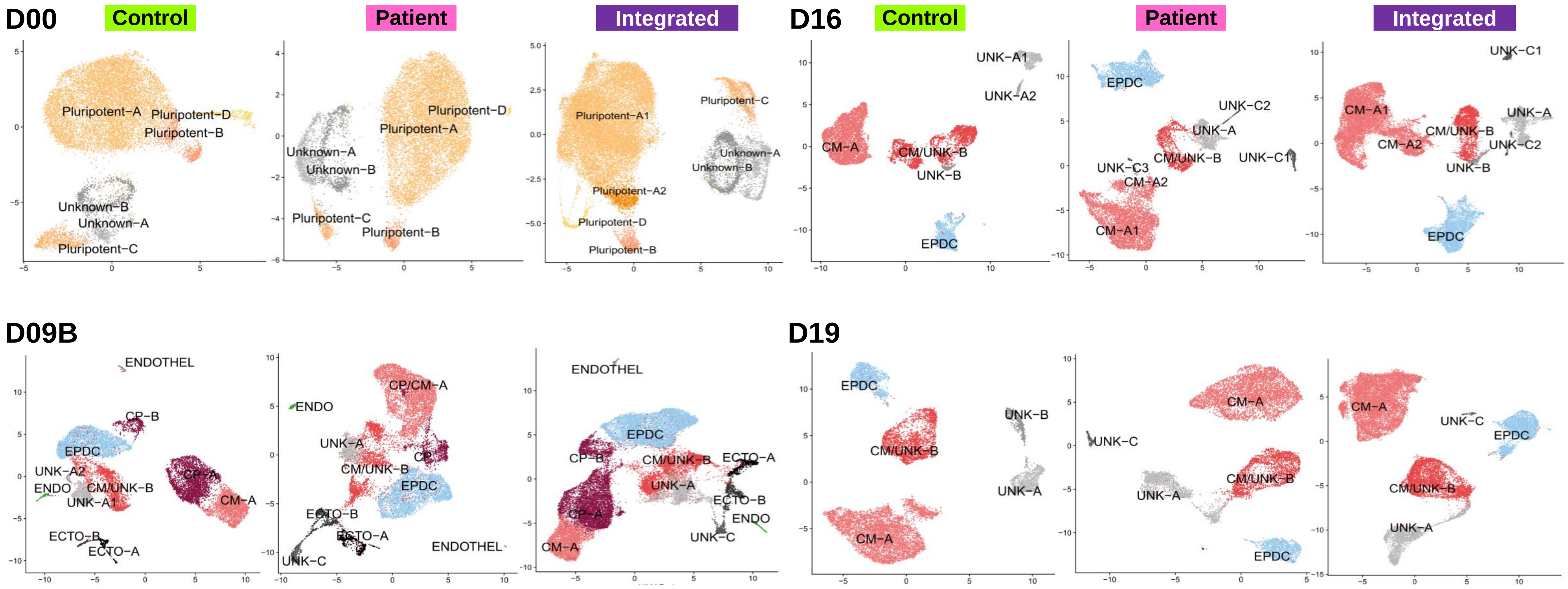

Fig. S8 Pr Cluster

C. Paired Sample Data Analyses: Integrated Singlet Data- Patient vs. Control (n= 4 Prs)

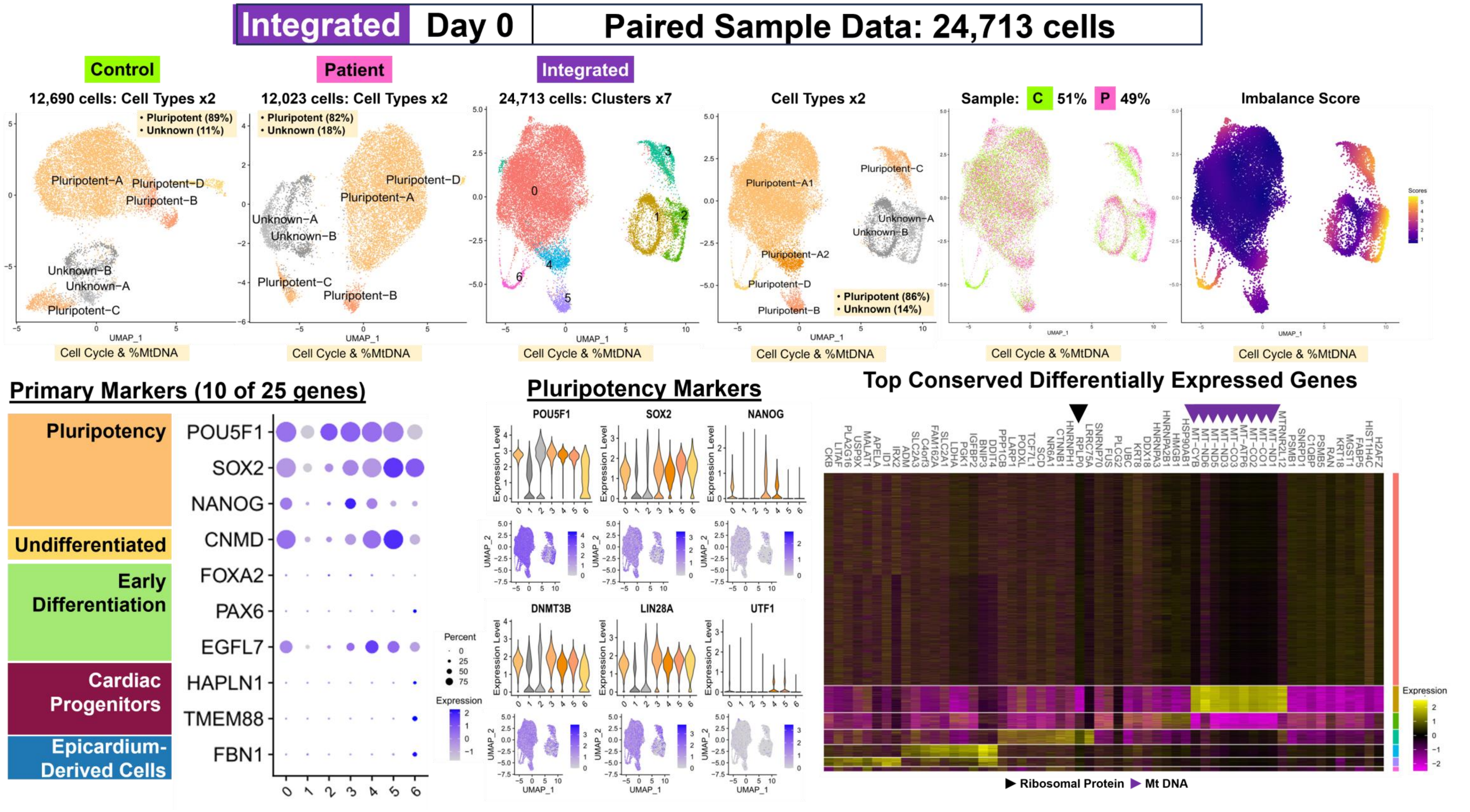

Fig. S8 Pr Cluster

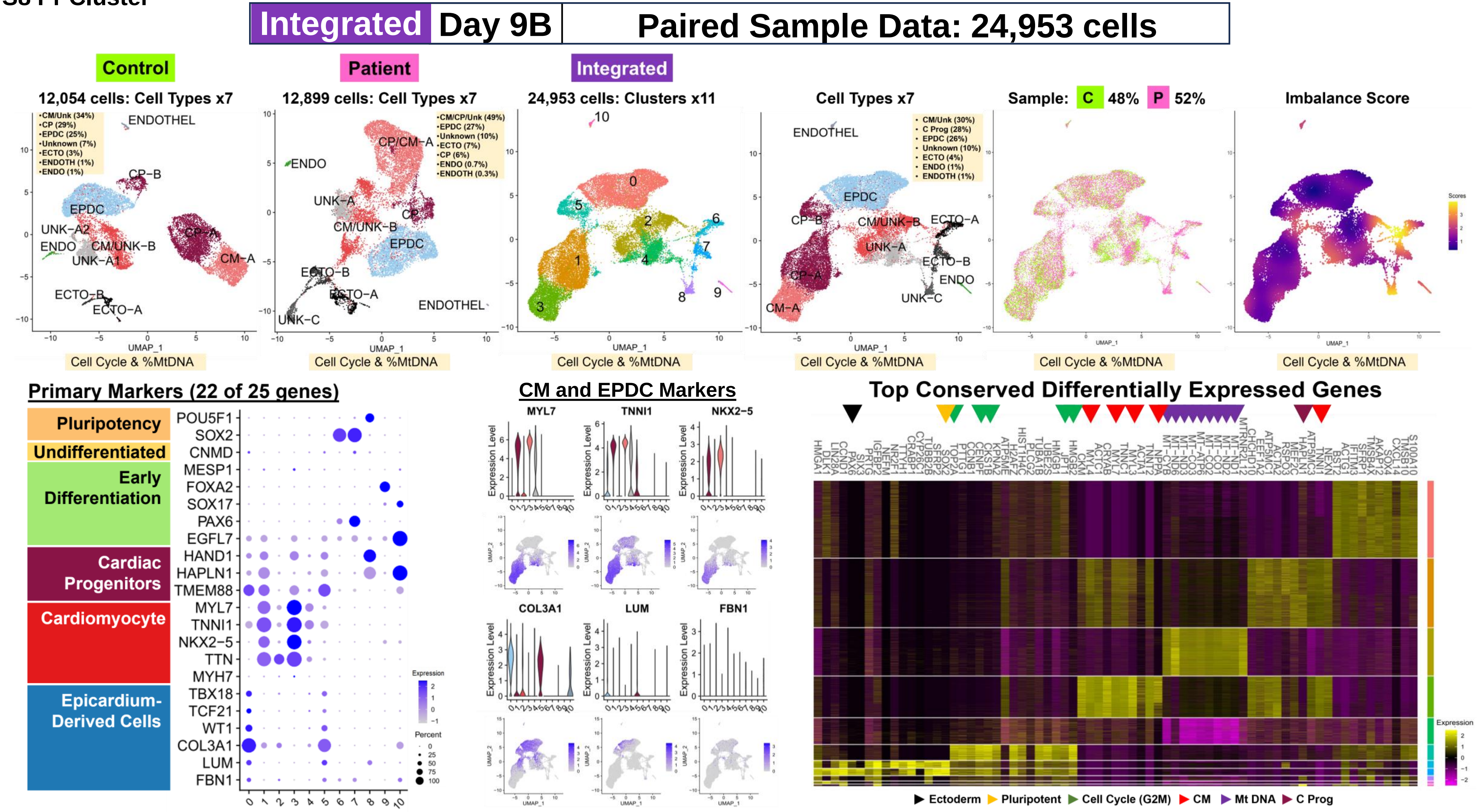

Fig. S8 Pr Cluster

Integrated | Day 16 | Paired Sample Data: 18,390 cells

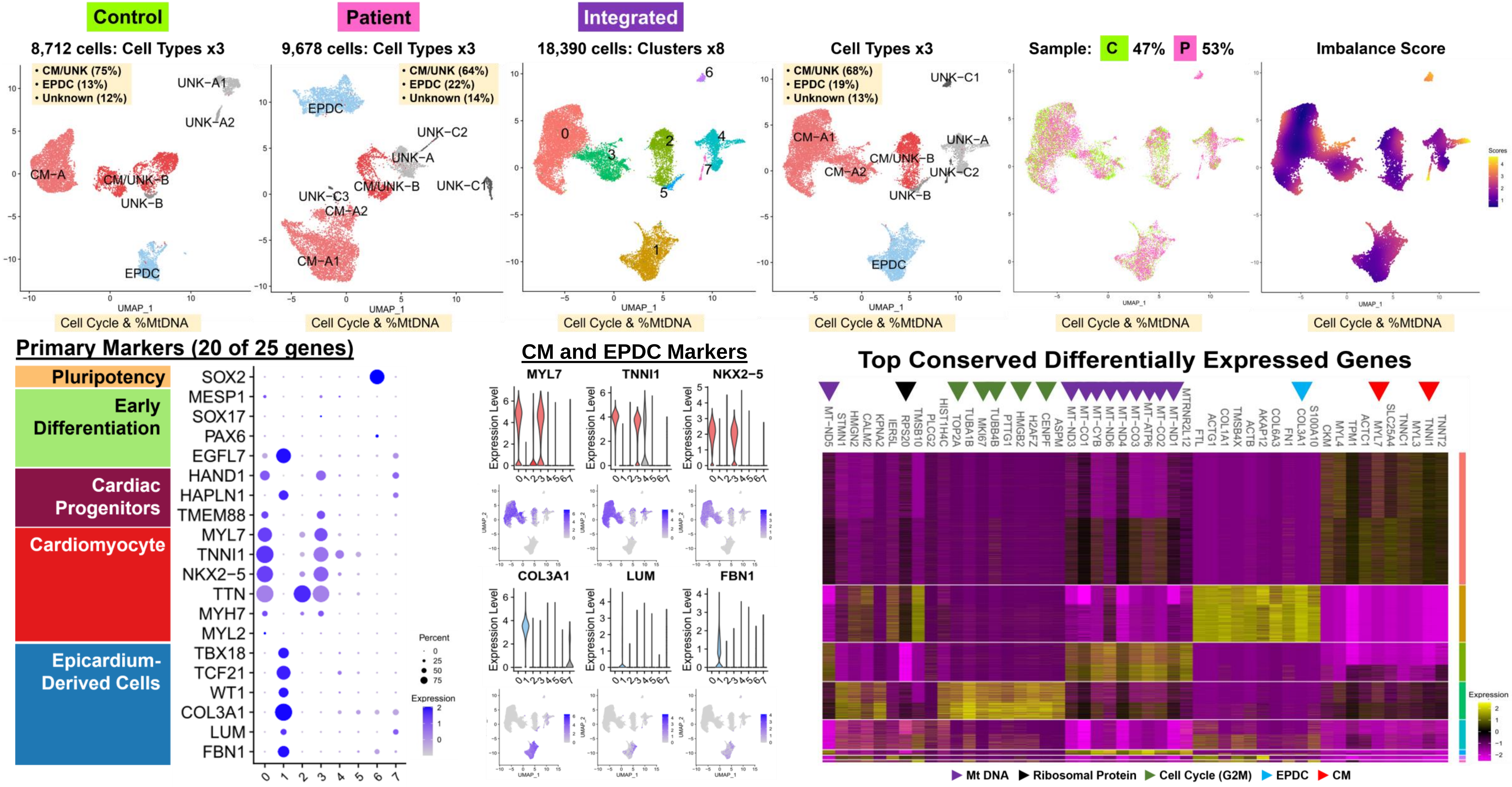

Fig. S8 Pr Cluster

Integrated	Day 19	Paired Sample Data: 21,213 cells

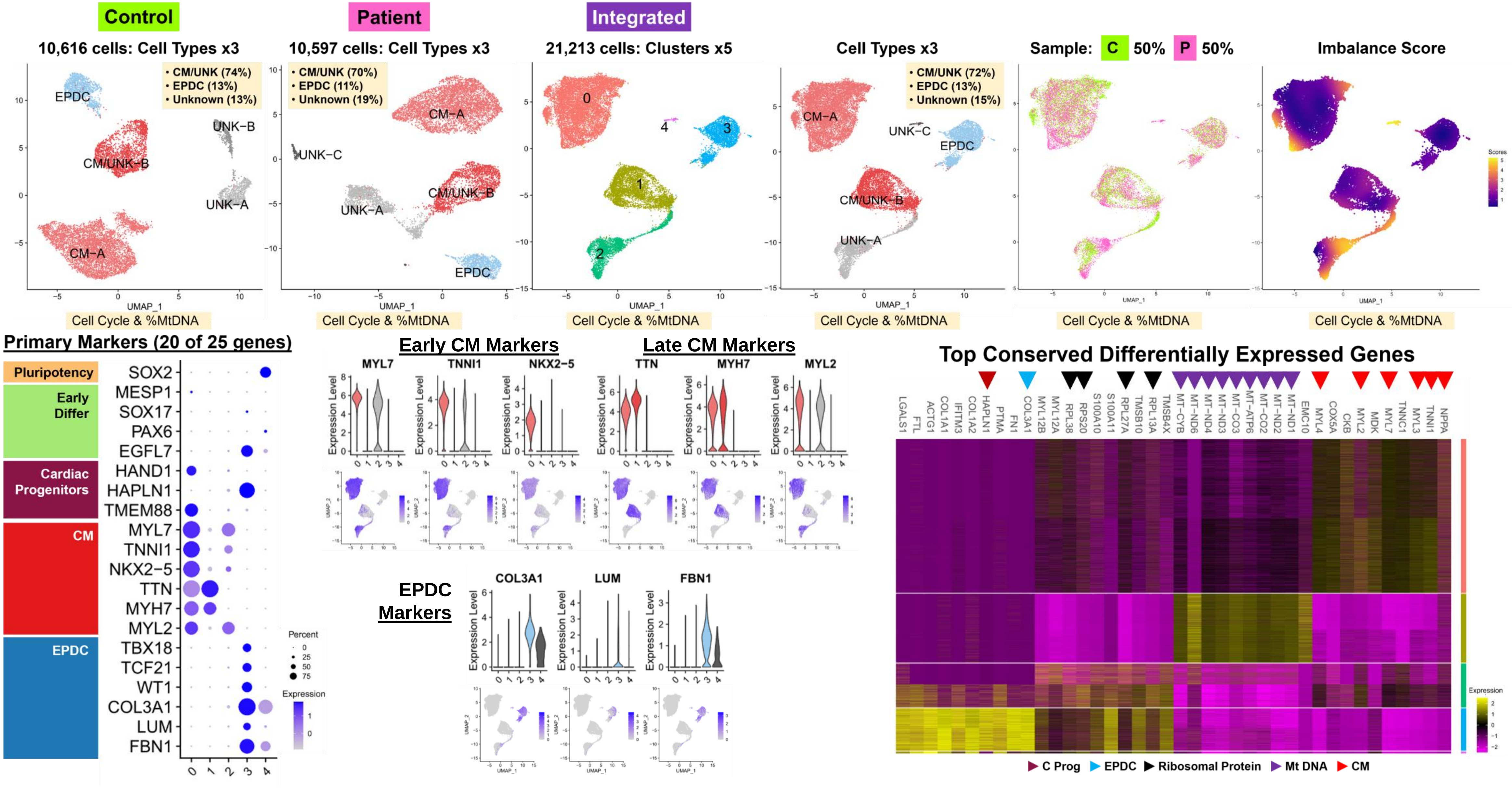

Fig. S9 Pr Subcluster

Workflow Step-III: Paired Sample Data Results

Individual Subcluster Analyses of Combined Subset Data for Possible Shared Subtypes

A. Summary: Merged vs. Integrated Subset Data- Clusters and Imbalance

Control *Subset Data* ✚ Patient *Subset Data*

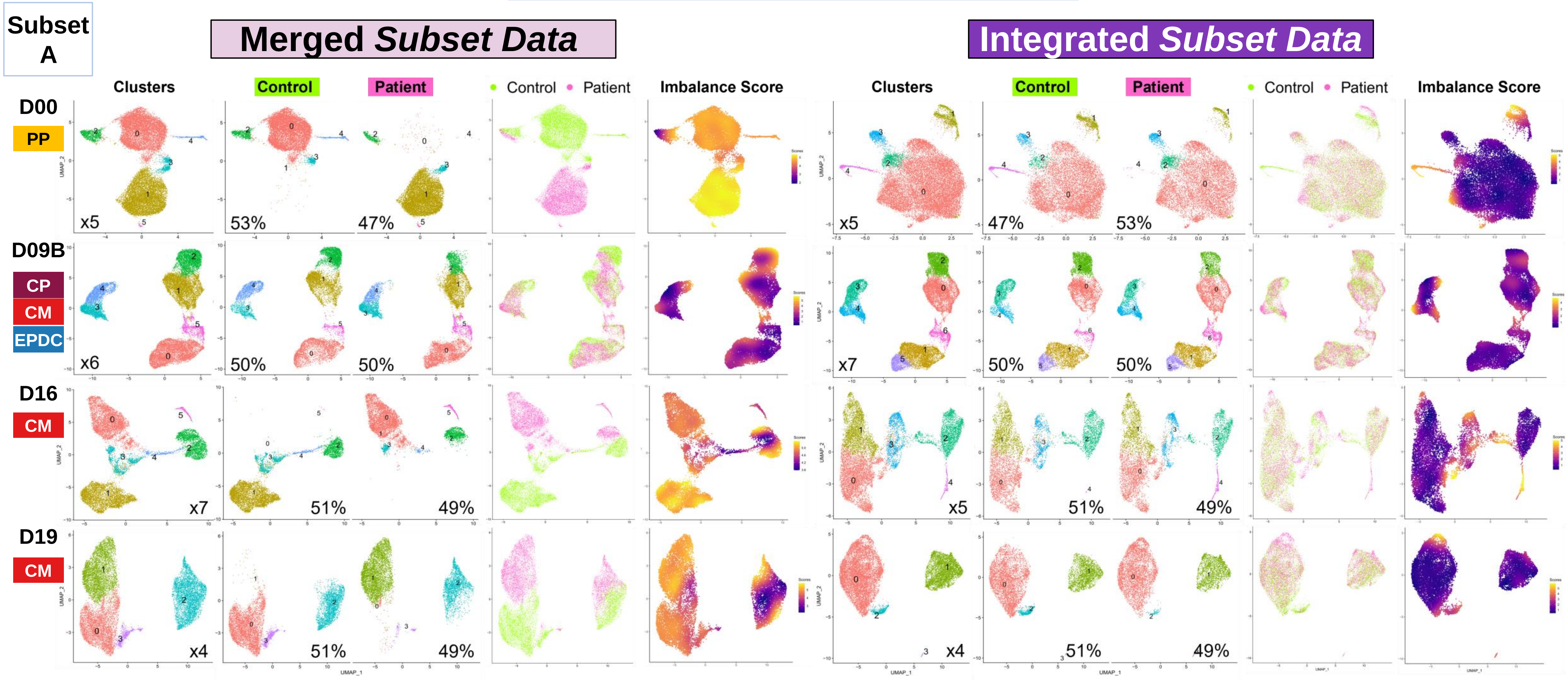

Fig. S9 Pr Subcluster

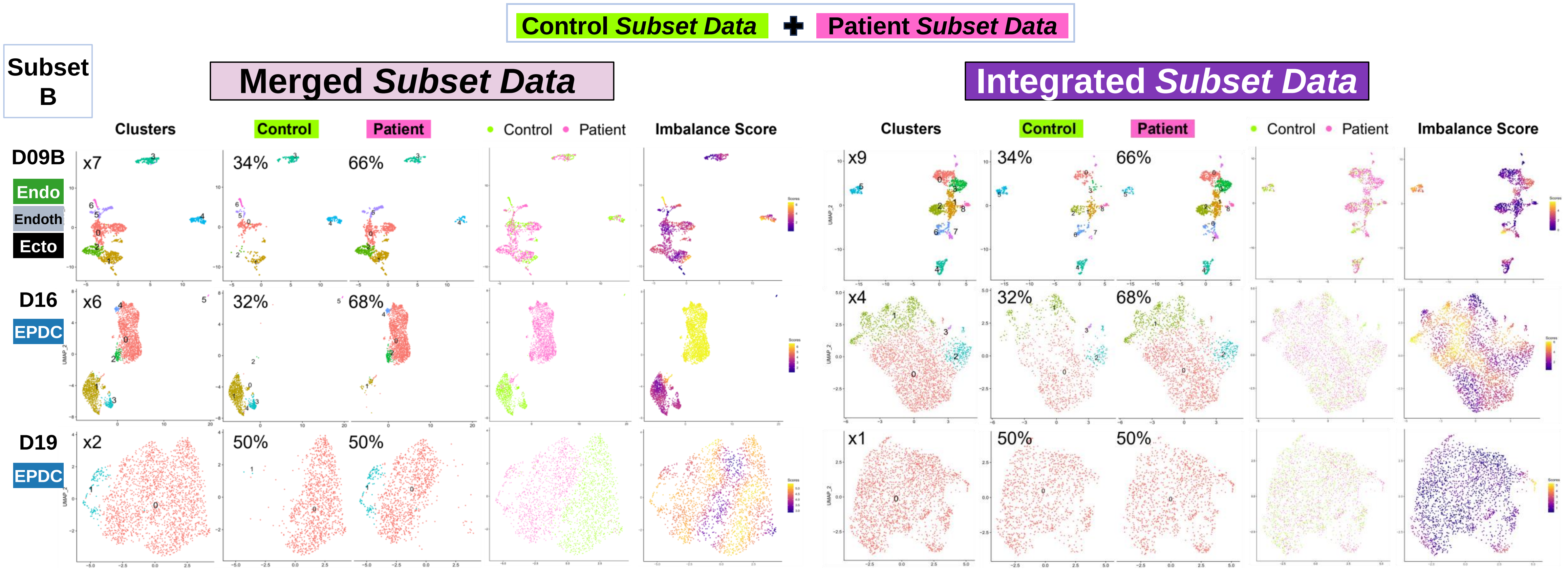

Fig. S9 Pr Subcluster

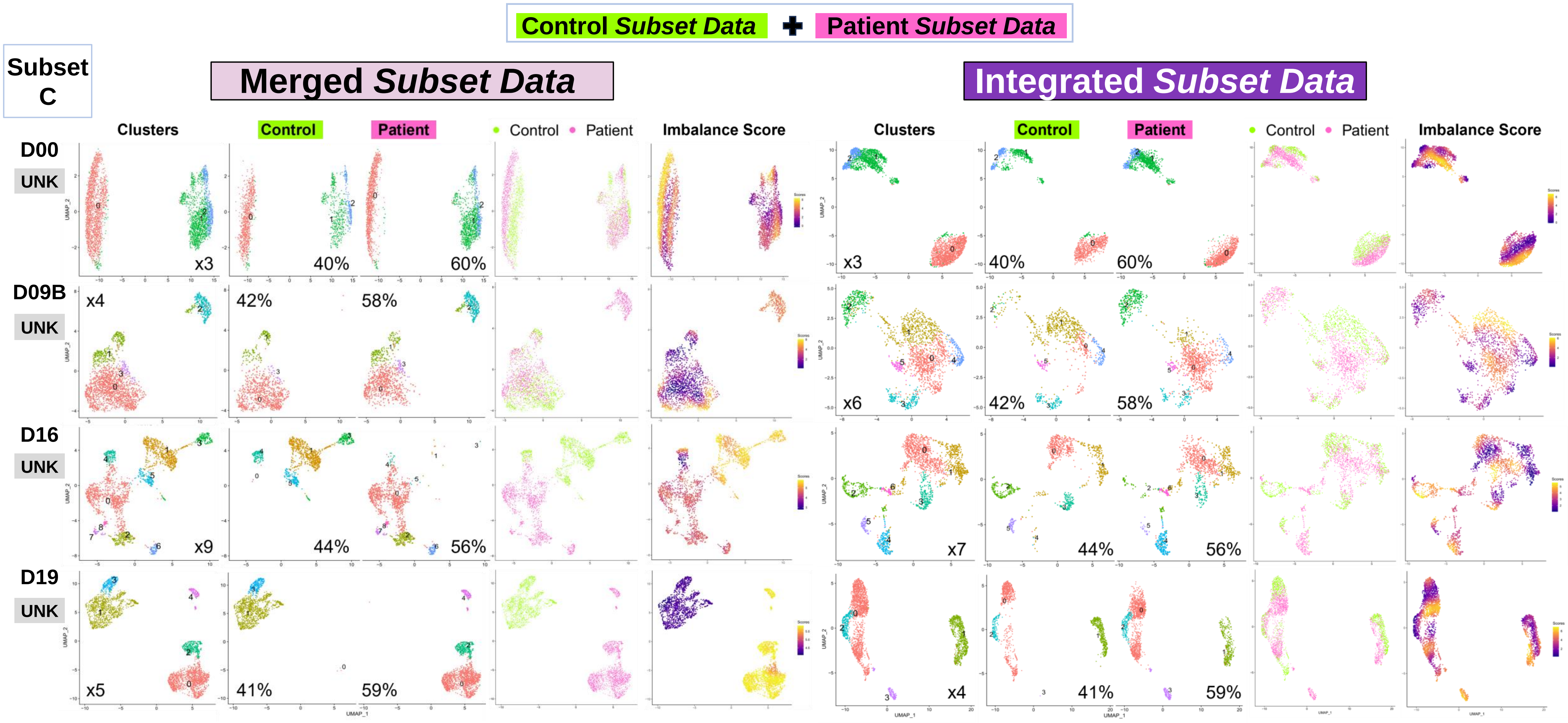

Fig. S9 Pr Subcluster

B. Summary: Cell Annotation of Integrated Subset Data (n=11 Prs: 88,420 Total Cells)

Control *Subset Data* + Patient *Subset Data* → Integrated *Subset Data*

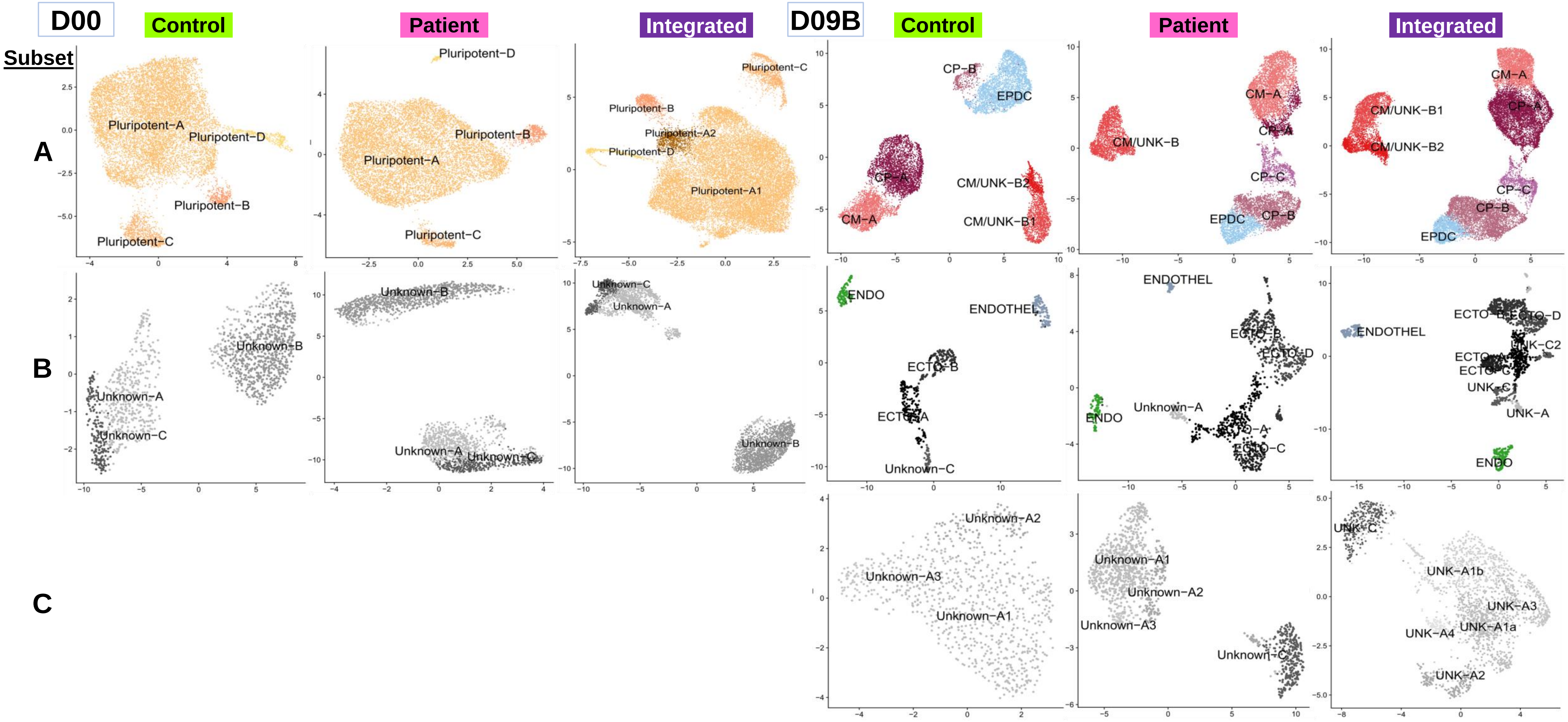

Fig. S9 Pr Subcluster

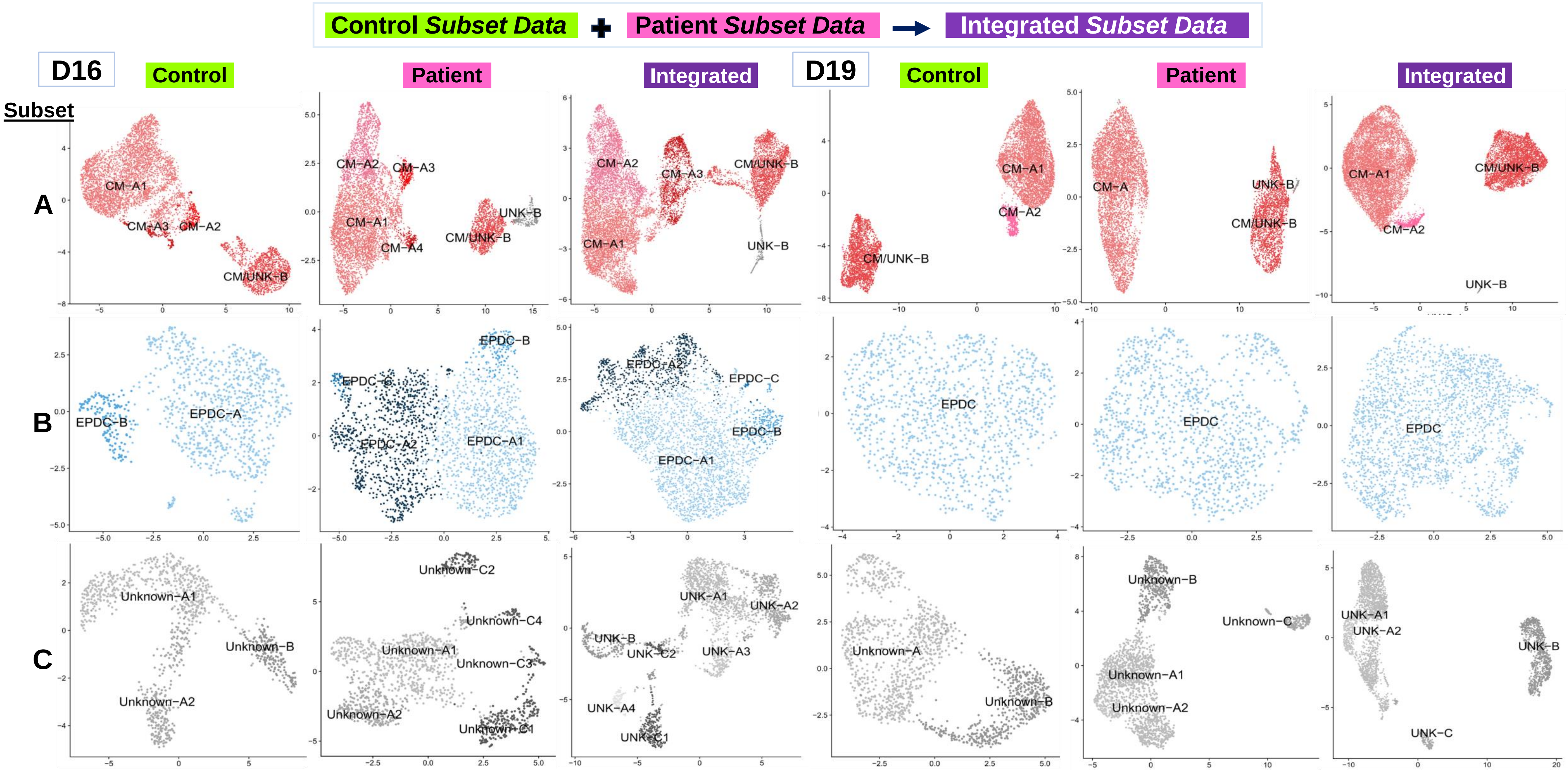

Fig. S9 Pr Subcluster

C. Paired Sample Data Analyses: Integrated Subset Data- Patient vs. Control (n= 11 Prs)

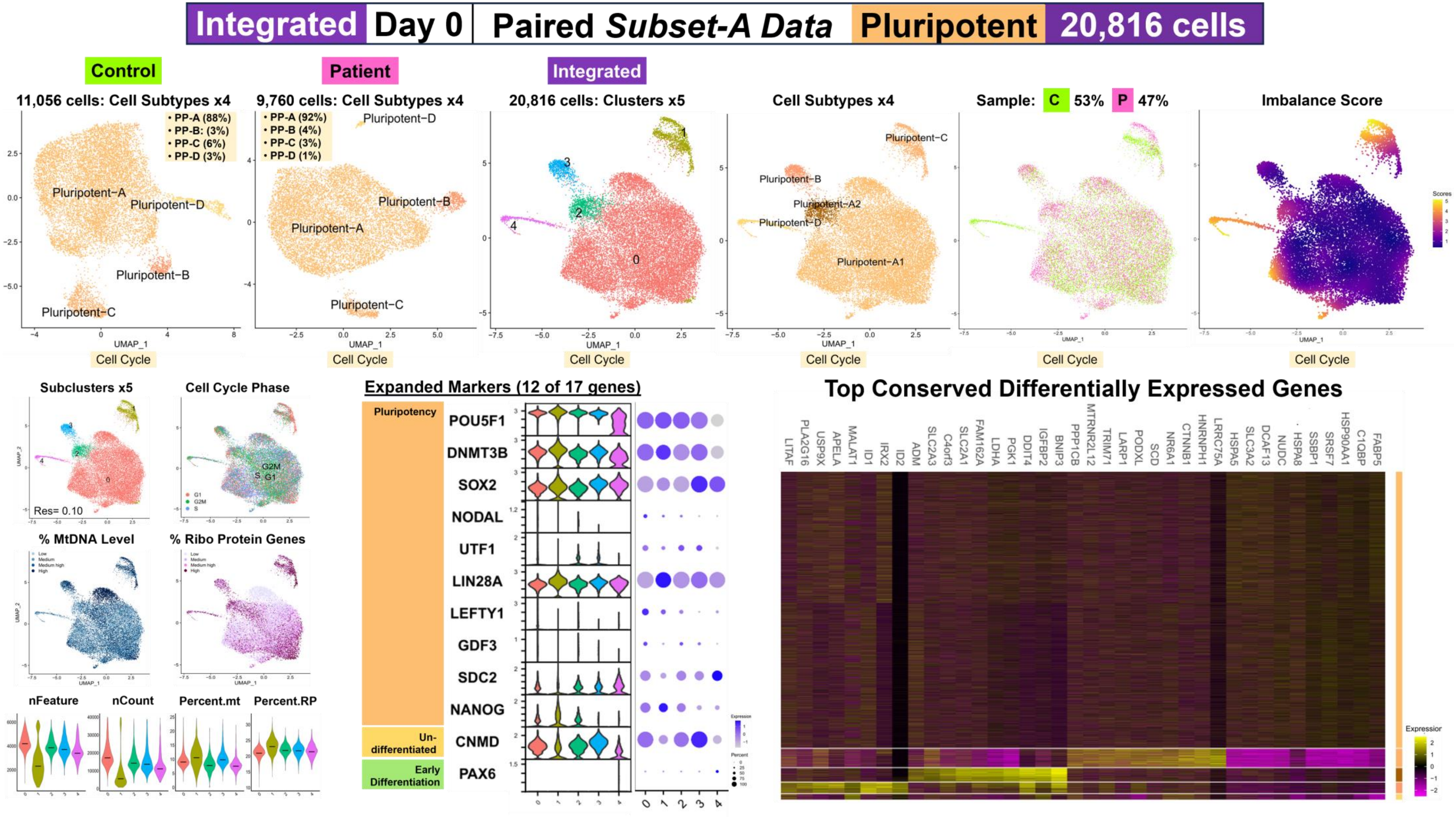

Fig. S9 Pr Subcluster

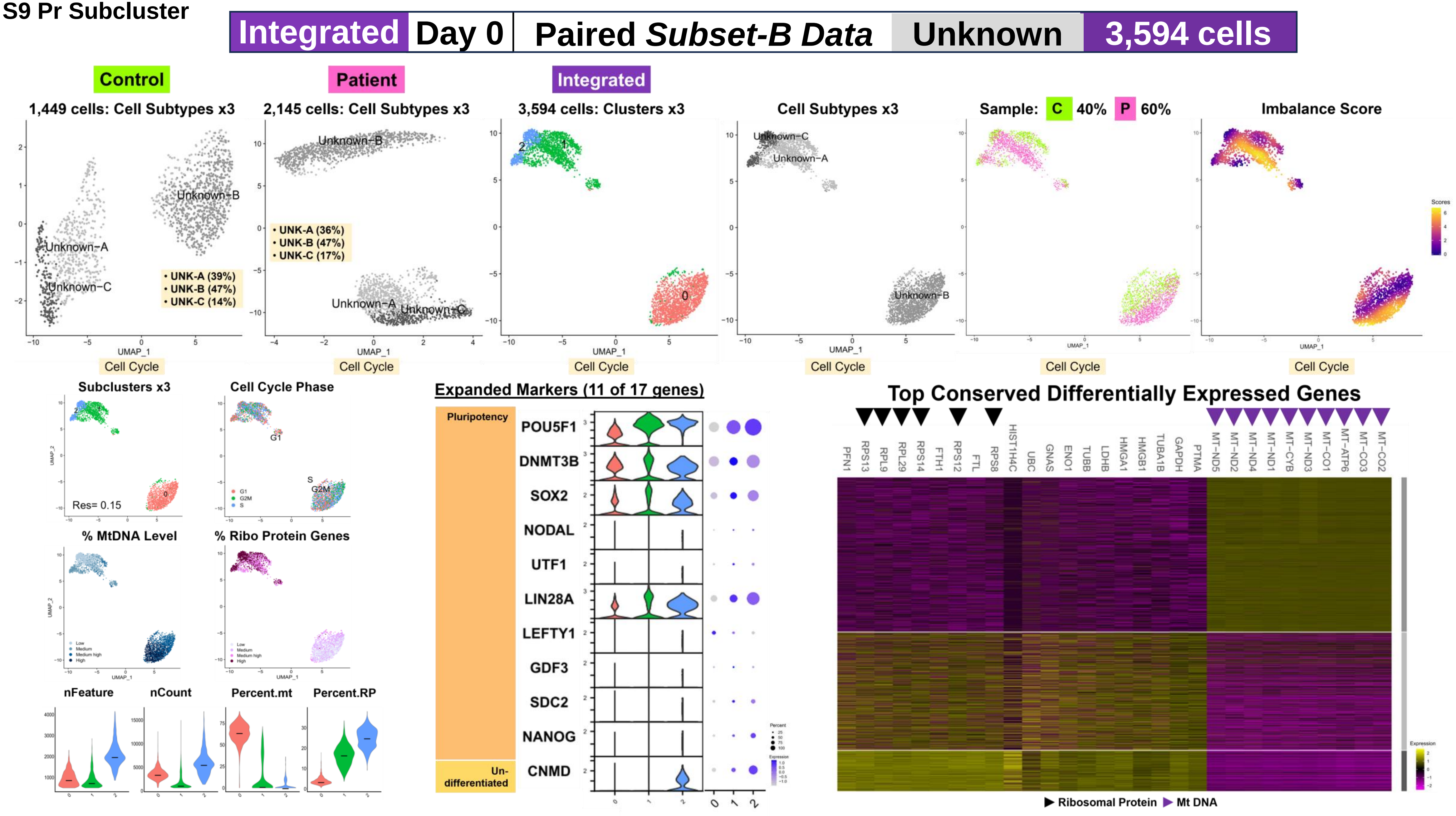

Fig. S9 Pr Subcluster

Integrated | Day 9B | Paired *Subset-A Data* CP-CM-EPDC 21,200 cells

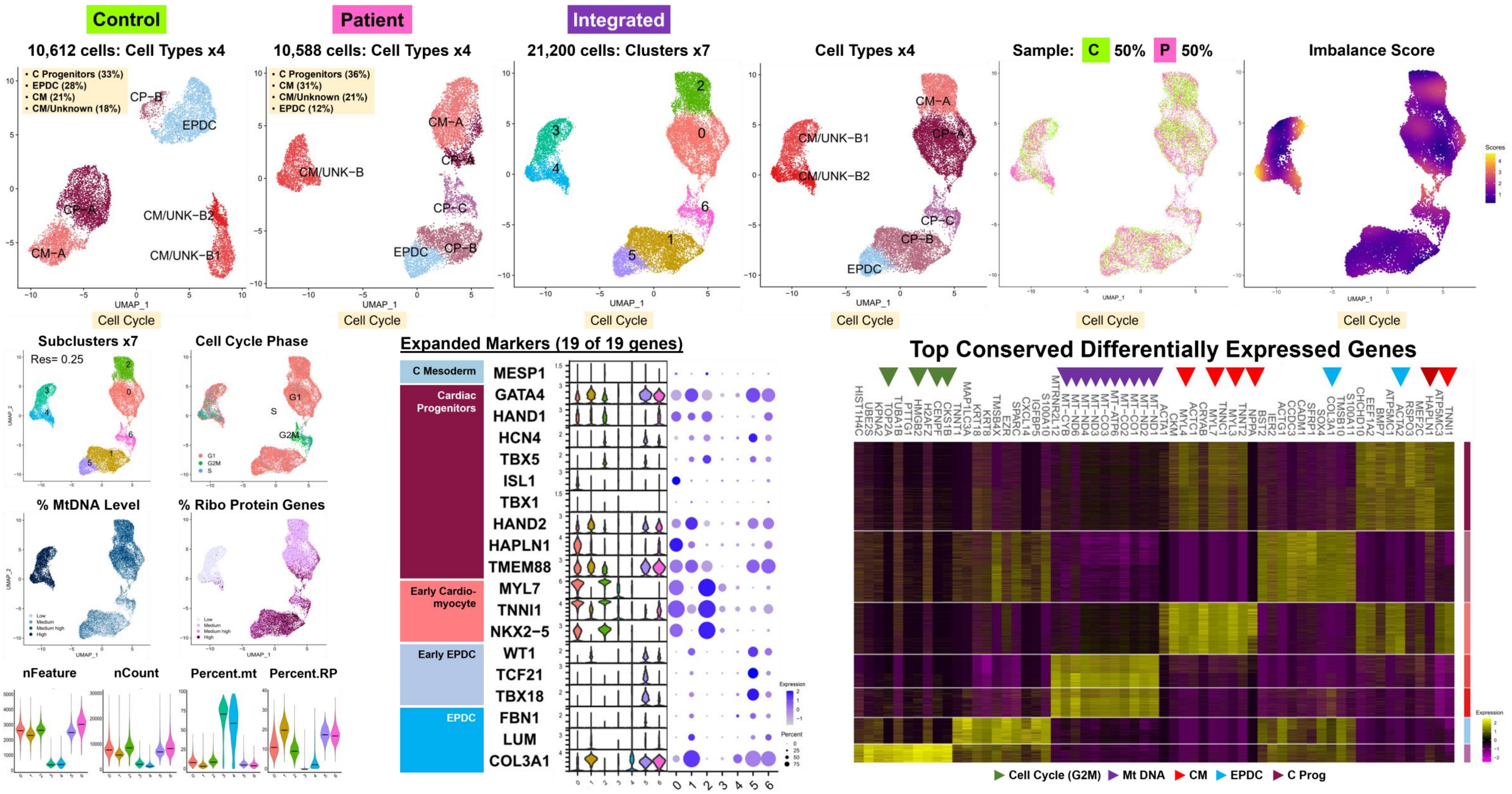

Fig. S9 Pr Subcluster

Integrated | Day 9B | Paired *Subset-B Data* | Endo-Ecto-Endoth | 1,531 cells

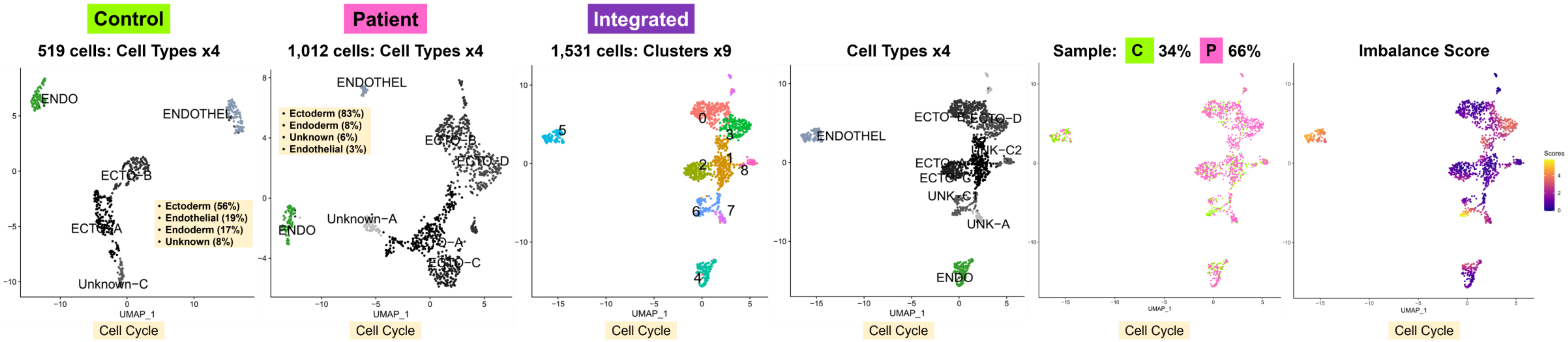

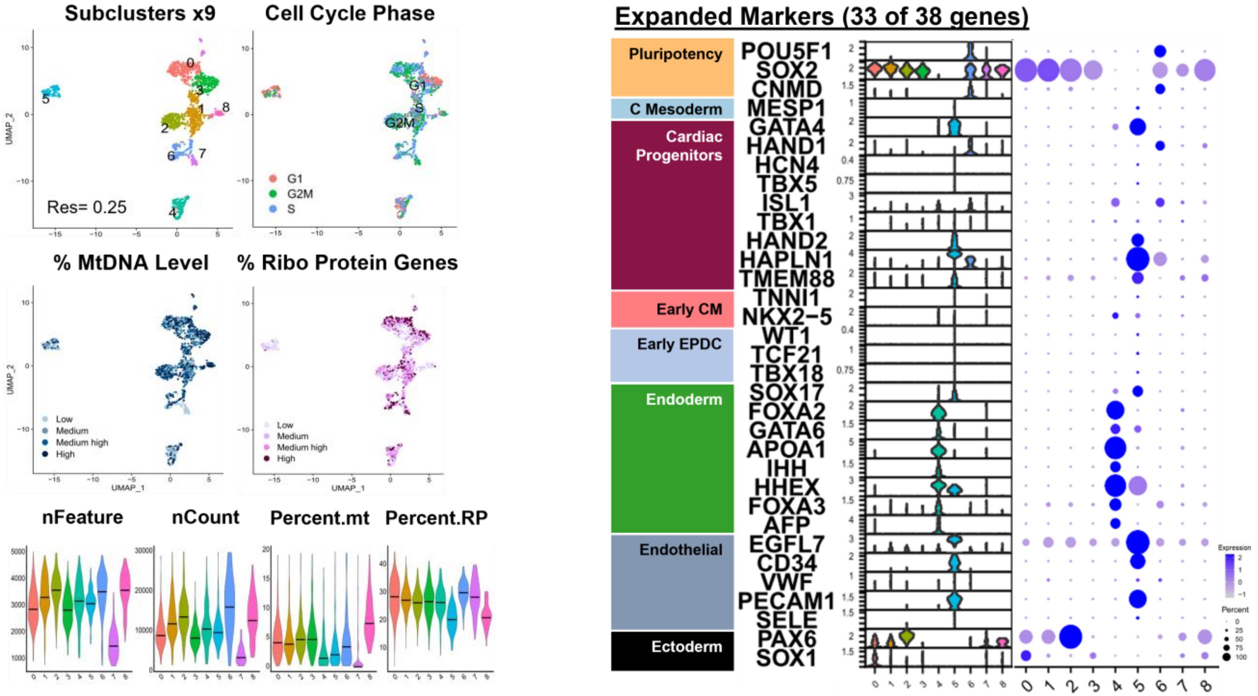

Hmap Conserved DEG:
Not done since <100 cells each for all Control and Patient clusters

Fig. S9 Pr Subcluster

Integrated	Day 9B	Paired *Subset-C Data*	Unknown	2,155 cells

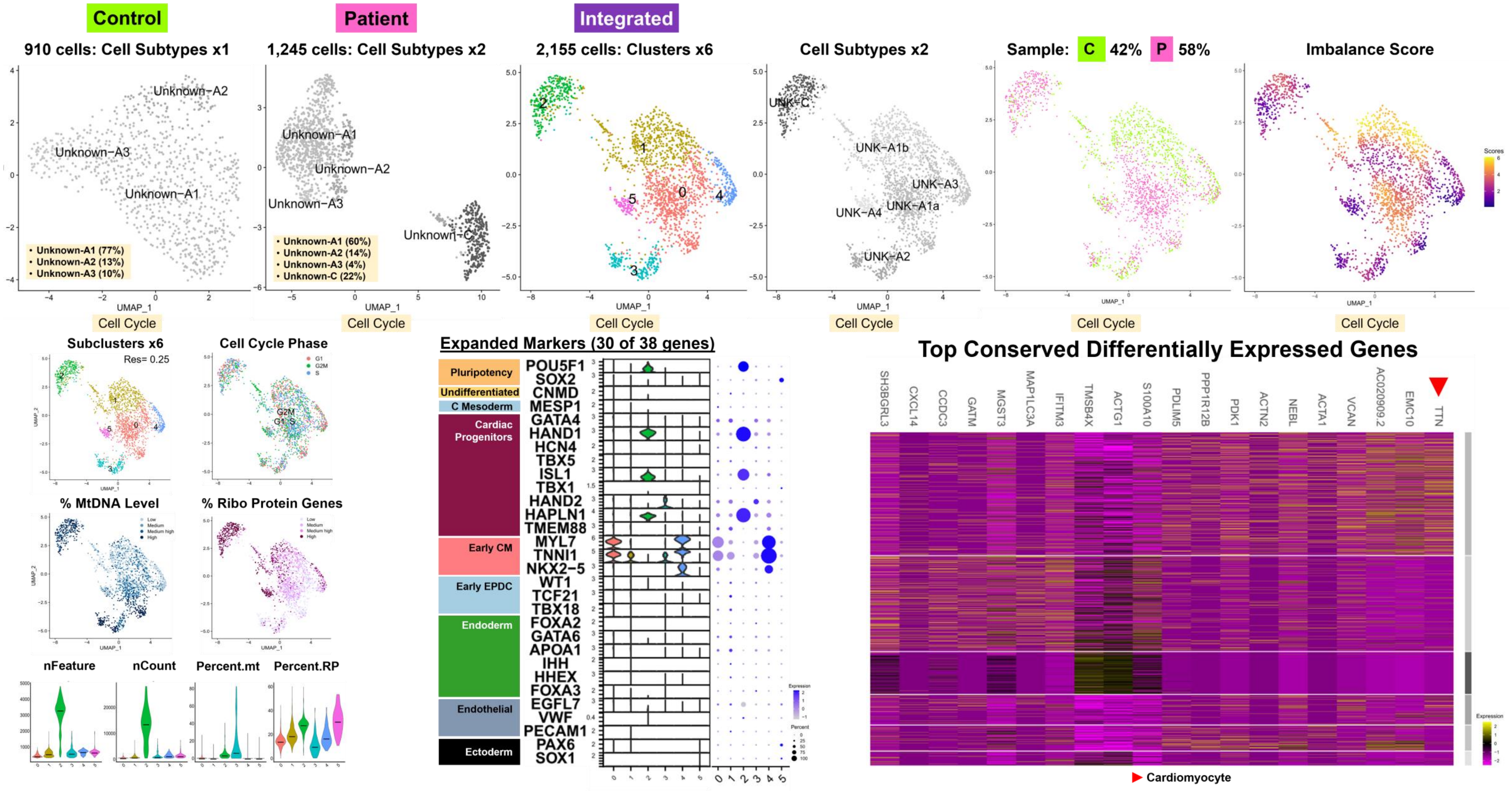

Fig. S9 Pr Subcluster

Integrated	Day 16	Paired *Subset-A Data*	CM	12,700 cells

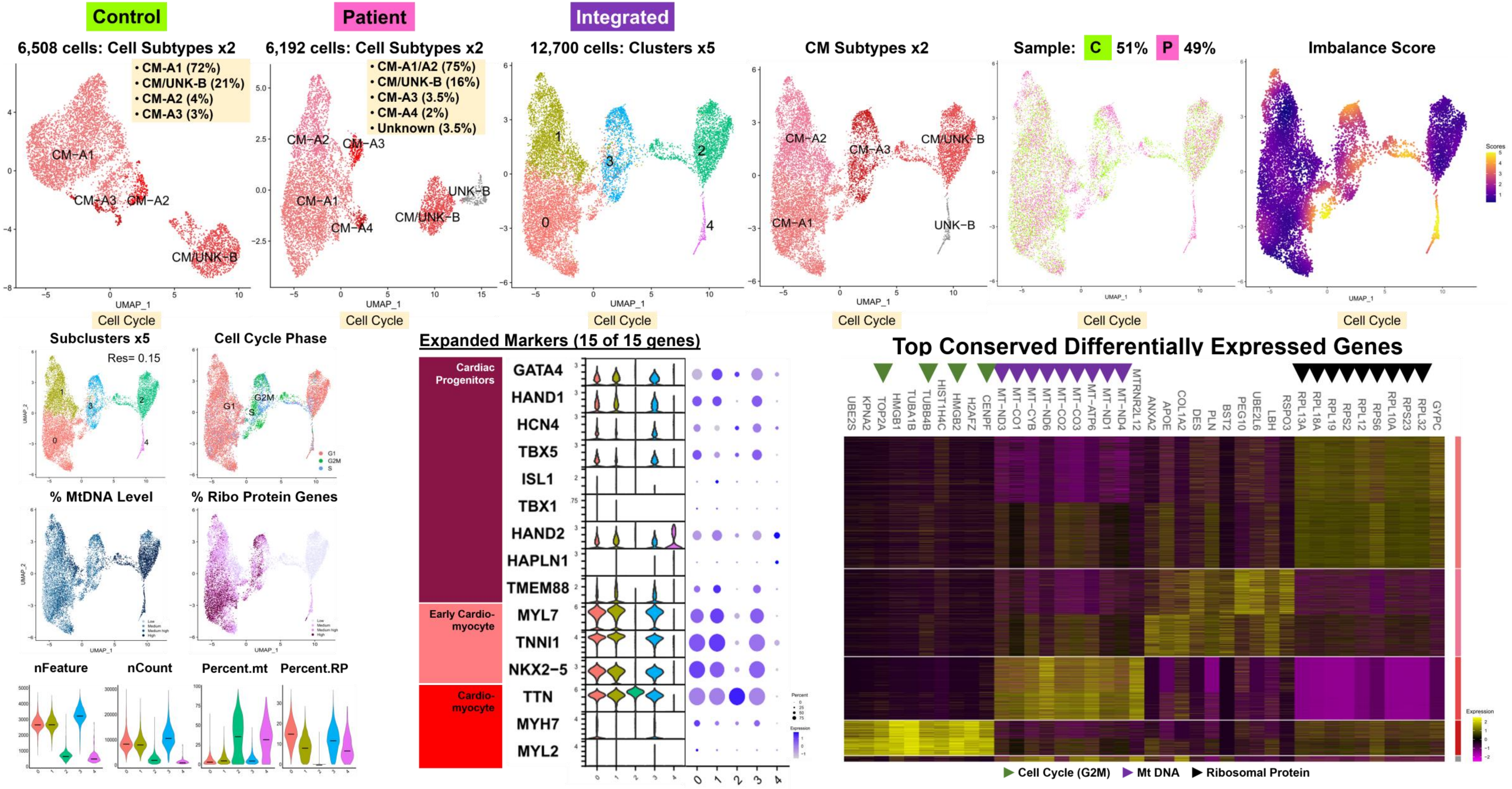

Fig. S9 Pr Subcluster

Integrated	Day 16	Paired *Subset-B Data*	EPDC	3,133 cells

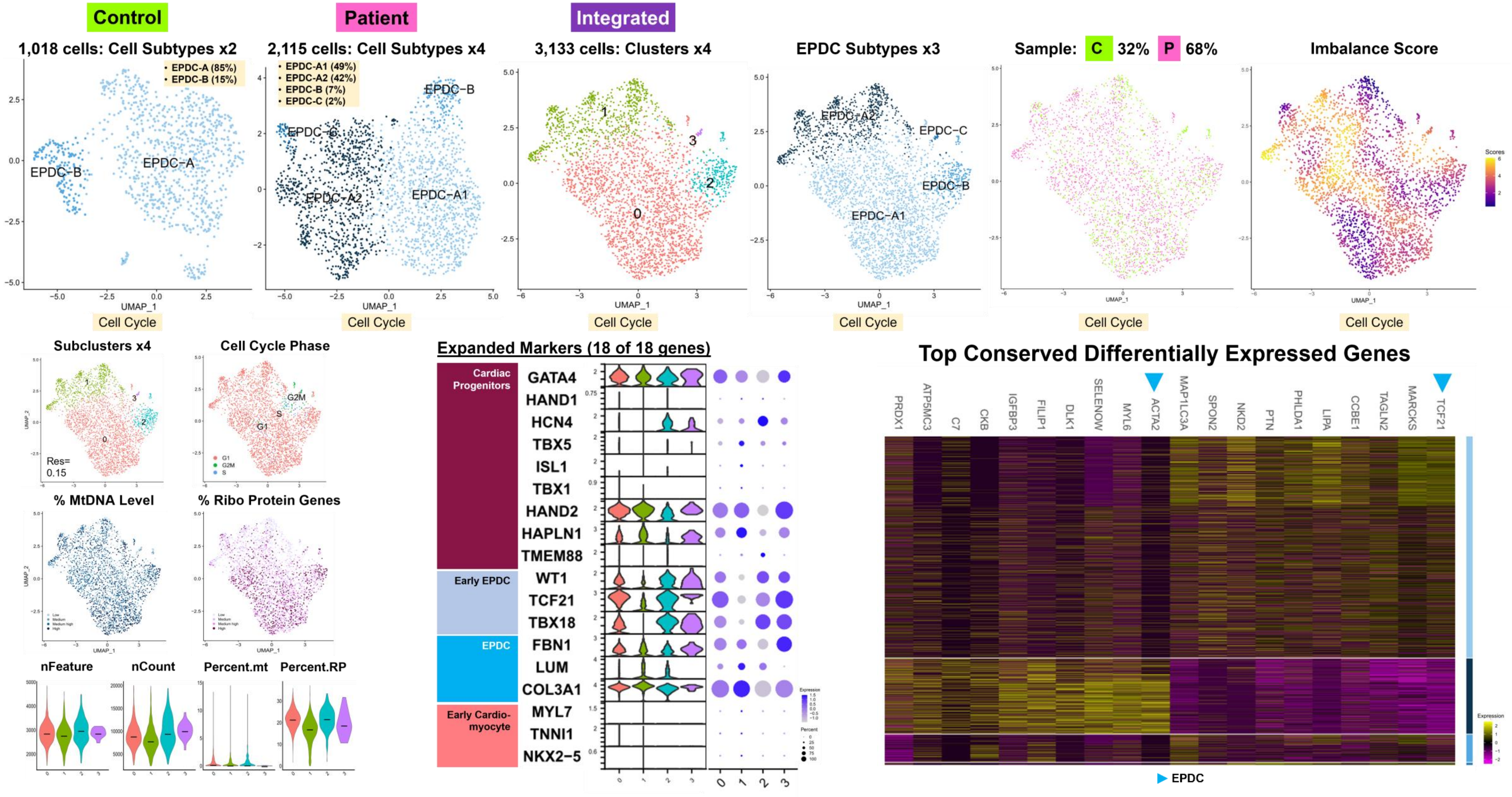

Fig. S9 Pr Subcluster

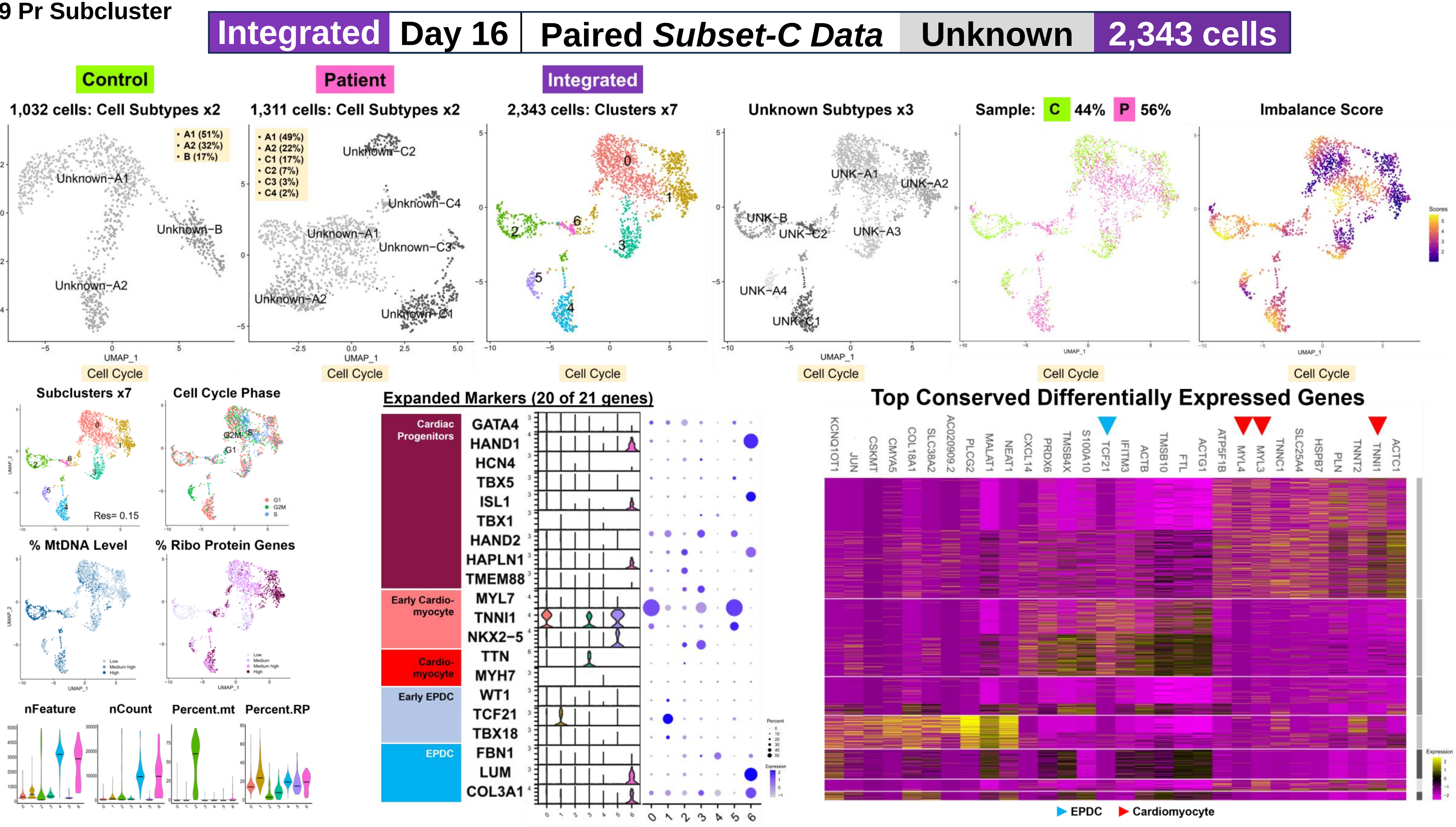

Fig. S9 Pr Subcluster

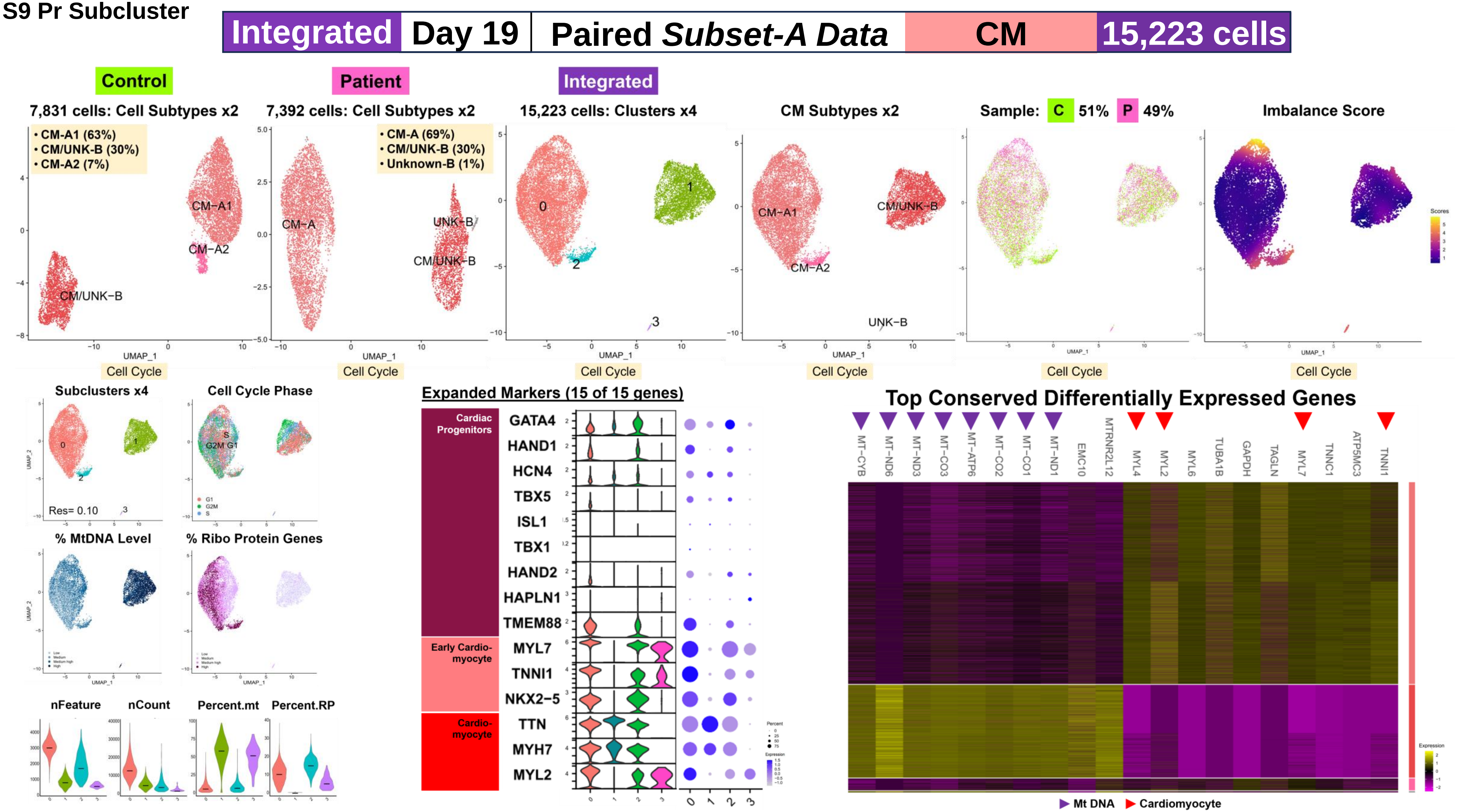

Fig. S9 Pr Subcluster

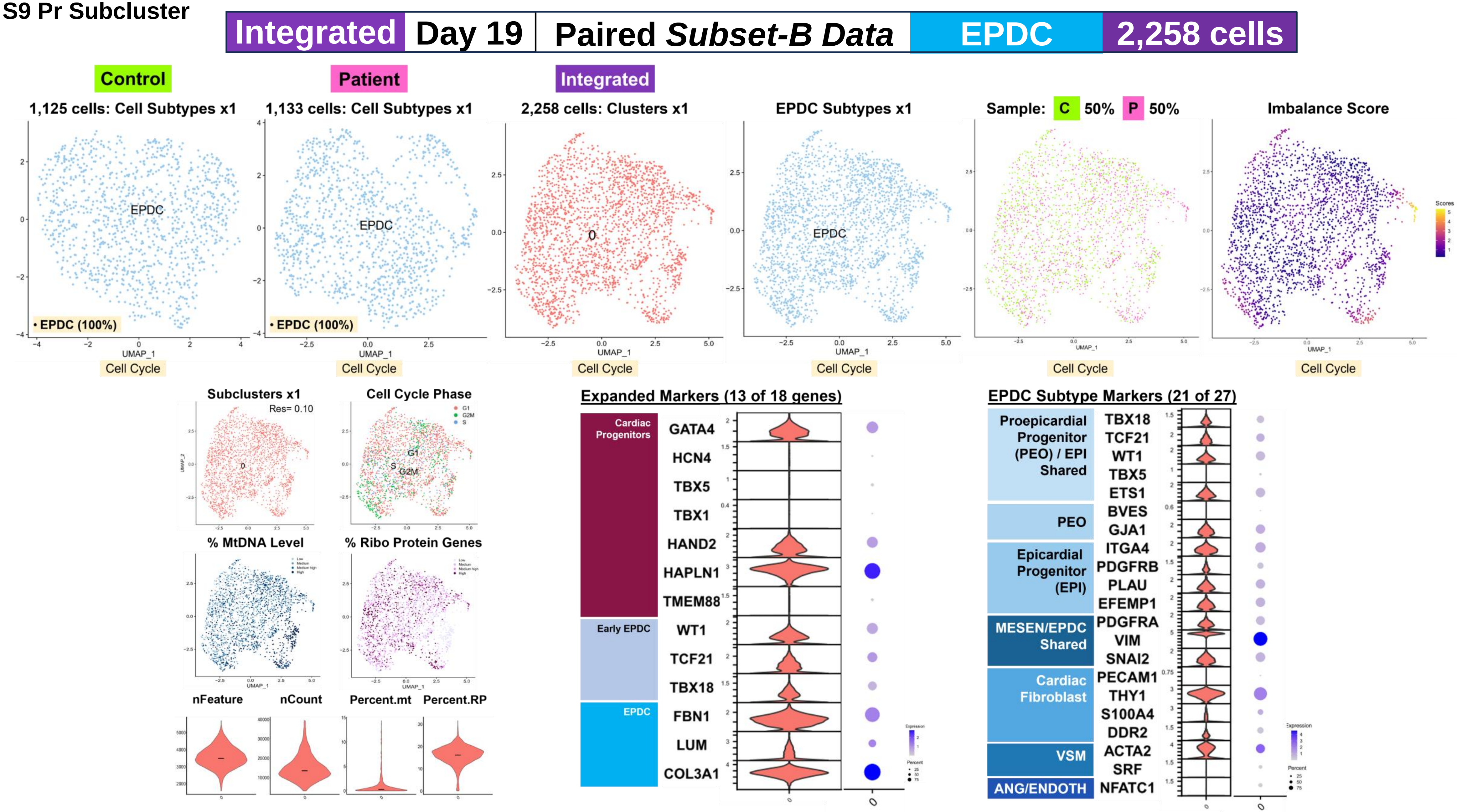

Fig. S9 Pr Subcluster

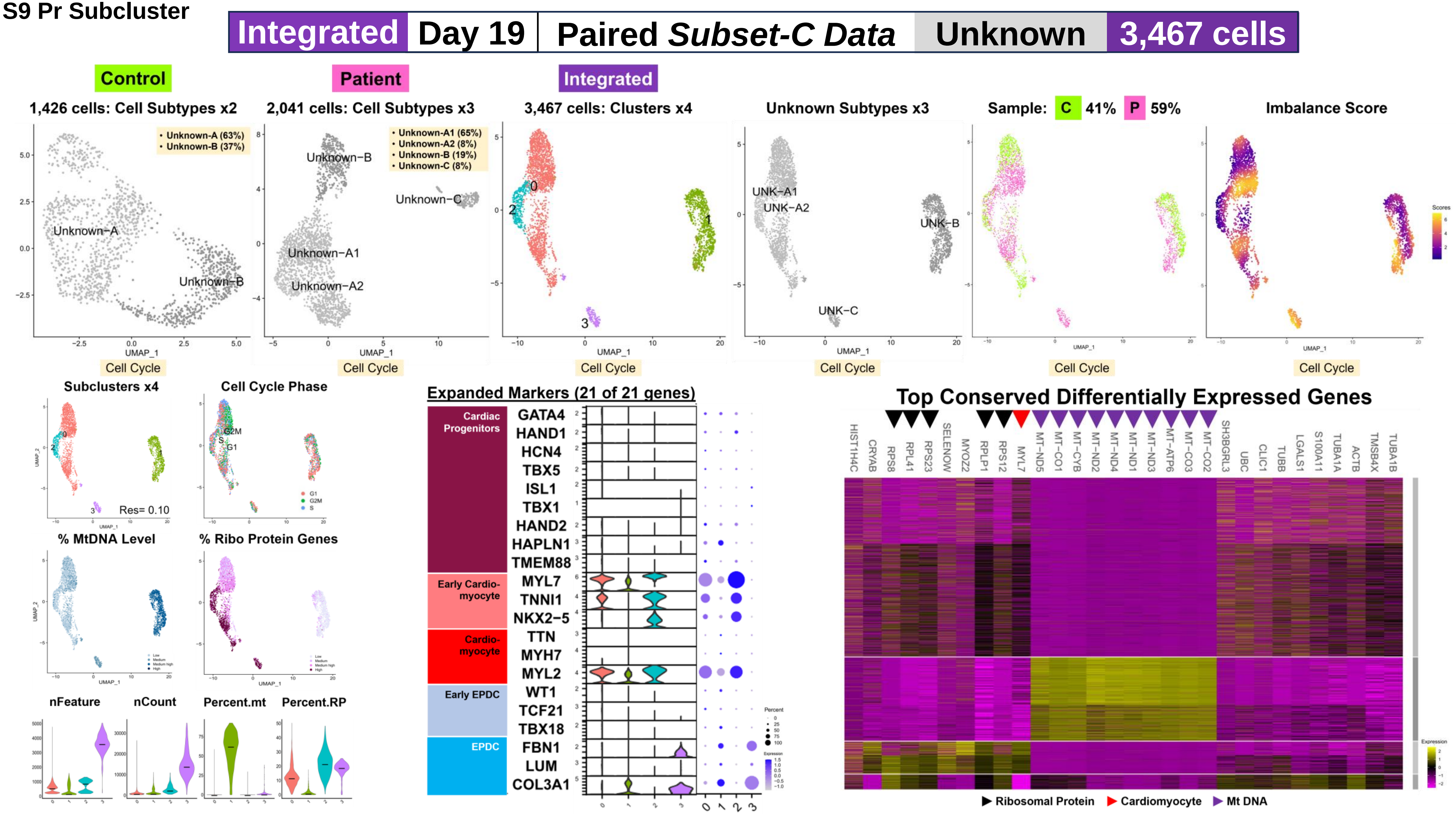

Fig. S10 Cell Type DEG

Workflow Step-III: Paired Sample Data Results

Comparative Analyses for Cell Type-Specific Differential Expression

A. Summary: Cell Type Differentially Expressed Genes (Cell Type DEG)

Integrated Subset Data: Individual Analysis of ‘Balanced’ Cell Subtypes (n=14: 71,541 Total Cells)

Day	INTEGR Subset[a]	Cells	PA1	CA1	Threshold DEGs[b] Total	Threshold DEGs[b] Under	Threshold DEGs[b] Over	ORA[c] GS Under	ORA[c] GS Over	GO Biological Processes: Top Enriched GS[c]		DEGs-NoThreshold Total	DEGs-NoThreshold Under	DEGs-NoThreshold Over	GSEA[d] GS	GSEA MSigDB Hallmark GS: Enriched GS[d]
0	PP-A1A2	18,554	48%	52%	75	22	53	6	0	Ox Phos		6,395	3,022	3,373	0	
	PP-B	716	54%	46%	3	3	0	4	0	RNA Splicing/Dosage Comp		5,942	2,934	3,008	0	
9B	CPROG-A	5,946	49%	51%	82	20	62	2	33	Musc Dev/Diff	Ribosome Process	3,913	2,154	1,759	1	OX PHOS
	CPROG-B	4,750	53%	47%	15	6	9	2	2	Cell Recog/Epigen	Musc Dev/Myofibril	3,259	1,779	1,480	0	
	CPROG-C	1,216	64%	36%	2	2	0	-	0			4,697	2,511	2,186	0	
	CM-A	3,433	35%	65%	26	12	14	0	0			4,018	2,205	1,813	0	
	CM/U-B1B2	4,127	54%	46%	5	3	2	19	-	Gene Silencing/Dosage Comp		167	120	47	0	
	EPDC	1,728	56%	44%	7	3	4	26	45	Dosage Comp	Glycolysis	3,674	1,879	1,795	0	
16	CM-A1A2	8,639	49%	51%	475	136	339	32	131	Musc Dev/Signal	Musc Dev/Ox Phos	4,506	2,773	1,733	5	TGFB SIGNAL/GLY/OXPHOS/EMT/MYC
	CM/U-B	2,472	44%	56%	48	3	45	3	132	Dosage Comp	Metab/Musc Dev	619	398	221	2	GLYCOLYSIS / MYOGENESIS
	EPDC	3,133	68%	32%	80	12	68	0	38	Purine Ribonucleotide Metab		4,594	2,409	2,185	1	MTORC1 SIGNAL
19	CM-A1	9,978	51%	49%	392	165	227	22	118	Musc Diff/Myofibril	Musc Contraction	4,953	2,334	2,619	1	MYOGENESIS
	CM/U-B	4,591	48%	52%	47	8	39	5	72	Signal/Vasc Dev	Musc Contraction	965	395	570	0	
	EPDC	2,258	50%	50%	22	5	17	34	0	Epigen / Heterochromatin		5,846	3,084	2,762	0	
	Total	71,541			1,279	400	879	155	571	Total		53,548	27,997	25,551	10	
	Average	5,110	52%	48%	91	29	63	12	44	Average		3,825	2,000	1,825	0.7	
	Median	4,127			47	8	39	5	36	Median		4,506	2,334	1,813	0	

[a]Integrated (INTEGR) Data Subsets with primarily ‘balanced’ shared cell subtypes (n=14) used for Cell Type Differential Expression Analyses

[b]DEG Threshold: Adjusted p-value < 10e-50 & I Avg Log2 FC I > 0.25, Fold Change = 1.2x

[c]ORA Parameters: min # genes= 3, Adj p-value < 0.05, q-value cutoff 0.20; Highlighted in Red= Underexpressed GS and Green=Overexpressed GS in Patient cells compared to Control cells

[d]GSEA Parameters: Adj p-value cutoff= 0.05, GS size 10-500 genes; Highlighted in Pink= Downregulated GS; Bright Green= Upregulated GS in Patient compared to Control cells

Abbreviations: PP, Pluripotent; CPROG, Cardiac Progenitors; CM, Cardiomyocyte; EPDC, Epicardium-derived cells; U, Unknown; DEG, differentially expressed gene; ORA, Over-Representation Analysis; GS, Gene Set; GSEA, Gene Set Enrichment Analysis; MSigDB, Molecular Signatures Database; Ox Phos, Oxidative Phosphorylation; Comp, Compensation; Musc, Muscle; Dev, Development; Diff, Differentiation; Recog, Recognition; Epigen, Epigenetic; Metab, Metabolism; Signal, Signaling; Vasc, Vascular; EMT, Epithelial-Mesenchymal Transition; MYC, MYC Targets; Gly, Glycolysis

Fig. S10 Cell Type DEG

B. Summary: Volcano Plots and Cell Type DEG (n=14 Cell Subtypes: 71,541 Total Cells)

Day 00: x2 Cell Subtypes

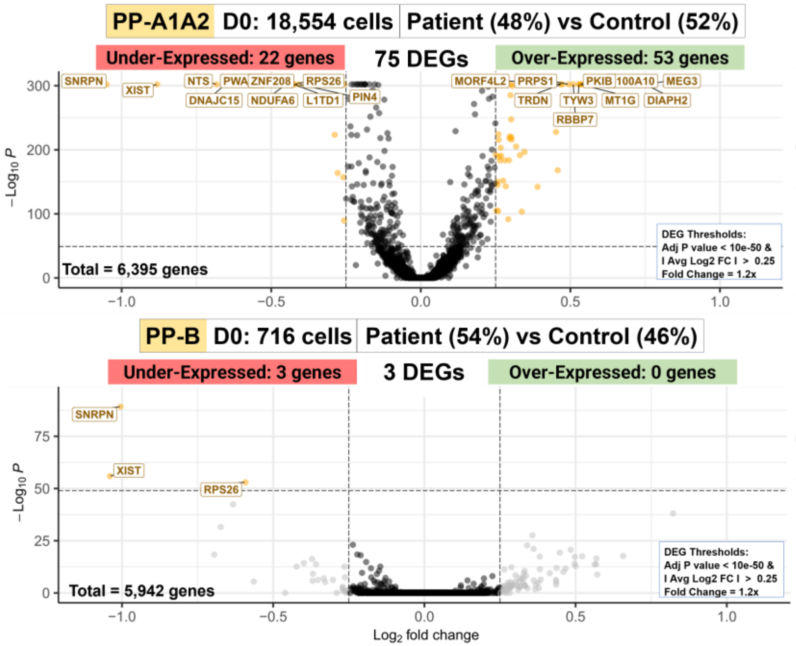

Top 10 Cell Type Threshold DEG are labeled.

Day 09B: x3 Cell Subtypes

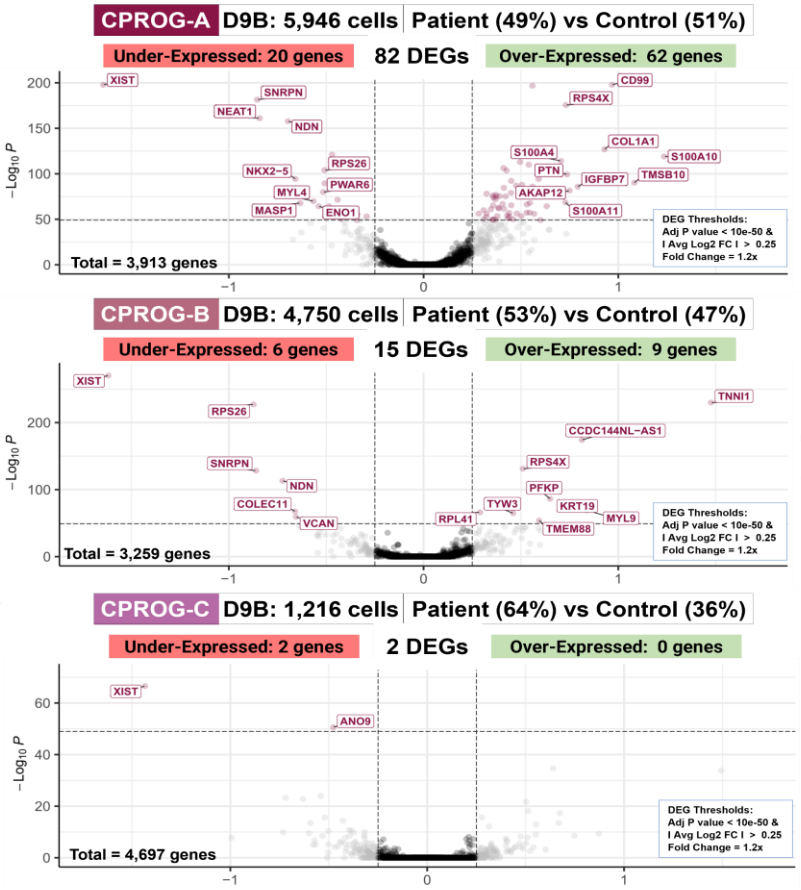

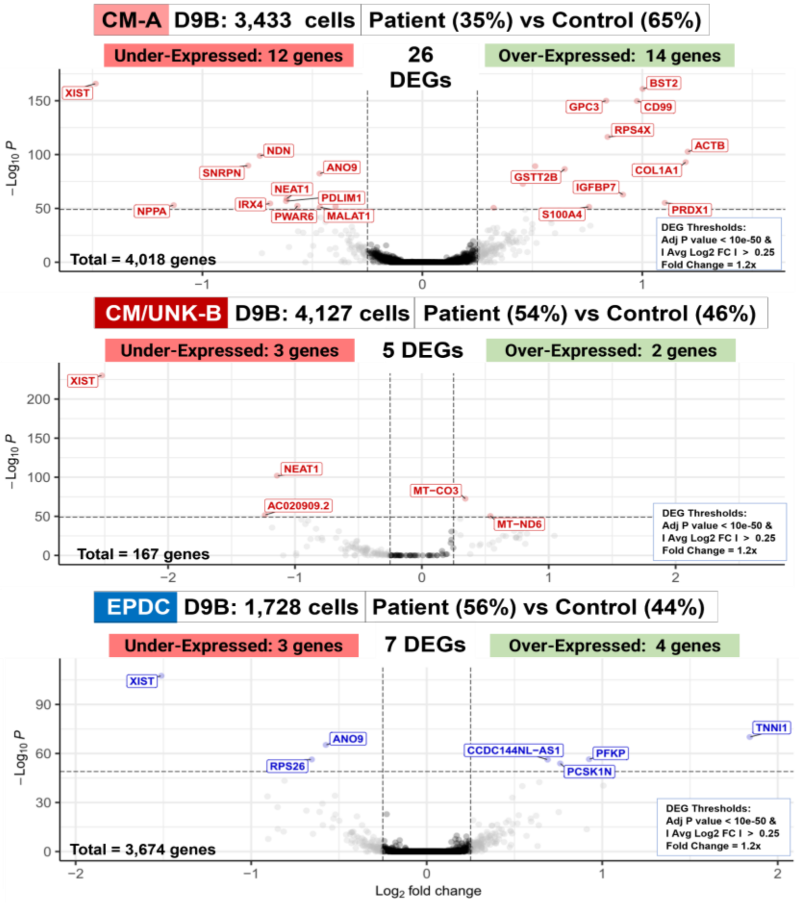

Fig. S10 Cell Type DEG

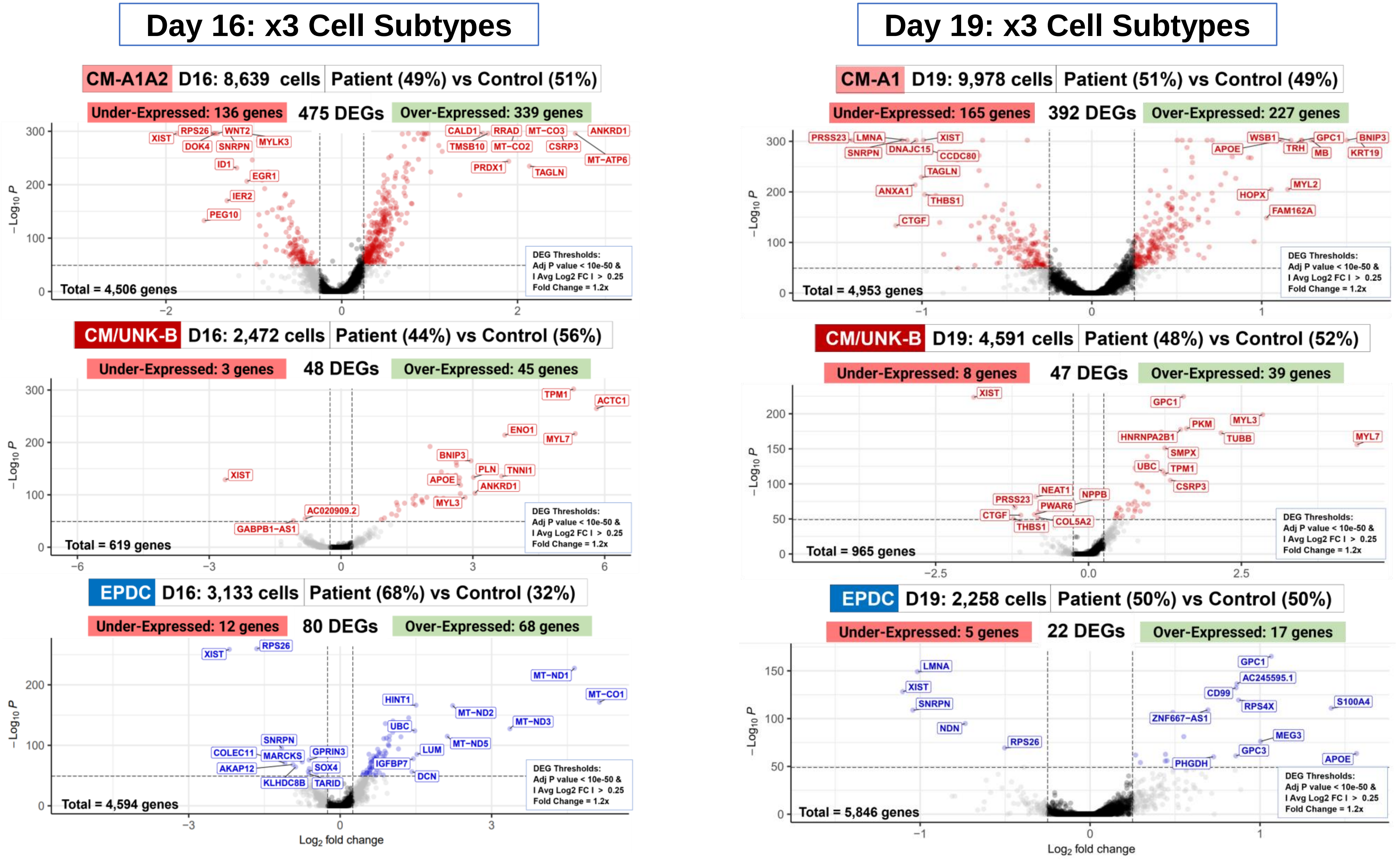

Fig. S10 Cell Type DEG

C. Individual Analyses of Integrated Subsets: Cell Type DE Analyses and Enrichment (n=14)

Integrated	D00	PP-A1A2	18,554 cells

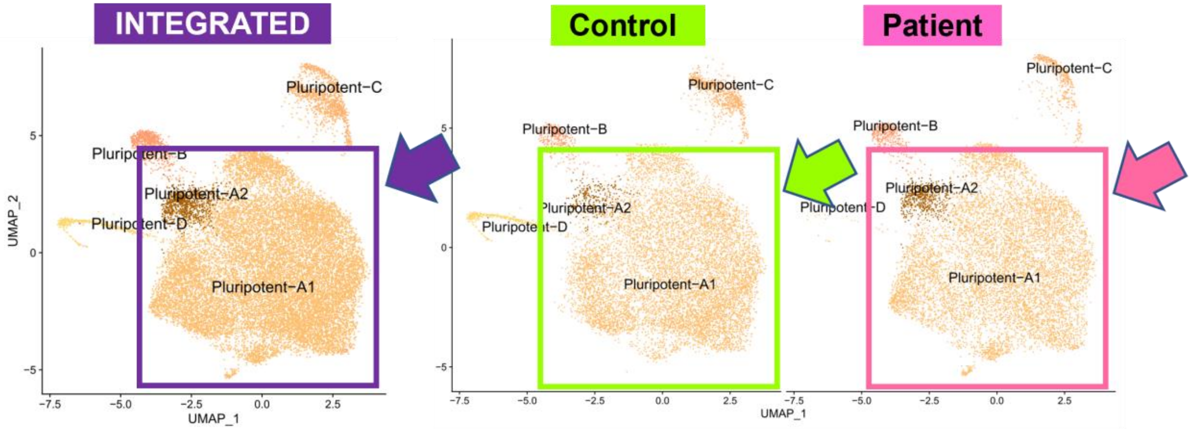

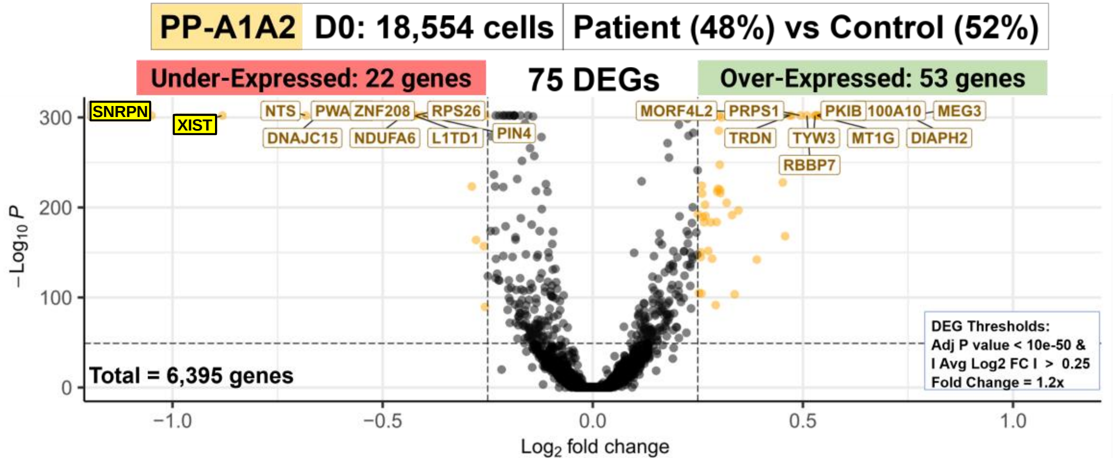

Over-Representation Analysis (ORA)

Under-Expressed: 22 genes Enriched Gene Sets = 6

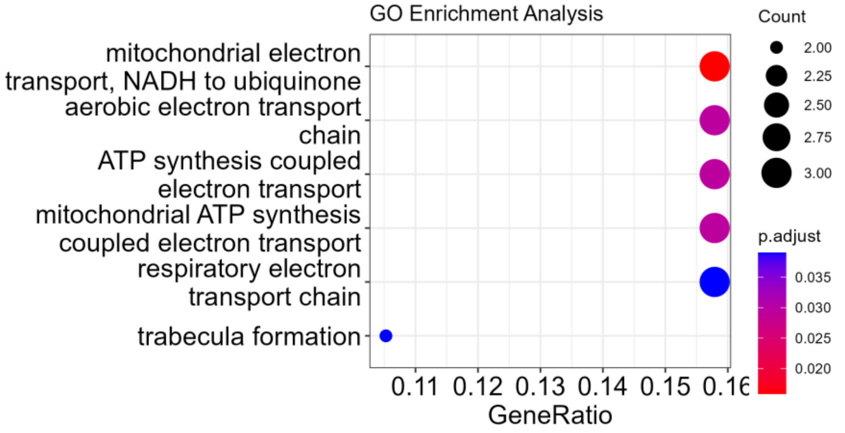

Over- Expressed: 53 genes Enriched Gene Sets = 0

Enrichment Map

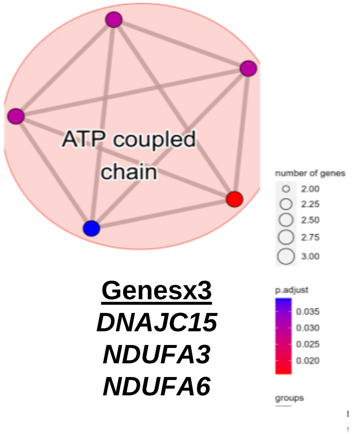

Genesx3
DNAJC15
NDUFA3
NDUFA6

Genesx2
HEY2
SFRP1

trabecula formation

Gene Set Enrichment Analysis (GSEA)

All 6395 DEGs
Under-expressed: 3022 genes
Over-expressed: 3373 genes

No Enriched MSigDB Hallmark Gene Sets

Fig. S10 Cell Type DEG

Integrated | D00 | PP-B | 716 cells

INTEGRATED | **Control** | **Patient**

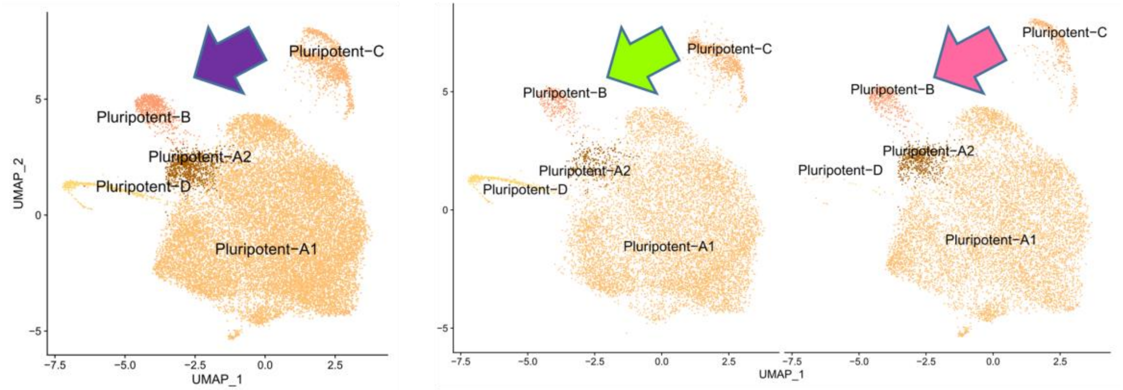

PP-B | D0: 716 cells | Patient (54%) vs Control (46%)

Under-Expressed: 3 genes | **3 DEGs** | **Over-Expressed: 0 genes**

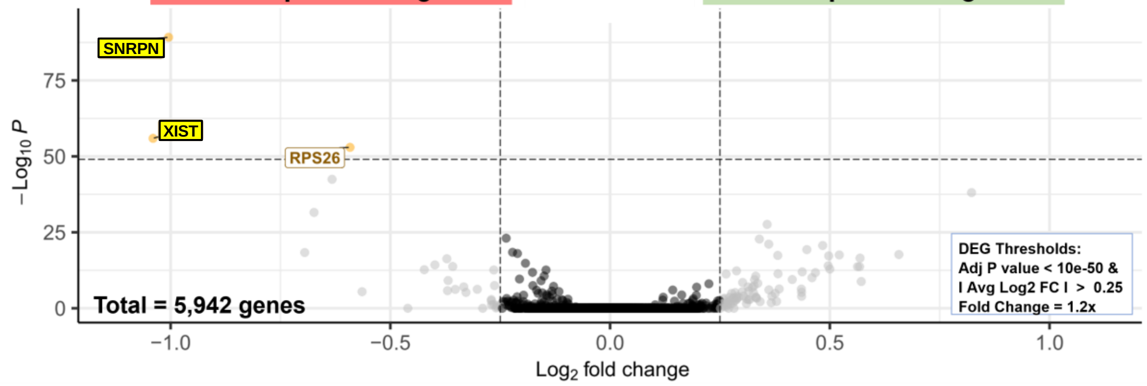

Over-Representation Analysis (ORA)

Under-Expressed: 3 genes — **Enriched Gene Sets = 4**

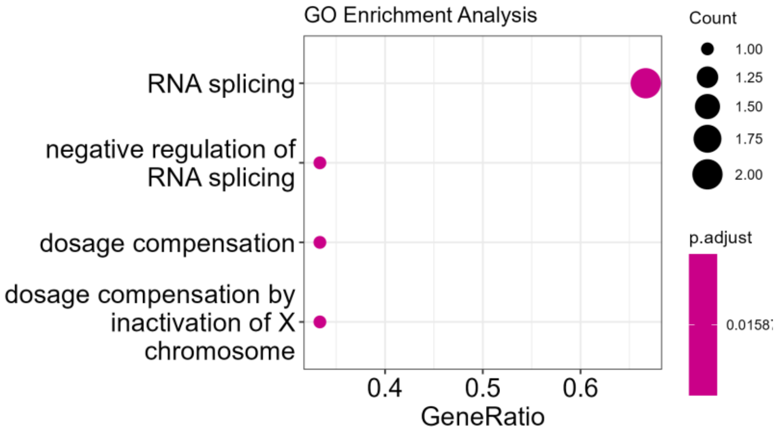

Enrichment Map

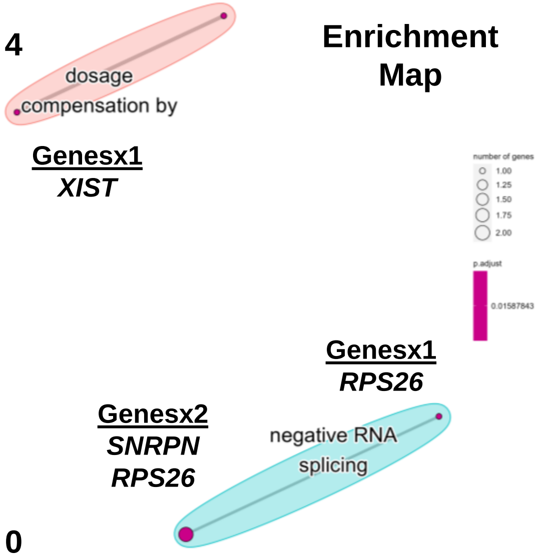

Over- Expressed: 0 genes — **Enriched Gene Sets = 0**

Gene Set Enrichment Analysis (GSEA)

All 5942 DEGs
Under-expressed: 2934 genes
Over-expressed: 3008 genes

No Enriched MSigDB Hallmark Gene Sets

Fig. S10 Cell Type DEG

Integrated | D09B | CPROG-A | 5,946 cells

INTEGRATED | **Control** | **Patient**

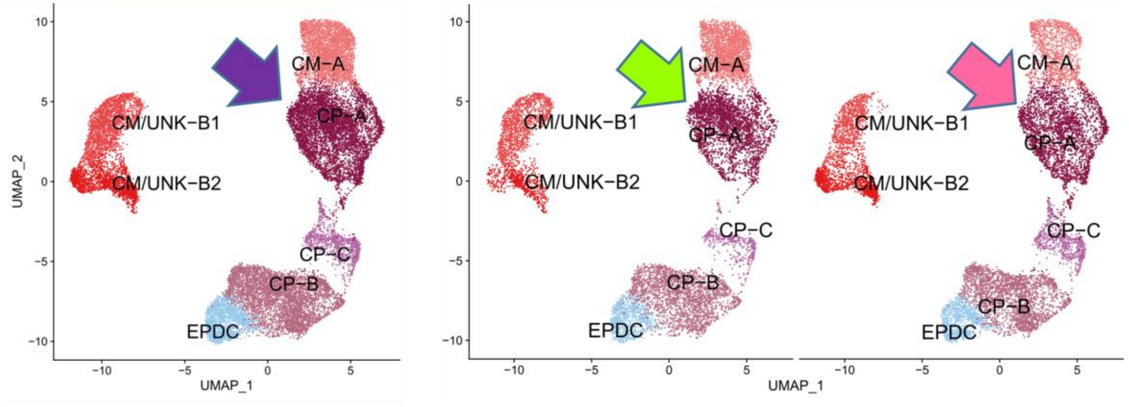

CPROG-A | D9B: 5,946 cells | Patient (49%) vs Control (51%)

Under-Expressed: 20 genes | **82 DEGs** | **Over-Expressed: 62 genes**

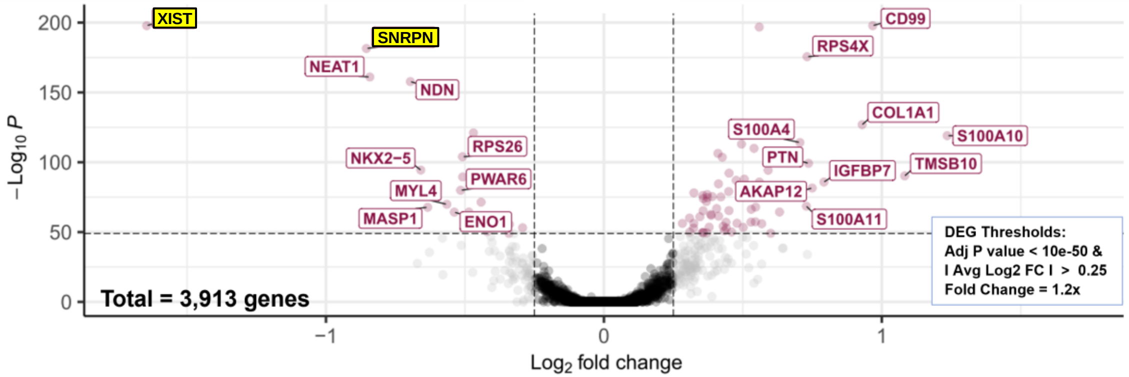

Over-Representation Analysis (ORA)

Under-Expressed: 20 genes | **Over- Expressed: 62 genes**

Enriched Gene Sets = 2

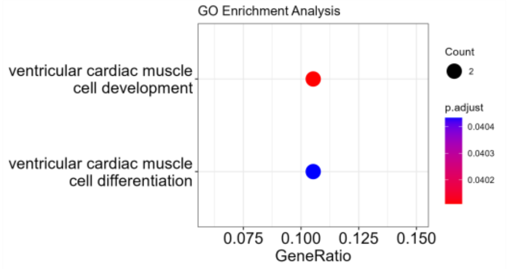

Enriched Gene Sets = 33

Top 5

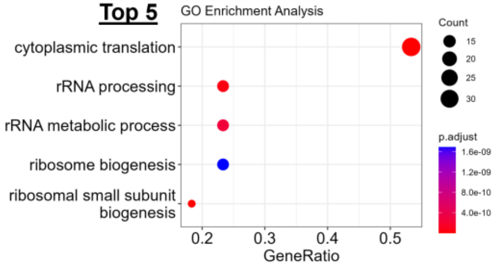

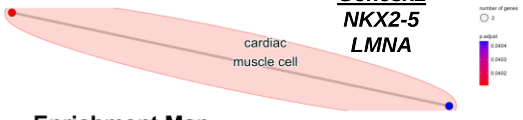

Genesx2
NKX2-5
LMNA

Enrichment Map
Gene Sets = 2

Enrichment Map
Top 25 Gene Sets

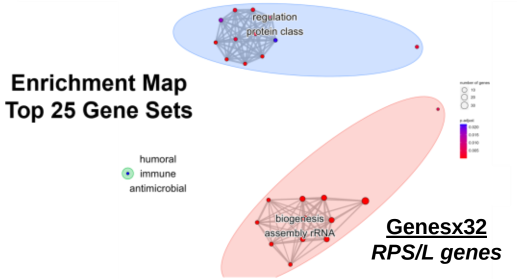

Genesx32
RPS/L genes

Gene Set Enrichment Analysis (GSEA)

Patient vs Control

All 3913 DEGs
Under-expressed: 2154 genes
Over-expressed: 1759 genes

Enriched gene sets x1

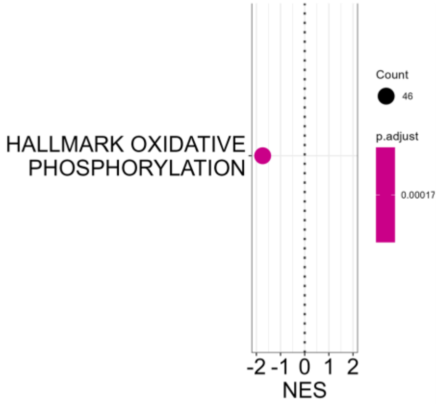

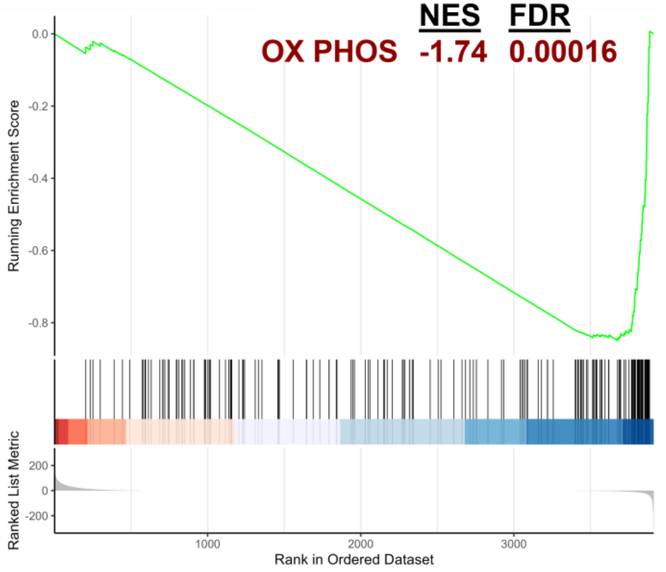

Fig. S10 Cell Type DEG

Integrated | D09B | CPROG-B | 4,750 cells

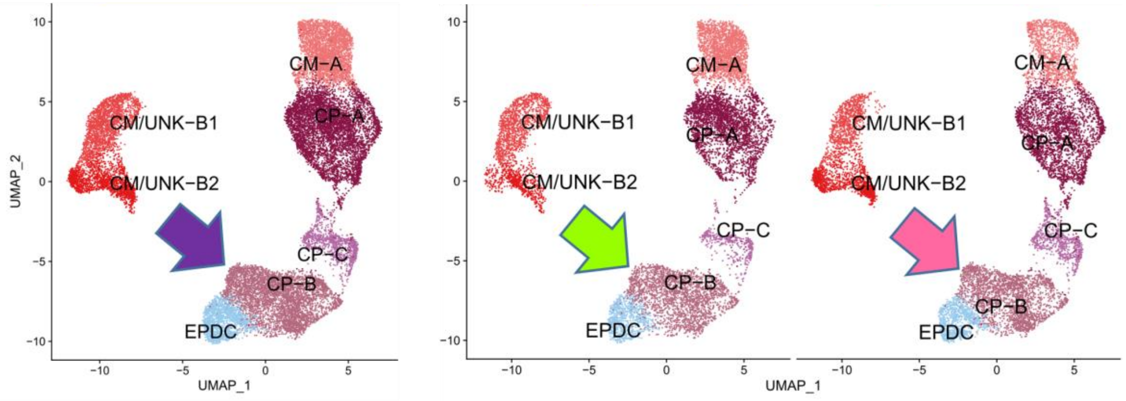

CPROG-B | D9B: 4,750 cells | Patient (53%) vs Control (47%)

Under-Expressed: 6 genes | 15 DEGs | Over-Expressed: 9 genes

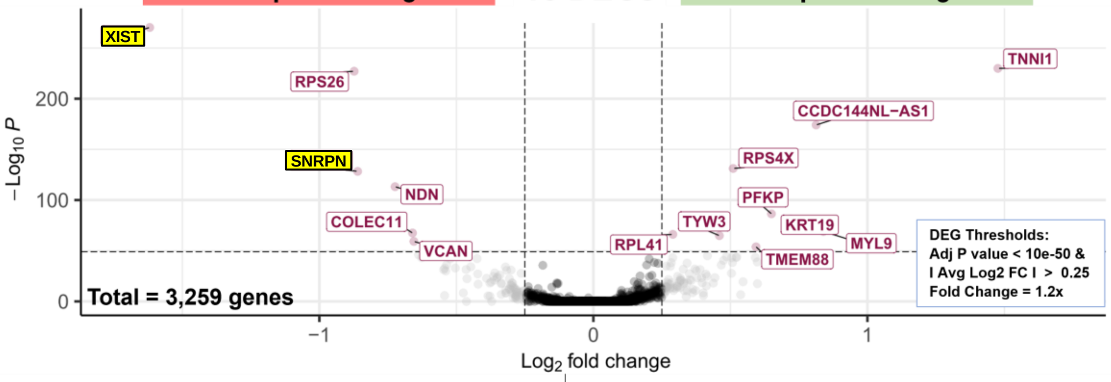

Over-Representation Analysis (ORA)

Under-Expressed: 6 genes

Enriched Gene Sets = 2

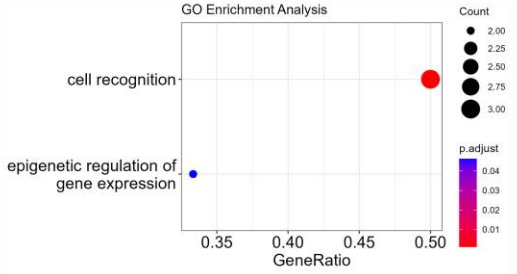

Over- Expressed: 9 genes

Enriched Gene Sets = 2

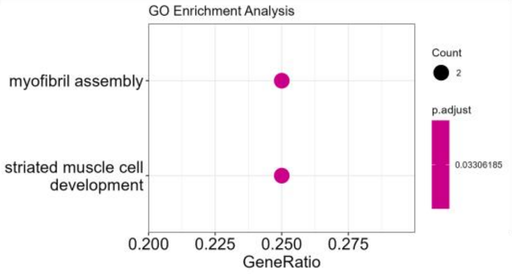

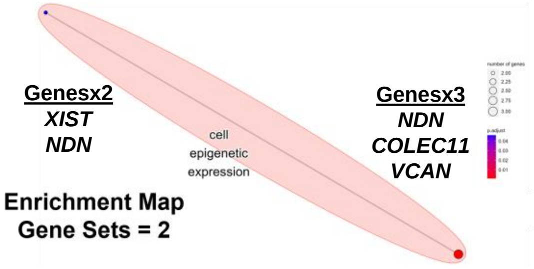

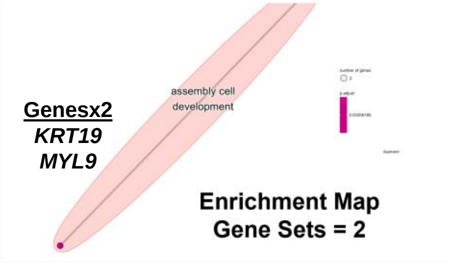

Gene Set Enrichment Analysis (GSEA)

All 3259 DEGs
Under-expressed: 1779 genes
Over-expressed: 1480 genes

No Enriched MSigDB Hallmark Gene Sets

Fig. S10 Cell Type DEG

Integrated | D09B | CPROG-C | 1,216 cells

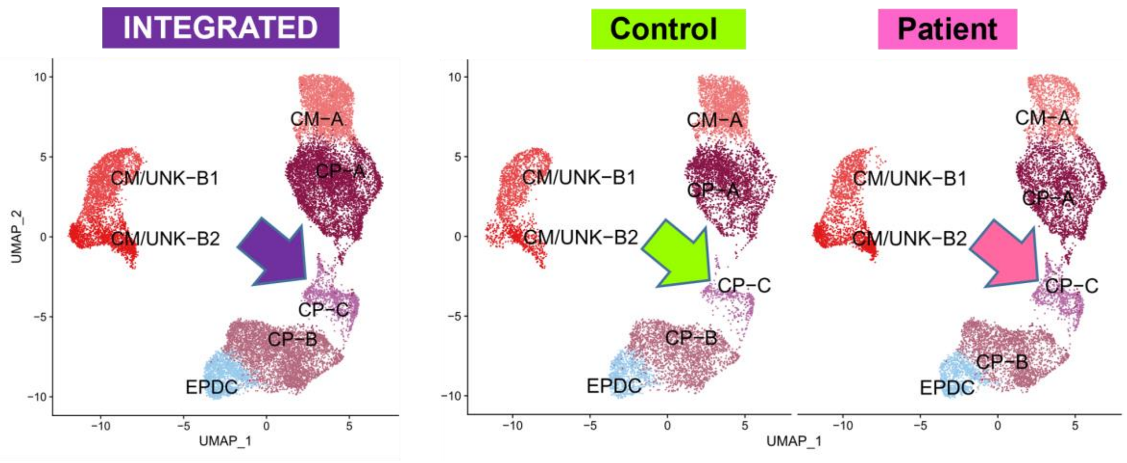

CPROG-C | D9B: 1,216 cells | Patient (64%) vs Control (36%)

Under-Expressed: 2 genes | 2 DEGs | Over-Expressed: 0 genes

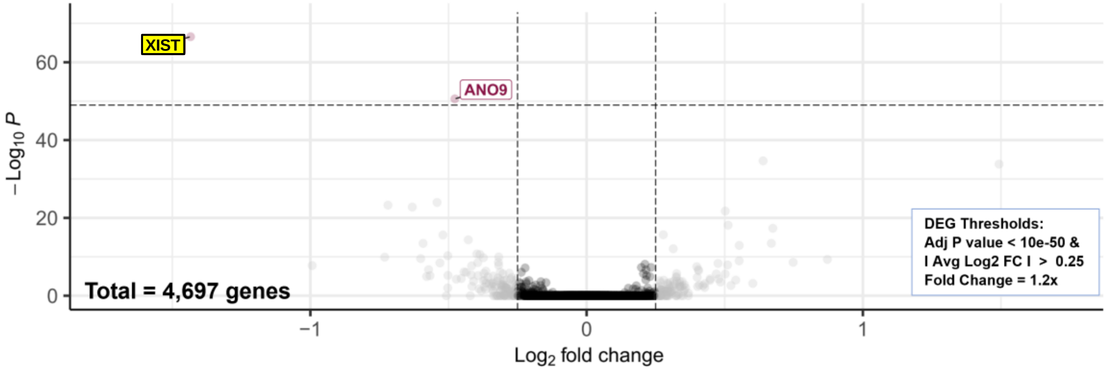

Over-Representation Analysis (ORA)

Under-Expressed: 2 genes

NOT DONE

Over- Expressed: 0 genes

Enriched Gene Sets = 0

Gene Set Enrichment Analysis (GSEA)

All 4697 DEGs
Under-expressed: 2511 genes
Over-expressed: 2186 genes

No Enriched MSigDB Hallmark Gene Sets

Fig. S10 Cell Type DEG

Integrated	D09B	CM-A	3,433 cells

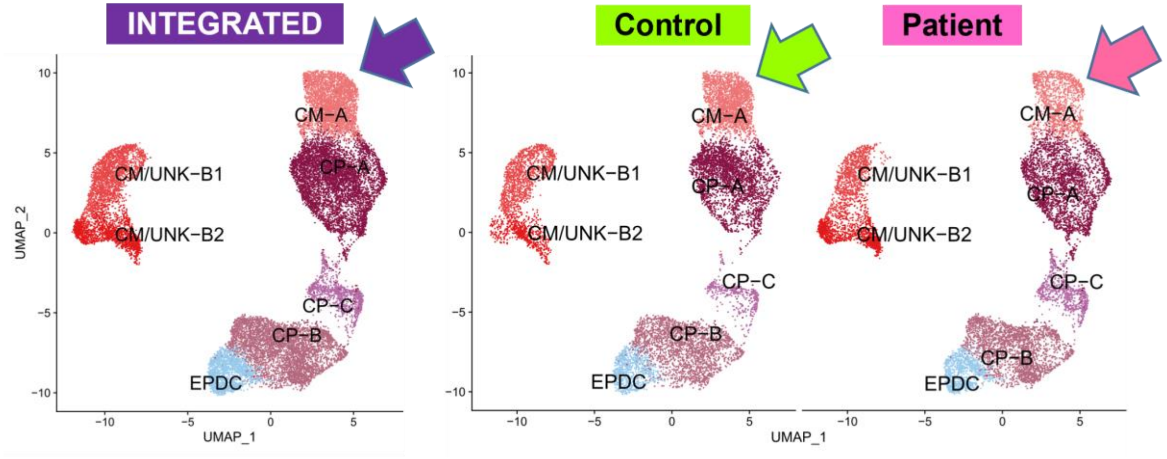

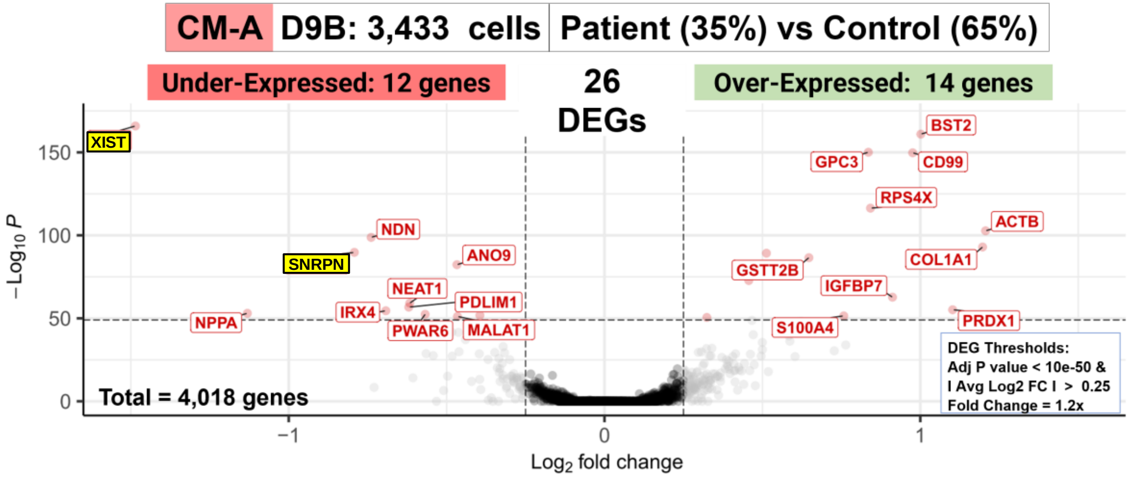

Over-Representation Analysis (ORA)

Under-Expressed: 12 genes — No Enriched GO Biological Process Gene Sets

Over-Expressed: 14 genes — No Enriched GO Biological Process Gene Sets

Gene Set Enrichment Analysis (GSEA)

All 4018 DEGs
Under-expressed: 2205 genes
Over-expressed: 1813 genes

No Enriched MSigDB Hallmark Gene Sets

Fig. S10 Cell Type DEG

Integrated | D09B | CM/UNK-B | 4,127 cells

INTEGRATED   Control   Patient

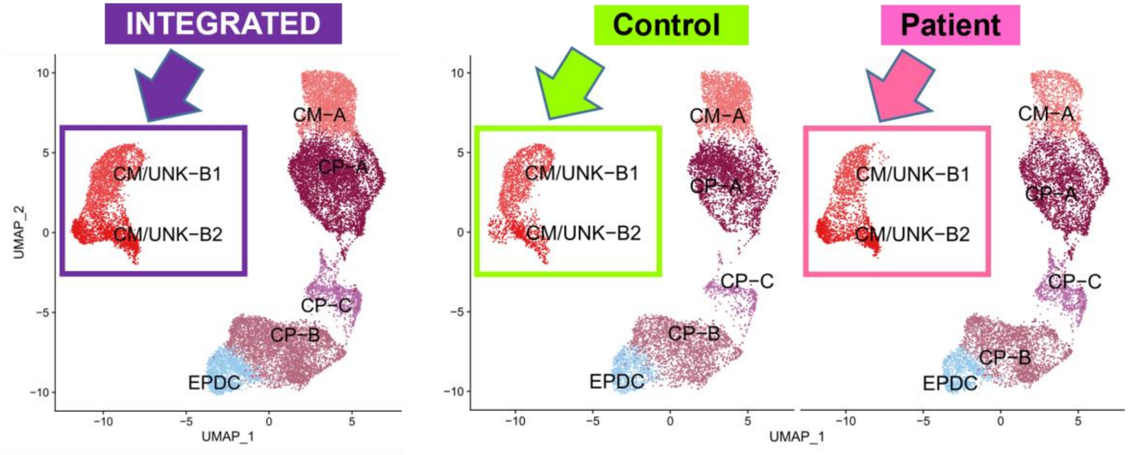

CM/UNK-B | D9B: 4,127 cells | Patient (54%) vs Control (46%)

Under-Expressed: 3 genes   **5 DEGs**   Over-Expressed: 2 genes

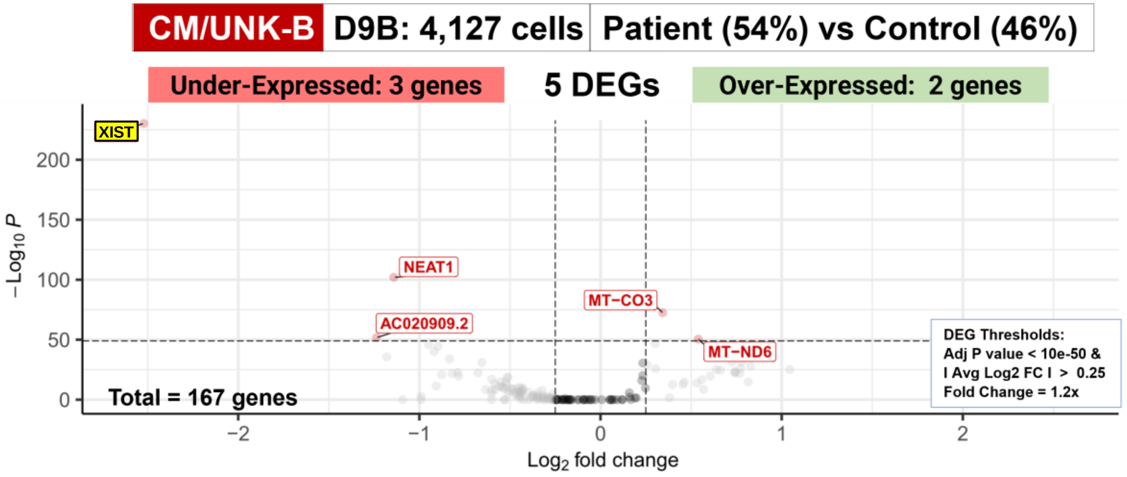

Over-Representation Analysis (ORA) RA)

Under-Expressed: 3 genes

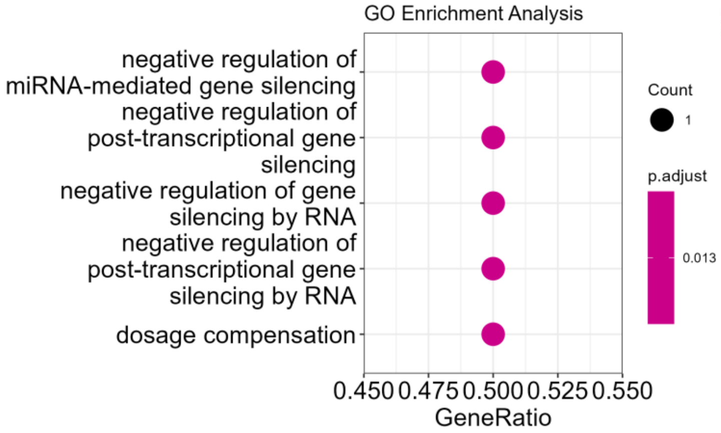

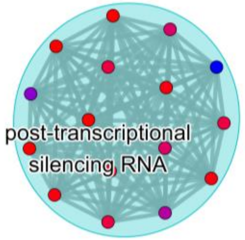

Genesx1
NEAT1

Enrichment Map

Enriched Gene Sets = 19

Over- Expressed: 2 genes   **NOT DONE**

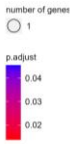

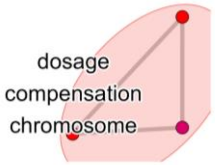

Genesx1
XIST

Gene Set Enrichment Analysis (GSEA)

All 167 DEGs
Under-expressed: 120 genes
Over-expressed: 47 genes

No Enriched MSigDB Hallmark Gene Sets

Fig. S10 Cell Type DEG

Integrated	D09B	EPDC	1,728 cells

INTEGRATED | Control | Patient

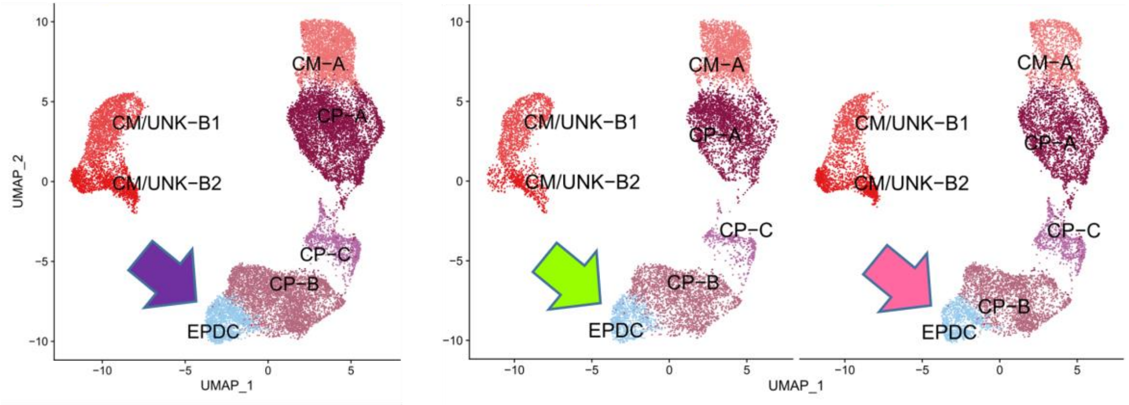

EPDC	D9B: 1,728 cells	Patient (56%) vs Control (44%)

Under-Expressed: 3 genes | 7 DEGs | Over-Expressed: 4 genes

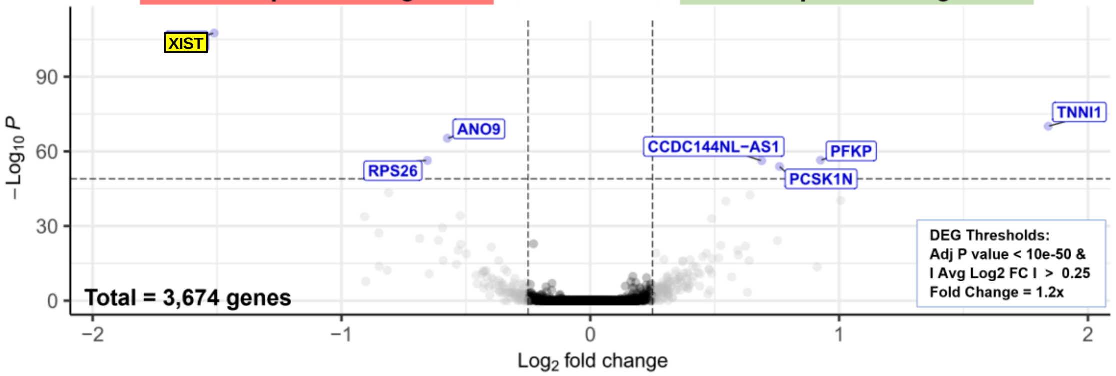

Over-Representation Analysis (ORA)

Under-Expressed: 3 genes

Enriched Gene Sets = 26

Top 5

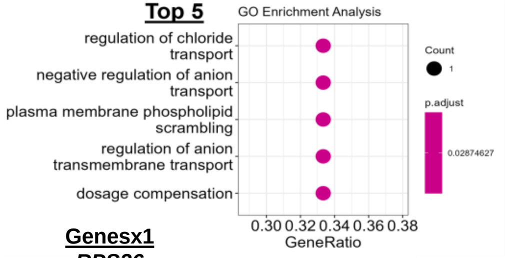

Over- Expressed: 4 genes

Enriched Gene Sets = 45

Top 5

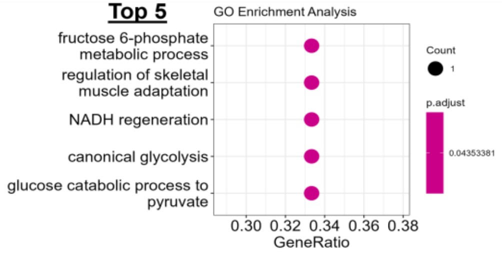

Enrichment Maps Top 25 Gene Sets

Genesx1 *RPS26*

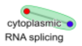

Genesx1 *ANO9*

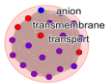

Genesx1 *XIST*

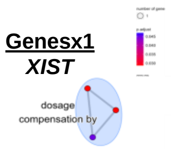

Genesx1 *TNNI1*

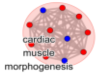

Genesx1 *PFKP*

Genesx1 *PCSK1N*

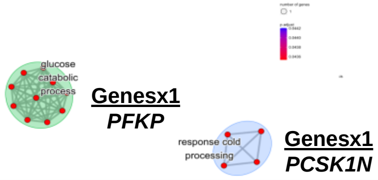

Gene Set Enrichment Analysis (GSEA)

All 3674 DEGs
Under-expressed: 1879 genes
Over-expressed: 1795 genes

No Enriched MSigDB Hallmark Gene Sets

Fig. S10 Cell Type DEG

Integrated | D16 | CM-A1A2 | 8,639 cells

INTEGRATED | Control | Patient

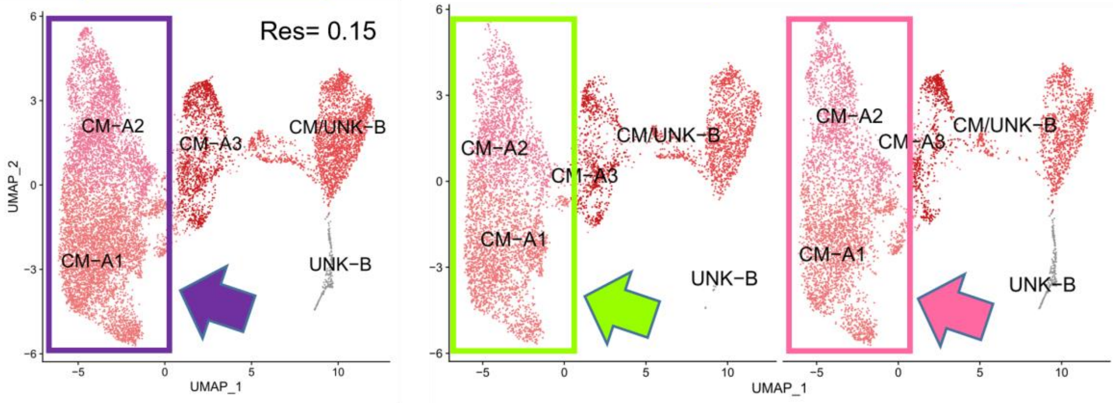

CM-A1A2 | D16: 8,639 cells | Patient (49%) vs Control (51%)

Under-Expressed: 136 genes | 475 DEGs | Over-Expressed: 339 genes

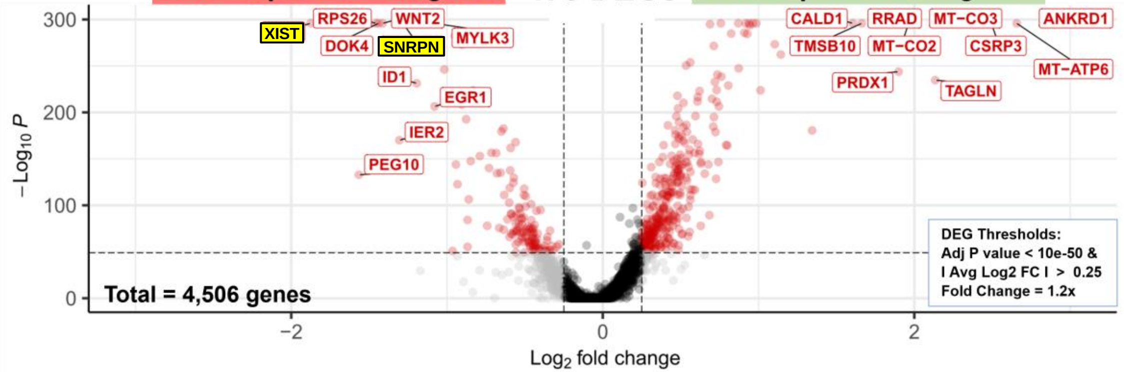

Over-Representation Analysis (ORA)

Under-Expressed: 136 genes | **Over- Expressed: 339 genes**

Enriched Gene Sets = 32 | Enriched Gene Sets = 131

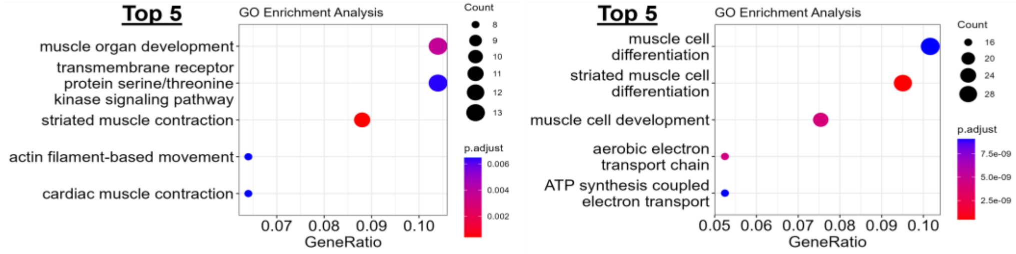

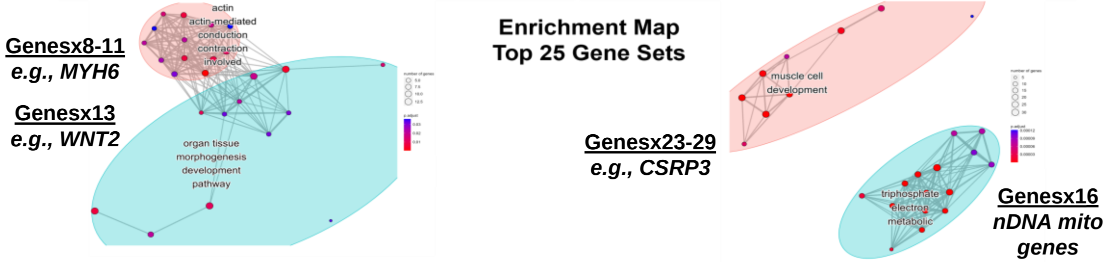

Gene Set Enrichment Analysis (GSEA)

All 4506 DEGs
Under-expressed: 2773 genes
Over-expressed: 1733 genes

Enriched gene sets x5

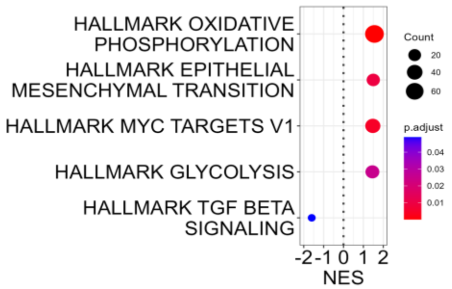

Patient vs Control

	NES	FDR
OX PHOS	1.57	<0.0001
EMT	1.50	0.007
MYC TARGETS V1	1.47	0.004
GLYCOLYSIS	1.45	0.020
TGFB SIGNAL	-1.60	0.039

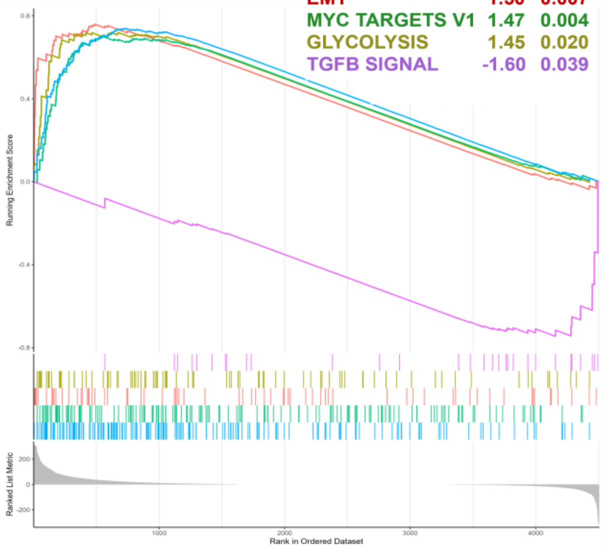

Fig. S10 Cell Type DEG

Integrated | D16 | CM/UNK-B | 2,472 cells

INTEGRATED **Control** **Patient**

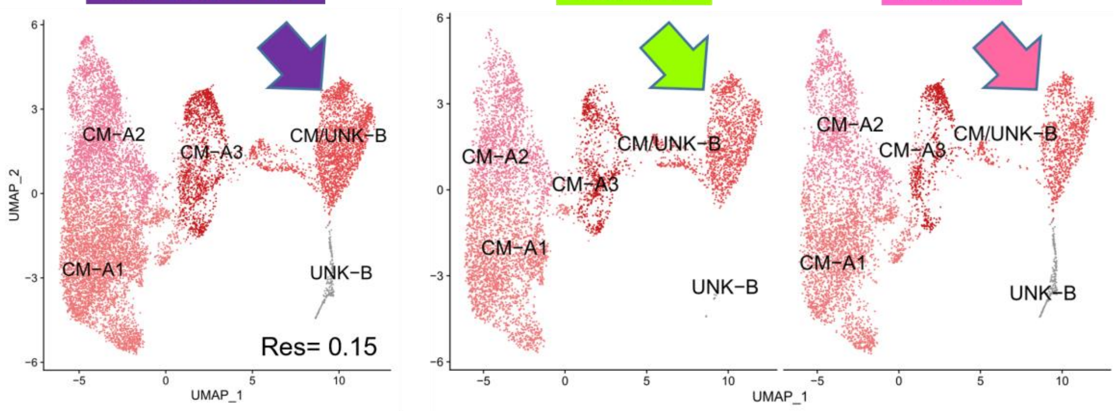

CM/UNK-B | D16: 2,472 cells | Patient (44%) vs Control (56%)

Under-Expressed: 3 genes **48 DEGs** **Over-Expressed: 45 genes**

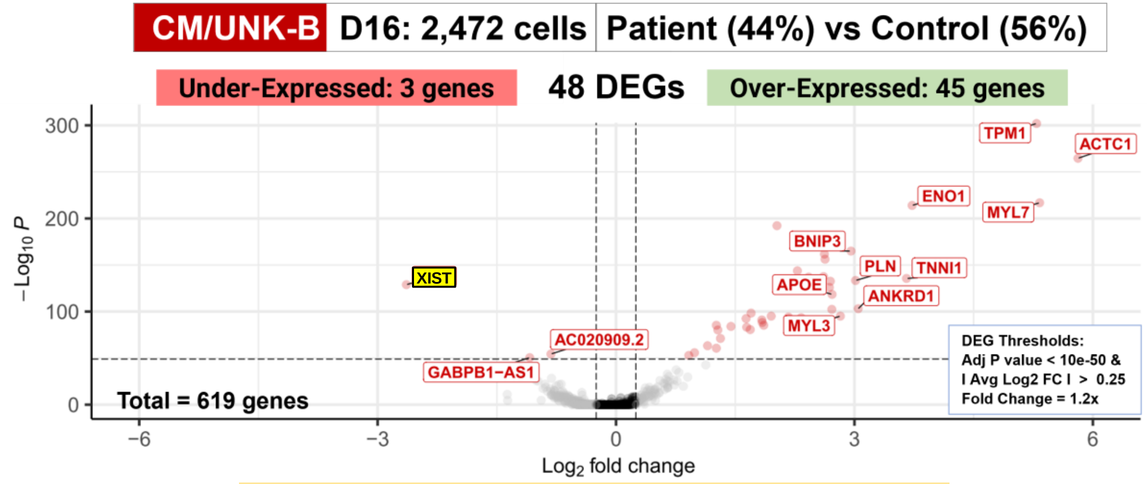

Over-Representation Analysis (ORA)

Under-Expressed: 3 genes

Enriched Gene Sets = 3

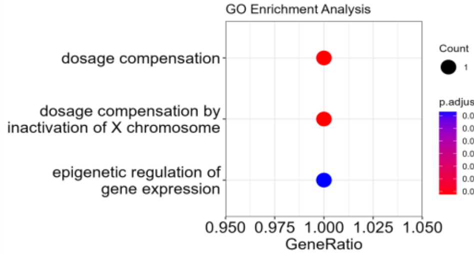

Over- Expressed: 45 genes

Enriched Gene Sets = 132

Top 5

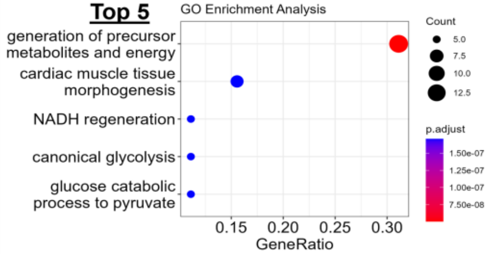

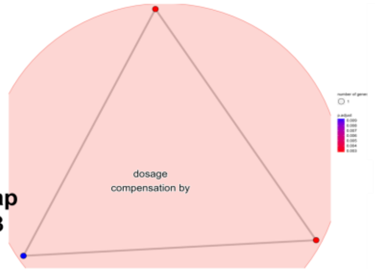

Genesx1
XIST

Enrichment Map
Gene Sets = 3

Enrichment Map
Top 25 Gene Sets

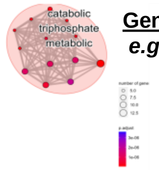

Genesx5-14
e.g., ENO1

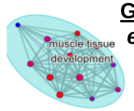

Genesx7-10
e.g., TPM1
ACTC1
TNNI1

Gene Set Enrichment Analysis (GSEA)

All 619 DEGs
Under-expressed: 398 genes
Over-expressed: 221 genes

Enriched gene sets x2

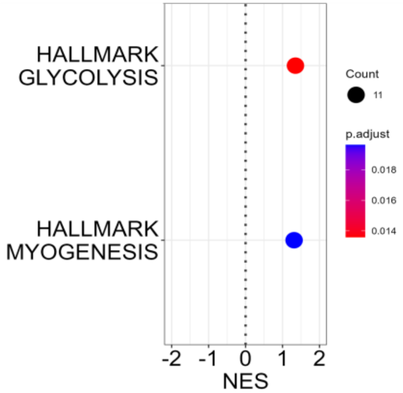

Patient vs Control

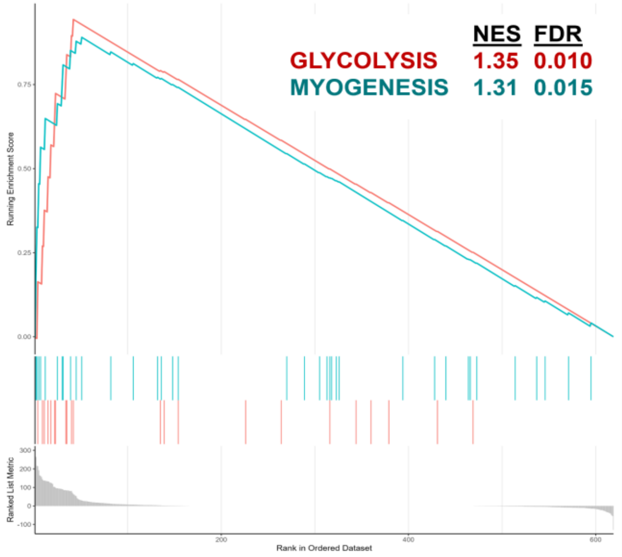

Fig. S10 Cell Type DEG

Integrated | D16 | EPDC | 3,133 cells

INTEGRATED | Control | Patient

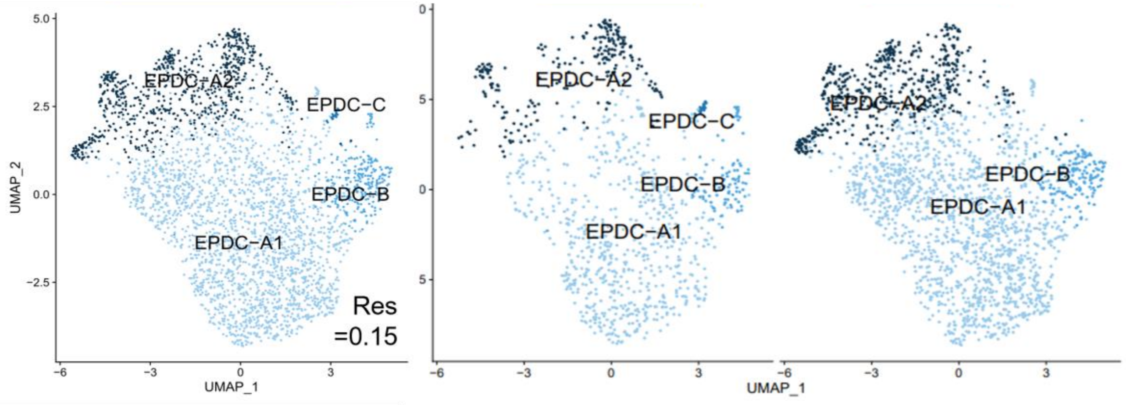

EPDC | D16: 3,133 cells | Patient (68%) vs Control (32%)

Under-Expressed: 12 genes | **80 DEGs** | Over-Expressed: 68 genes

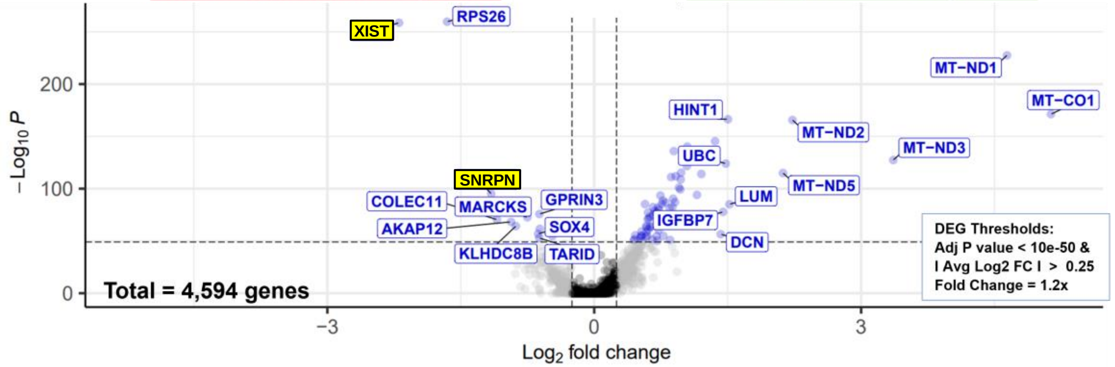

Over-Representation Analysis (ORA)

Under-Expressed: 12 genes

Enriched Gene Sets = 0

No Enrichment:
GO Biological Process Gene Sets

Over- Expressed: 68 genes

Enriched Gene Sets = 38

Top 5

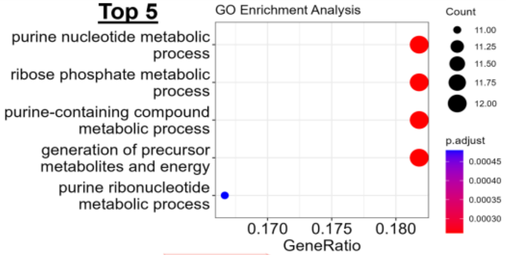

Enrichment Map
Top 25 Gene Sets

Genesx12
e.g., HINT1

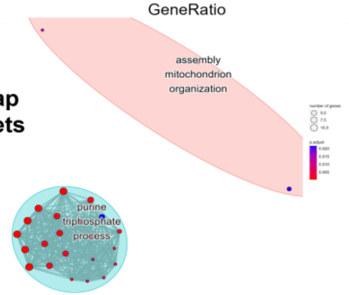

Gene Set Enrichment Analysis (GSEA)

All 4594 DEGs
Under-expressed: 2409 genes
Over-expressed: 2185 genes

Enriched gene sets x1

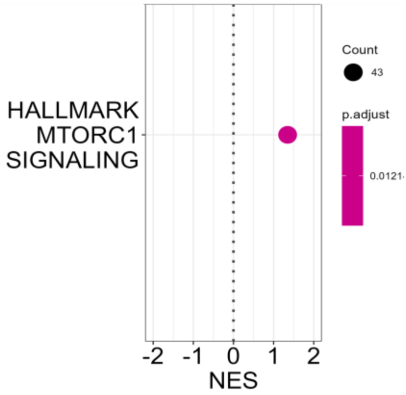

Patient vs Control

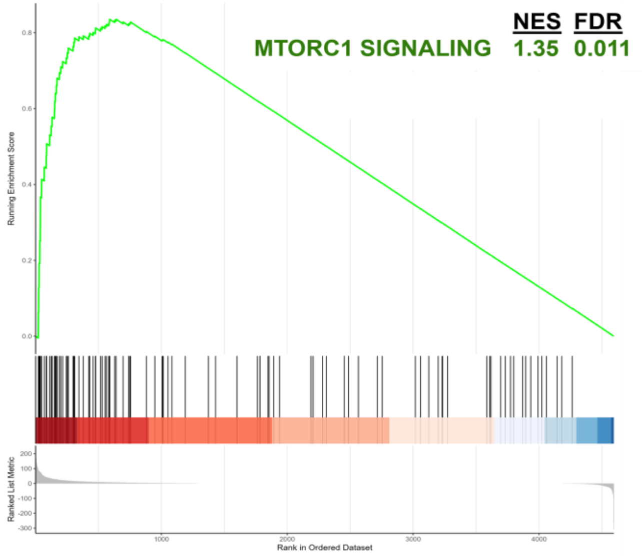

Fig. S10 Cell Type DEG

Integrated | D19 | CM-A1 | 9,978 cells

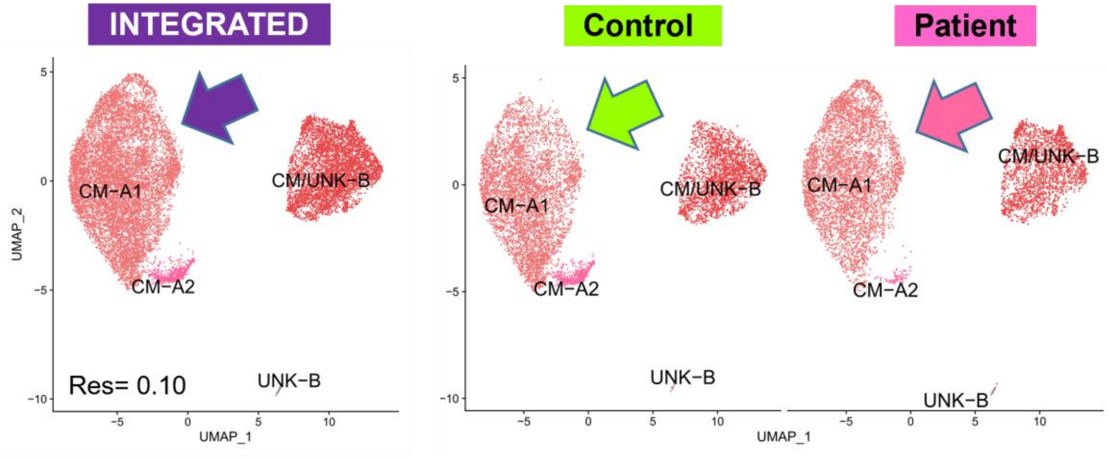

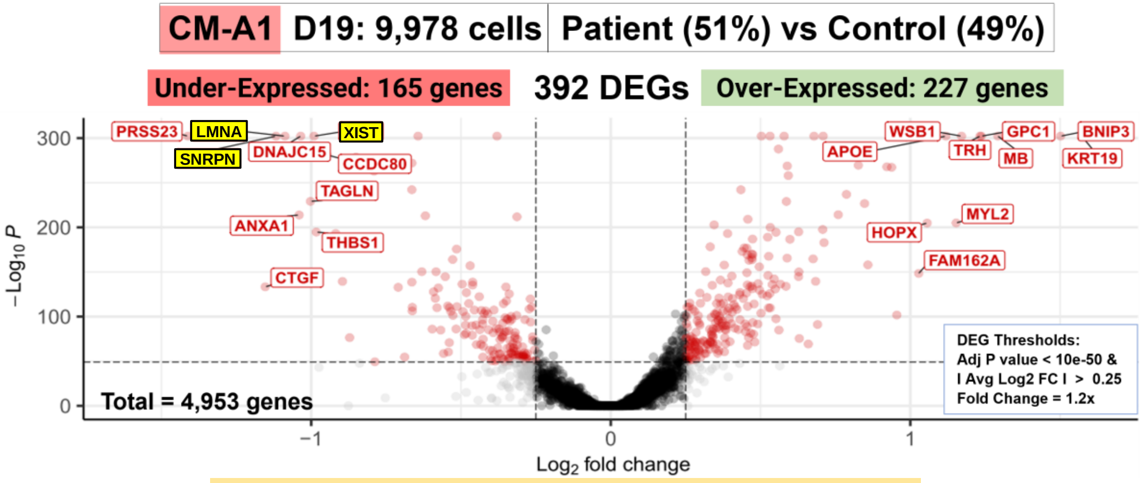

Over-Representation Analysis (ORA)

Under-Expressed: 165 genes

Enriched Gene Sets = 22

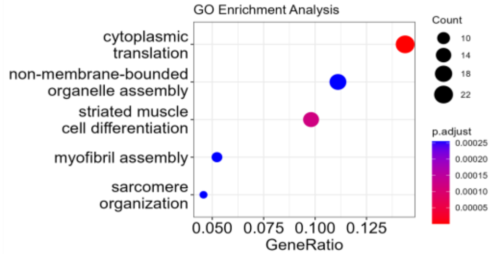

Over- Expressed: 227 genes

Enriched Gene Sets = 118

Top 5

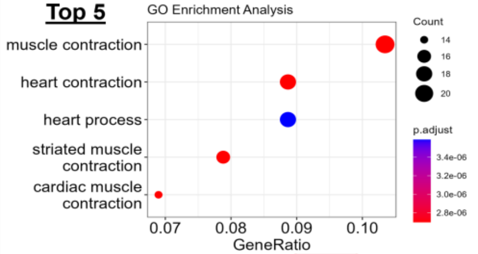

Enrichment Map
Gene Sets = 22

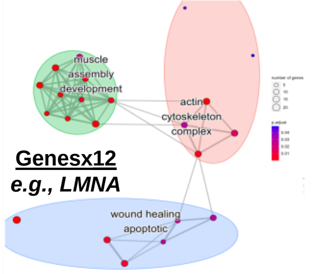

Enrichment Map
Top 25 Gene Sets

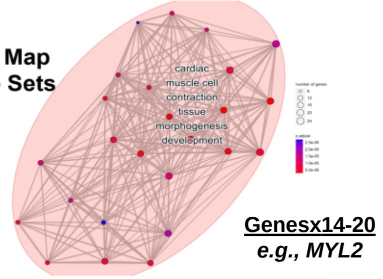

Gene Set Enrichment Analysis (GSEA)

Patient vs Control

All 4953 DEGs
Under-expressed: 2334 genes
Over-expressed: 2619 genes

Enriched gene sets x1

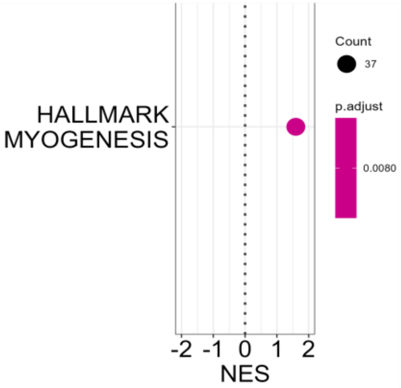

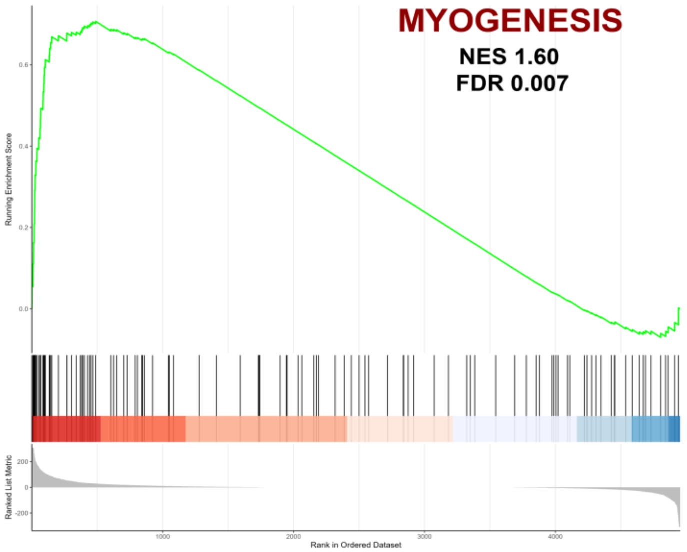

Fig. S10 Cell Type DEG

Integrated | D19 | CM/UNK-B | 4,591 cells

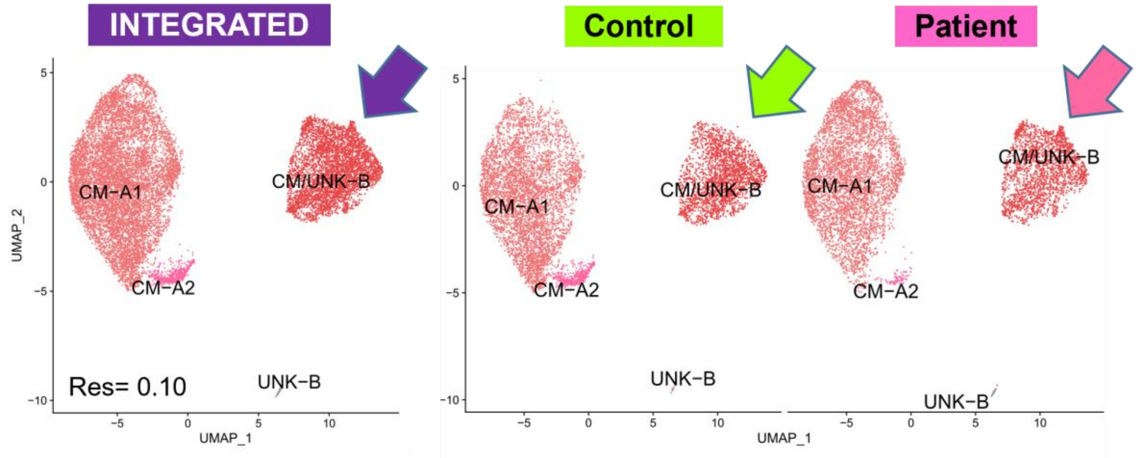

CM/UNK-B | D19: 4,591 cells | Patient (48%) vs Control (52%)

Under-Expressed: 8 genes | **47 DEGs** | **Over-Expressed: 39 genes**

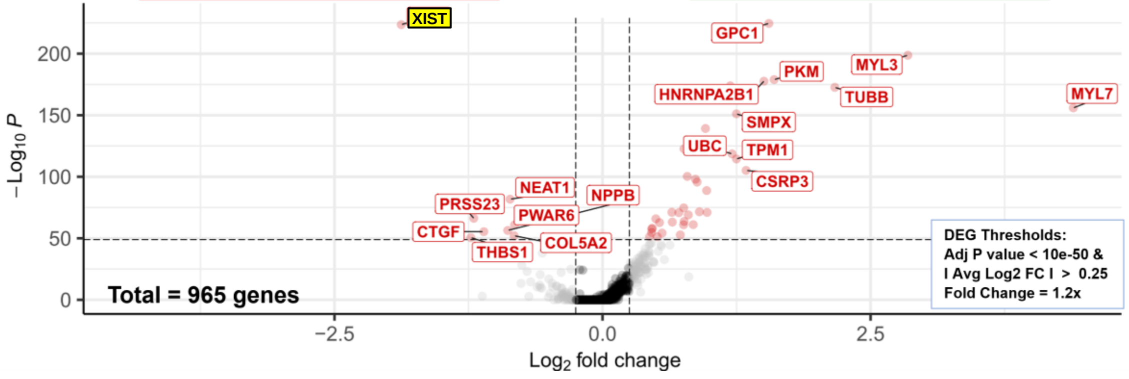

Over-Representation Analysis (ORA)

Under-Expressed: 8 genes

Enriched Gene Sets = 5

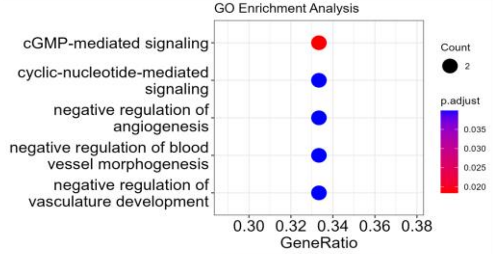

Enrichment Map Gene Sets = 5

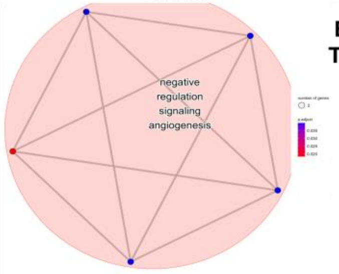

Genesx2
NPPB
THBS1

Over- Expressed: 39 genes

Enriched Gene Sets = 72

Top 5

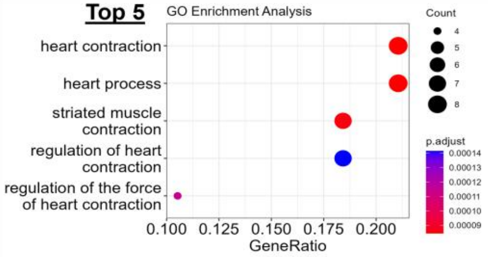

Enrichment Map Top 25 Gene Sets

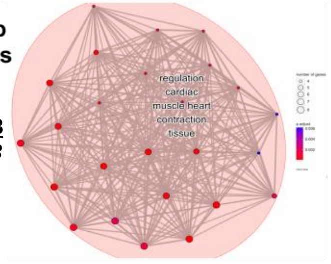

Genesx4-8
e.g., MYL3
MYL7
TPM1
CSRP3

Gene Set Enrichment Analysis (GSEA)

All 965 DEGs
Under-expressed: 395 genes
Over-expressed: 570 genes

No Enriched MSigDB Hallmark Gene Sets

Fig. S10 Cell Type DEG

Integrated | D19 | EPDC | 2,258 cells

INTEGRATED **Control** **Patient**

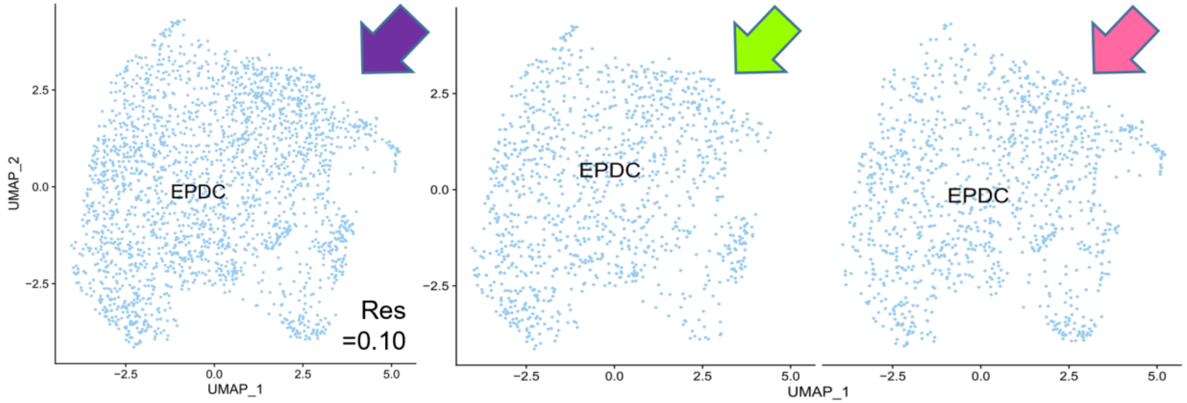

EPDC | D19: 2,258 cells | Patient (50%) vs Control (50%)

Under-Expressed: 5 genes **22 DEGs** **Over-Expressed: 17 genes**

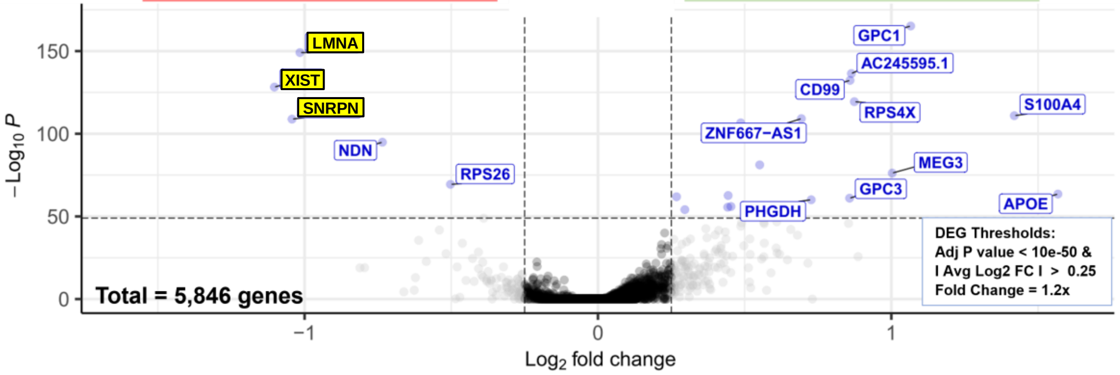

Over-Representation Analysis (ORA)

Under-Expressed: 5 genes

Enriched Gene Sets = 34

Top 5

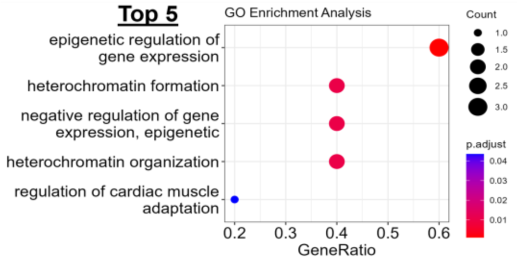

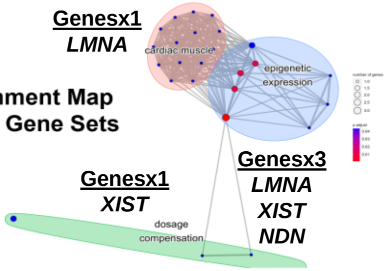

Over- Expressed: 17 genes

Enriched Gene Sets = 0

No Enrichment:
GO Biological Process
Gene Sets

Gene Set Enrichment Analysis (GSEA)

All 5846 DEGs
Under-expressed: 3084 genes
Over-expressed: 2762 genes

No Enriched MSigDB Hallmark Gene Sets

D. Cell Type DEG: *LMNA*, X-Linked Genes, and Imprinted Genes Across 14 Cell Subtypes

Integrated | **'Balanced' Paired Data Subsets (n= 6 Pairs)** | **75,330 cells**

Lamin A/C (LMNA)
Underexpressed
Day 19 Patient Cells

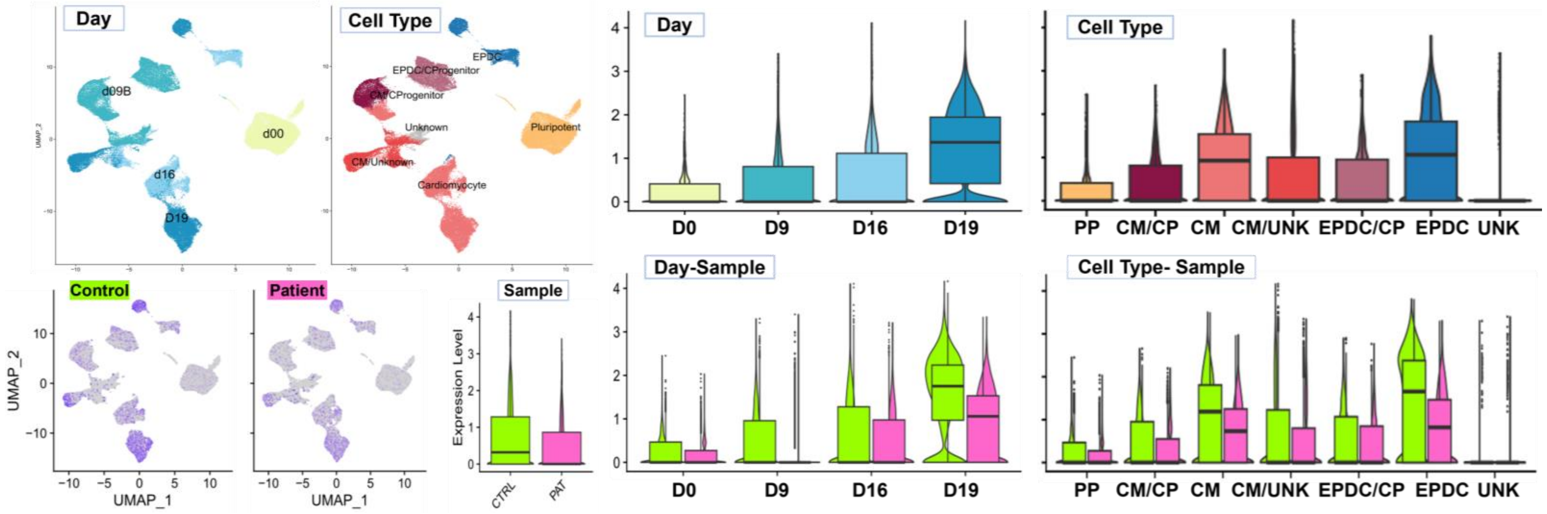

Lamin B1

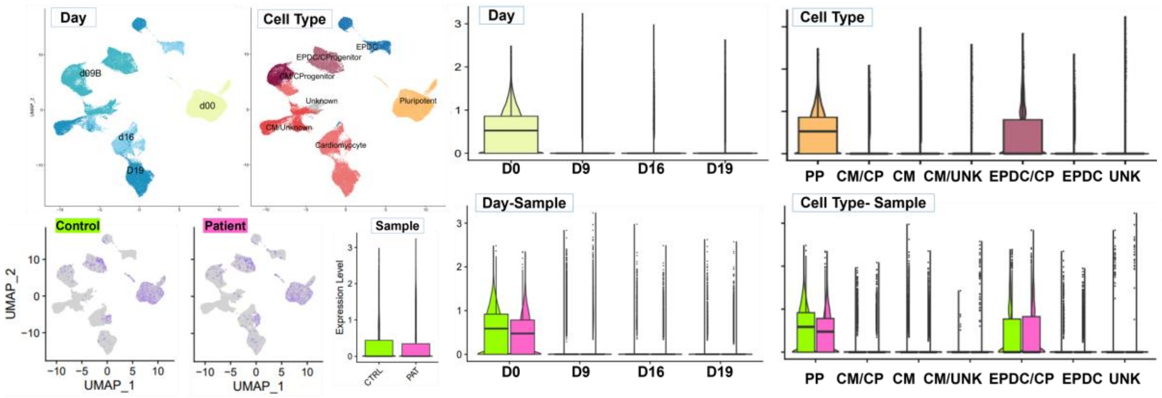

Lamin B2

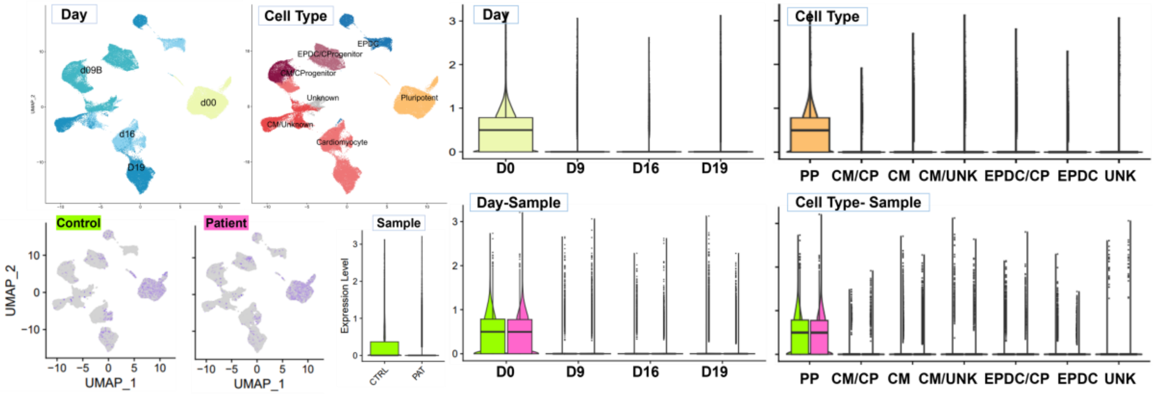

Fig. S10 Cell Type DEG

X Inactive Specific Transcript (XIST)
Underexpressed
All Patient Cells

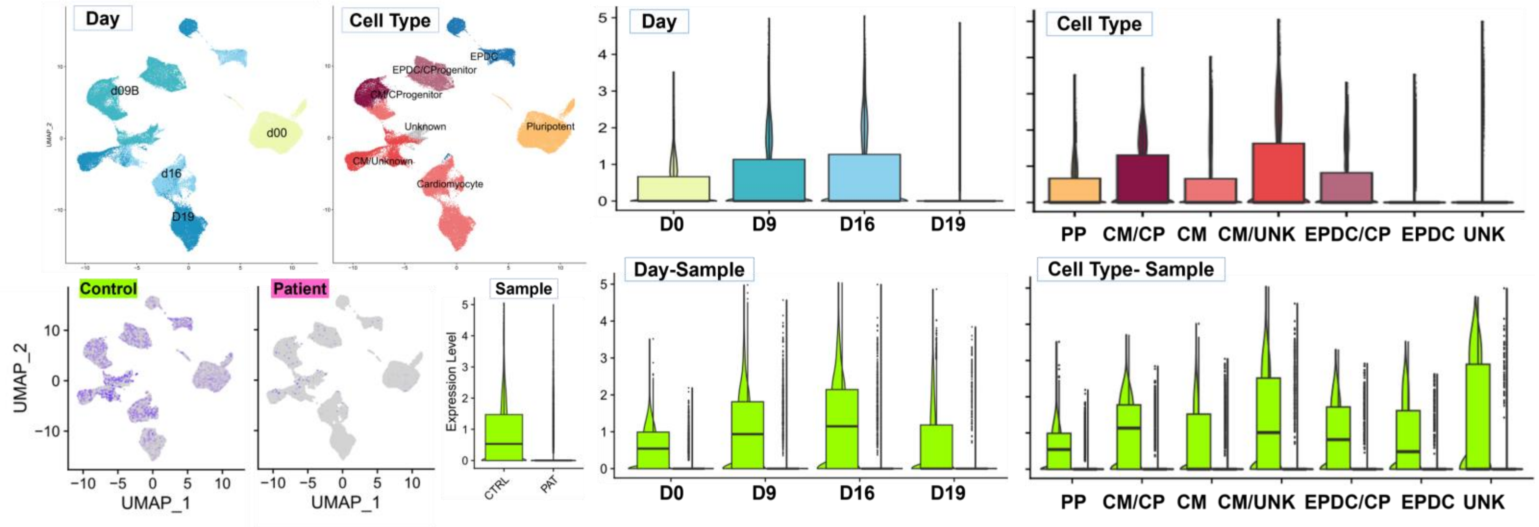

X-Linked Glypican-3 (GPC3)
Overexpressed
Day 9 Patient Cells

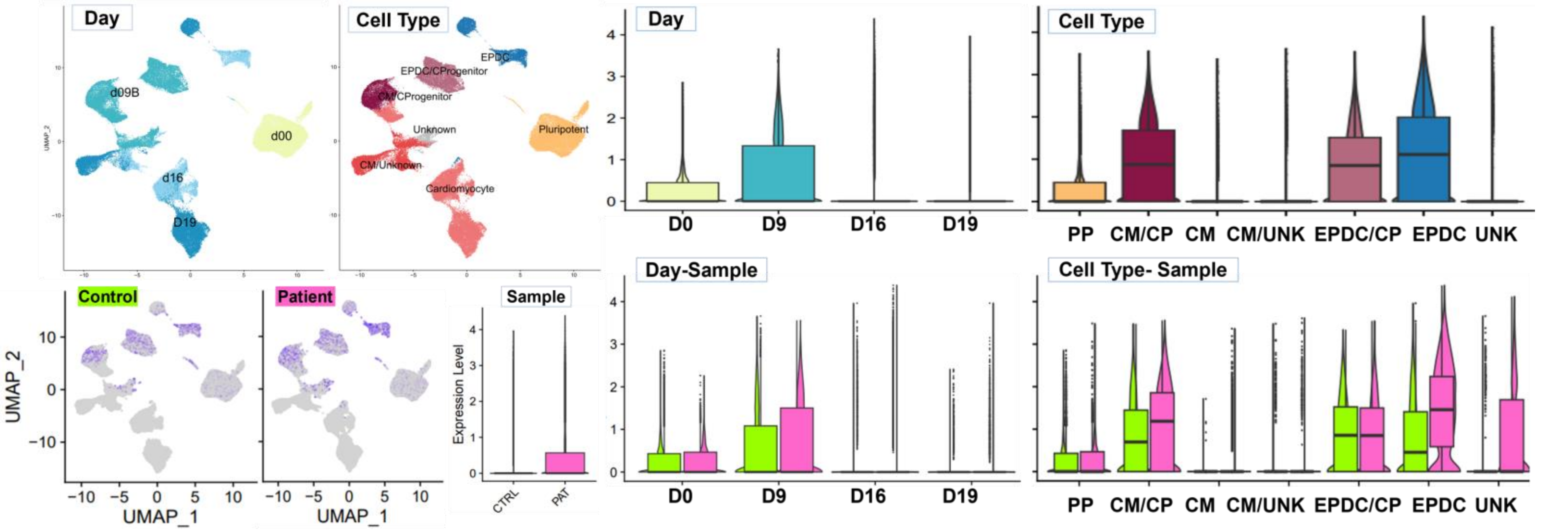

Fig. S10 Cell Type DEG

Imprinted (PAT)
SNRPN
chr15q11.2
Underexpressed
All Patient Cells

Imprinted (MAT)
MEG3
chr14q32.2
Overexpressed
Pluripotent (PP) &
EPDC Patient Cells

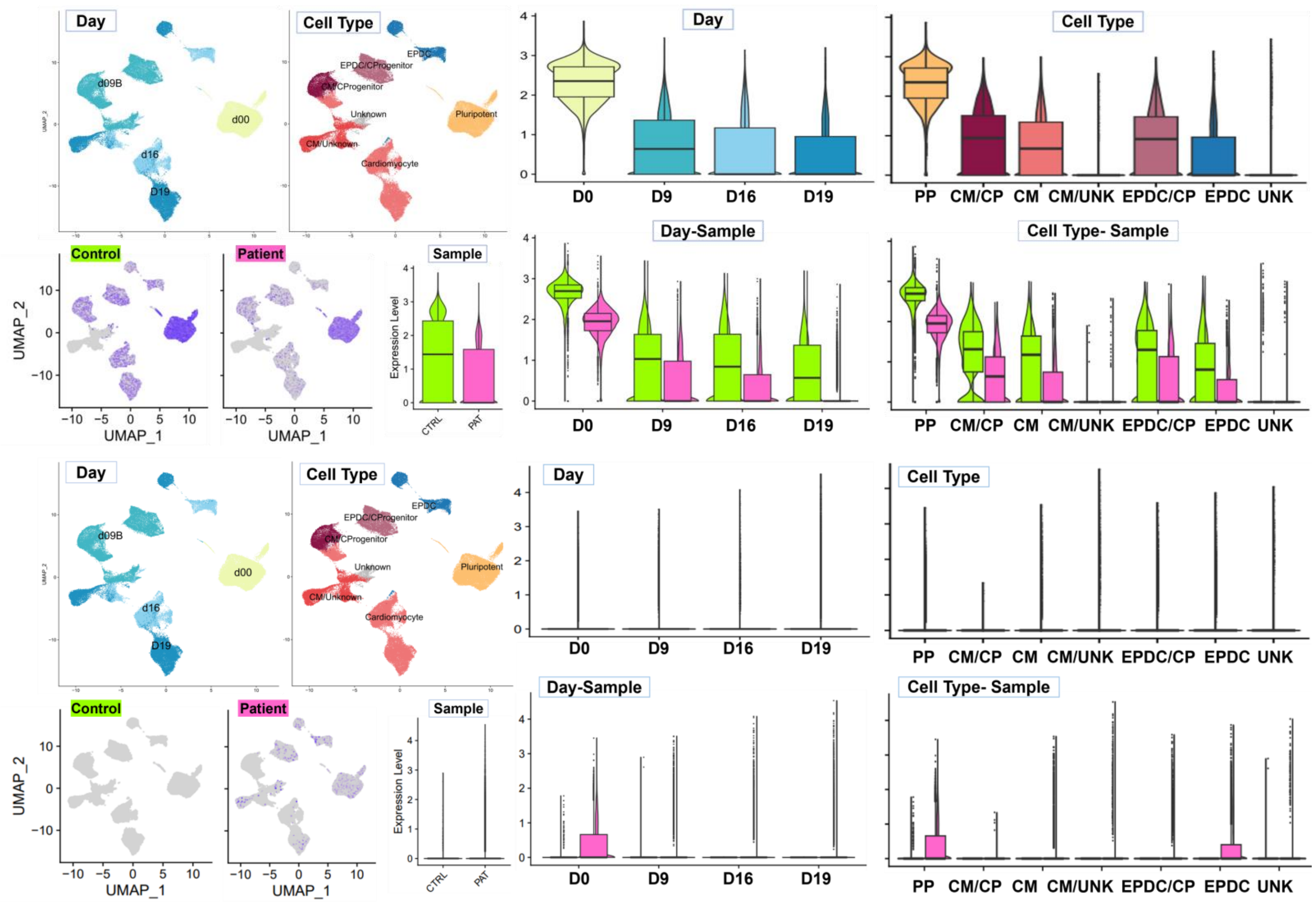

Fig. S10 Cell Type DEG

X-linked *PIN4*
Underexpressed Day 00 Patient Cells

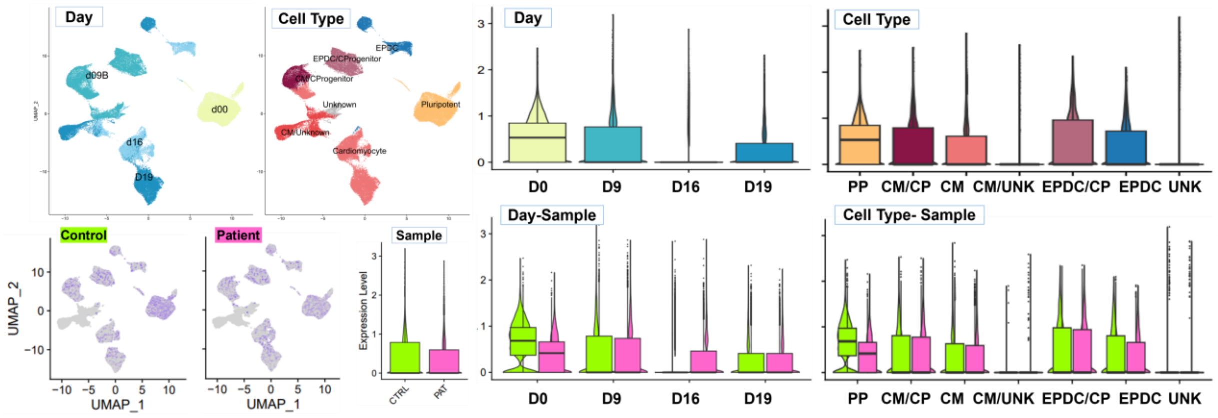

Imprinted (PAT) *NDN* at chr15q11.2
Underexpressed in Patient Cells

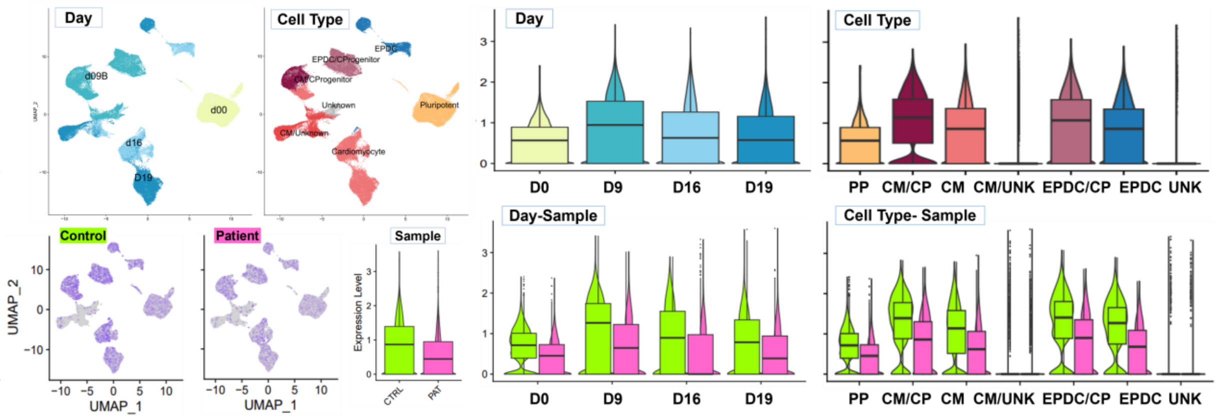

Imprinted (PAT) *PWAR6* at chr15q11.2
Underexpressed in Patient Cells

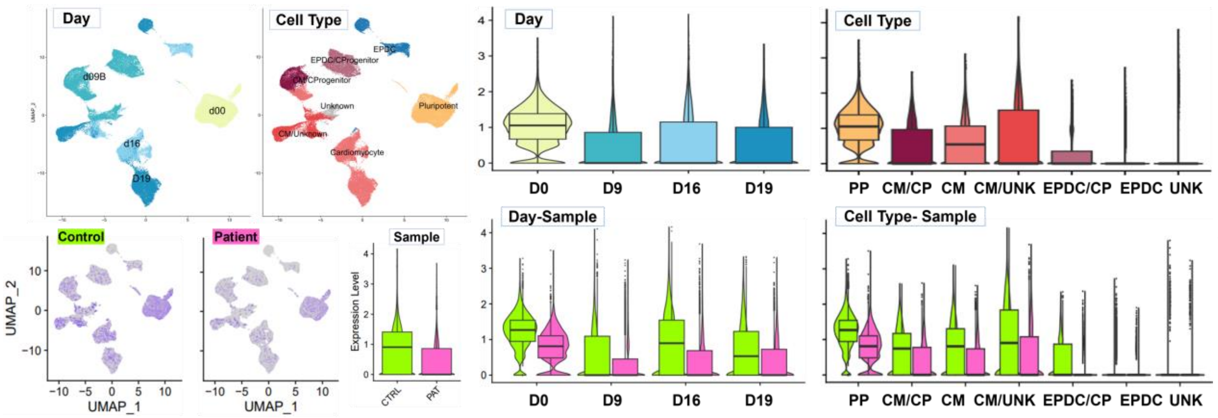

Imprinted (PAT) *PEG10* at chr7q21.3
Underexpressed in Day 16 CM Patient Cells

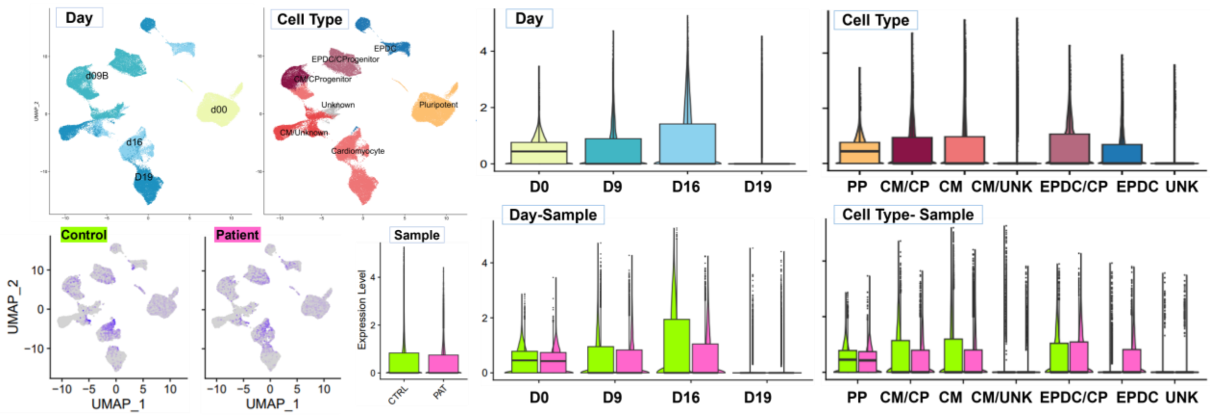

Fig. S10 Cell Type DEG

E. Cell Type DEG Enrichment: Module Scoring of GSEA Significant Gene Sets

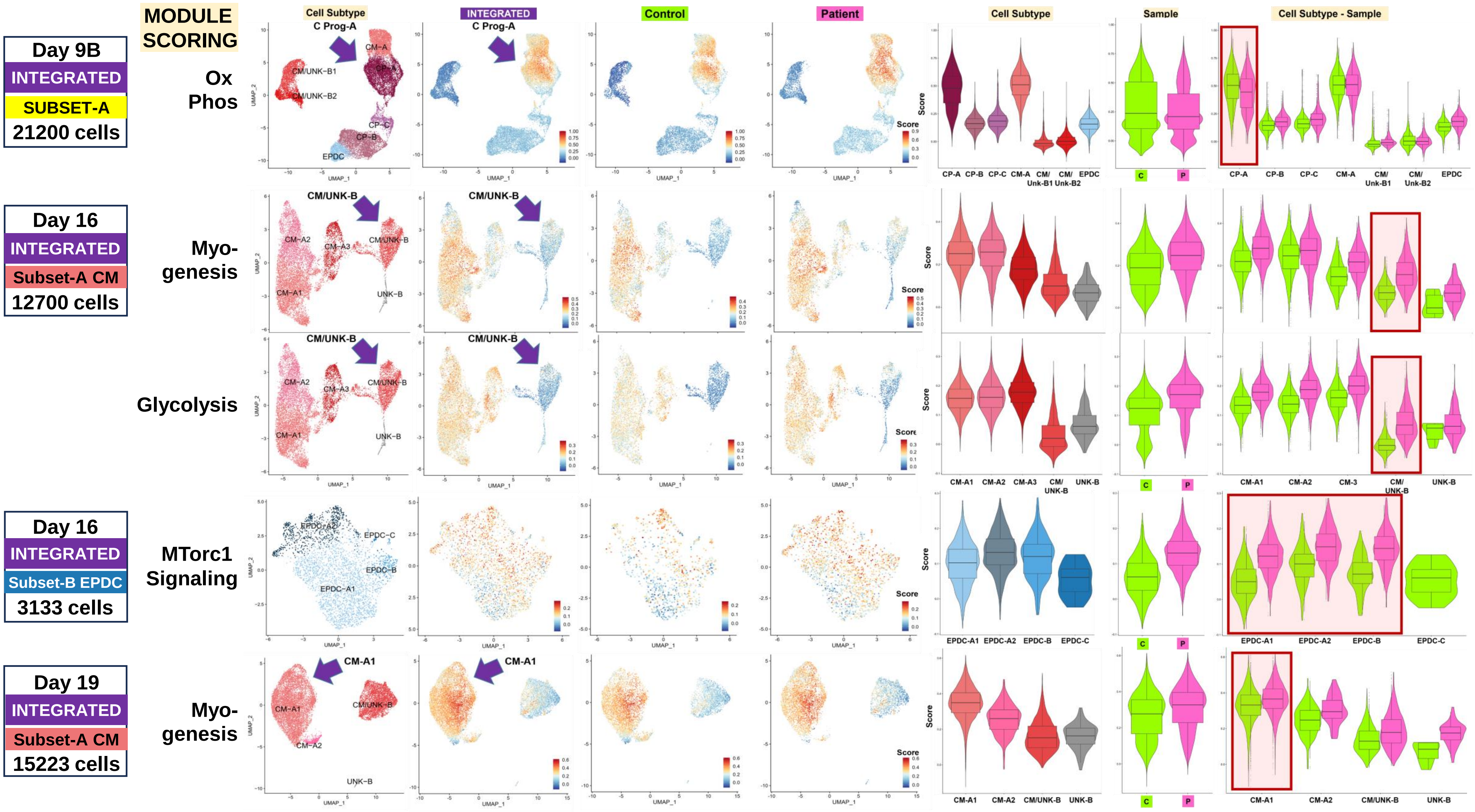

Fig. S10 Cell Type DEG

Day 16
INTEGRATED
CM Subset
12700 cells

MODULE SCORING

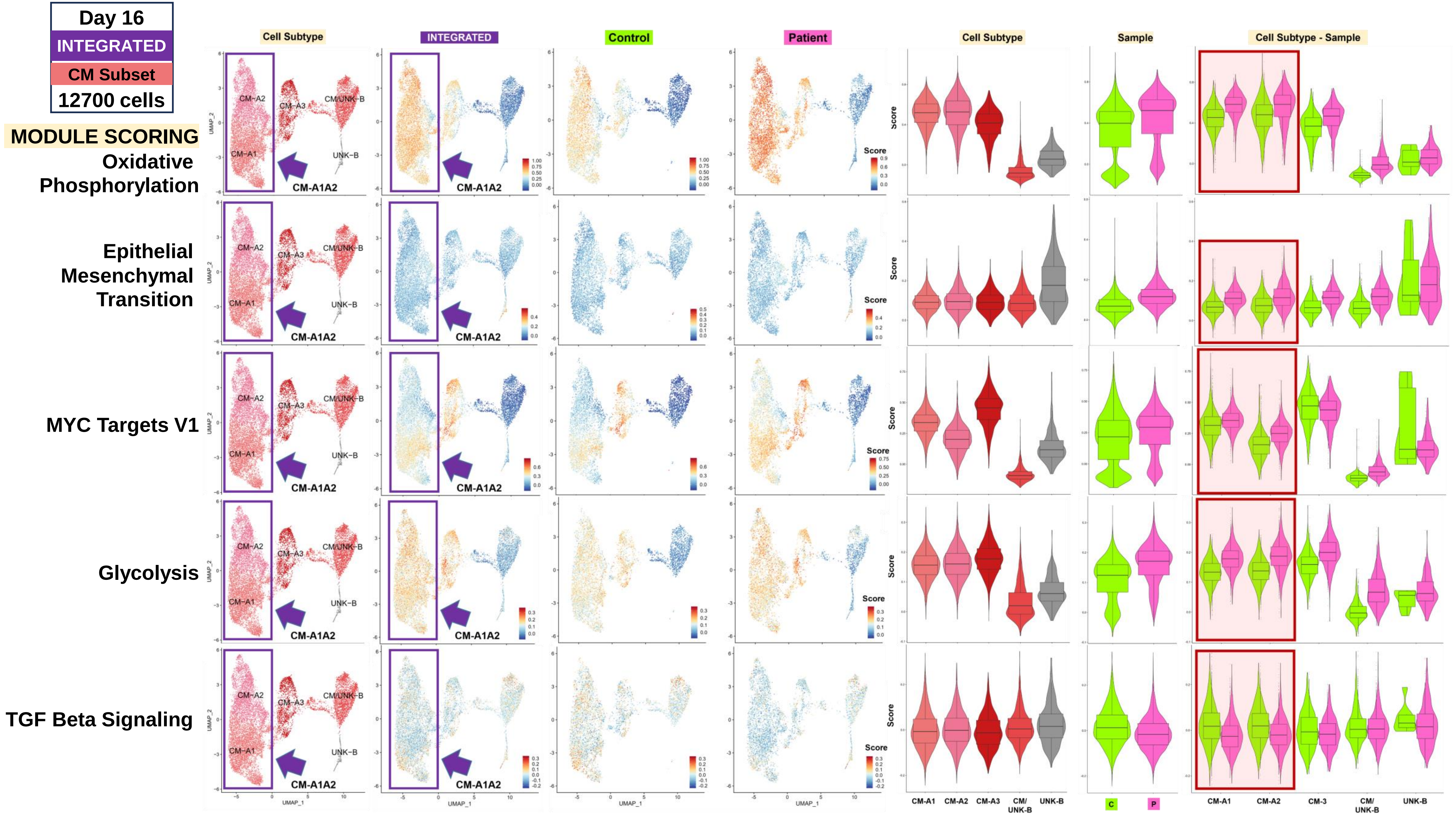

Fig. S10 Cell Type DEG

Integrated ‘Balanced’ Paired Data Subsets (n= 6 Pairs) **75,330 cells**

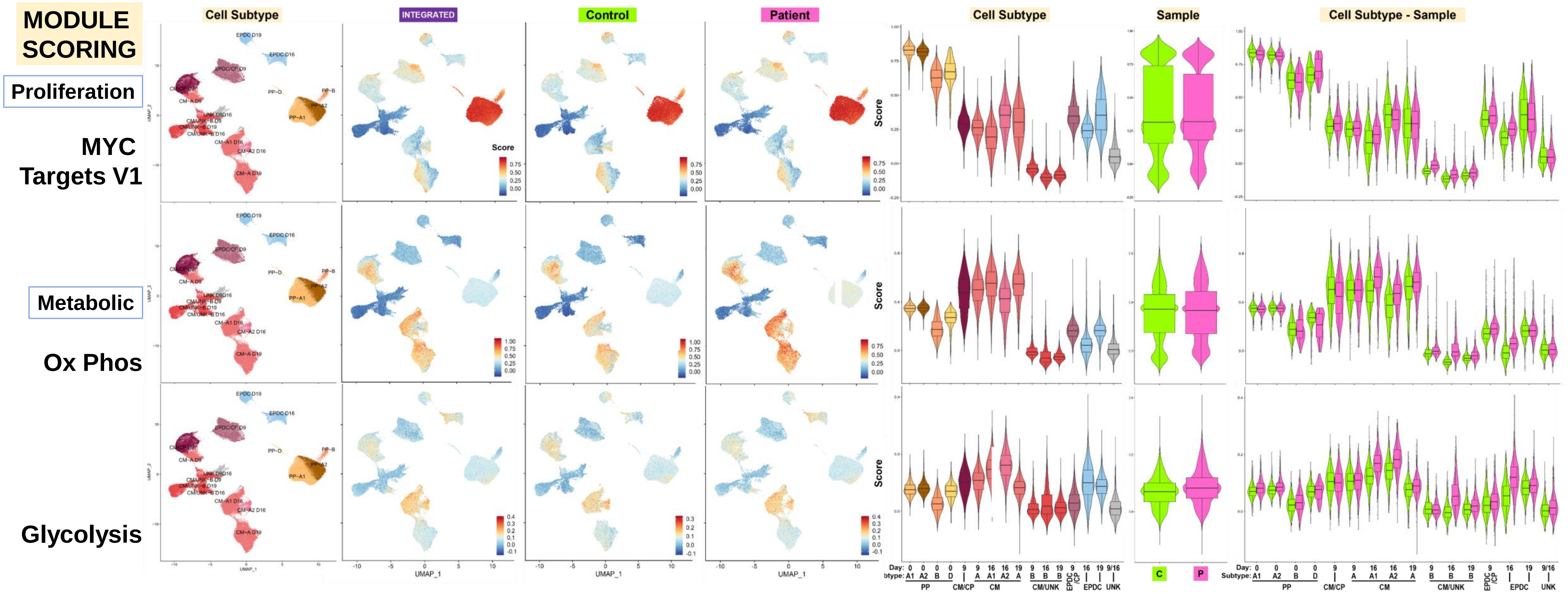

Fig. S10 Cell Type DEG

Integrated ‘Balanced’ Paired Data Subsets (n= 6 Pairs) 75,330 cells

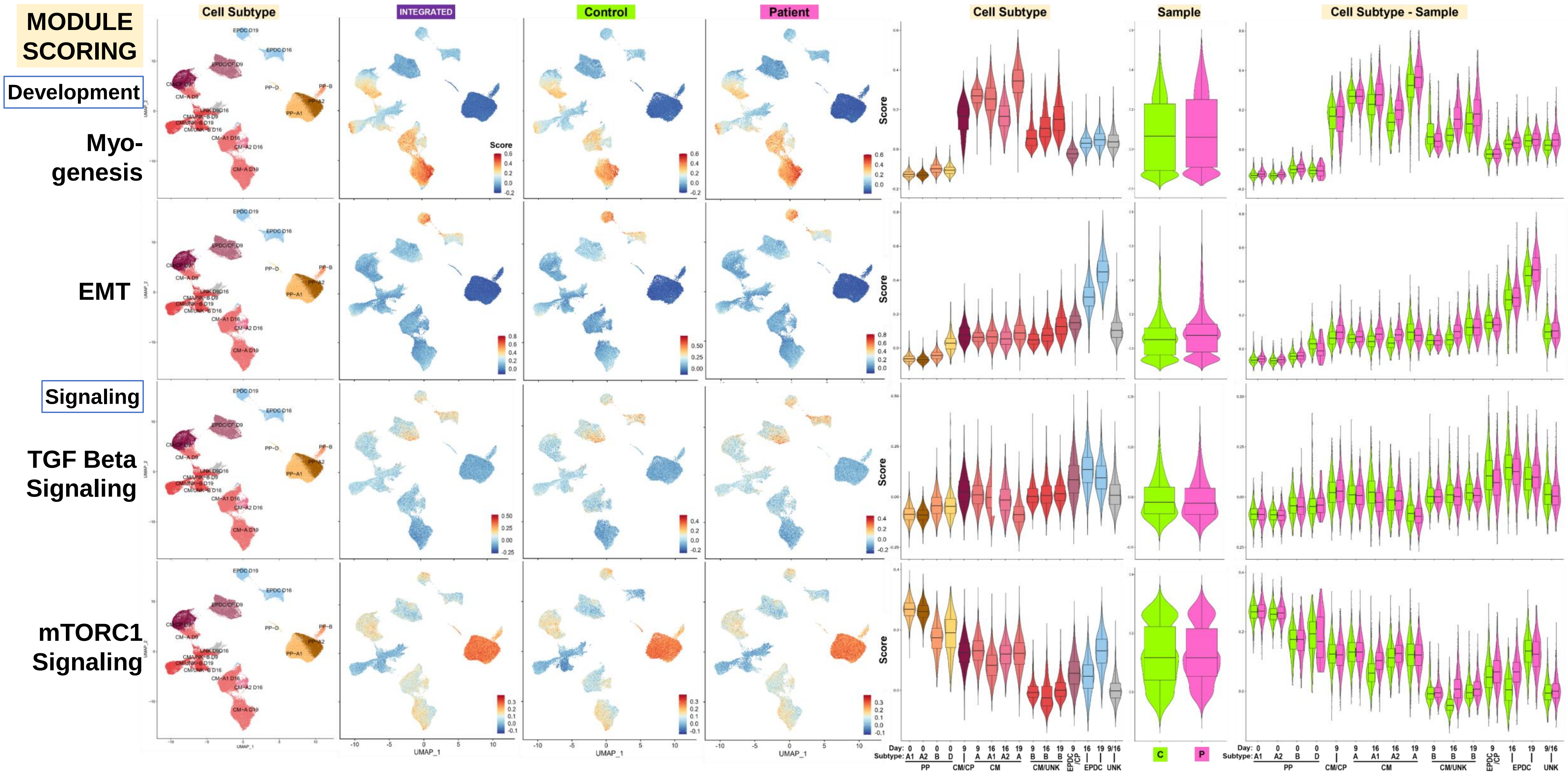

Fig. S11
Lineage DEG SS

Workflow Step-III: Single Subset Data Results

Trajectory Analyses for Lineage-Specific Differential Expression and Enrichment

A. Summary: Annotated Subset Data to Cell Lineages

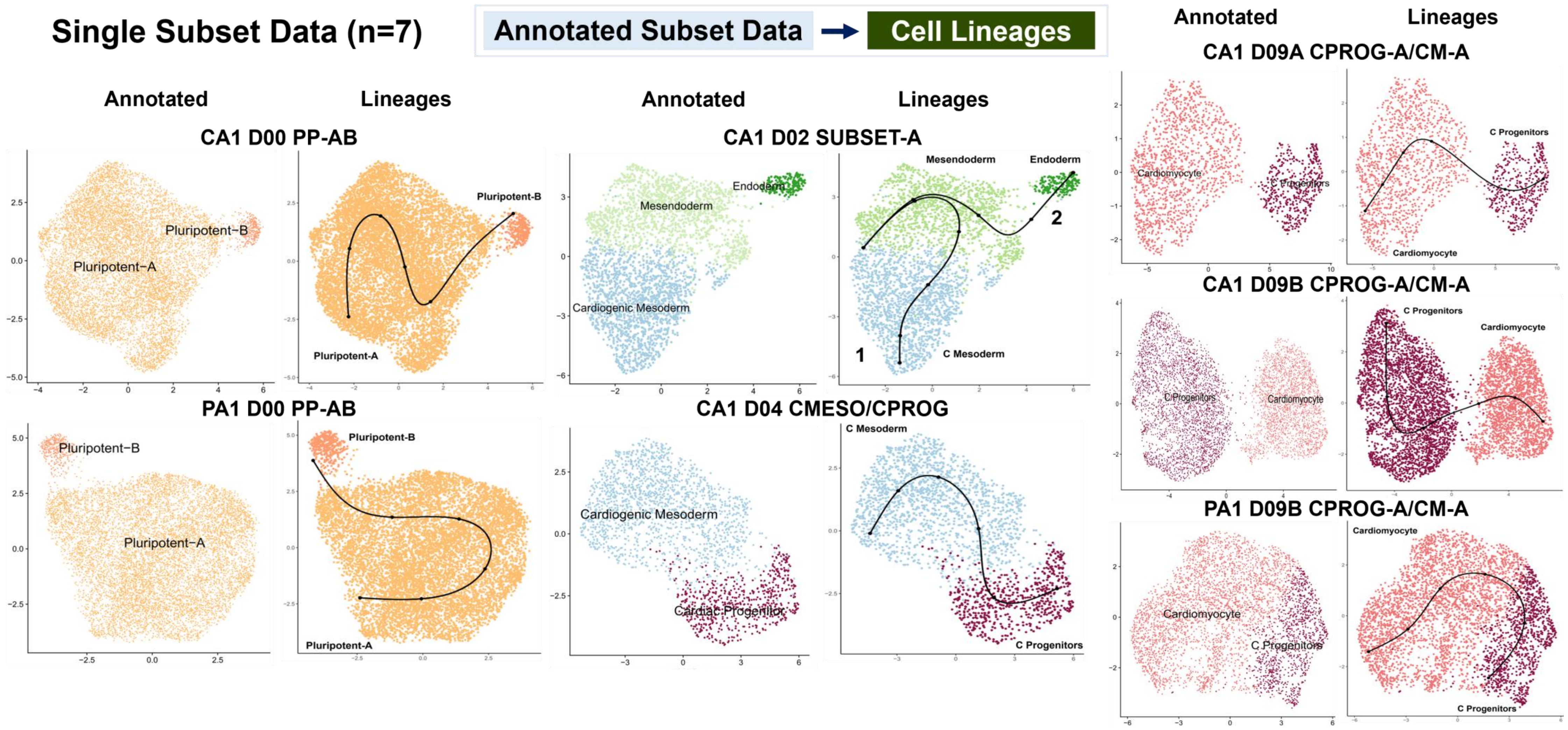

Fig. S11
Lineage DEG SS

B. Single Subset Data: Trajectory, Lineage DEG, and Enrichment Analyses (n=7)

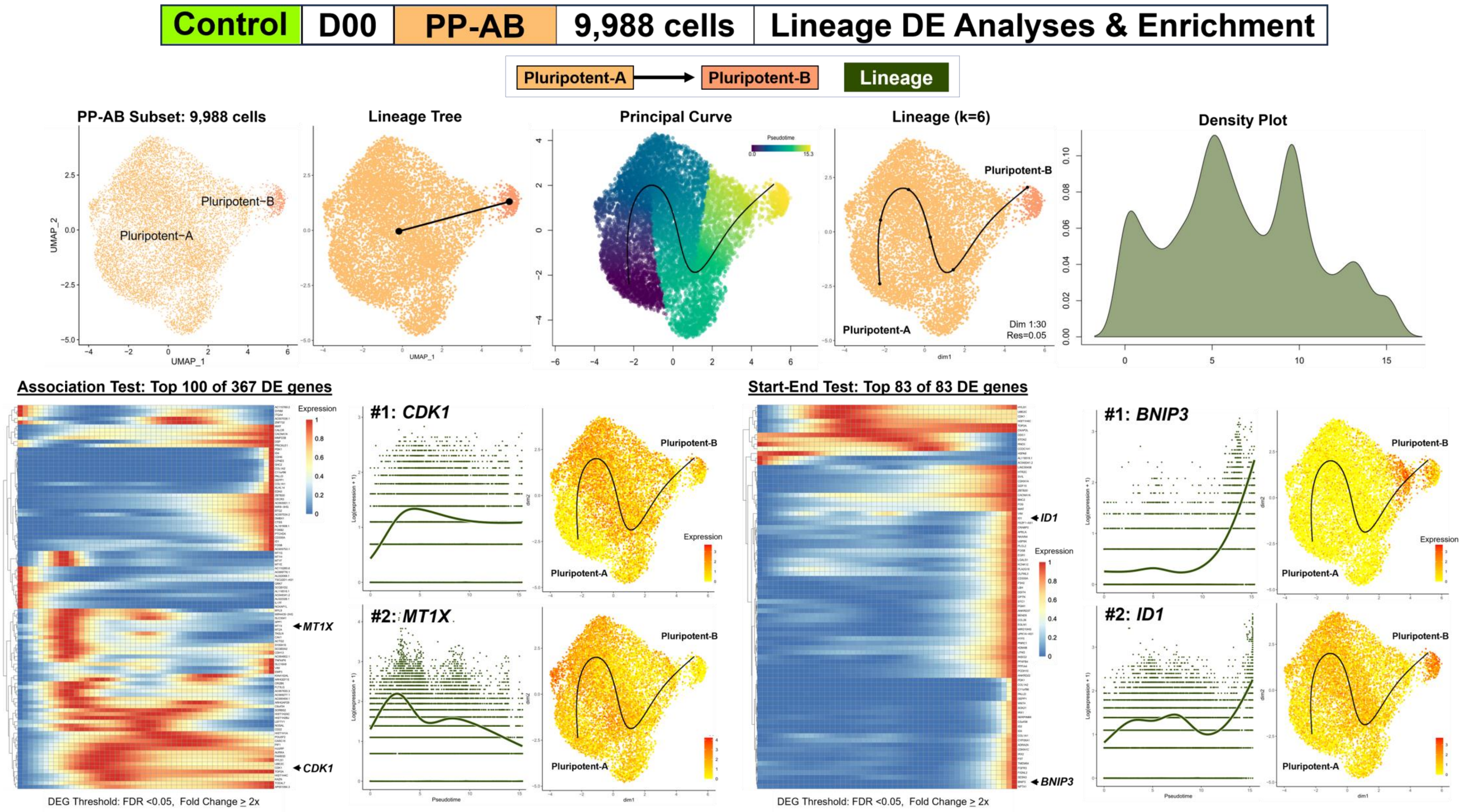

Fig. S11
Lineage DEG SS

Control	D00	PP-AB	9,988 cells	Lineage DE Analyses & Enrichment

Over-Representation Analysis

Pluripotent-A → Pluripotent-B | Lineage

Top 100 of 367 Association DEG

Enriched Gene Sets = 36

Top 83 of 83 Start-End DEG

Enriched Gene Sets = 45

Enrichment Plots: Top 5 Gene Sets

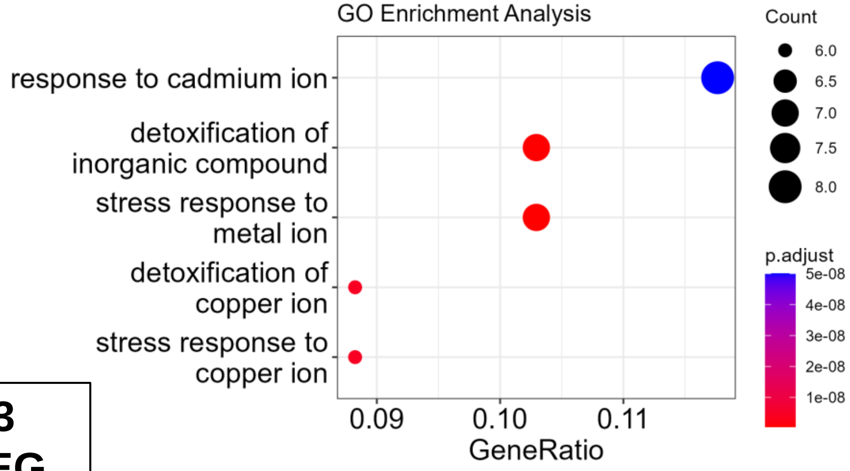

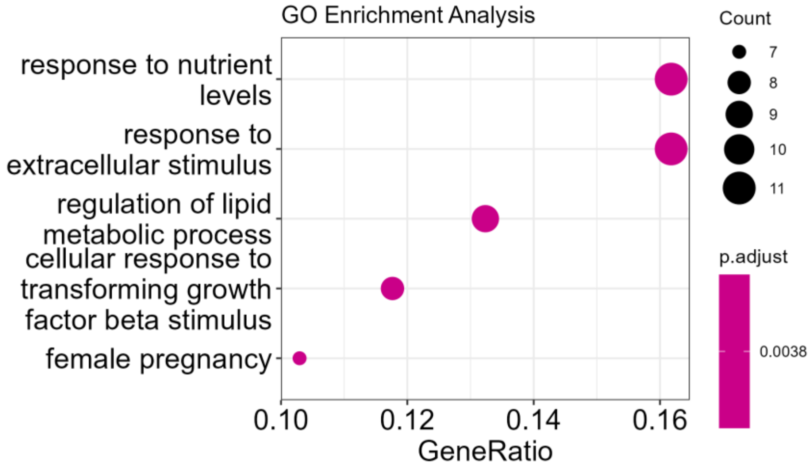

Enrichment Maps Top 25 Gene Sets

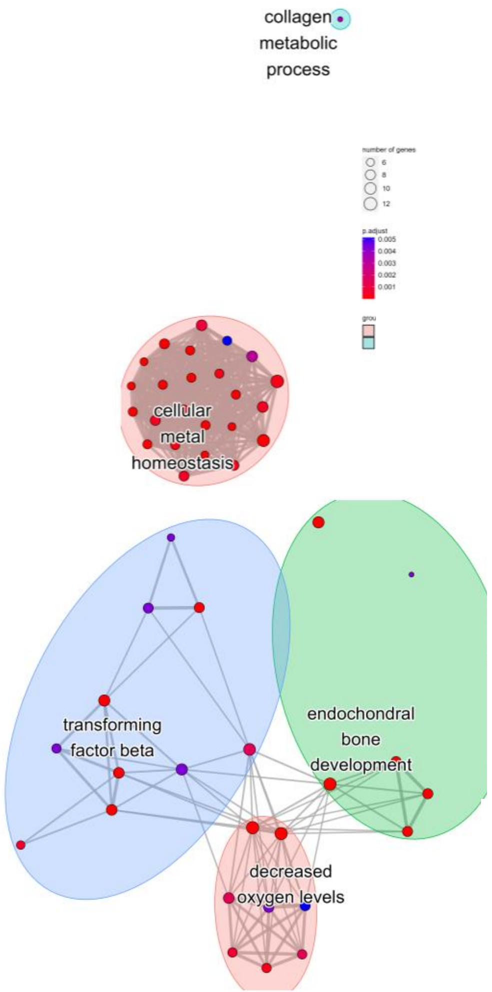

Enrichment Trees Top 25 Gene Sets

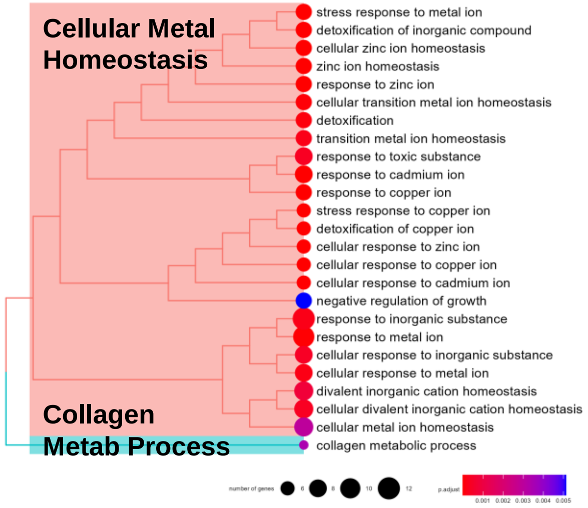

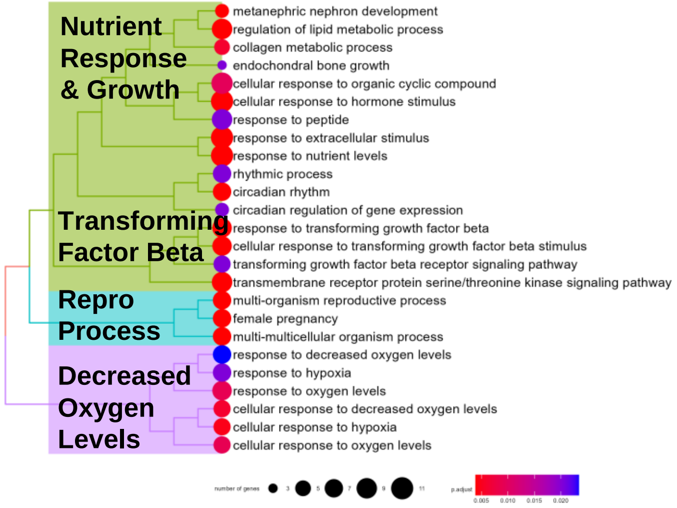

Fig. S11
Lineage DEG SS

Patient	D00	PP-AB	9,358 cells	Lineage DE Analyses & Enrichment

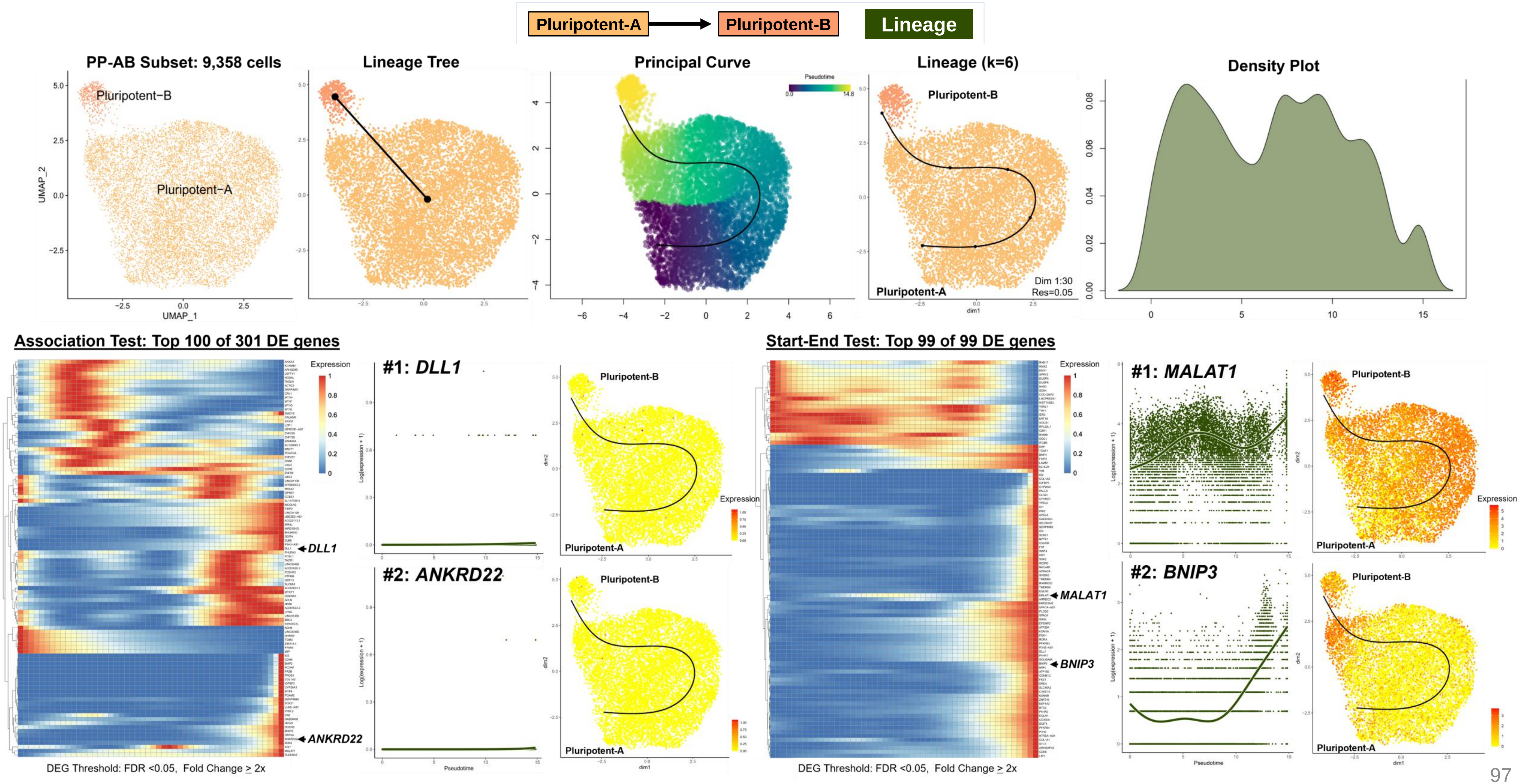

Fig. S11
Lineage DEG SS

Patient	D00	PP-AB	9,358 cells	Lineage DE Analyses & Enrichment

Over-Representation Analysis

Pluripotent-A → Pluripotent-B | Lineage

Enrichment Plots: Top 5 Gene Sets | **Enrichment Maps Top 25 Gene Sets** | **Enrichment Trees Top 25 Gene Sets**

Top 100 of 301 Association DEG

Enriched Gene Sets = 174

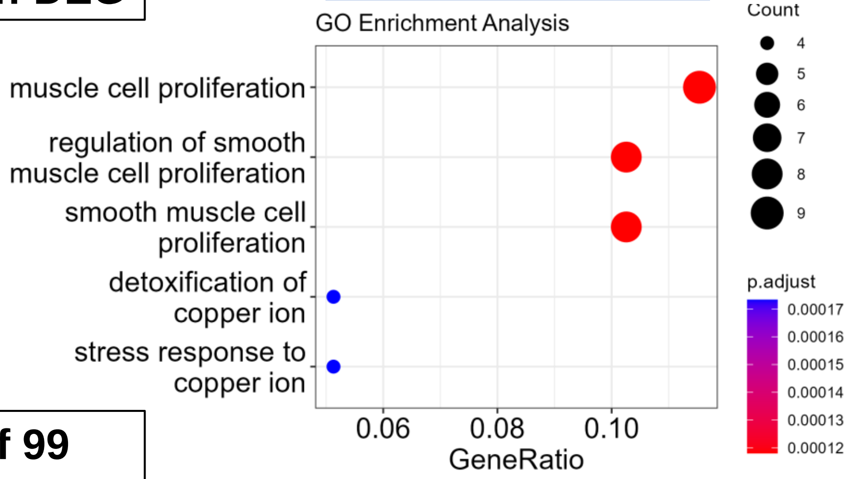

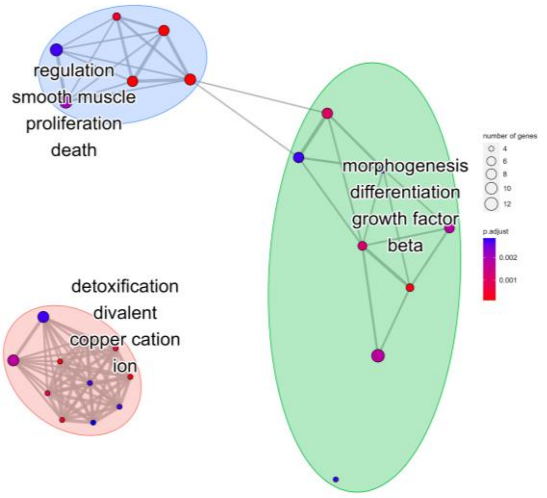

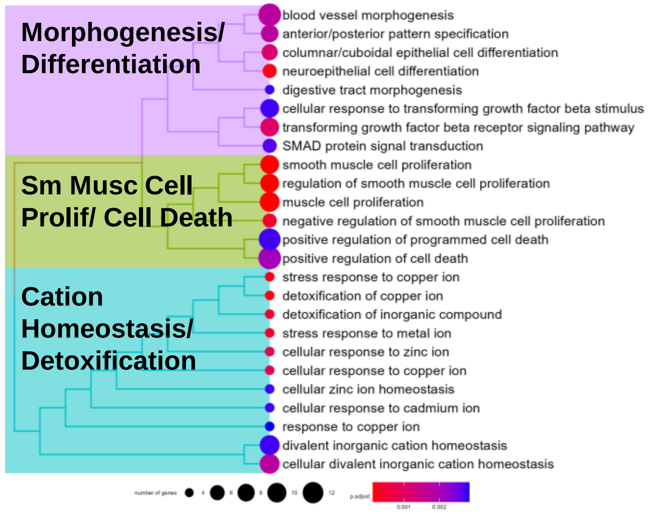

Top 99 of 99 Start-End DEG

Enriched Gene Sets = 246

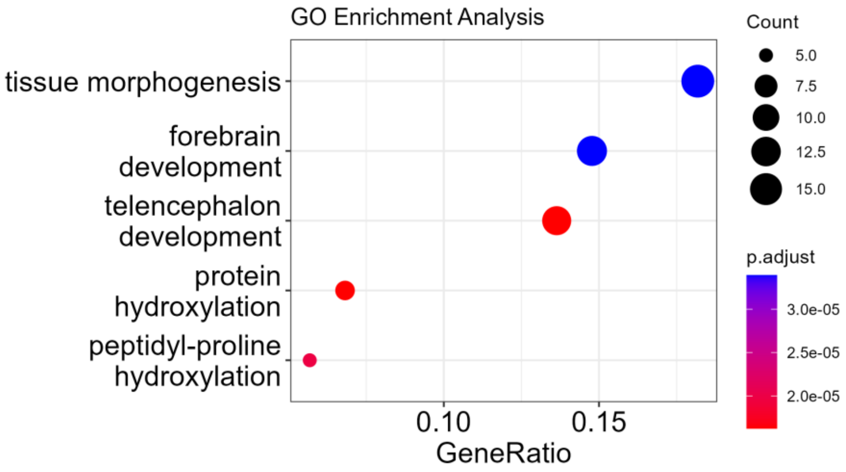

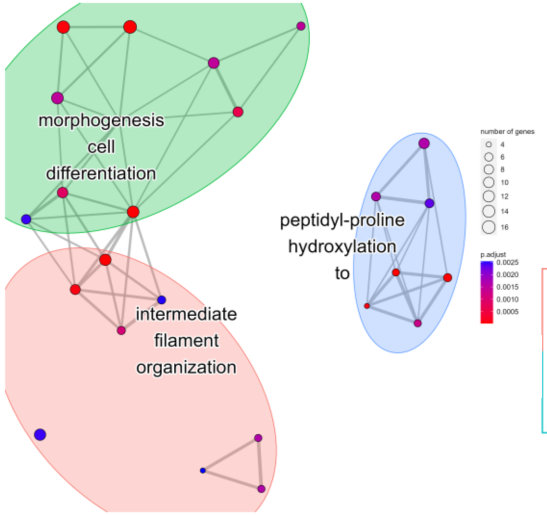

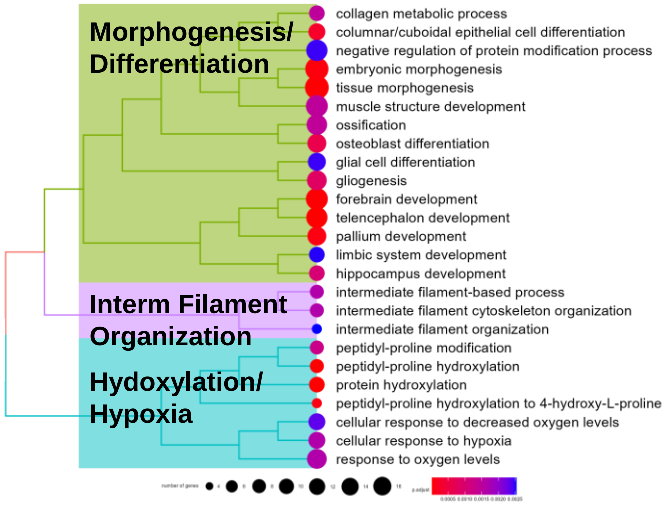

Fig. S11
Lineage DEG SS

Control	D02	ME/CMESO/ENDO	3,145 cells	Lineage DE Analyses & Enrichment

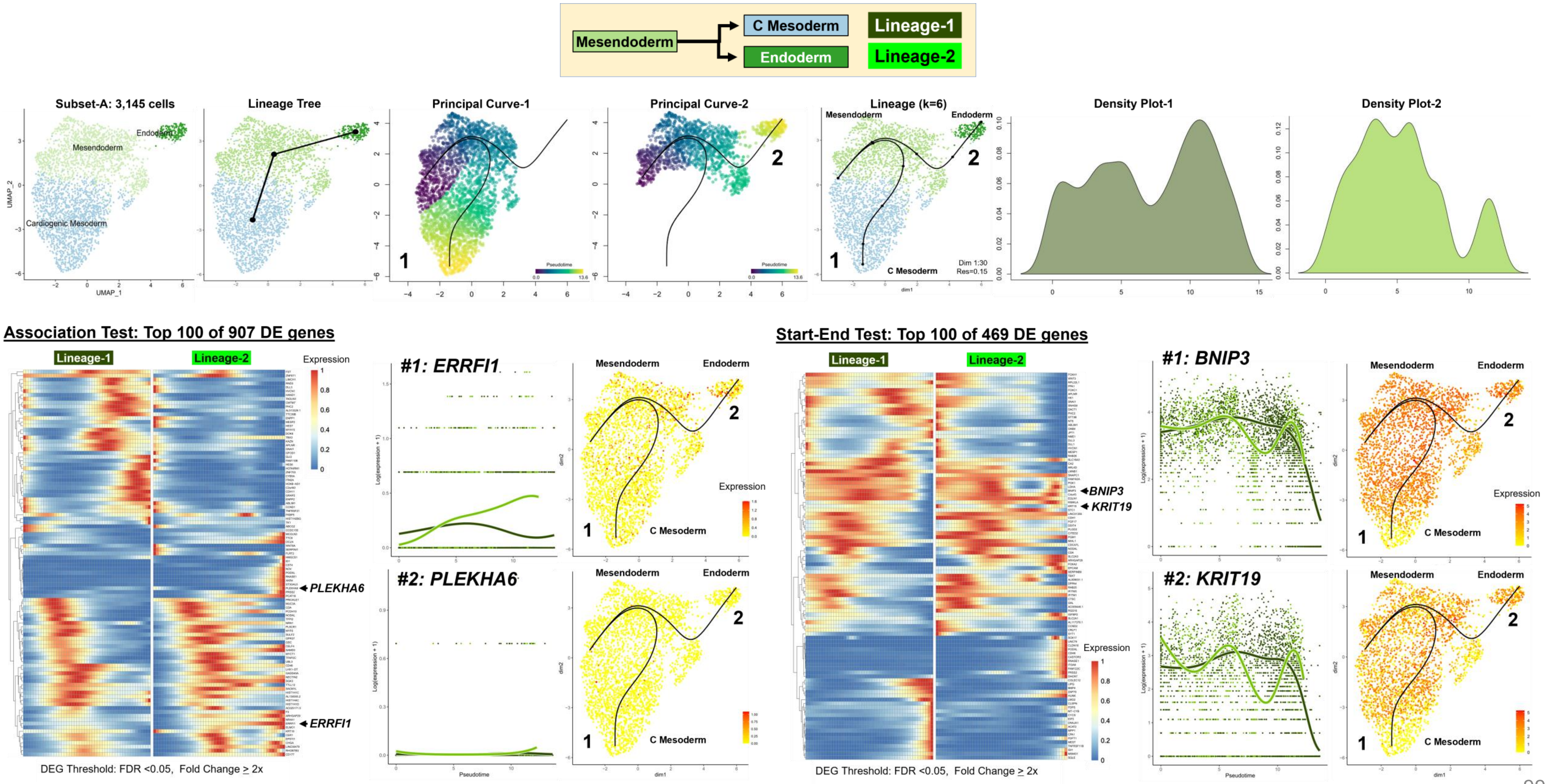

Fig. S11
Lineage DEG SS

Control	D02	ME/CMESO/ENDO	3,145 cells	Lineage DE Analyses & Enrichment

Over-Representation Analysis

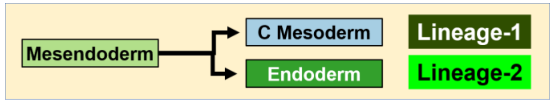

Enrichment Plots: Top 5 Gene Sets

Enrichment Maps Top 25 Gene Sets

Enrichment Trees Top 25 Gene Sets

Top 100 of 907 Association DEG

Enriched Gene Sets = 27

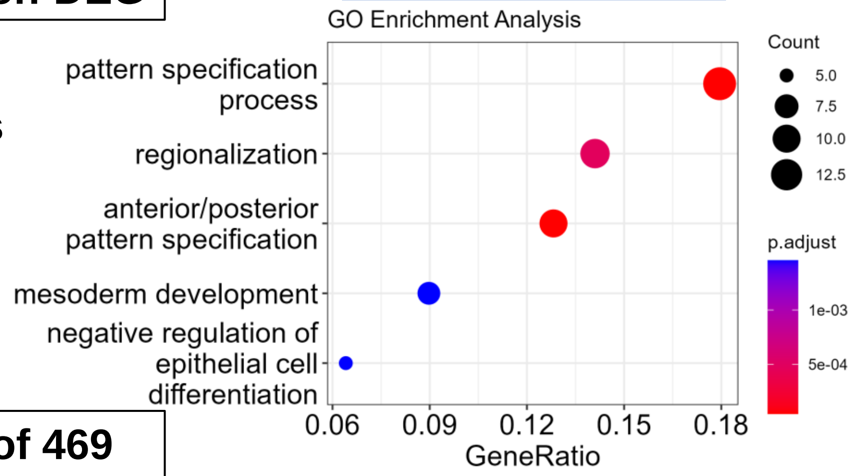

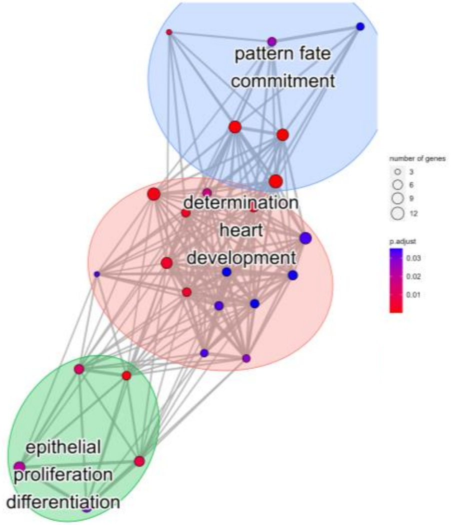

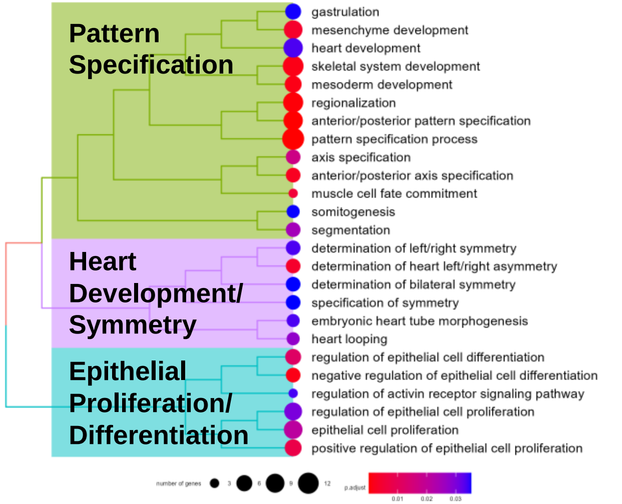

Top 100 of 469 Start-End DEG

Enriched Gene Sets = 187

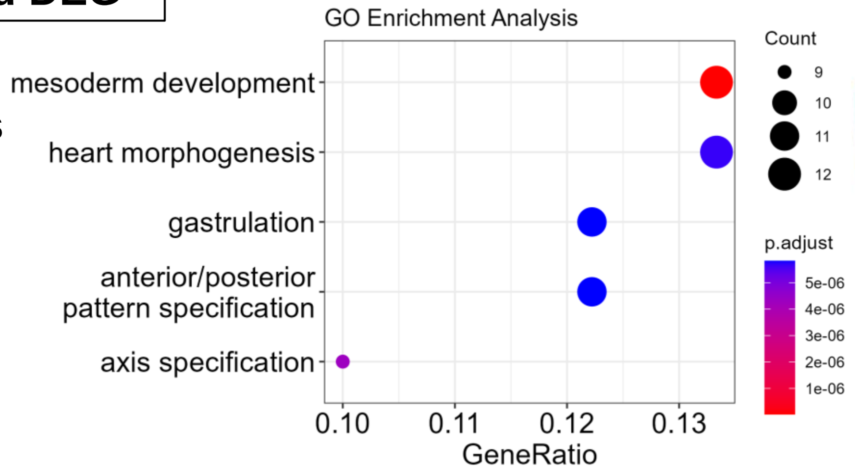

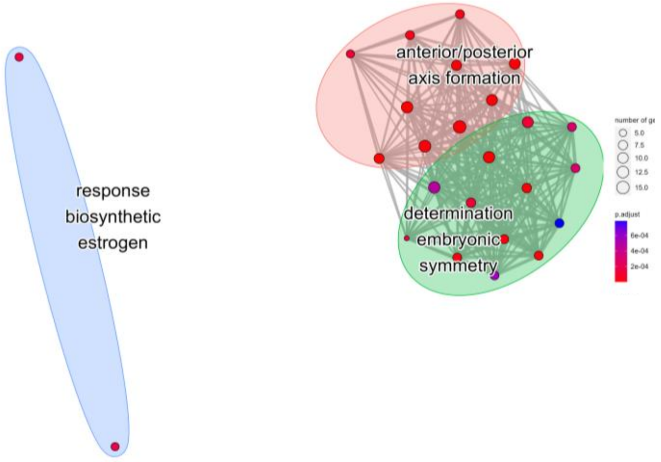

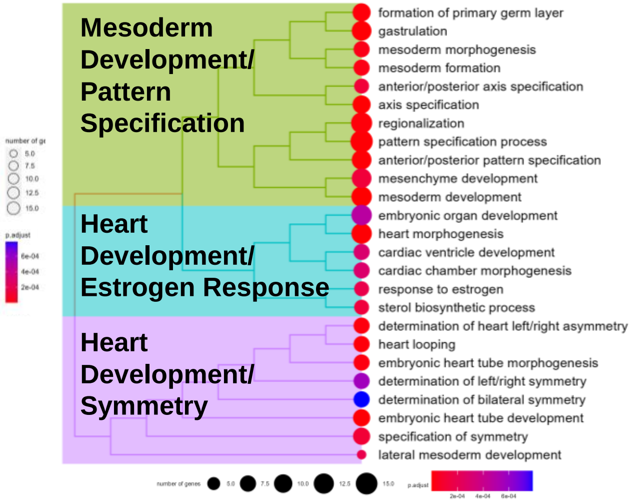

Fig. S11
Lineage DEG SS

Control	D04	CMESO/CPROG	2,229 cells	Lineage DE Analyses & Enrichment

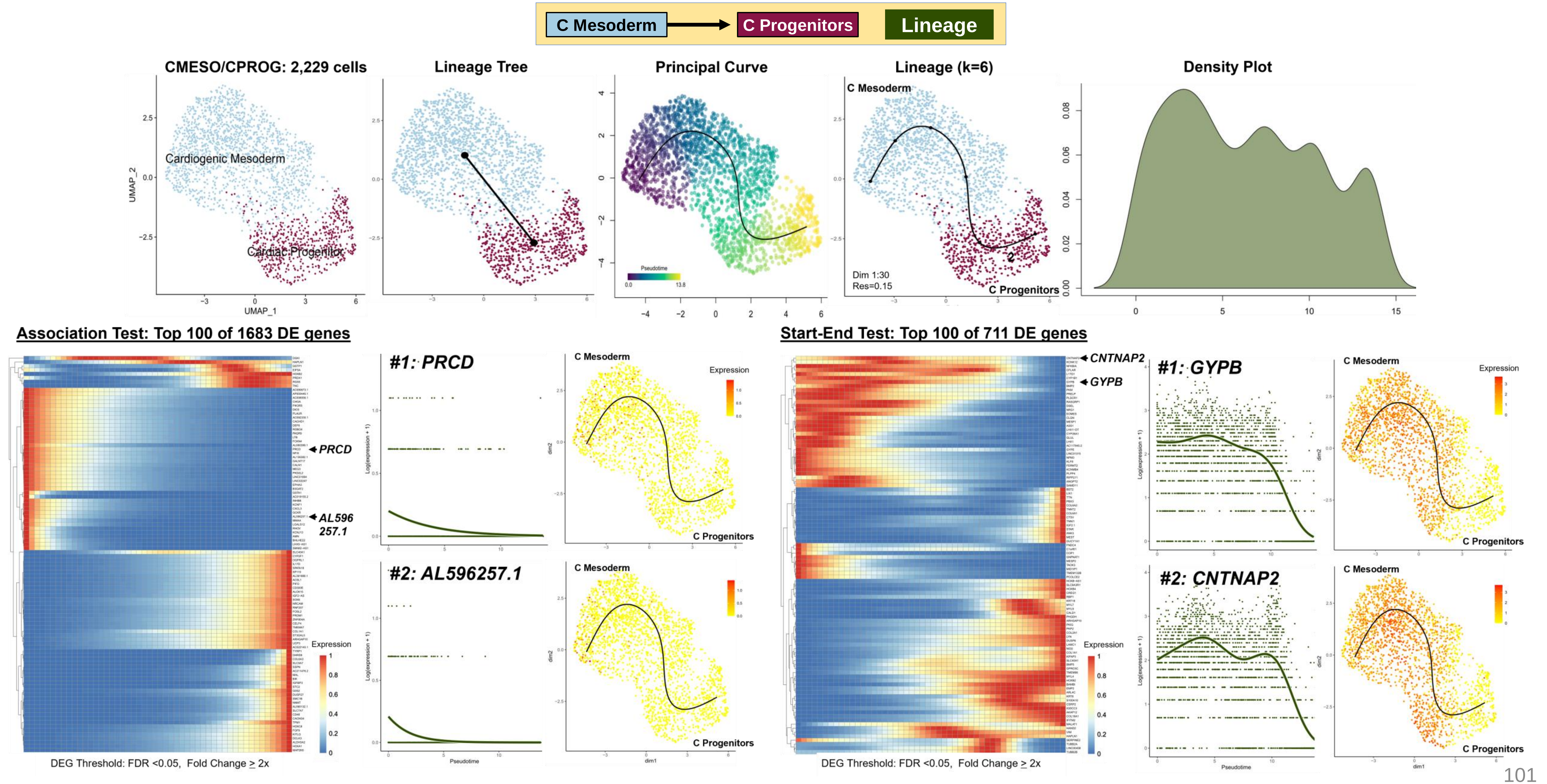

Fig. S11
Lineage DEG SS

Control	D04	CMESO/CPROG	2,229 cells	Lineage DE Analyses & Enrichment

Over-Representation Analysis

C Mesoderm → C Progenitors   Lineage

Top 100 of 1683 Association DEG

Enrichment Plots: Top 5 Gene Sets

Enrichment Maps Top 25 Gene Sets

Enrichment Trees Top 25 Gene Sets

Enriched Gene Sets = 0

No Enrichment: GO Biological Process Gene Sets

No Enrichment: GO Biological Process Gene Sets

No Enrichment: GO Biological Process Gene Sets

Top 100 of 711 Start-End DEG

Enriched Gene Sets = 114

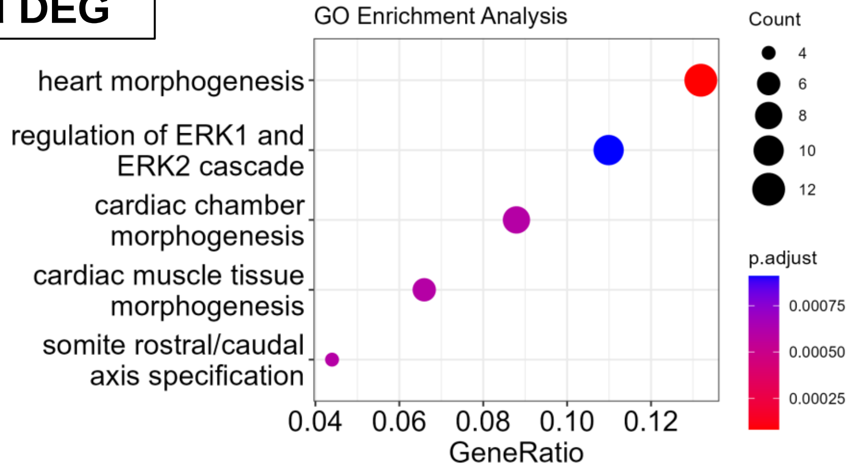

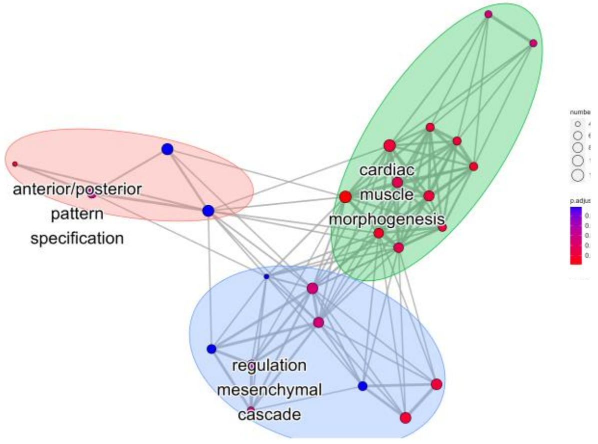

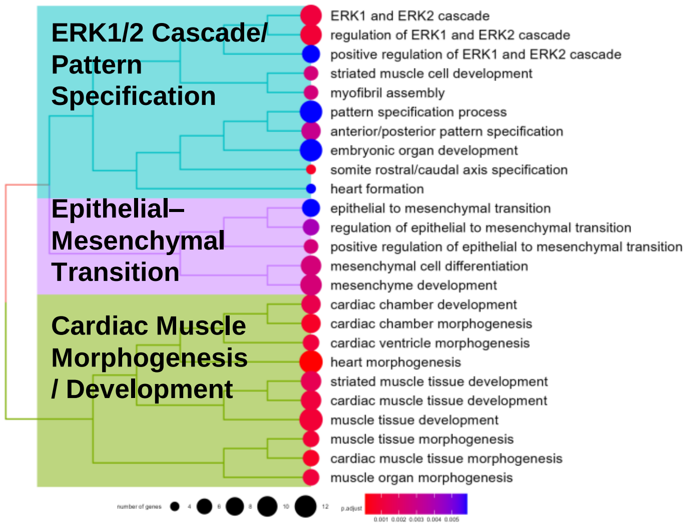

Fig. S11
Lineage DEG SS

Control	D09A	CPROG-A/CM-A	1,671 cells	Lineage DE Analyses & Enrichment

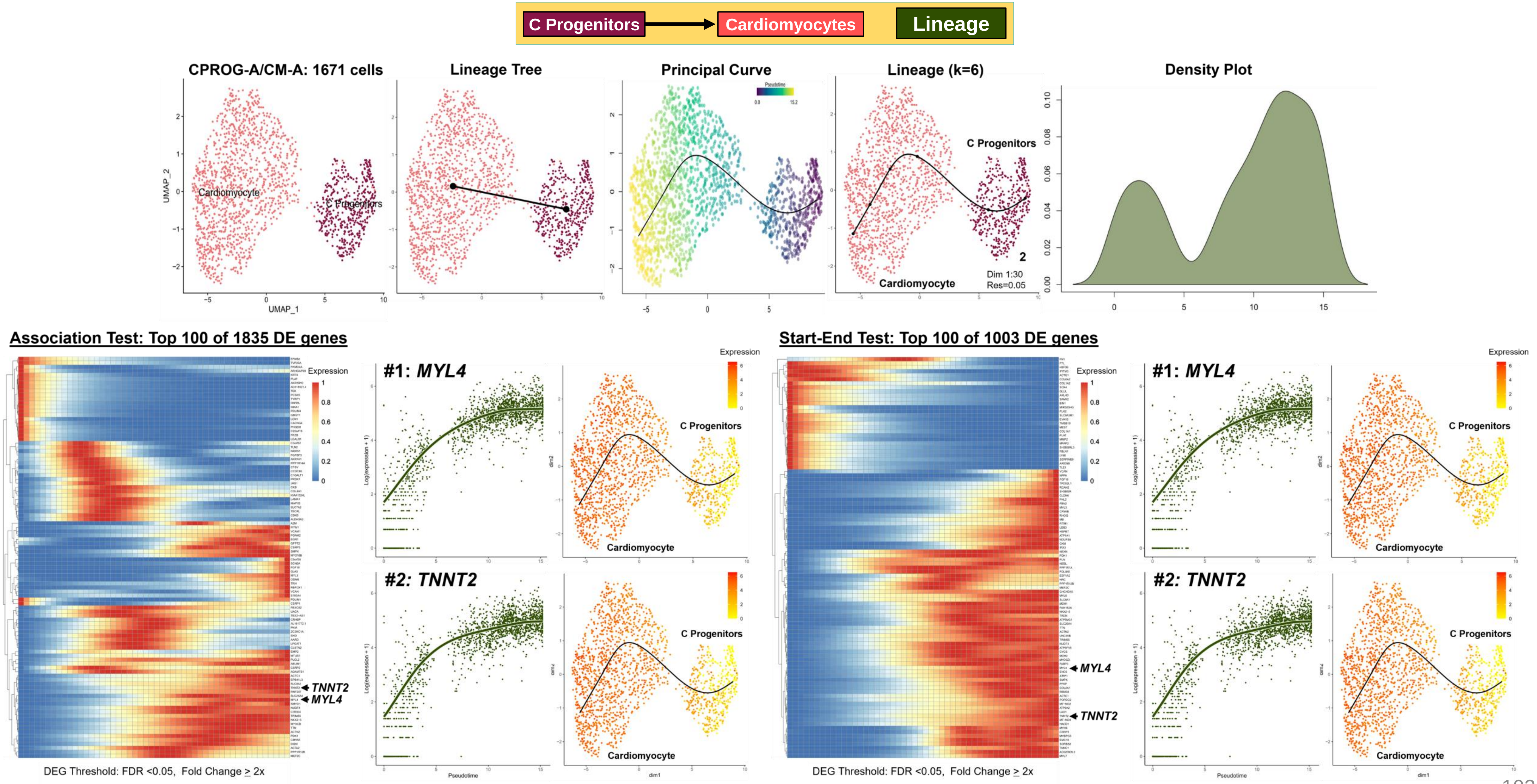

Fig. S11
Lineage DEG SS

Control	D09A	CPROG-A/CM-A	1,671 cells	Lineage DE Analyses & Enrichment

Over-Representation Analysis

C Progenitors → Cardiomyocytes | Lineage

Enrichment Plots: Top 5 Gene Sets

Enrichment Maps Top 25 Gene Sets

Enrichment Trees Top 25 Gene Sets

Top 100 of 1835 Association DEG

Enriched Gene Sets = 139

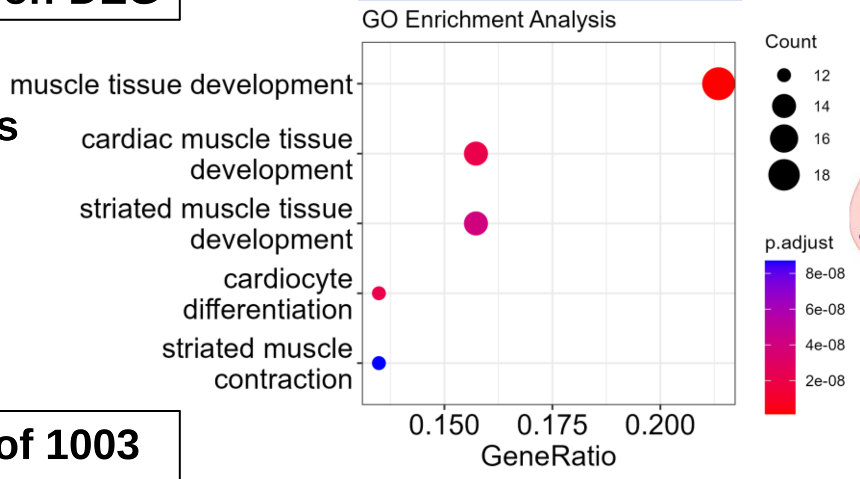

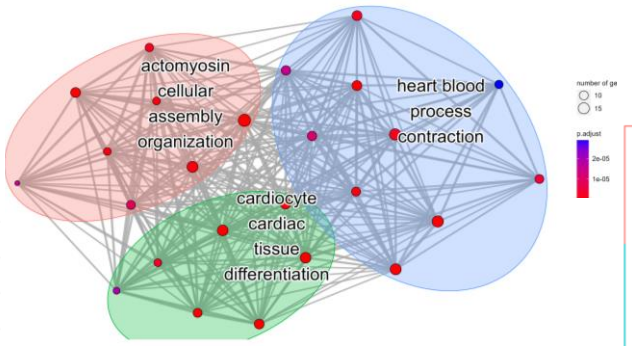

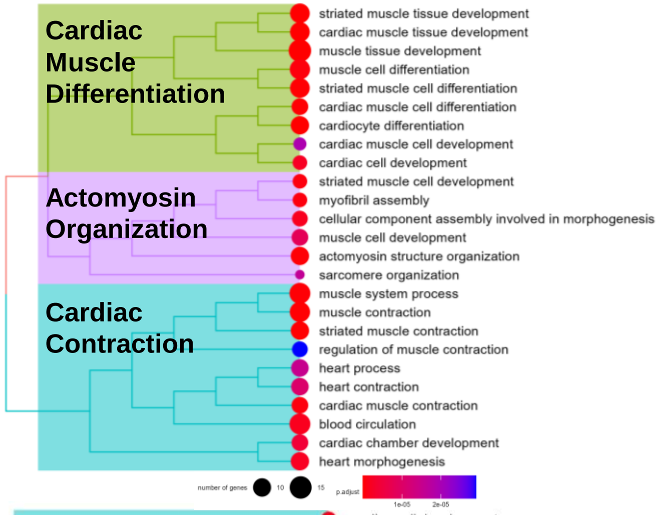

Top 100 of 1003 Start-End DEG

Enriched Gene Sets = 239

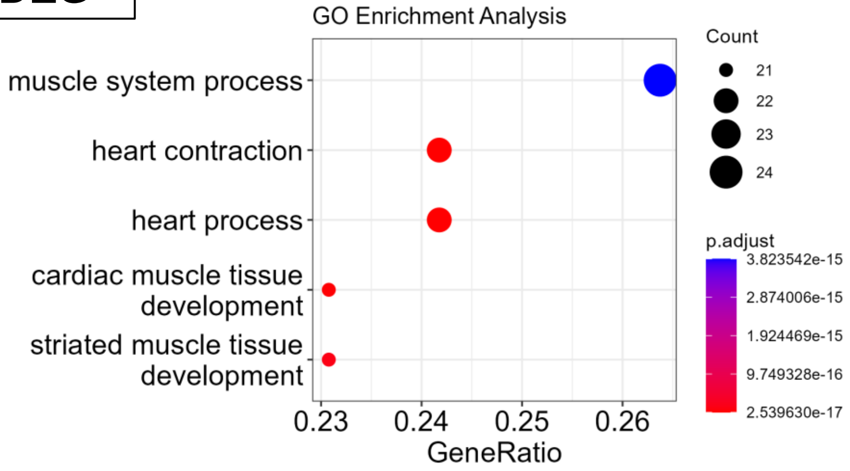

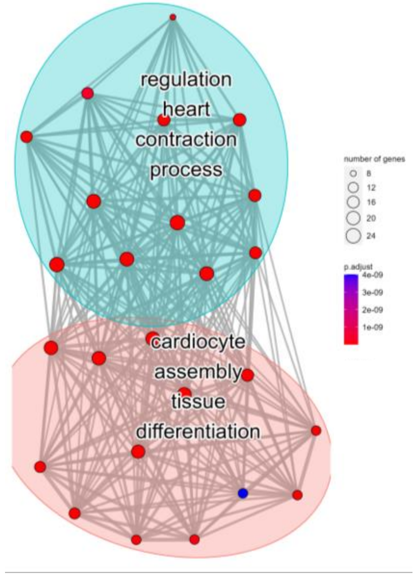

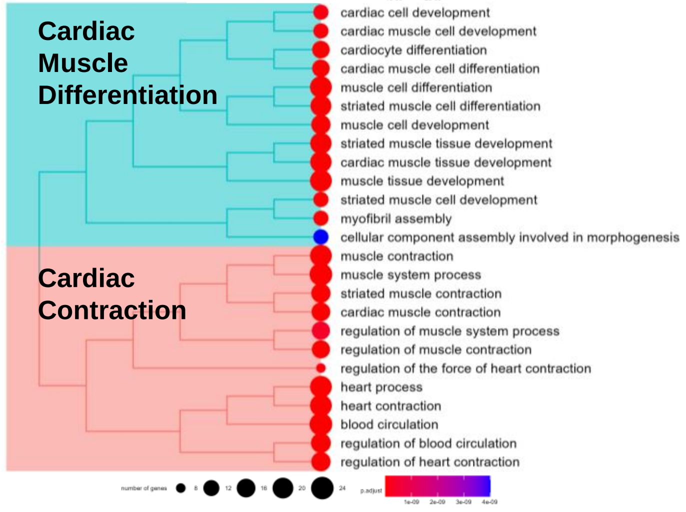

Fig. S11
Lineage DEG SS

Control	D09B	CPROG-A/CM-A	5,293 cells	Lineage DE Analyses & Enrichment

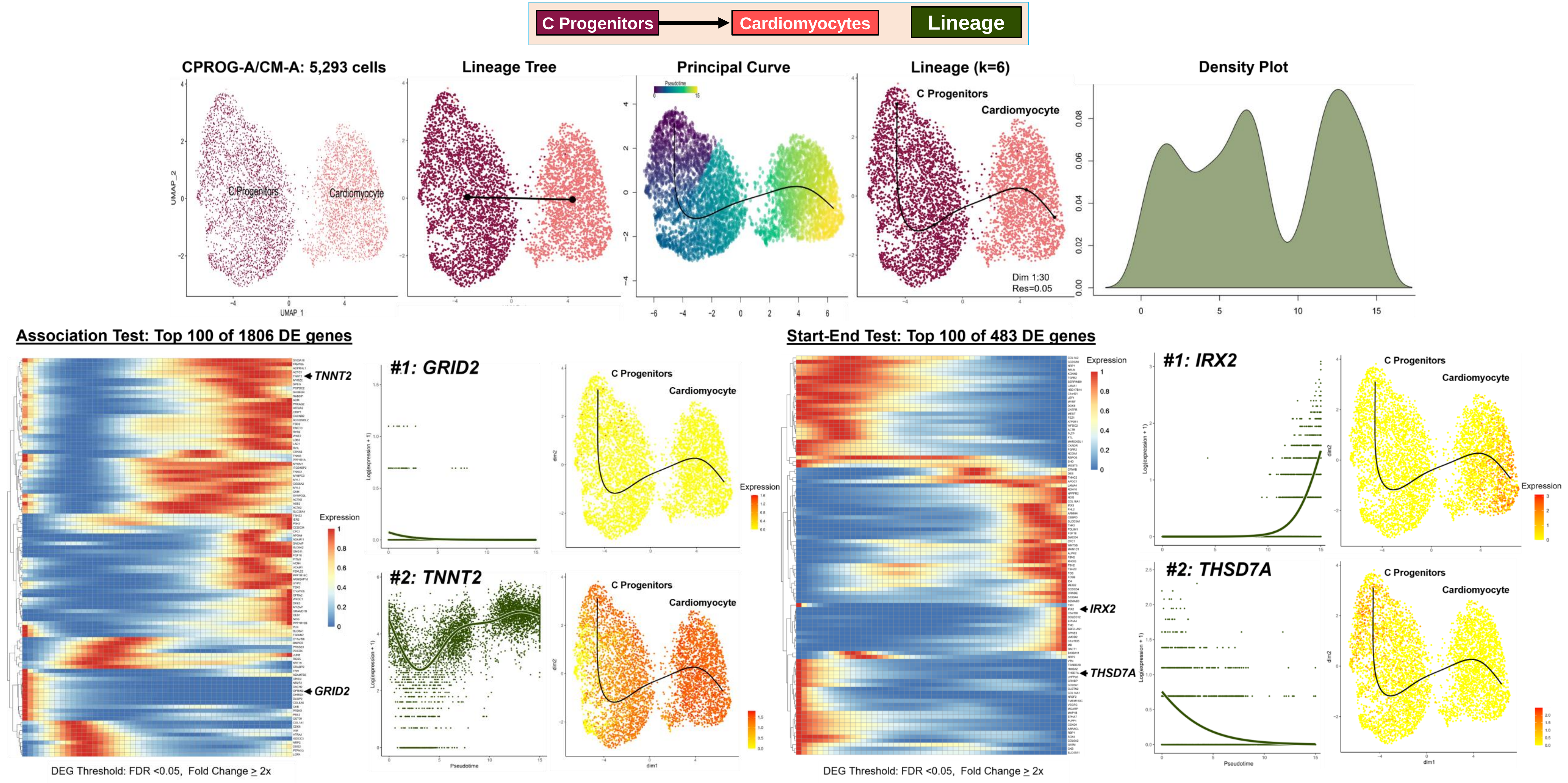

Fig. S11
Lineage DEG SS

Control	D09B	CPROG-A/CM-A	5,293 cells	Lineage DE Analyses & Enrichment

Over-Representation Analysis

C Progenitors → Cardiomyocytes Lineage

Enrichment Plots: Top 5 Gene Sets	Enrichment Maps Top 25 Gene Sets	Enrichment Trees Top 25 Gene Sets

Top 100 of 1806 Association DEG

Enriched Gene Sets = 153

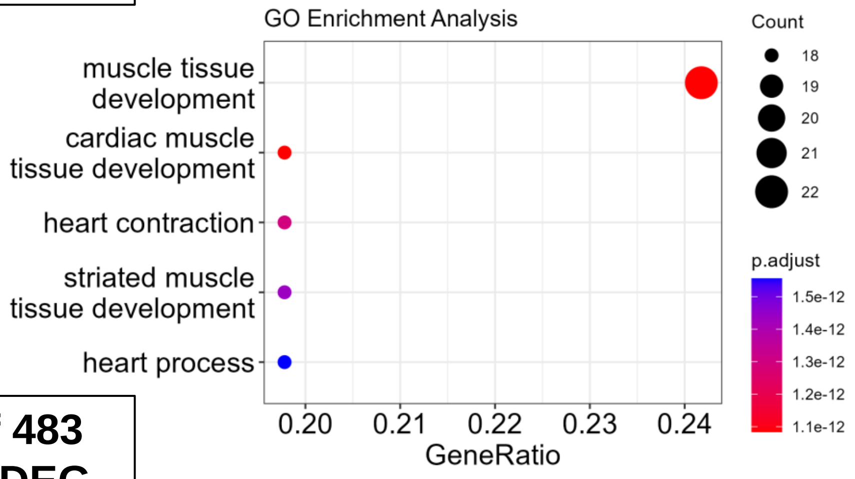

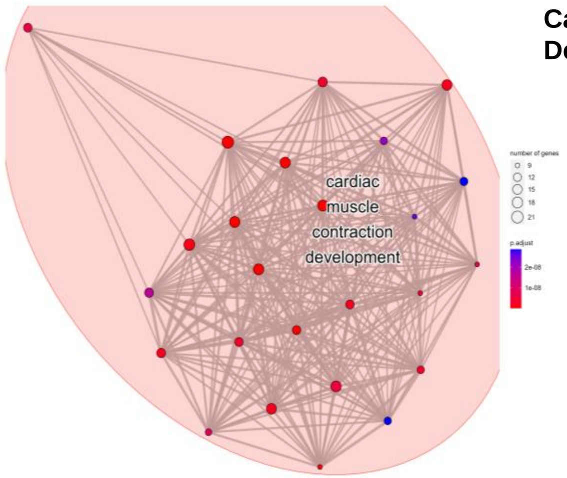

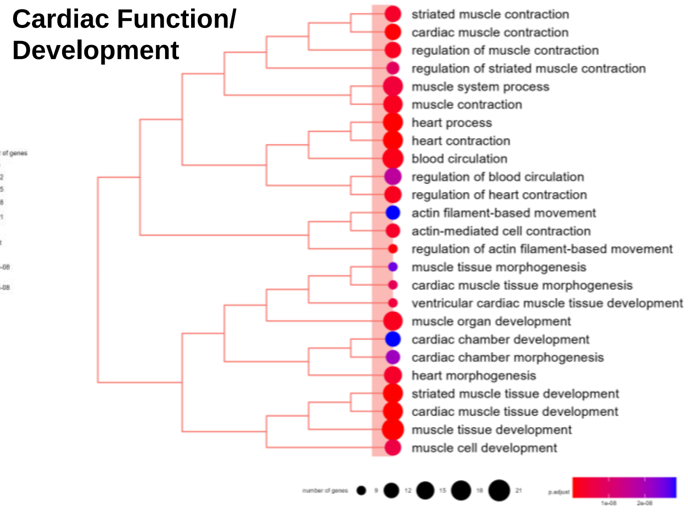

Top 100 of 483 Start-End DEG

Enriched Gene Sets = 305

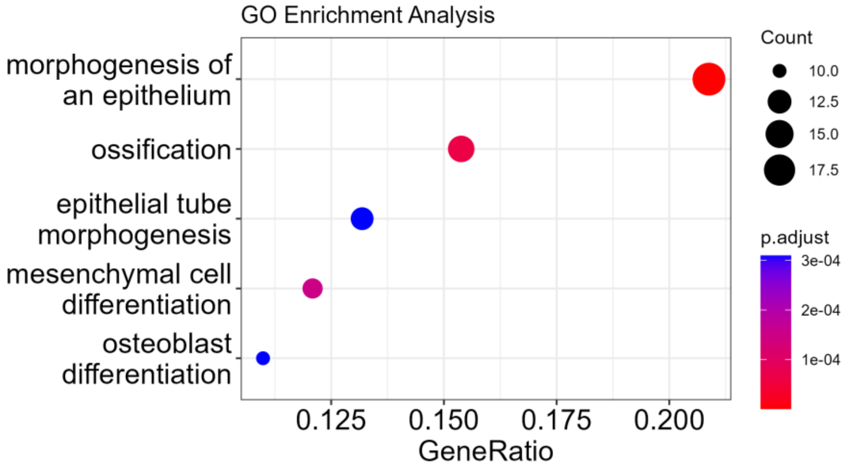

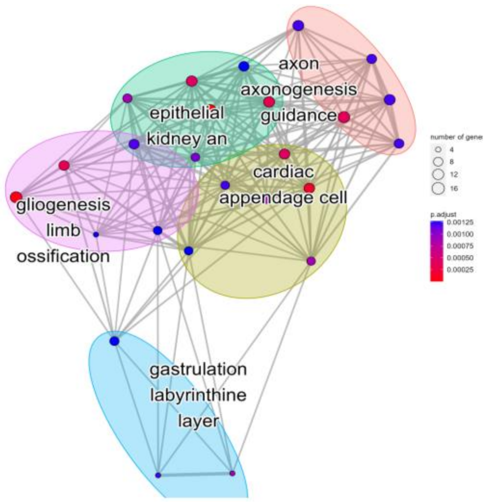

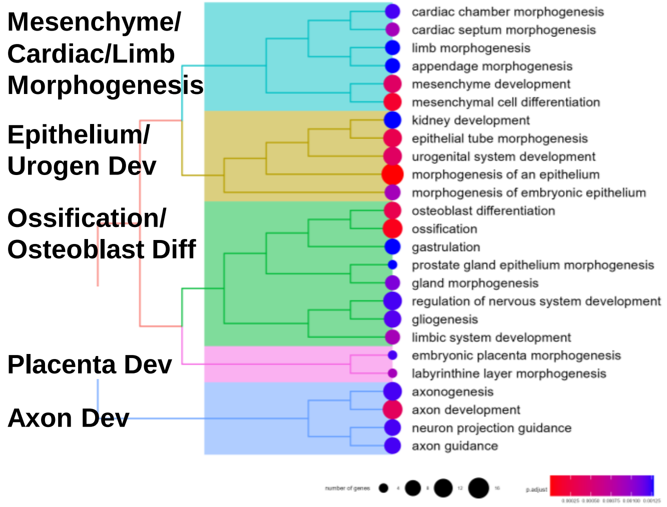

Fig. S11
Lineage DEG SS

Patient	D09B	CPROG-A/CM-A	4,091 cells	Lineage DE Analyses & Enrichment

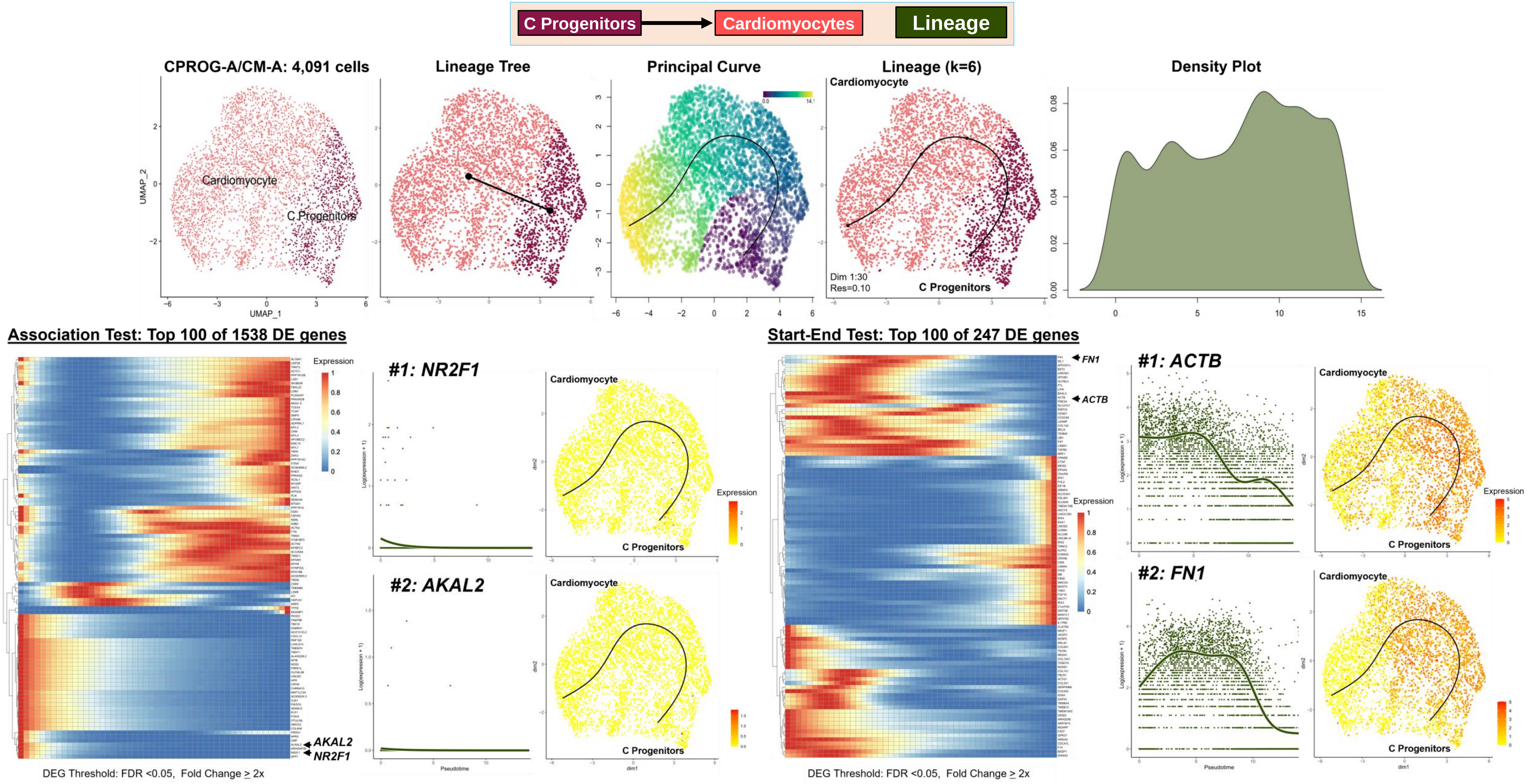

Fig. S11
Lineage DEG SS

Patient	D09B	CPROG-A/CM-A	4,091 cells	Lineage DE Analyses & Enrichment

Over-Representation Analysis

C Progenitors → Cardiomyocytes | Lineage

Enrichment Plots: Top 5 Gene Sets

Enrichment Maps Top 25 Gene Sets

Enrichment Trees Top 25 Gene Sets

Top 100 of 1538 Association DEG

Enriched Gene Sets = 117

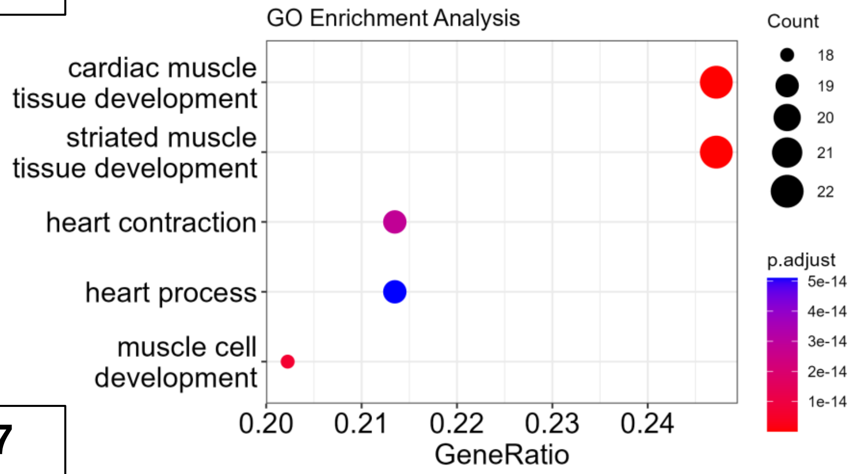

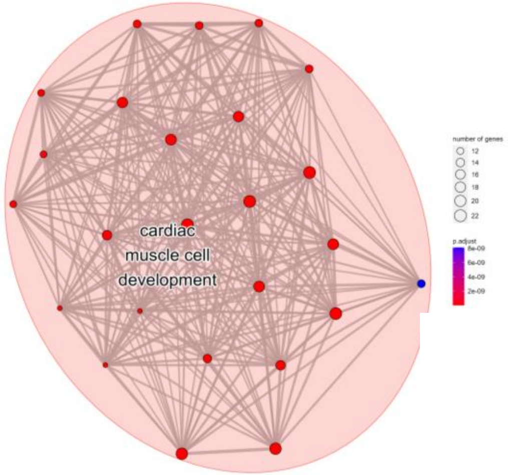

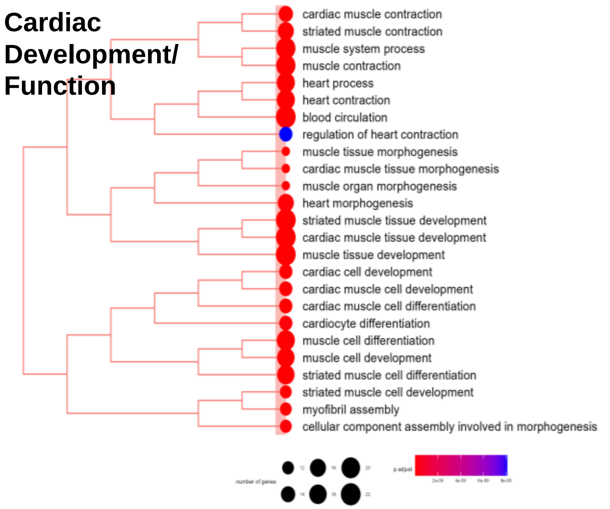

Top 100 of 247 Start-End DEG

Enriched Gene Sets = 195

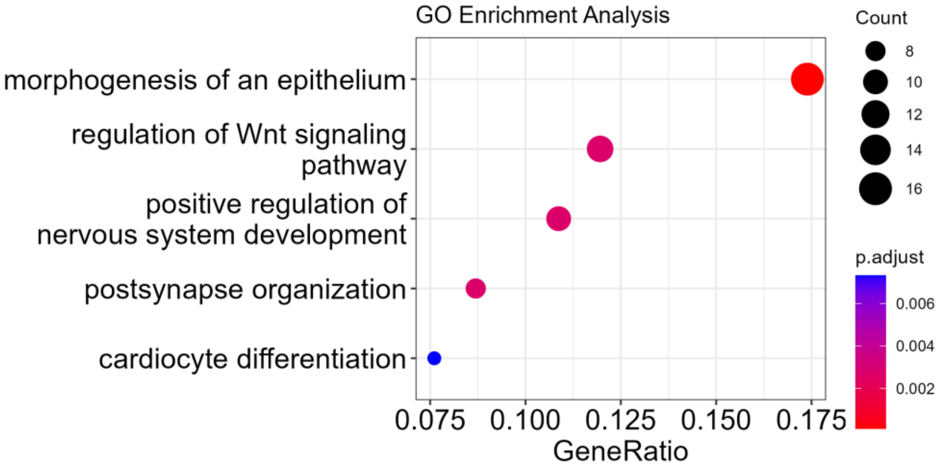

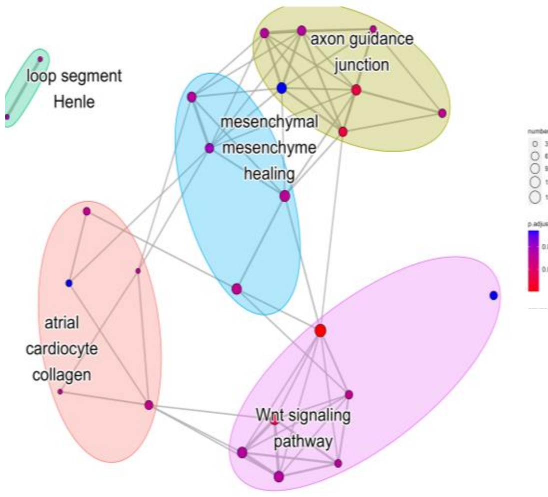

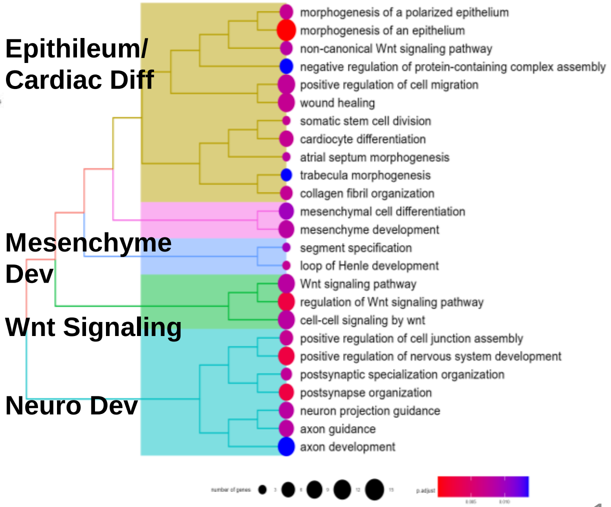

Fig. S12 Lineage DEG
INTEGR

Workflow Step-III: Paired Subset Data Results

Trajectory Analyses for Lineage-Specific Differential Expression and Enrichment

A. Summary: Lineage Differentially Expressed Genes (Lineage DEG)

Integrated Subset Data: 62,488 Total Cells

Dataset	Toplogy/ Lineage(s)	Condiments Tests (p=) Topology	Progress	Differ	DEG Type[a]	ORA[b] DEGs	GS	Top GO BP Enriched GS	GSEA[b] DEGs	GS	MSigDB Hallmark Enriched GS
DAY 0: Integrated BALANCED Subsets (Pluripotent-A, Pluripotent-B) 19,346 cells	Single Trajectory	Different (2.2 e-16)	Different (1.5 e-14)	N/A	AT	152	43	Metal Homeostasis	1130	0	None
					CT	97	4	Metal Homeostasis	827	0	None
					Top 100						
					Gr-A	74	22	Metal Homeostasis			
					Gr-B	23	2	Transcription			
Day 9B,16,19: Integrated BALANCED Subsets (C Prog, CM-A, EPDC) 43,142 cells	Bifurcating Trajectory	Different (6.9 e-4)	Different (4.0 e-12)	Different (6.8 e-4)	AT	2452	218	Mitosis/Cell Cycle	2880	6	E2F Targets
					CT	45	0	None	2290	0	G2M Checkpoint Spermatogenesis Mitotic Spindle DNA Repair MYC Targets V1
					L-1 CT	391	19		1404	0	None
					Top 100						
					Gr-A	10	3	Muscle Adaption Aerobic Respiration			
					Gr-B	10	0	None			
					Gr-C	59	6	Growth Factor Response Metal Homeostasis			
					Gr-D	21	61	BMP Signaling			
					L-2 CT	25	0		2191	0	None
					Top 25						
					Gr-A	19	2	Phagocytosis			
					Gr-B	3	10	Antigen Processing Dosage Compensation			
					Gr-C	3	12	Cyclin Kinase			

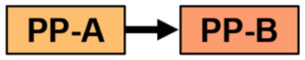

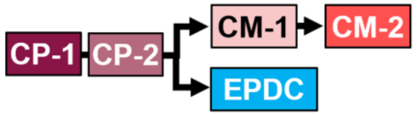

[a]Lineage DEG Type: Association Test (AT) and Condition Test (CT)
[b]Parameters: 1. ORA: DEG Threshold= FDR<0.05 & FC>2x, rank by Wald stat; min #DEGs= 3, adj p-value<0.05, q-value cutoff 0.20; 2. GSEA: DEG Threshold= FDR<0.05 & FC>2x, WaldStat>0.0; scoreType "pos", Adj p-value cutoff= 0.05, GS size 10-500
Abbreviations: PP, pluripotent; C Prog/CP, Cardiac Progenitors; CM, Cardiomyocyte; EPDC, Epicardium-derived cells; Progress, Progression; Differ, Differentiation; DEG, differentially expressed gene; ORA, Over-Representation Analysis; GO BP, Gene Ontology Biological Processes; GS, gene set; GSEA, Gene Set Enrichment Analysis; MSigDB, Molecular Signatures Database; Gr, Group; L, Lineage

Fig. S12 Lineage DEG
INTEGR

B. Summary: UMAP Plots and Cell Lineages (n=2: 62,488 Total Cells)

Control Subset Data ✚ **Patient Subset Data** → **Integrated Subset Data** → **Cell Lineages**

D00 PP-AB

19,346 cells

Integrated

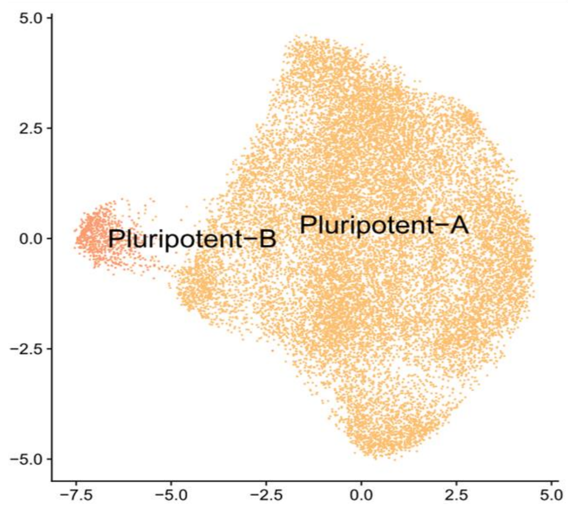

Lineages

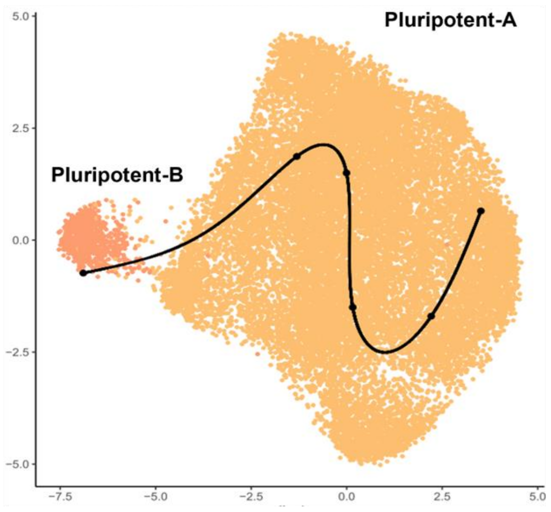

D09B-D16-D19 CPROG/CM-A/EPDC

43,142 cells

Integrated

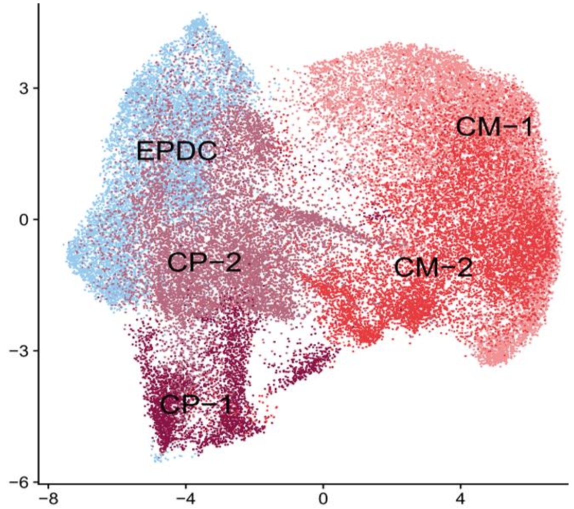

Lineages

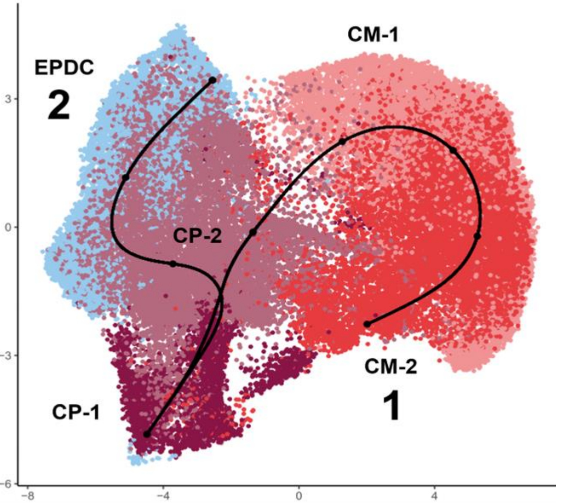

Fig. S12 Lineage DEG
INTEGR

C. Paired Subset Data Analyses: Pluripotent Cell Lineage (19,346 Total Cells)

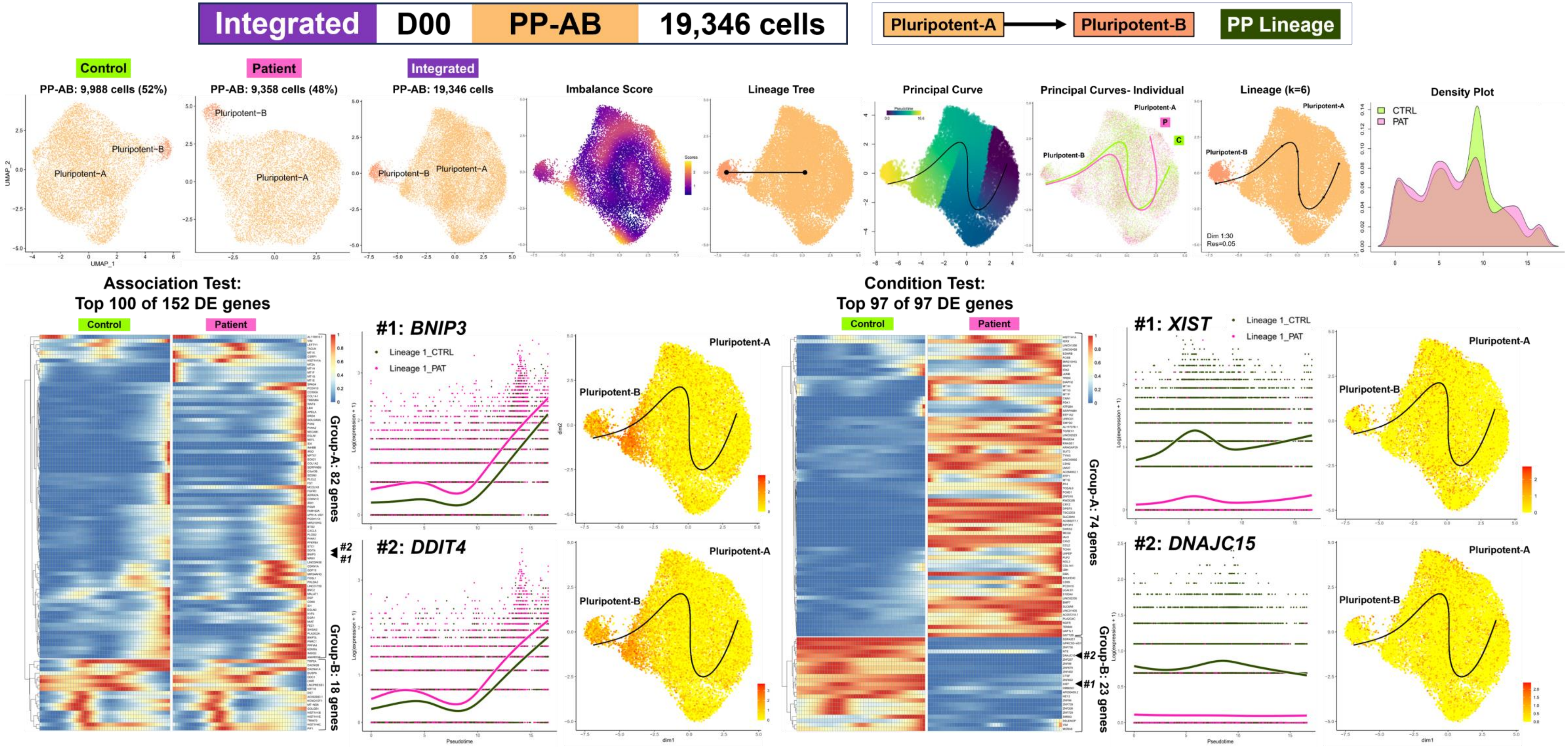

Fig. S12 Lineage DEG INTEGR

Integrated | D00 | PP-AB | 19,346 cells

Pluripotent-A → Pluripotent-B | PP Lineage

ASSOCIATION Test DEG Enrichment

Over-Representation Analysis

Top 100 of 152 Association DEG

Enrichment Plots: Top 5 Gene Sets

Enrichment Maps Top 25 Gene Sets

Enrichment Trees Top 25 Gene Sets

Group-A
82 genes
Enriched Gene Sets = 48

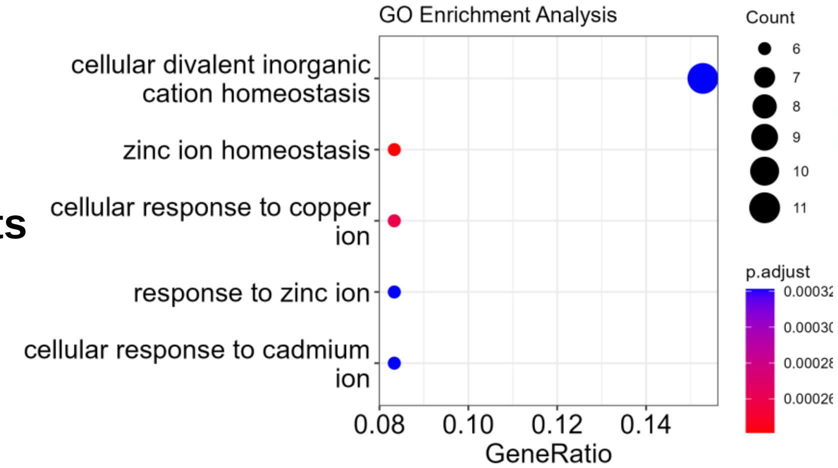

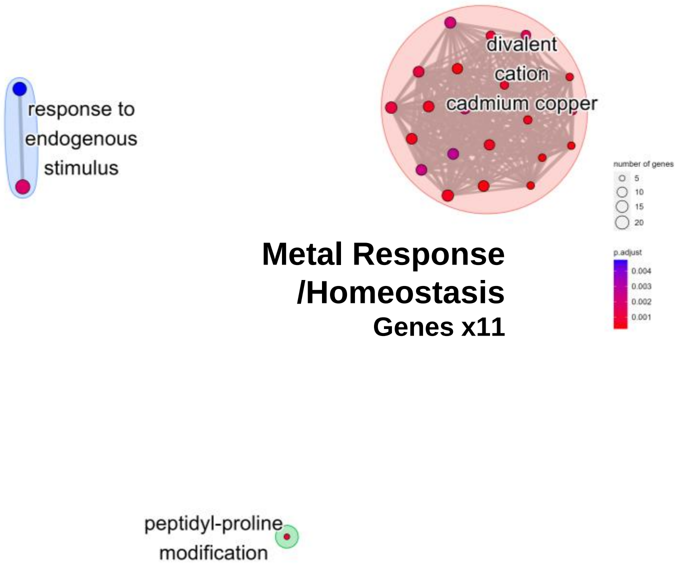

Metal Response /Homeostasis
Genes x11

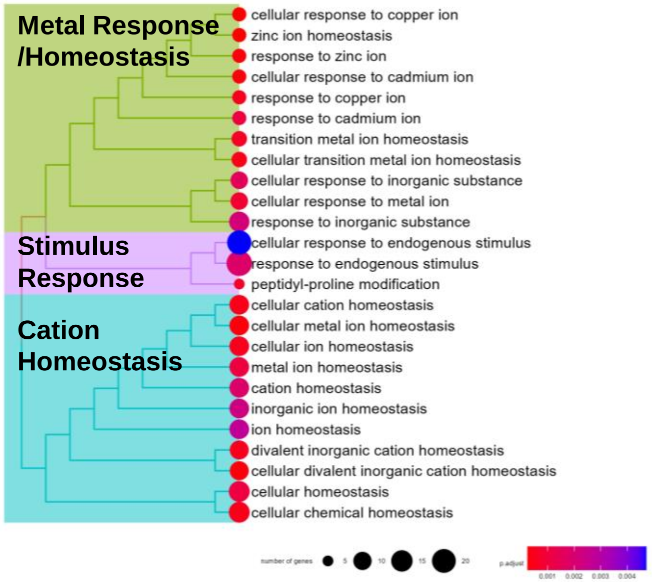

Group-B
18 genes
Enriched Gene Sets = 0

No Enriched GO BP Gene Sets | No Enriched GO BP Gene Sets | No Enriched GO BP Gene Sets

Gene Set Enrichment Analysis

Association Test: 1130 DEG Total

No Enriched MSigDB Hallmark Gene Sets

Fig. S12 Lineage DEG INTEGR

Integrated	D00	PP-AB	19,346 cells

Pluripotent-A → Pluripotent-B | PP Lineage

CONDITION Test DEG Enrichment

Over-Representation Analysis

Top 97 of 97 Condition DEG

Enrichment Plots: Top 5 Gene Sets

Enrichment Maps Top 25 Gene Sets

Enrichment Trees Top 25 Gene Sets

Group-A
74 genes
Enriched Gene Sets = 22

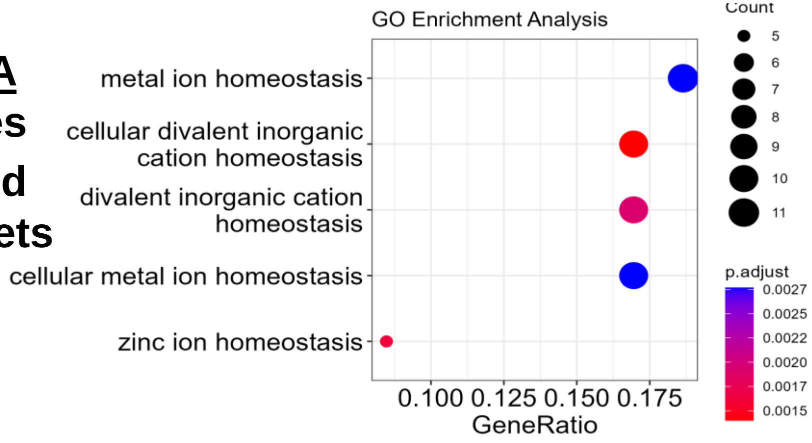

Metal/Cation Homeostasis
Genes x11

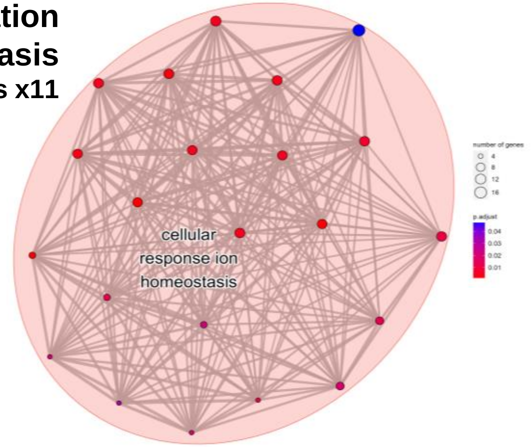

Metal/Cation Homeostasis

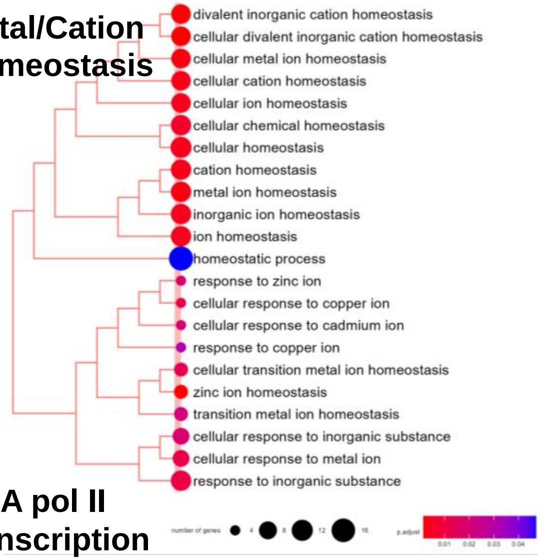

Group-B
23 genes
Enriched Gene Sets = 2

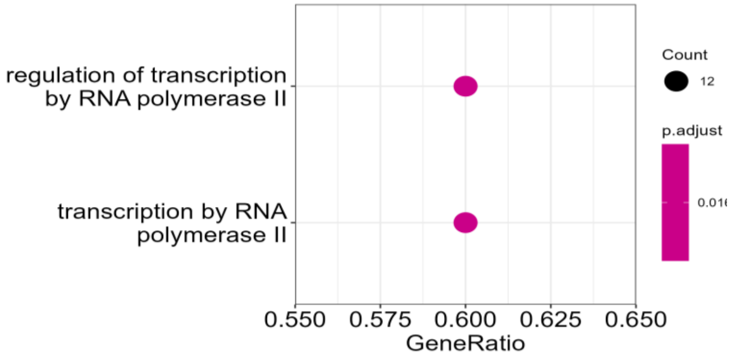

RNA pol II transcription
Genes x12

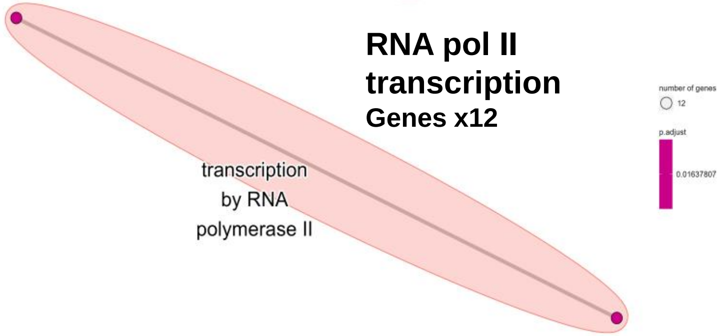

RNA pol II transcription

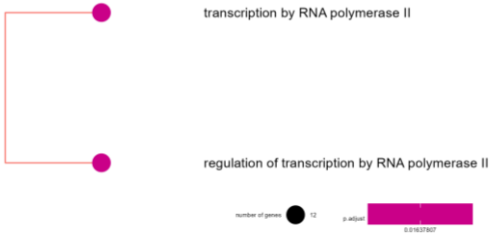

Gene Set Enrichment Analysis

Condition Test: 827 DEG Total

No Enriched MSigDB Hallmark Gene Sets

Fig. S12 Lineage DEG
INTEGR

D. Paired Subset Data Analyses: Cardiac Progenitor Lineages (43,142 Total Cells)

Integrated	D09B-D16-D19	CPROG/CM-A/EPDC	43,142 cells

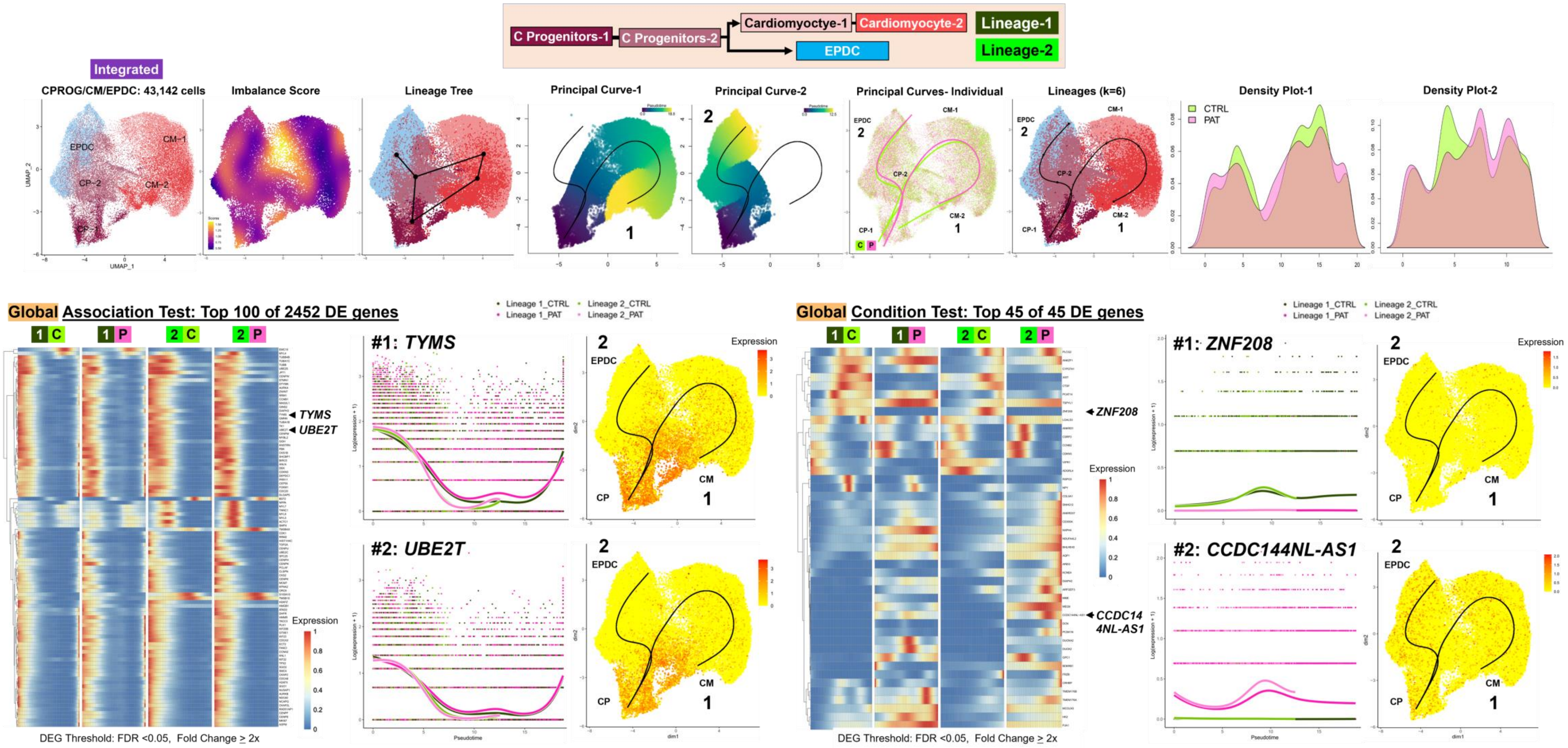

Fig. S12 Lineage DEG
INTEGR

Integrated | D09B-D16-D19 | CPROG/CM-A/EPDC | 43,142 cells

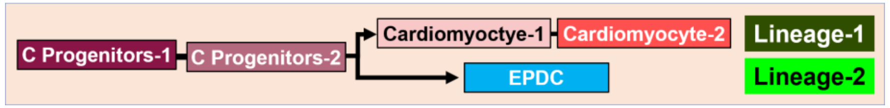

Over-Representation Analysis

Top 100 of 2452 Association DEG

Enrichment Plots: Top 5 Gene Sets

Enrichment Maps Top 25 Gene Sets

Enrichment Trees Top 25 Gene Sets

Enriched Gene Sets = 218

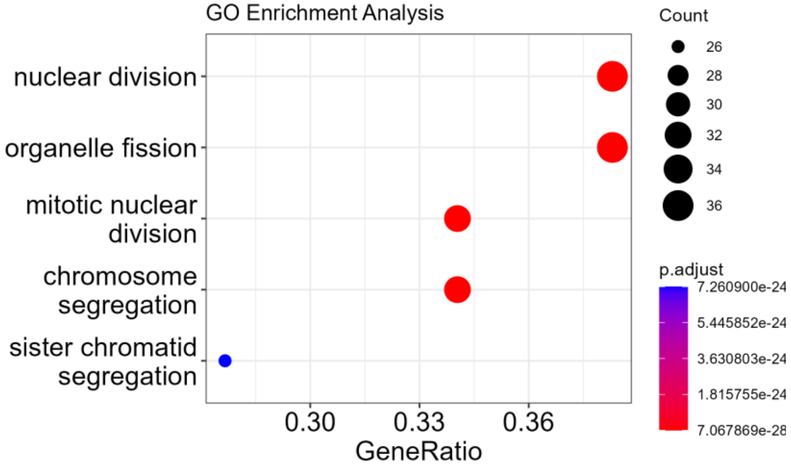

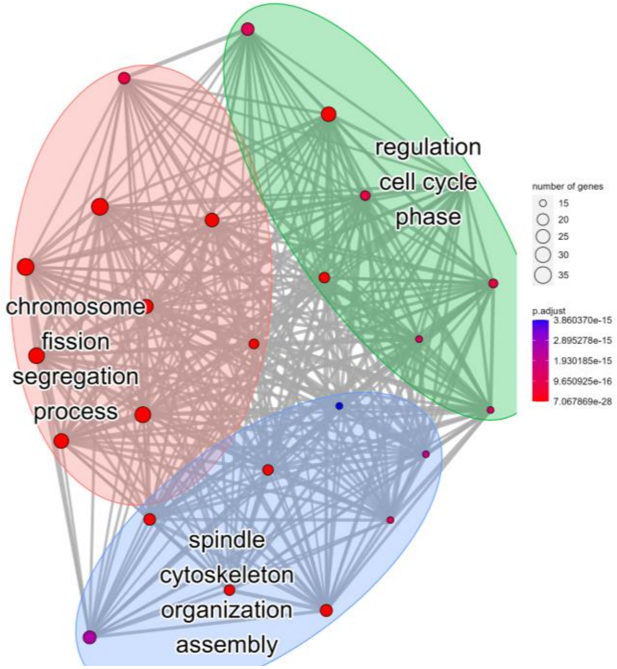

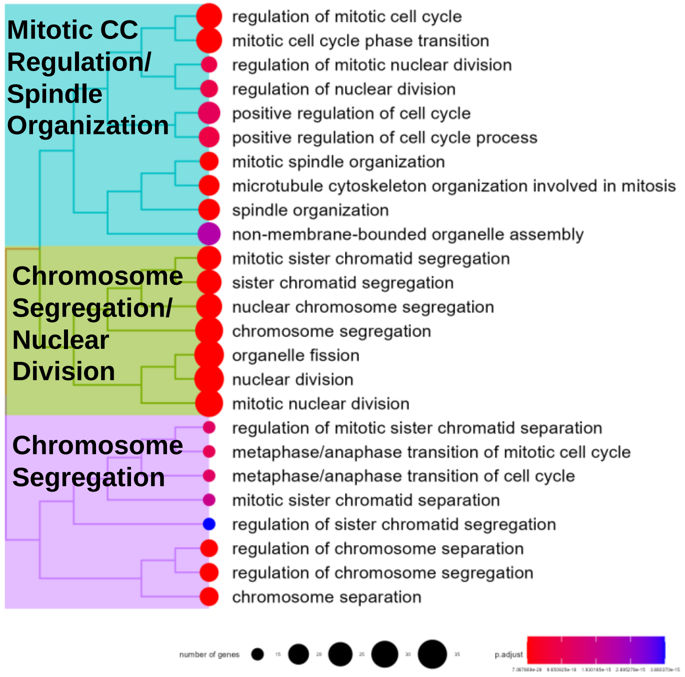

Top 45 of 45 Condition DEG

Enriched Gene Sets = 0

No Enriched GO BP Gene Sets

No Enriched GO BP Gene Sets

No Enriched GO BP Gene Sets

Fig. S12 Lineage DEG INTEGR

Integrated	D09B-D16-D19	CPROG/CM-A/EPDC	43,142 cells

Gene Set Enrichment Analysis

Association Test: 2880 DEG Total

Enriched Gene Sets = 6

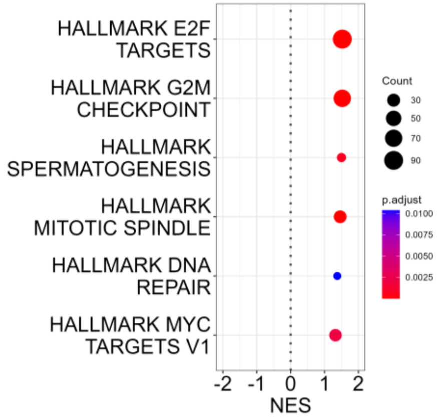

	NES	FDR
E2F TARGETS	1.52	2.2E-09
G2M CHECKPOINT	1.52	2.2E-09
SPERMATOGENESIS	1.50	0.00068
MITOTIC SPINDLE	1.46	2.7E-07
DNA REPAIR	1.38	0.0096
MYC TARGETS V1	1.32	0.0019

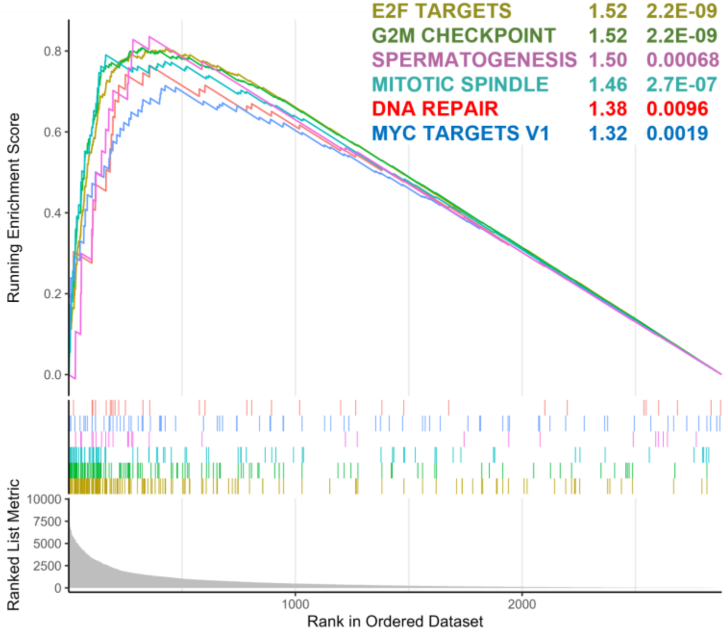

MODULE SCORING

E2F TARGETS

G2M CHECKPOINT

MITOTIC SPINDLE

MYC TARGETS V1

SPERMATO-GENESIS

DNA REPAIR

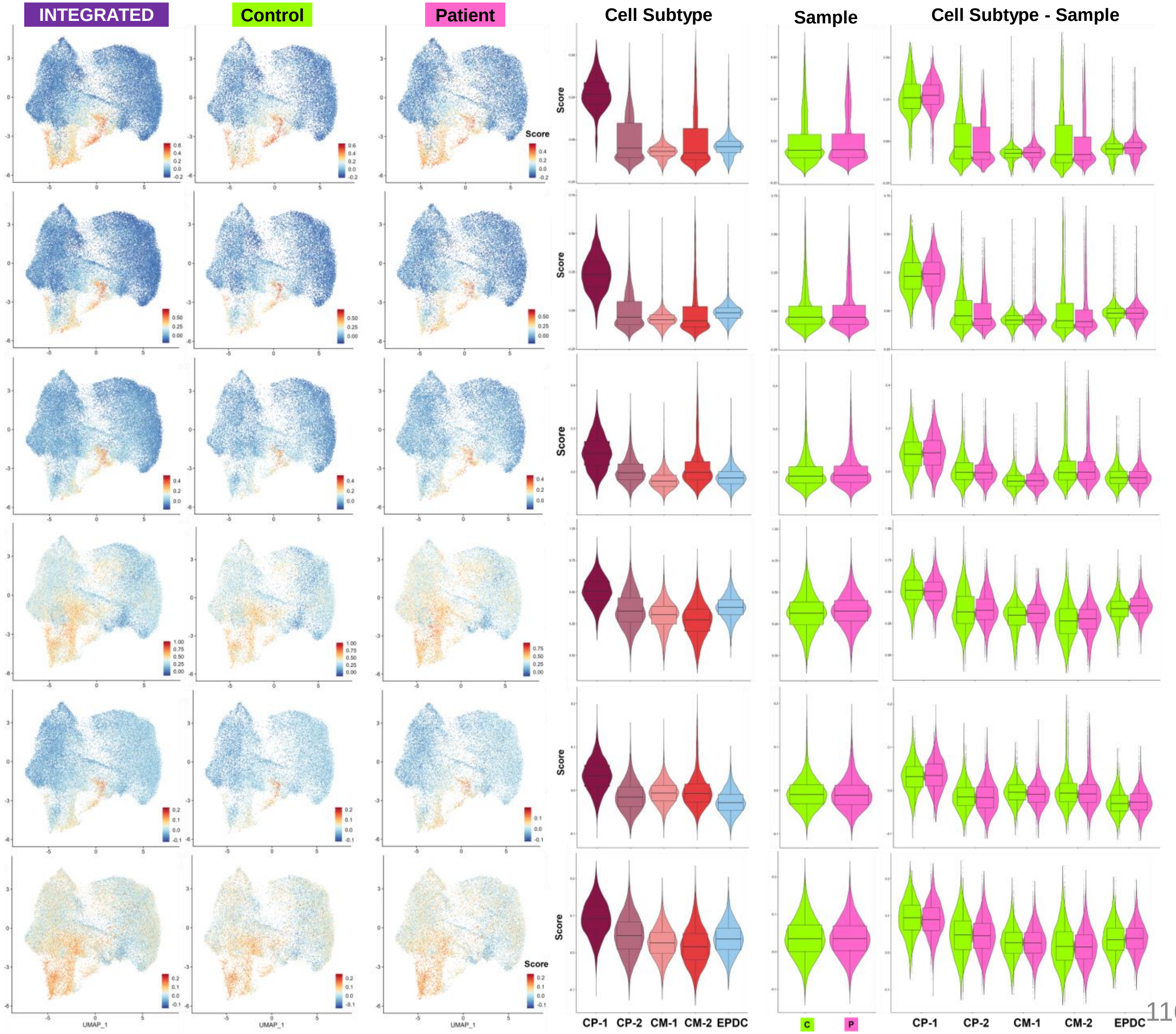

Fig. S12 Lineage DEG
INTEGR

Integrated	D09B-D16-D19	CPROG/CM-A/EPDC	43,142 cells

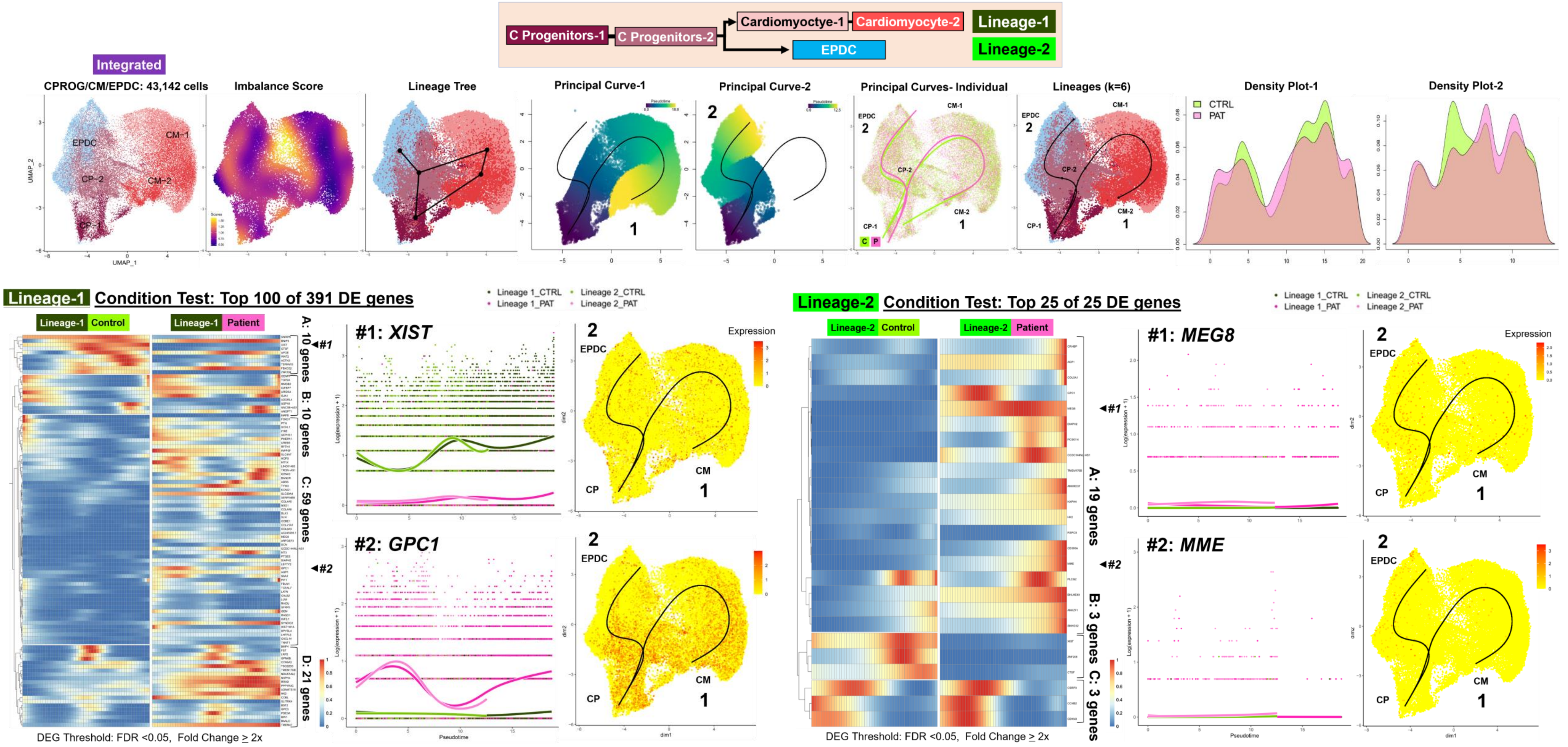

Fig. S12 Lineage DEG
INTEGR

Integrated | D09B-D16-D19 | CPROG/CM-A/EPDC | 43,142 cells

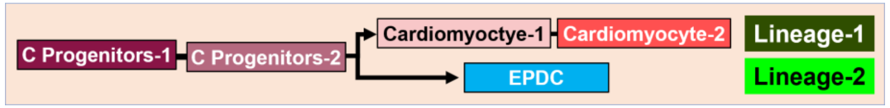

Over-Representation Analysis

Lineage-1

Top 100 of 391 Condition DEG

Enrichment Plots: Top 5 Gene Sets

Enrichment Maps Top 25 Gene Sets

Enrichment Trees Top 25 Gene Sets

Group A: 10 genes

Enriched Gene Sets = 3

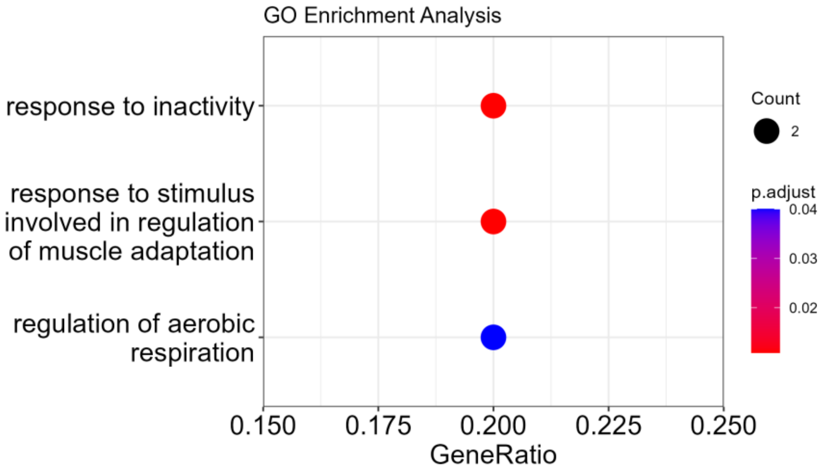

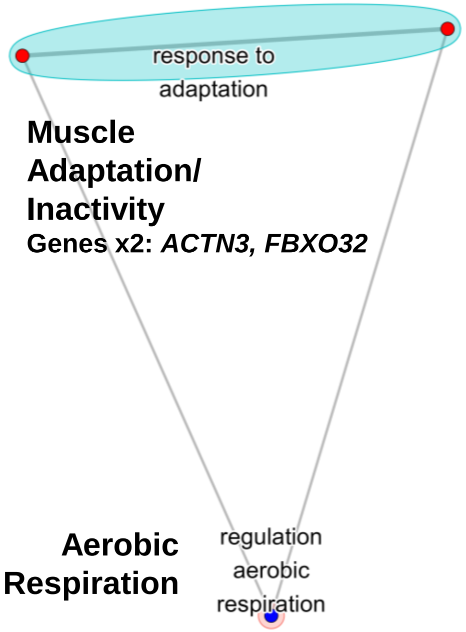

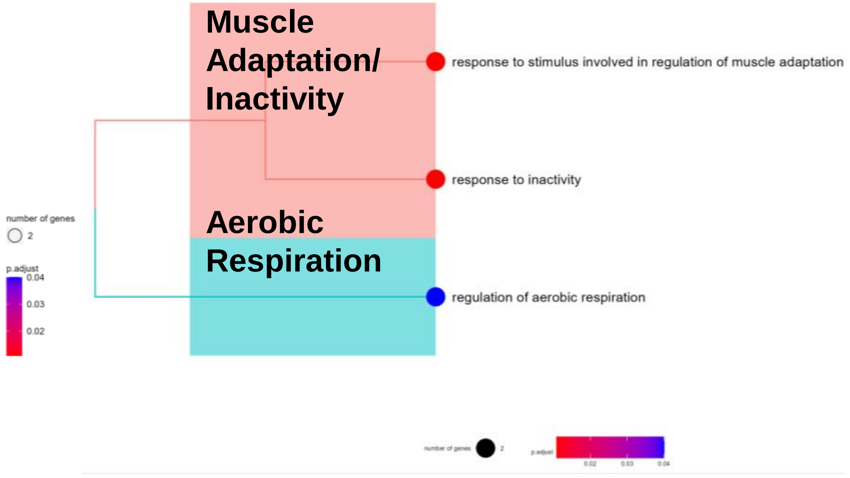

Group B: 10 genes

Enriched Gene Sets = 0

No Enriched GO BP Gene Sets

No Enriched GO BP Gene Sets

No Enriched GO BP Gene Sets

Fig. S12 Lineage DEG INTEGR

Integrated	D09B-D16-D19	CPROG/CM-A/EPDC	43,142 cells

Over-Representation Analysis

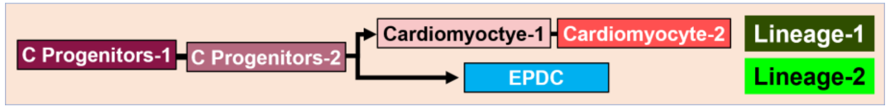

Lineage-1

Top 100 of 391 Condition DEG

Enrichment Plots: Top 5 Gene Sets

Enrichment Maps Top 25 Gene Sets

Enrichment Trees Top 25 Gene Sets

Group C: 59 genes

Enriched Gene Sets = 6

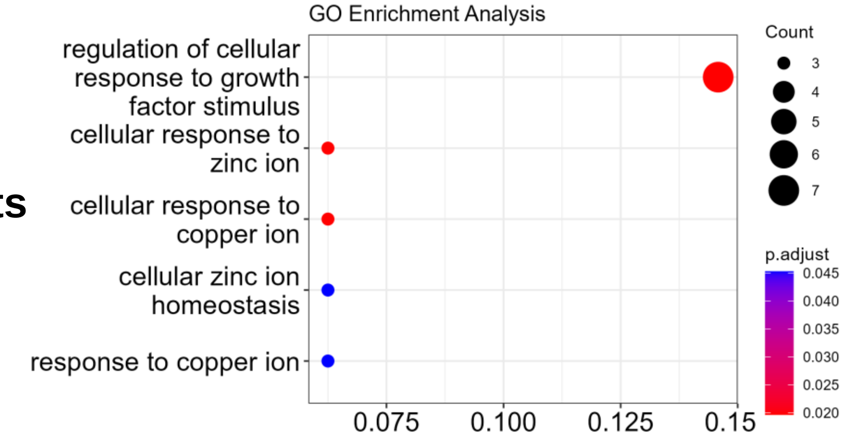

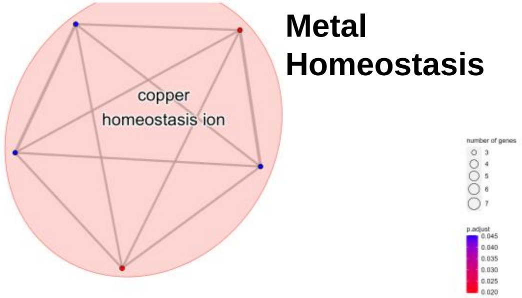

Metal Homeostasis

Growth Factor Response

Genes x7: *FOXD1, PMEPA1, CCBE1, DCNMT3, MT3, GPC1, SFRP5*

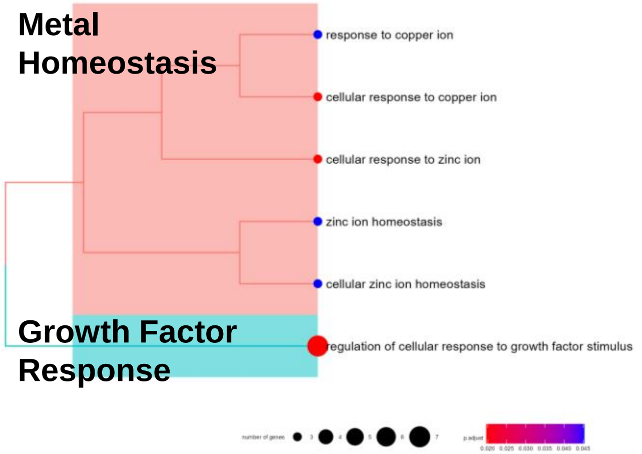

Metal Homeostasis

Growth Factor Response

Group D: 21 genes

Enriched Gene Sets = 61

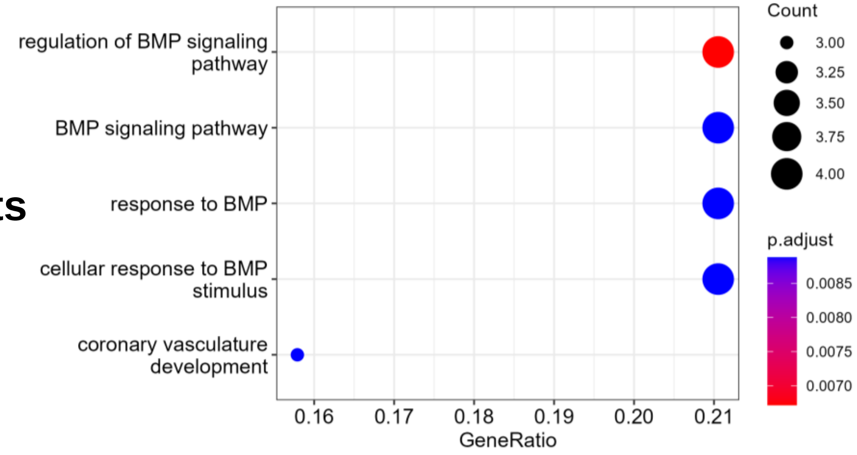

BMP Signaling

Genes x4: *BMP4, FST, LRP2,GPC3*

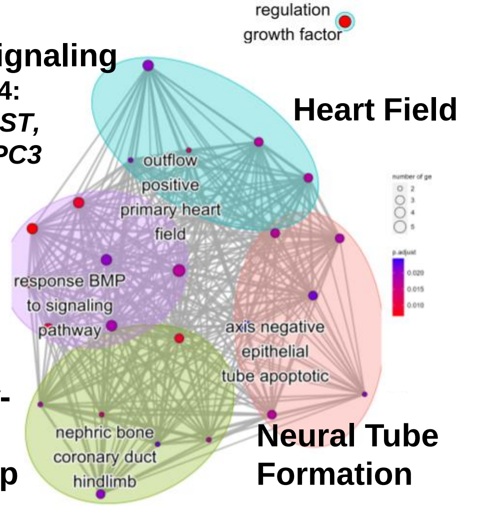

Heart Field

Kidney-Limb Develop

Neural Tube Formation

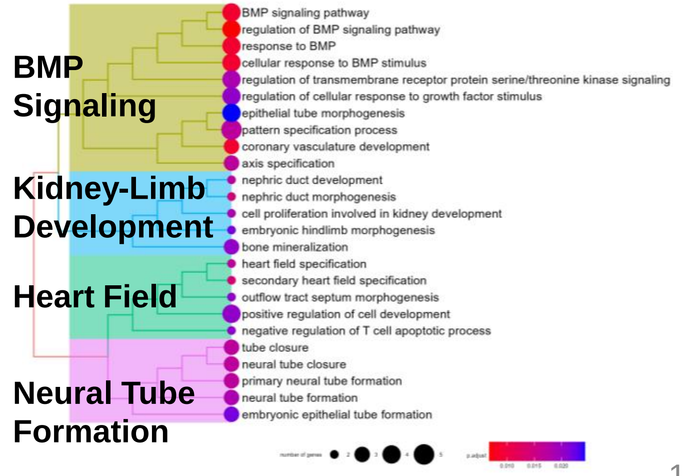

BMP Signaling

Kidney-Limb Development

Heart Field

Neural Tube Formation

Fig. S12 Lineage DEG
INTEGR

Integrated | D09B-D16-D19 | CPROG/CM-A/EPDC | 43,142 cells

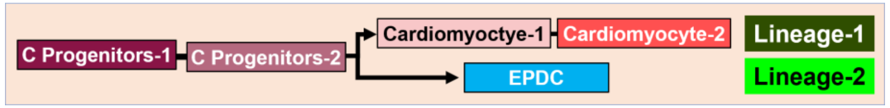

Over-Representation Analysis

Lineage-2

Top 25 of 25 Condition DEG

Enrichment Plots: Top 5 Gene Sets

Enrichment Maps Top 25 Gene Sets

Enrichment Trees Top 25 Gene Sets

Group A: 19 genes

Enriched Gene Sets = 2

Group B: 3 genes

Enriched Gene Sets = 10

Group C: 3 genes

Enriched Gene Sets = 12

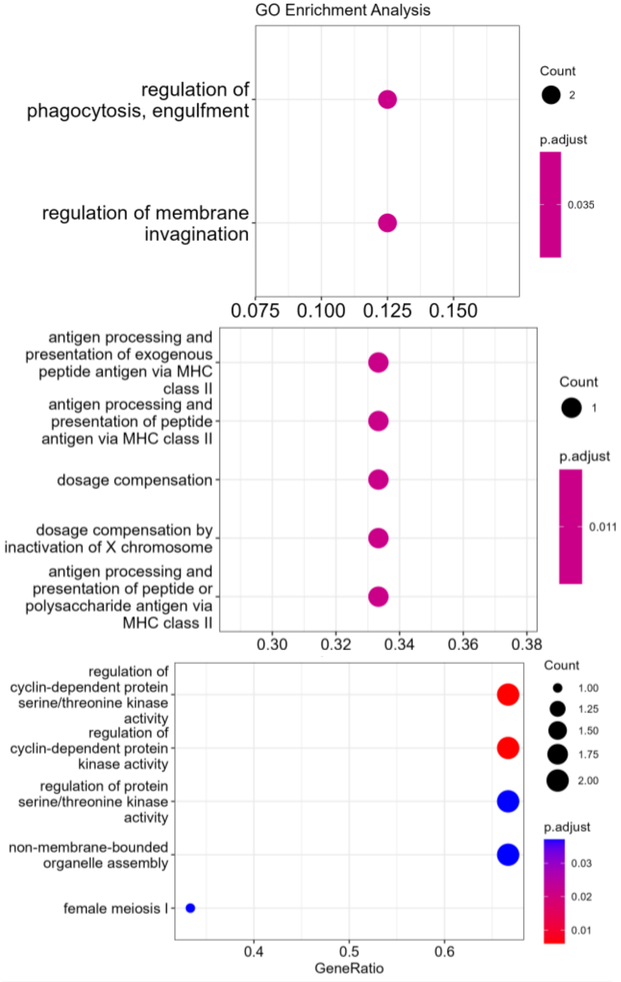

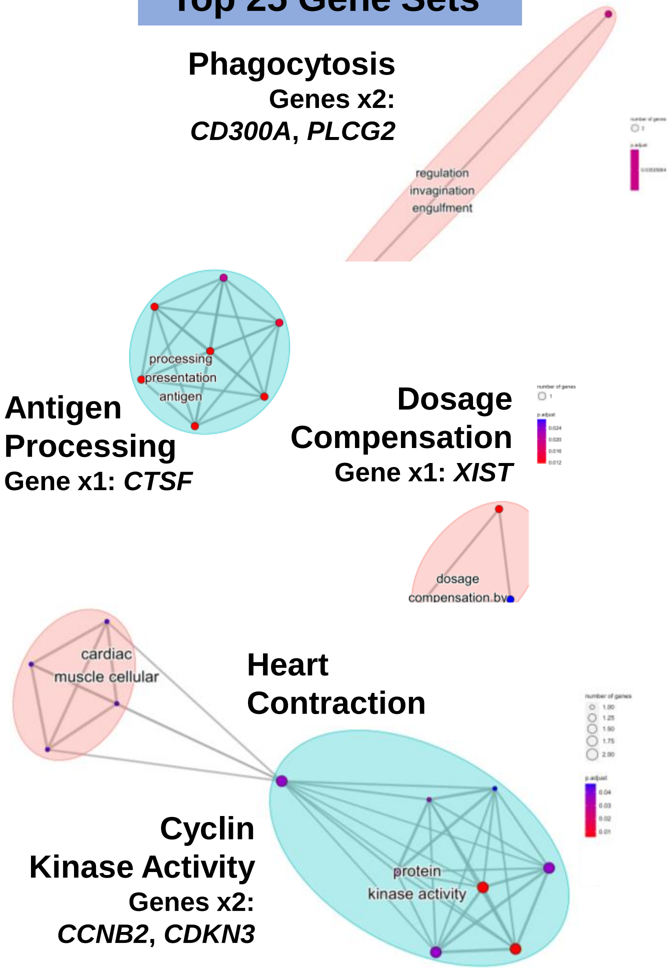

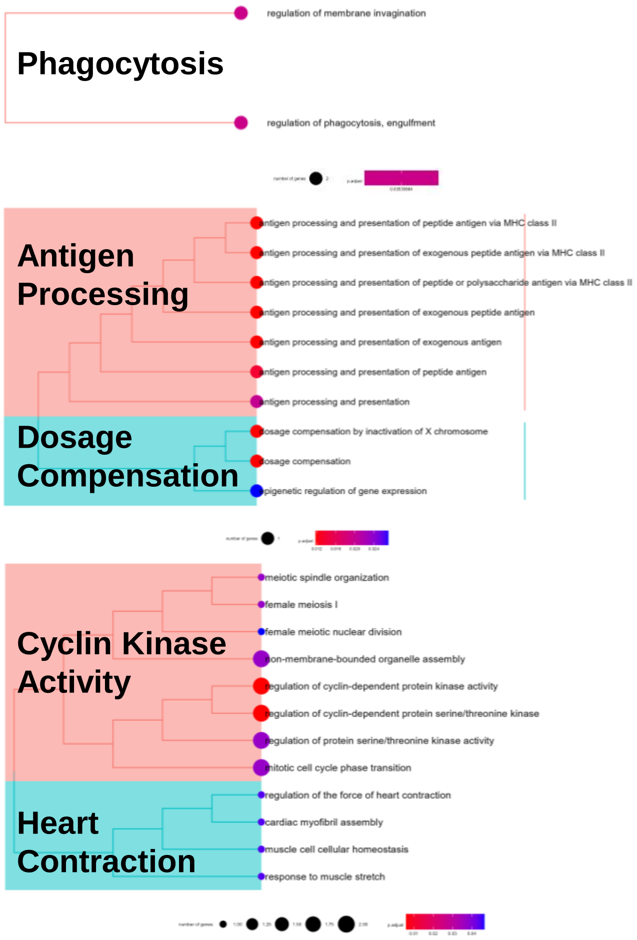

Fig. S13

Blot 1

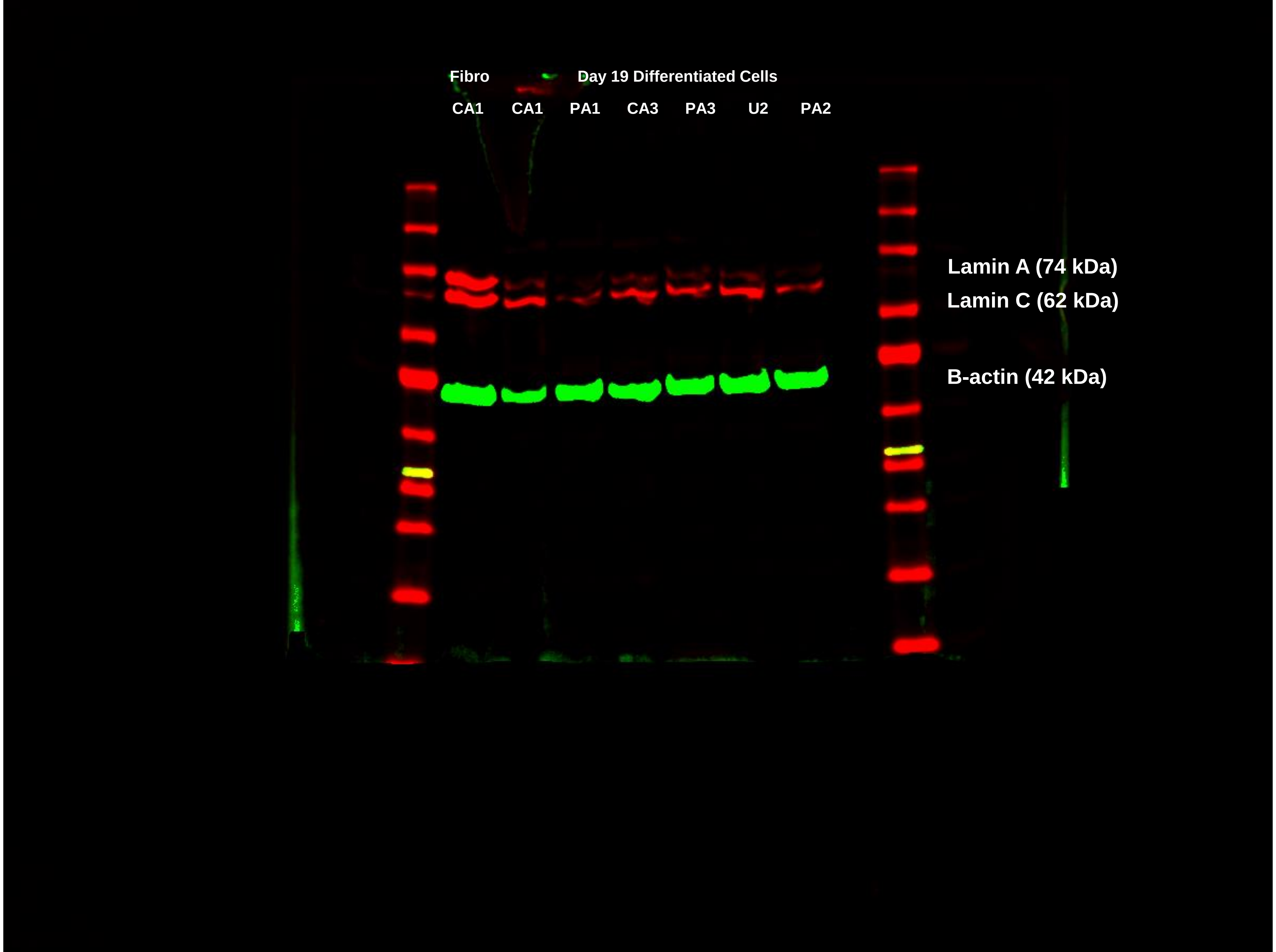

Fig. S13

Blot 2

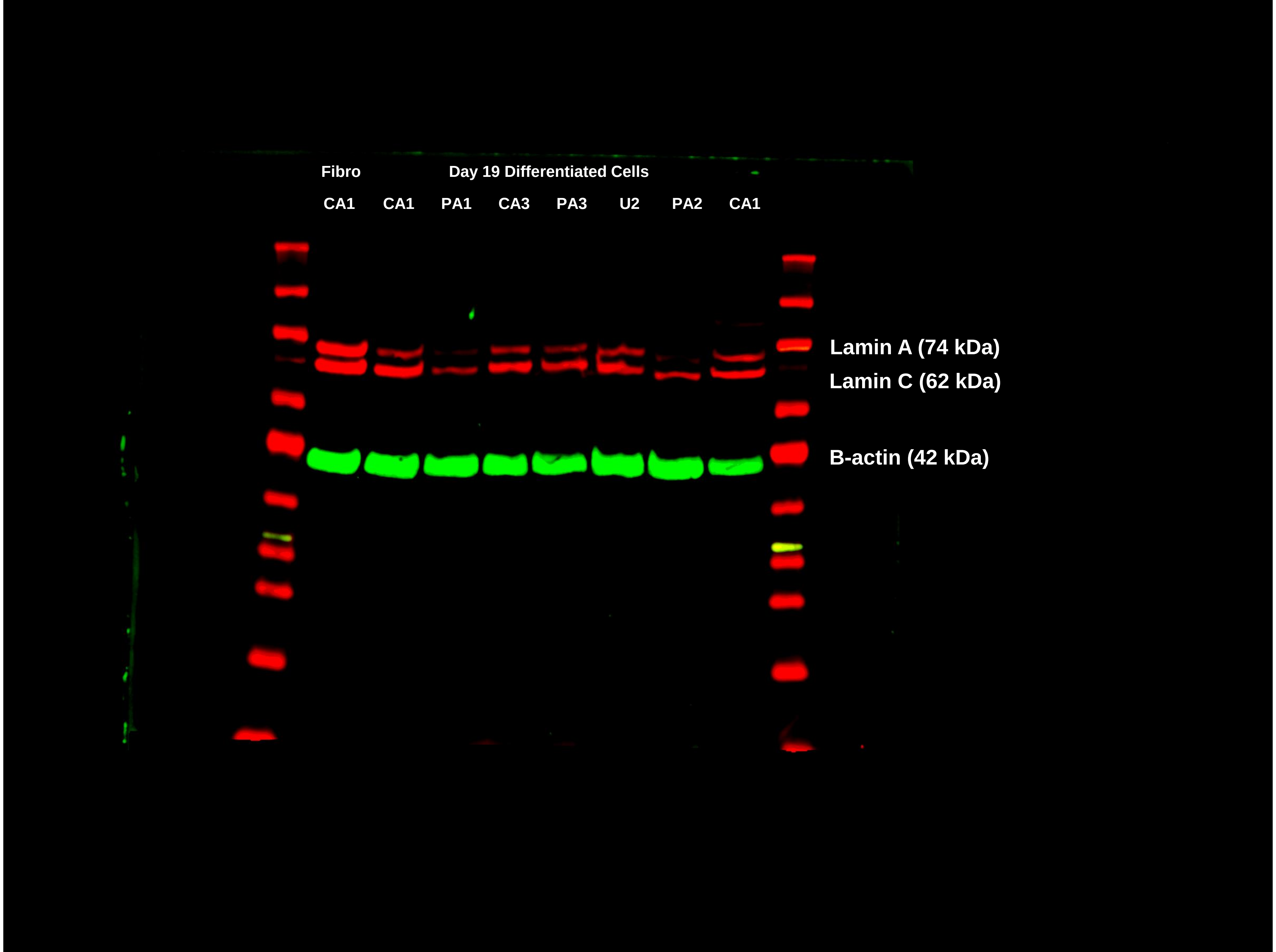

Fig. S13

Blot 3

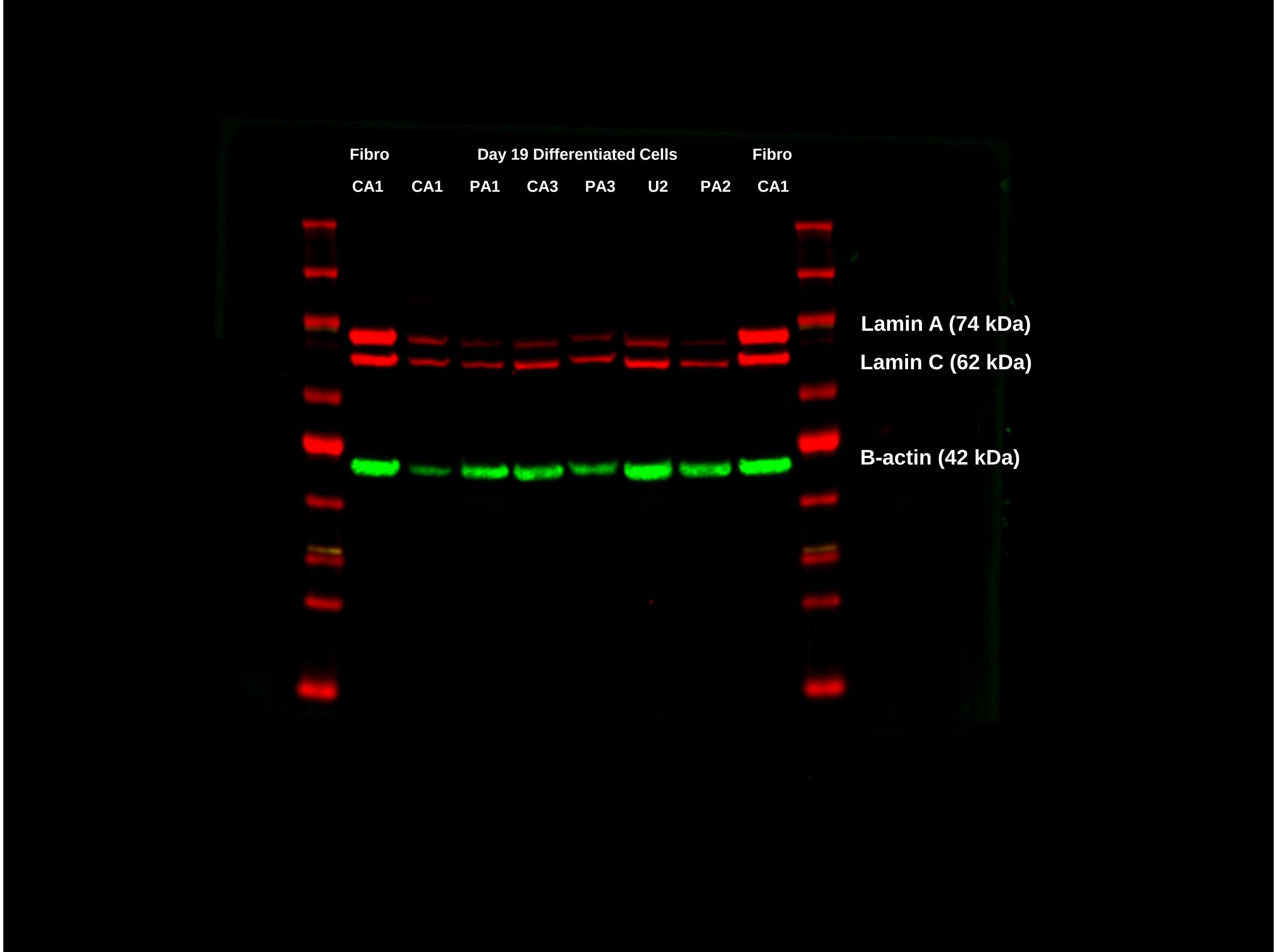

Supplement: Supplementary file 1 [file cells-13-01479-s001.zip › Zaragoza.Cells.REVISED.SUPPL.Figures_08.03.2024.pdf]
