# Supplementary material for: LMNA-Related Dilated Cardiomyopathy: Single-Cell Transcriptomics during Patient-Derived iPSC Differentiation Support Cell Type and Lineage-Specific Dysregulation of Gene Expression and Development for Cardiomyocytes and Epicardium-Derived Cells with Lamin A/C Haploinsufficiency
Source: Cells. 2024 Sep 3;13(17):1479. doi: 10.3390/cells13171479 (PMC11394257; doi:10.3390/cells13171479)
Supplement: Supplementary file 1 [file cells-13-01479-s001.zip › Zaragoza.Cells.REVISED.SUPPL.Text_08.14.2024.pdf]

LMNA-Related Dilated Cardiomyopathy: Single-Cell Transcriptomics during Patient-derived iPSC Differentiation Support Cell type and Lineage-specific Dysregulation of Gene Expression and Development for Cardiomyocytes and Epicardium-Derived Cells with Lamin A/C Haploinsufficiency.

M. Zaragoza, T-A. Bui, H. Widyastuti, M. Mehrabi, Z. Cang, Y. Sha, A. Grosberg, Q. Nie; Univ. of California, Irvine, Irvine, CA

Supplementary Text

2. Materials and Methods

2.1. Generation and Validation of Control and Patient iPSC Lines

To validate each iPSC line, we evaluated independent clones for absence of chromosome abnormalities by karyotype analysis and for normal pluripotency by immunocytochemistry (ICC) staining as reported (**Table S1**) [1]. Validation also included differentiation capability by ICC staining for three germ layer markers in embryoid bodies (EB). For each clone, we harvested iPSC at 70-80% confluency using 0.5 mM EDTA (Thermo Fisher Scientific (TFS), Waltham, MA) and transferred iPSC clumps to non-treated 35-mm culture dishes with Essential 6 Medium (TFS) and 5 µM ROCK inhibitor Y-27632 (Cellagen Technology, San Diego, CA). After four to seven days, we transferred unattached EB to Matrigel-coated chamber slides for spontaneous differentiation. After 14 to 28 days of EB differentiation, we stained cells for markers of endoderm (alpha-fetoprotein or Forkhead Box A2), mesoderm (smooth muscle actin), and ectoderm (beta-III tubulin) using the 3-Germ Layer Immunocytochemistry Kit (A25538, TFS) (**Table S2**).

2.2. In Vitro iPSC-CM Differentiation and Cell Collection

Although we used standard protocols to generated iPSC-CM (**Fig. 1A**), differences between Protocol-A and Protocol-B [2] include iPSC growth substrates and media. Protocol-B [2] involved seeding iPSC on Geltrex (TFS) coated 12-well plates in Essential 8 media (TFS). In contrast, for Protocol-A, we seeded thawed iPSC at passage 12 (P12) or above in Matrigel-coated 6-well plates in mTESR1 media (STEMCELL Technologies (SCT), Vancouver, Canada) and 10 µM ROCK inhibitor. After 24 hours, we replaced media with mTESR1 media and no ROCK inhibitor with daily changes. At 85-90% confluency, we passaged iPSC using ReLeSR (SCT). After at least two passages, we singularized iPSC with TrypLE Select Enzyme (TFS) and seeded cells to Matrigel-coated 12-well or 24-well plates in mTESR1 media and 10 µM ROCK inhibitor.

For CM differentiation, Protocol-A used the GiWi protocol [3, 4]. To determine optimal cell density, we seeded each iPSC clone at different concentrations three to four days prior to differentiation (D-3 or D-4) for growth to approximately 90-95% confluency. On Day 0 of differentiation (D00), we changed mTESR1 media to CM differentiation medium consisting of RPMI 1640 media (TFS) with B27

supplement without insulin (RPMI/B27-) (TFS) and with 7.5 µM CHIR99201 (Cayman Chemical Company, Ann Arbor, MI). After 48 hours (D02), we replaced media with RPMI/B27- with 7.5 µM IWP2 (R&D Systems, Minneapolis, MN). After 48 hours (D04), we replaced media with RPMI/B27-. On Day 6 of differentiation (D06), we replaced media with RPMI 1640 media supplemented with B27 supplement with insulin (RPMI/B27+) (TFS). The protocol produced beating clusters on D08 to D10.

To collect iPSC-CM at selected time points, we continued culturing in RPMI/B27+ media with changes every other day and used the STEMdiff CM Dissociation Kit (SCT) for harvesting. After washing wells twice with DPBS, we harvested cells by incubation in pre-warmed Dissociation Medium for 10 to 12 minutes at 37C, resuspended dissociated cells in Support Medium by gentle trituration with a 10 mL pipette, transferred the cell suspension to a conical tube with Support Medium, and centrifuged at 300 x *g* for 5 minutes. Finally, to check concentration and viability, we analyzed resuspended cells using trypan blue staining and Countess II Automated Cell Counter (TFS).

2.3. Serial scRNA-seq Studies Using 10x Genomics Platform

For Sample Set-A (n=4 samples, CA1-A unpaired: D02, D04, D09A, D30), we harvested cells from two wells of one 12-well plate using TrypLE Select Enzyme and filtered using a 35 µM cell strainer to create single-cell suspensions with expected cell sizes, 5–10 µm in diameter for early iPSC-CM and 30 µm × 10 µm for late iPSC-CM (>35 days) [5]. We counted cells and checked for viability, washed cells twice using DPBS with 0.04% bovine serum albumin, and recounted cells for the final cell count. We resuspended cells in Eppendorf DNA LoBind tubes (TFS) to a target cell concentration of 7 x 10(5) to 1 x 10(6) cells/mL and cell viability of at least 80%.

For Sample Set-B (n=8 samples, CA1-B and PA1 pairs: D00, D09B, D16, D19), we first harvested iPSC (P15) at ~70-80% confluency from three 12-well plates (36 total wells) and pooled cells for each sample (CA1-B and PA1: D00). We also reseeded iPSC (P16) for CM differentiation. At Day 9, 16, and 19, we harvested cells from six 12-well plates (72 total wells) and pooled cells for each sample (CA1-B: D09B, D16, D19 and PA1: D09B, D16, D19). For Day 16 and 19, we collected cells after metabolic selection by glucose deprivation and lactate supplementation [6, 7] for four days (D10 to D14) using the Gibco enrichment protocol (MAN0014828, TFS).

For scRNA-seq, we transferred cell samples on ice immediately after collection to the UCI Genomics High-Throughput Facility. Sample processing used the droplet-based Chromium system [8] and Chromium Single Cell 3' Gene Expression reagent kits v3 (10X Genomics, Pleasanton, CA). The Facility quantified single-cell cDNA libraries and multiplexed libraries by Illumina paired-end sequencing for 28 cycles for Read 1 (cell barcode + UMI tag), 8 cycles for sample index, and 100 cycles for Read

2 (cDNA insert). Library sequencing involved first, shallow sequencing using HiSeq 4000 with the goal of obtaining 3,000 to 9,000 raw reads per cell to estimate the number of cells captured for each sample, and then, deeper sequencing using NovaSeq 6000 with the goal of obtaining at least 50,000 raw reads per cell. Using Illumina bcl2fastq2 conversion software, the Facility processed raw data files (BCL) for index reads, demultiplexed data into sample specific FASTQ files for Read 1, Read 2, and sample index, and then stored FASTQ sequence files on the UCI High Performance Computing Cluster.

2.4. scRNA-seq Bioinformatics Workflow

Step-I. Data Processing: For this first step in our Workflow (**Fig. 1B**), we processed Single Sample Data using Cell Ranger (10X Genomics) with default parameters for read processing and mapping, transcript and cell quantification, and generation of summary metrics (**Table S4**). Using the 10X genomics: 'cellranger count' pipeline (10X genomics), we processed FASTQ files of each sample to identify high-quality reads with valid cell barcodes and UMIs and mapped these reads to human reference genome (GRCh38-3.0.0) provided by 10x Genomics [9] to produce BAM files. Using the number of confidently mapped reads to the transcriptome in each cell, we quantified transcripts to generate a gene-barcode matrix, an output table of cell barcodes and gene UMI counts defined by matrix.mtx, barcode.tsv, and features.tsv files for each sample. We used both raw unfiltered gene-barcode matrix (raw_feature_bc_matrix) and raw filtered gene-barcode matrix (filtered_feature_bc_matrix) in our analysis. In addition, we used Cell Ranger output BAM files (possorted_genome_bam.bam and possorted_genome_bam.bam.bai) for each sample and the Integrative Genomics Viewer (IGV) [10] to visualize aligned reads to the genome and to evaluate expressed Single Nucleotide Variants (SNV). We reported results of initial analysis for two samples, CA1-B and PA1 at Day 19 [2].

To obtain high-quality cells for clustering and downstream analysis, we conducted QC processing for Single Sample Data in three consecutive stages to identify and remove background RNA contamination, low-quality cells, and cell doublets (**Fig. 1B**). We used R and RStudio version for Windows to run three software packages at each stage: A. SoupX [11]; B. Seurat [12]; and C. DoubletFinder [13] (**Fig. 1B**). For each sample, QC processing generated four types of data matrices (gene-barcode matrices): 1. Raw Data Matrix (Cell Ranger filtered gene-barcode matrix), 2. Corrected Data Matrix (SoupX background-corrected gene-barcode matrix), 3. Filtered Data Matrix (Seurat gene-barcode matrix after removal of low-quality cells), 4. Singlet Data Matrix (DoubletFinder gene-barcode matrix after removal of doublet cells). To visualize effects of QC processing at each stage, we compared pre- and post-processing data by examining QC covariates and/or gene expression using frequency

histograms, DimPlot, FeaturePlot, and VlnPlot functions in Seurat. The parameters and results at each stage are provided for Single Sample (**Table S5**) and Combined Data (**Table S6**).

The first stage of QC processing used SoupX [11] to process Raw Data Matrix into Corrected Data Matrix for each sample by estimating levels of background RNAs in each sample from empty droplets, estimating cell-specific contamination fraction (rho) using clustering information and negative gene markers, and producing a background-corrected gene-barcode matrix for each of the 12 samples. First, we processed the Raw Data Matrix (filtered_feature_bc_matrix) into a data object using the standard clustering pipeline in Seurat (see below). We then used the SoupX 'SoupChannel' function to create an object from the table of counts (toc) and the table of droplets (tod), derived from the filtered_feature_bc_matrix and unfiltered raw_feature_bc_matrix Cell Ranger output files, respectively, and to characterize the background RNAs levels (soup expression profile). Next, we estimated contamination fraction (rho), the fraction of UMIs originating from the background in each cell using two methods in SoupX: the automatic 'autoEstCont' function and the manual 'calculateContaminationFraction' function by providing a selected list of cell type-specific negative gene markers (expected non-expressed genes in at least one cluster for the sample). To remove a greater portion of the contamination, we used the greater rho value from the two methods. Possible overcorrection for background contamination likely has minimal negative effects [11]. For Day 0, we did not conduct manual calculation because the iPSC clusters had similar expression profiles and lacked appropriate negative marker genes; thus, we used the automatically estimated global rho value. For CA1 Day 0, this value was low (0.01) with low complexity (<10 marker genes found); therefore, the greater rho value (0.13) for PA1 Day 0 was used. Finally, we applied 'adjustCounts' to produce a background-corrected gene-barcode matrix (Corrected Data Matrix).

The second stage of QC processing used Seurat [12] to process Corrected Data Matrix into Filtered Data Matrix for each sample by identifying characteristics of low-quality cells using three common QC covariates: nFeature (numbers of genes detected), nCount (the number of UMIs), and %Mt (percentage of reads mapped to mitochondrial (Mt) genome) [14]. For each sample, we evaluated distributions of QC covariates for each cell using frequency histograms, scatter plots, and violin plots [14]. To excluded low-quality cells [15], we selected appropriate thresholds separately for each sample by visual inspection for outlier peaks to filter out outlier cells that may represent dying cells with small numbers of genes (nFeature) or potential doublet cells with high number of UMIs (nCount). To filter out low-quality cells that may represent broken cells [15], thresholds for high %Mt reads also are used. However, since %Mt reads can be variable depending on cell type and energy requirements with heart

having a higher Mt content [16, 17], we decided to use thresholds for %Mt reads only after clustering and cell annotation to data subsets with Non-CM cells.

The third stage of QC processing used DoubletFinder (DF) [13] to process Filtered Data Matrix into Singlet Data Matrix for each sample by predicting gene expression features of simulated doublet cells, classifying cells as either singlet and doublet, and removing heterotypic doublets. First, we processed the Filtered Data Matrix (high-quality cells) into a data object using the standard clustering pipeline in Seurat (see below). Next, we used the DF 'paramSweep_v3' function (PCs = 1:30) to estimate optimal pK values for each sample. Based on number of cells captured for each sample (**Table S4**), we selected values for expected proportion of doublets (Exp) from the Multiplet Rate Table provided in the User Guide for the Single Cell 3' Gene Expression v3 assay (CG000204, 10X Genomics). Based on estimated number of cells loaded for each sample, we calculated number of expected doublets (nExp). We adjusted this number (nExp-adj) using the DF 'modelHomotypic' function to estimate proportion of homotypic doublets. Finally, we used these parameters in doubletFinder_v3 (pN = 0.25) to classify each cell as either a predicted singlet or doublet cell and removed Doublet cells to generate post-DF data of high-quality cells (Singlet Data Matrix).

<u>Step-II. Data Analysis- Cluster, Annotate, and Subset</u>: For this second step in our Workflow (**Fig. 1B**), we focused on determining cell identities for each sample in two stages: A. Individual Analyses of Singlet Data for Main Cell Types and B. Subcluster Analyses of Subset Data for Possible Cell Subtypes. For both stages, we used the standard clustering analysis workflow in Seurat: data normalization, calculation of variable features, data scaling with and without regressing out potential confounding variables (regression covariates), dimensionality reduction by Principal Component Analysis (PCA), unsupervised clustering, and visualization. We used the functions (parameters): 'NormalizeData' (method = "LogNormalize"), 'FindVariableFeatures' (nfeatures = 3000), 'ScaleData' (vars.to.regress), 'RunPCA' (npcs = 50), 'FindNeighbors', 'FindClusters,' and 'RunUMAP.' For dimension value (dims), we used 1:30 for Single Sample Data and 1:50 for the larger Combined Data. We tested a range of values for clustering resolution and chose final values on results with similar clusters and cell types between paired samples and on comparable results described in previous scRNA-seq studies [18-23]. The clustering parameters used are provided for Single Sample Data (**Table S7**).

To evaluate potential confounding biological factors (regression covariates), our analysis also included cell quantification of cell cycle (CC) phase and Mt content [16, 24-26]. For each cell, we calculated scores for CC phase ("G2M.Score" and "S.Score") using the Seurat 'CellCycleScoring' function based on expression of 97 canonical G2/M-phase and S-phase markers [12, 25]. Using CC scores, we categorized cells as G2/M-phase, S-phase, G1-phase. For each cell, we calculated %Mt

using the Seurat ‘PercentageFeatureSet’ function. Using quartiles values calculated by the ‘summary’ function in R [27], cells were categorized as "Low", "Medium", "Medium high", or "High" %Mt. After categorization for each sample, we corrected data using the function ‘ScaleData’ (vars.to.regress) to regress out %Mt, CC Scores, and both covariates and compared clustering results with and without covariate regression. The covariates used are provided for Single Sample Data (**Table S7**).

After covariate regression and clustering, we annotated clusters by Individual Analyses of Singlet Data for Main Cell Types using expression panels of known markers (**Table S8**) and Cluster DEG in Seurat. We used a Primary Marker Panel (25 genes) to identify the main cell type and Expanded Marker Panels (up to 38 genes) to confirm cell type and evaluate for possible cell subtypes among similar clusters. These Marker Panels consisted of genes expressed in reported cell types and subtypes during iPSC-CM differentiation and maturation [28-32] and in previous scRNA-seq studies for iPSC [33], endoderm [34, 35], iPSC-CM or hES-CM [18-23, 36], and human tissues [37]. To detect residual undifferentiated cells, we included the *CNMD* gene [38]. After cell annotation, we confirmed our results by evaluating Cluster DEG for known cell type markers and signatures by gene set enrichment analysis (see below). First, we found Cluster DEG using ‘FindAllMarkers’ (test.use= “wilcox”, logfc.threshold = 0.25, min.pct = 0.1) and then ranked top DEG for each cluster by log fold-change of the average expression relative to all other clusters. We compared and visualized cluster expression for known markers and top DEG using DimPlot, VlnPlot, FeaturePlot, and DoHeatmap functions in Seurat. If cell type was not clearly established after evaluation, we annotated the cluster as “Unknown.”

After annotation for main cell types, we conducted Subcluster Analyses of Subset Data for Possible Cell Subtypes, using similar steps in our Individual Analyses as described above. First, we divided annotated Single Sample Data into separate subsets by cell type(s) using the ‘subset’ function in Seurat. We separated CM, Non-CM, and Unknown cells into different subsets for cell type-specific QC processing and removal of Unknown cells prior to data integration and Comparative Analyses. Our subset data analysis involved: 1. Evaluation for common QC covariates; 2. Cell type-specific QC processing that used high threshold levels for %Mt to identify low-quality cells in Non-CM cells; 3. Correction by regressing out CC Scores that might confound trajectory inference [14]; 4. Unsupervised clustering using the standard clustering pipeline in Seurat; and 5. Cell annotation using Marker Panels (**Table S8**) and Subcluster DEG. In addition, we used two Cell Subtype Marker Panels to identify possible CM subtypes (27 genes for ventricular, atrial, and nodal cells) [18, 30, 39] and EPDC subtypes (27 genes for EPDC Progenitors, Cardiac Fibroblast, Vascular Smooth Muscle, and Angioblasts/Endothelial cells) [20, 23, 30] (**Table S8**). For Unknown cells, we classified cells into possible subtypes using Marker Panels, Subcluster DEG, and common QC covariates: nFeature,

nCount, and %Mt. The parameters for cell type-specific QC processing and subclustering are provided for Single Sample Data (**Table S7**).

Step-III. Data Combining and Comparative Analyses: For the third step in our Workflow (**Fig. 1B**), we created two types of Combined Data from the 12 samples: Integrated Data primarily to compare paired samples, Patient versus (vs.) Control at D00, D09B, D16, and D19, by cell type and lineage differential gene expression and pathway enrichment and Merged Data (Non-Integrated) to summarize and evaluate our results. To evaluate data integration, we compared clustering results between Merged Data without integration and Integrated Data with integration using Seurat. In Combined Data, we also compared the "imbalance score" for each cell to test whether nearby cells have the same condition using the 'imbalance_score (k = 20, smooth = 40)' function in Condiments [40]. We defined 'balanced' cell types and subtypes as shared cells with low imbalance scores compared to the other cells in Combined Data. The parameters are provided for integration and evaluation of Combined Data for Paired Sample Data and Subset Data (**Table S9**).

To identify 'balanced' cell types/subtypes for Comparative Analyses between conditions, we first analyzed Combined Data of paired samples (D00, D09B, D16, and D19). Like our analyses for Single Sample Data, we conducted both Individual and Subcluster Analyses of Combined Data of Paired Sample Data (**Table S9**). First, we created Merged Data using the 'merge' function to combine two processed Seurat objects from CA1 and PA1: Paired Sample Data (Singlet) (n=4 pairs) or Paired Subset Data (n=11 pairs). Next, we created Integrated Data (**Table S9**) first by splitting Merged Data into a list of objects by condition (Patient and Control) using the 'SplitObject' function (split.by = "orig.ident"). After normalization, we applied the standard canonical correlation analysis (CCA) integration pipeline [12]: CCA to identify shared variation between conditions, identification of "anchors," and alignment of Control and Patient cells into an integrated Seurat object. We used the functions (parameters): 'SelectIntegrationFeatures' (nfeatures = 3000), 'FindIntegrationAnchors,' and 'IntegrateData.' After standard clustering analysis, we annotated as described above using Marker Panels (**Table S8**) and Cluster DEG. For Integrated Data, we identified Cluster DEG conserved between conditions using the function 'FindConservedMarkers' (grouping.var = "Sample", test.use = "wilcox", logfc.threshold = 0.25, min.pct = 0.1). We ranked Cluster DEG by average fold change of expression (avg_fc = (PAT_avg_log2FC + CTRL_avg_log2FC) /2)), visualized top Cluster DEG using 'DoHeatmap' in Seurat, and confirmed cell types by Over-Representation Analysis (ORA) [41] as described below.

To identify cell type-specific differentially expressed genes (Cell Type DEG) between conditions, we conducted Comparative Analyses (Patient vs. Control) in 'balanced' cell subtypes of Integrated

Data. First, we identified all Cell Type DEG between conditions using the function 'FindMarkers' (ident.1 = "patient", ident.2 = "control", test.use= "wilcox", logfc.threshold = 0). Next, we divided Cell Type DEG by direction of change in Patient cells compared to Control cells: overexpressed genes (positive DEG, logfc.threshold > 0) and underexpressed genes (negative DEG, logfc.threshold < 0). We identified and ranked top Cell Type DEG using chosen thresholds for statistical significance and difference in average expression (adjusted p-value < 10e-50 and I Average Log2 FoldChange I > 0.25 (Fold Change = 1.2x)). We visualized and compared Cell Type DEG using 'EnhancedVolcano' [42] and 'VennDiagram' [43].

To identify biological process or pathways altered between conditions, we evaluated Cell Type DEG by enrichment analysis using two common approaches: Over-Representation Analysis (ORA) [41] and Gene Set Enrichment Analysis (GSEA) [44]. For each cell subtype, we tested individually the lists of threshold DEG (overexpressed or underexpressed genes with minimum DEG = 3) for overrepresentation in the Gene Ontology (GO) biological processes gene sets (n = 7751) [45] using the 'EnrichGo' function (adjusted p-value < 0.05, q-value cutoff = 0.20) in Cluster Profiler [46]. To evaluate all DEG (selected without a threshold), we first ordered the complete lists of DEG (overexpressed and underexpressed genes) by GSEA metric (= -log10(p_val_adj) * sign(avg_log2FC)). We then tested ranked lists for enrichment in the Molecular Signatures Database (MSigDB) Hallmark gene sets (n = 50) [44, 47] using the 'GSEA' function (GSEA (minGSSize = 10, maxGSSize = 500, eps = 1e-10, p-value Cutoff = 0.05) in Cluster Profiler [46]. We visualized enrichment results using 'dotplot', 'emapplot', and 'gseaplot2' in Cluster Profiler [46] and used Module Scoring with 'AddModuleScore' function in Seurat to quantify, compare, and visualize patterns of expression of key DEG in significant ORA and GSEA gene sets.

To evaluate lineage-specific differential expression, we conducted trajectory inference using Slingshot [48] and expression analysis using TradeSeq [49]. To determine possible cell lineages in each sample, we first analyzed Single Subset Data (**Table S10**) for selected cell subtypes and adjacent subtypes of one condition (Control or Patient). First, we converted each processed object to a SingleCellExperiment (SCE) data class using the 'as.SingleCellExperiment' function in Seurat. Next, in Slingshot, we conducted trajectory inference with semi-supervision with 'slingshot' or 'getLineages' and 'getCurves' (reducedDim = 'UMAP', approx_points = FALSE, stretch = 0, extend = "n") and 'slingMST.' These functions identified lineage topology (global lineage structure) by constructing a minimum spanning tree (MST) on clusters, fitted a principal curve through data that defined a trajectory, and calculated a pseudotime variable for each cell. For each sample, we selected starting (start.clus) and ending (end.clus) clusters based on progression of cell types and subtypes during iPSC-CM differentiation [28, 29] and cell lineages reported in previous scRNA-seq studies for iPSC [33],

endoderm [35], and iPSC-CM or hES-CM [19, 21, 23, 36, 50, 51]. We visualized results in UMAP plots generated using 'ggplot' and base R 'plot' and density plots for progression.

Next, to identify differential expressed genes within each lineage (Lineage DEG), we analyzed SCE objects in TradeSeq [49] (**Table S10**). First, we used the 'fitGAM' function to make a general additive model (GAM) of relationship between expression of each gene and pseudotime. Using the 'evaluateK' function (k = 3:10, nGenes = 200), we selected the number of knots. We tested genes for two types of Lineage DEG (Threshold: FDR <0.05 & Fold Change > 2x): Association Test (AT) DEG and Start-End Test (SET) DEG. Using the 'associationTest' function, we identified AT Lineage DEG for which average gene expression significantly changed along pseudotime of the lineage. Using the 'startVsEndTest' function, we identified SET Lineage DEG for which average gene expression significantly changed between starting and ending pseudotime points of the lineage. For samples with a bifurcating trajectory, we identified Global AT and SET Lineage DEG by testing across both lineages (global = TRUE). We visualized and compared expression patterns of top Lineage DEG and known marker genes using 'pheatmap' and Tradeseq functions 'plotSmoothers' and 'plotGeneCount'.

After integrating Paired Subset Data (**Table S11**), we conducted Comparative Analyses for lineage differential expression across conditions (Patient vs. Control) and pathway enrichment analyses. As described above for Single Subset Data, we analyzed Integrated Subset Data for trajectory inference and lineage differential expression using Slingshot [48] and Tradeseq [49] (**Table S11**). Using Condiments [40], we then tested for differences in lineage topology, progression, differentiation, and expression between conditions. Using the 'topologyTest' (rep = 100) function, we tested for differential topology: different individual trajectories for each condition. Using 'progressionTest' (global = TRUE, lineages = TRUE), we tested for differential progression: different distributions of cells found along the trajectories for each condition. For bifurcating trajectories, we used 'differentiationTest' (global = TRUE, pairwise = TRUE) for differential differentiation: different cell distributions between lineages for each condition.

For Integrated Subset Data (**Table S11**), we then tested for two types of Lineage DEG (Threshold: FDR <0.05 & Fold Change > 2x): Association Test (AT) and Condition Test (CT) Lineage DEG. Using the 'associationTest' function, we identified AT Lineage DEG for which average gene expression significantly changed along pseudotime of the lineage for both conditions. Using the 'conditionTest', we identified CT Lineage DEG for which average gene expression significantly changed along pseudotime of the lineage between conditions. For bifurcating trajectories, we tested for differential expression between conditions to identify Global AT and CT Lineage DEG that changed for both lineages (global = TRUE) and CT Lineage-Specific DEG that changed in only one lineage (lineages = TRUE).

As described above for Cell Type DEG, we conducted enrichment analyses for Lineage DEG by ORA [41] using Cluster Profiler [46] to identify biological processes or pathways that changed with pseudotime in Single Subset Data (**Table S10**) and Integrated Subset Data (**Table S11**). We conducted ORA using top 100 AT, SET, and CT Lineage DEG (ranked by Wald statistic) and smaller groups of DEG obtained by hierarchical cluster analysis using the functions 'hclust' and 'cutree' (k=2 to 4). In addition, like Cell Type DEG, we evaluated all CT Lineage DEG (FDR <0.05 & WaldStat > 0.0) using GSEA (scoreType = "pos") for enrichment of MSigDB Hallmark gene sets [44, 47] in Cluster Profiler [46].

2.5. Lamin A/C Western Blot

Cell samples were collected and lysed using RIPA Lysis and Extraction Buffer with Inhibitor Cocktail (TFS) at a ratio of 100 µL for every 1,000,000 cells. Using the Pierce BCA Protein Assay Kit (TFS), we determined protein concentration of cell lysate. We combined 20 µg of total protein lysate, Bolt LDS Sample Buffer and Reducing Agent (TFS), and distilled water in 30 µL total that was heated and loaded on to Bolt 4-12% Bis-Tris Plus Gels (TFS). After gel electrophoresis, we used the Mini Blot Module (TFS) to transfer proteins to a PVDF membrane that was processed using the iBind Western Device and iBind Fluorescent Detection solutions (TFS). To detect target proteins, we used primary antibodies for the Lamin A/C N-terminus (sc-376248, Santa Cruz Biotechnology, Dallas, TX) and Beta-Actin as the internal loading control and fluorophore-conjugated secondary antibodies for visualization (**Table S2**). As a positive control for Lamin A/C expression, we included protein lysate from Control (CA1) fibroblast. We repeated gel electrophoresis and immunoblotting to produce three technical replicates (TR).

For protein visualization and quantification of band signal, we used the Azure c600 imaging system and AzureSpot software (Azure Biosystems, Dublin, CA). For each TR blot, we first determined protein levels as normalized ratios (NR) of Lamin A, Lamin C, and total Lamin A+C protein for each individual sample (n=6: CA1-B, U2, CA3, PA1, PA2, PA3) by dividing the Lamin A/C band volume (BV) with the corresponding Beta-Actin BV. From the three TR blots, we then calculated mean protein levels (Mean NR of TR) of Lamin A, Lamin C, and Lamin A/C for each sample, and from the three biological replicates (BR), we calculated mean protein levels (Mean NR of BR) for Control samples (n=3: CA1-B, U2, CA3) and Patient samples (n=3: PA1, PA2, PA3). We also compared Patient samples pairwise to biologically (sex and age) matched Control samples (PA1 to CA1, PA2 to U2, PA3 to CA3) using Relative Normalized Ratio (RNR) by dividing the Lamin A/C protein level (NR) of the Patient sample with the Control sample to calculate mean fold change (FC) and percent change (% change) of TR. To evaluate

technical variability between blots for pairwise comparisons, we calculated coefficient of variation (CV= SD of TR/ Mean FC of TR) and % change/CV. For statistical comparisons of Patient and Control data, we used the independent samples t-test and defined significance as $p < 0.05$.

3. Results

3.2. Processed data: 110,521 (88%) high-quality cells of 125,554 cells collected (Workflow Step-I).

First, we processed FASTQ sequence files for the 12 cell samples using Cell Ranger to obtain summary metrics for read mapping and cell/gene quantification (**Table S4**). The metrics showed that deep sequencing estimated 125,554 total cells captured with 40,978 mean reads per cell and 93.2% Reads Mapped Confidently to Genome. The eight Control samples had greater total number of captured cells with similar values for % Reads Mapped compared to the four Patient samples. These results are consistent with high-quality sequencing data [52] and sufficient read depth for an initial analysis to identify main cell types in both Control and Patient cell samples [53]

Next, we conducted QC processing for Single Sample Data in three consecutive stages to identify and remove background RNA contamination using SoupX, low-quality cells using Seurat, and doublet cells using DoubletFinder that generated four types of data (gene-barcode matrices): 1. Raw Data Matrix, 2. Corrected Data Matrix, 3. Filtered Data Matrix, 4. Singlet Data Matrix for each sample (**Table S6**) and for Combined Data (**Fig. S3**). QC processing of Raw Data Matrices identified 110,521 (88%) high-quality cells of 125,554 total cells and similar proportions of high-quality cells in eight Control samples and four Patient samples (**Fig. S3A**). Of 15,032 (12%) total cells removed, 8,272 (55%) were identified as low-quality cells and 6,761 (45%) as doublet cells. By comparing Merged Data for all 12 samples after each stage, our results demonstrate significant effects of QC processing not only in the total number of cells at each stage but also in clustering and gene expression patterns after background RNA removal (**Fig. S3E**) and in distributions of QC covariates (nFeature, nCount, and %Mt) between different samples and clusters (**Fig. S3BCD**), between low-quality and high-quality cells (**Fig. S3F**), and between singlet and doublet cells (**Fig. S3F**). For example, although Control and Patient samples had similar median values of the QC covariates for each cell (**Fig. S3B**), distributions of QC covariates varied by sample (**Fig. S3C**) and by cluster (**Fig. S3D**). Overall, these results emphasize the importance of comprehensive QC processing with assessment after each stage and using parameters that were determined separately for each sample [14] (**Table S5**) and for different cell types (CM vs. Non-CM) (**Table S7**).

3.3. Complex heterogeneity with ten main cell types in Control samples, eight shared cell types between paired samples, and multiple possible cell subtypes (Workflow Step-II).

Overall, cell type heterogeneity increased during early iPSC differentiation with all ten cell types identified from Day 0 to Day 9; thereafter, CM and EPDC became the predominant cell types (**Fig. S5**). At Day 0, PP cells (89%) served as the predominate cell type with expression of three marker genes, *POU5F1*, *SOX2*, and *NANOG*. At Day 2, three early differentiated cell types (96%) predominated that were identified by marker expression of *EOMES* for ME, *MESP1* for CMESO, and *SOX17* for ENDO. At Day 4, nearly all cells (98%) were identified as either PP, CMESO, ENDO, or CP with expression of *HAND1*, *HAPLN1*, and *TMEM88* to mark CP cells. At Day 9, differentiation of two independent clones (CA1-A and CA1-B) showed increased cell heterogeneity with most cells (69-88%) as CP cells or CP-derived cells (CM-A, CM/UNK-B, and EPDC) with multiple marker expression that included *MYL7*, *TNNI1*, and *NKX2-5* for CM and *COL3A1*, *LUM*, and *FBN1* for EPDC. Day 9 also consisted of ENDO cells with *FOXA2* expression and ENDOTH cells with *EGFL2* expression, two cell types derived from both clones and ECTO cells with *PAX6* expression, a cell type derived from one clone (CA1-B). At Day 16 and Day 19 (post-metabolic selection), we identified most cells (~75%) as CM (CM-A and CM/UNK-B) and a smaller subset as EPDC (13%). At Day 30 (without metabolic selection), these two types of cells comprised most cells (82%); however, CM (38%) were found in a lower proportion compared to EPDC (44%). For all samples, we confirmed cell type by finding known marker genes among top DEG for the cluster (Cluster DEG) (**Fig. S5**, **Table S7**, **Excel Table S1-1**).

In our Subcluster Analyses, we subsetted Single Sample Data into 32 total subsets and found 30 total possible subtypes in eight cell types (PP, ME, ENDO, ECTO, CP, CM, EPDC, UNK) (**Fig. S4A**, **Fig. S6**, **Table S7**, **Excel Table S1-2**). For eight paired samples (D00, D09B, D16, D19), we found 19 possible subtypes in six cell types (PP, ECTO, CP, CM, EPDC, UNK): 13 subtypes conserved in both conditions and 6 subtypes in one condition.

For Control samples, both CM and EPDC showed maturation and then differentiation into cell subtypes. For Control CM, we found four possible subtypes: an early pair (CM-A, CM/UNK-B) at Days 9 to 19 and a further differentiated pair: Atrial CM (ATR-CM) and Ventricular CM (VENTR-CM) at Day 30 (**Fig. S6B**). Similarly, for Control EPDC, we found seven possible subtypes: an early set at Day 16 (EPDC-A, EPDC-B, EPDC-C) and a further differentiated set: Epicardial Progenitor (EPI), Cardiac Fibroblast (CFIBRO), Vascular Smooth Muscle (VSM), and an Unspecified EPDC subtype (EPDC-UNSP) at Day 30 (**Fig. S6B**). From Days 9 to 19, both CM and EPDC had results consistent with cell type maturation as increased proportions of cells with expression of later markers (CM: *TTN*, *MYH7*, *MYL2;* EPDC: *COL3A1*, *LUM*, *FBN1*) compared to early markers (CM: *MYL7*, *TNNI1*, *NKX2-5*; EPDC:

TBX18, *TCF21*, *WT1*). At Day 30, early subtypes were not found; instead, we identified the six later differentiated subtypes: ATR-CM, VENTR-CM, EPI, CFIBRO, VSM, an Unspecified EPDC subtype with differentiating patterns of gene expression in CM and EPDC Subtype Marker Panels (**Fig. S6B**).

For eight paired Control and Patient samples, we found 19 total possible subtypes in six cell types (PP, ECTO, CP, CM, EPDC, UNK): 13 subtypes conserved and six subtypes in one condition (**Fig. S6**). For PP and CM, we found only conserved subtypes. Both Control and Patient PP cells (D00: Subset-A) had four possible subtypes: PP-A (88-92%), PP-B, PP-C, and PP-D with variable expression levels of ten PP marker genes (*POU5F1*, *SOX2*, *NANOG*, *DNMT3B*, *NODAL*, *UTF1*, *LIN28A*, *LEFTY1*, *GDF3*, and *SDC2*) and one undifferentiated cell marker: *CMND*. Likewise, both Control and Patient early CM (D09B: Subset-A) and post-metabolic selection CM (D16 and D19: Subset-A) had two possible subtypes (CM-A and CM/UNK-B) with distinguishing features as seen in our Individual Analyses. In addition to lower expression levels of Mt DNA genes, CM-A cells had higher expression of Ribosomal Protein Genes (*RPL* and *RPS*) compared to CM/UNK-B cells.

In contrast to PP and CM, we found subtype differences for four cell types: CP, EPDC, ECTO, and UNK between paired Control and Patient samples (**Fig. S6**). While both Control and Patient CP cells (D09B: Subset-A) had two possible subtypes (CP-A and CP-B), we identified a third subtype (CP-C) in only the Patient sample using nine CP marker genes (*HAND1*, *HAPLN1*, *TMEM88*, *GATA4*, *HCN4*, *TBX5*, *ISL1*, *TBX1*, and *HAND2*). Likewise, both Control and Patient EPDC (D16: Subset-B) had two possible subtypes (EPDC-A and EPDC-B) and a third subtype (EPDC-C) in only the Patient sample. For ECTO (D09B: Subset-B), we identified four possible subtypes: ECTO-A, ECTO-B, ECTO-C, ECTO-D with later two subtypes in only Patient cells; however, these subtypes each had low cell number (~130 to 250 cells) using only two ECTO markers: *PAX6*, *SOX1*. For Unknown cells, we found three possible subtypes: UNK-A, UNK-B, and UNK-C using levels of covariates and marker expression. We defined UNK-A as cells with low nCount levels, UNK-B as cells with low nCounts levels and highest %Mt levels, and UNK-C as cells with higher nCount and Marker expression levels compared to the other possible subtypes. While both Control and Patient cells (D00: Subset-B and D09B, D16, D19: Subset-C) had the UNK-A subtype, the other two subtypes had subsets with only Control cells (UNK-B D16:Subset-C) or Patient cells (UNK-C D16 and D19: Subset-C).

References

1. Morival, J.L.P., et al., *DNA methylation analysis reveals epimutation hotspots in patients with dilated cardiomyopathy-associated laminopathies.* Clin Epigenetics, 2021. **13**(1): p. 139.
2. Mehrabi, M., et al., *A Study of Gene Expression, Structure, and Contractility of iPSC-Derived Cardiac Myocytes from a Family with Heart Disease due to LMNA Mutation.* Ann Biomed Eng, 2021. **49**(12): p. 3524-3539.

3. Allen Institute for Cell Science. *Cardiomyocyte Differentiation Method, Version 1.0. Available from: https://www.allencell.org/methods-for-cells-in-the-lab.html*. 2018.
4. Lian, X., et al., *Directed cardiomyocyte differentiation from human pluripotent stem cells by modulating Wnt/β-catenin signaling under fully defined conditions.* Nat Protoc, 2013. **8**(1): p. 162-75.
5. Robertson, C., D.D. Tran, and S.C. George, *Concise review: maturation phases of human pluripotent stem cell-derived cardiomyocytes.* Stem Cells, 2013. **31**(5): p. 829-37.
6. Tohyama, S., et al., *Distinct metabolic flow enables large-scale purification of mouse and human pluripotent stem cell-derived cardiomyocytes.* Cell Stem Cell, 2013. **12**(1): p. 127-37.
7. Burridge, P.W., et al., *Chemically defined generation of human cardiomyocytes.* Nat Methods, 2014. **11**(8): p. 855-60.
8. Zheng, G.X., et al., *Massively parallel digital transcriptional profiling of single cells.* Nat Commun, 2017. **8**: p. 14049.
9. 10X Genomics. *Cell Ranger Build Notes for References - 3.0.0 at support.10xgenomics.com/single-cell-gene-expression/software/release-notes/build*. 2018.
10. Robinson, J.T., et al., *Integrative genomics viewer.* Nat Biotechnol, 2011. **29**(1): p. 24-6.
11. Young, M.D. and S. Behjati, *SoupX removes ambient RNA contamination from droplet-based single-cell RNA sequencing data.* Gigascience, 2020. **9**(12).
12. Stuart, T., et al., *Comprehensive Integration of Single-Cell Data.* Cell, 2019. **177**(7): p. 1888-1902.e21.
13. McGinnis, C.S., L.M. Murrow, and Z.J. Gartner, *DoubletFinder: Doublet Detection in Single-Cell RNA Sequencing Data Using Artificial Nearest Neighbors.* Cell Syst, 2019. **8**(4): p. 329-337.e4.
14. Luecken, M.D. and F.J. Theis, *Current best practices in single-cell RNA-seq analysis: a tutorial.* Mol Syst Biol, 2019. **15**(6): p. e8746.
15. Ilicic, T., et al., *Classification of low quality cells from single-cell RNA-seq data.* Genome Biol, 2016. **17**: p. 29.
16. Osorio, D. and J.J. Cai, *Systematic determination of the mitochondrial proportion in human and mice tissues for single-cell RNA-sequencing data quality control.* Bioinformatics, 2021. **37**(7): p. 963-967.
17. Subramanian, A., et al., *Biology-inspired data-driven quality control for scientific discovery in single-cell transcriptomics.* Genome Biol, 2022. **23**(1): p. 267.
18. Churko, J.M., et al., *Defining human cardiac transcription factor hierarchies using integrated single-cell heterogeneity analysis.* Nat Commun, 2018. **9**(1): p. 4906.
19. Friedman, C.E., et al., *Single-Cell Transcriptomic Analysis of Cardiac Differentiation from Human PSCs Reveals HOPX-Dependent Cardiomyocyte Maturation.* Cell Stem Cell, 2018. **23**(4): p. 586-598.e8.
20. D'Antonio-Chronowska, A., et al., *Association of Human iPSC Gene Signatures and X Chromosome Dosage with Two Distinct Cardiac Differentiation Trajectories.* Stem Cell Reports, 2019. **13**(5): p. 924-938.
21. Ruan, H., et al., *Single-cell reconstruction of differentiation trajectory reveals a critical role of ETS1 in human cardiac lineage commitment.* BMC Biol, 2019. **17**(1): p. 89.
22. Grancharova, T., et al., *A comprehensive analysis of gene expression changes in a high replicate and open-source dataset of differentiating hiPSC-derived cardiomyocytes.* Sci Rep, 2021. **11**(1): p. 15845.
23. Floy, M.E., et al., *Direct coculture of human pluripotent stem cell-derived cardiac progenitor cells with epicardial cells induces cardiomyocyte proliferation and reduces sarcomere organization.* J Mol Cell Cardiol, 2022. **162**: p. 144-157.
24. Wagner, A., A. Regev, and N. Yosef, *Revealing the vectors of cellular identity with single-cell genomics.* Nat Biotechnol, 2016. **34**(11): p. 1145-1160.

25. Tirosh, I., et al., *Dissecting the multicellular ecosystem of metastatic melanoma by single-cell RNA-seq.* Science, 2016. **352**(6282): p. 189-96.
26. M, P., et al. *hbctraining/scRNA-seq_online: scRNA-seq Lessons from HCBC (first release). Zenodo. //doi.org/10.5281/zenodo.5826256*. 2022.
27. Piper, M., et al. *hbctraining/scRNA-seq_online: scRNA-seq Lessons from HCBC (first release). Zenodo. //doi.org/10.5281/zenodo.5826256*. 2022.
28. Lian, X., et al., *Robust cardiomyocyte differentiation from human pluripotent stem cells via temporal modulation of canonical Wnt signaling.* Proc Natl Acad Sci U S A, 2012. **109**(27): p. E1848-57.
29. Burridge, P.W., et al., *Production of de novo cardiomyocytes: human pluripotent stem cell differentiation and direct reprogramming.* Cell Stem Cell, 2012. **10**(1): p. 16-28.
30. Edgar, R., et al., *LifeMap Discovery™: the embryonic development, stem cells, and regenerative medicine research portal.* PLoS One, 2013. **8**(7): p. e66629.
31. Yang, X., L. Pabon, and C.E. Murry, *Engineering adolescence: maturation of human pluripotent stem cell-derived cardiomyocytes.* Circ Res, 2014. **114**(3): p. 511-23.
32. Später, D., et al., *How to make a cardiomyocyte.* Development, 2014. **141**(23): p. 4418-31.
33. Nguyen, Q.H., et al., *Single-cell RNA-seq of human induced pluripotent stem cells reveals cellular heterogeneity and cell state transitions between subpopulations.* Genome Res, 2018. **28**(7): p. 1053-1066.
34. Biddy, B.A., et al., *Single-cell mapping of lineage and identity in direct reprogramming.* Nature, 2018. **564**(7735): p. 219-224.
35. Cuomo, A.S.E., et al., *Single-cell RNA-sequencing of differentiating iPS cells reveals dynamic genetic effects on gene expression.* Nat Commun, 2020. **11**(1): p. 810.
36. Galdos, F.X., et al., *Combined lineage tracing and scRNA-seq reveals unexpected first heart field predominance of human iPSC differentiation.* Elife, 2023. **12**.
37. Karlsson, M., et al., *A single-cell type transcriptomics map of human tissues.* Sci Adv, 2021. **7**(31).
38. Sekine, K., et al., *Robust detection of undifferentiated iPSC among differentiated cells.* Sci Rep, 2020. **10**(1): p. 10293.
39. Zhao, M.T., N.Y. Shao, and V. Garg, *Subtype-specific cardiomyocytes for precision medicine: Where are we now?* Stem Cells, 2020. **38**(7): p. 822-833.
40. Roux de Bézieux, H., et al., *Trajectory inference across multiple conditions with condiments.* Nat Commun, 2024. **15**(1): p. 833.
41. Huang, d.W., B.T. Sherman, and R.A. Lempicki, *Bioinformatics enrichment tools: paths toward the comprehensive functional analysis of large gene lists.* Nucleic Acids Res, 2009. **37**(1): p. 1-13.
42. Blighe, K., S. Rana, and M. Lewis. *EnhancedVolcano: Publication-ready volcano plots with enhanced colouring and labeling. R package version 1.18.0, //github.com/kevinblighe/EnhancedVolcano.* 2023.
43. Chen, H. and P.C. Boutros, *VennDiagram: a package for the generation of highly-customizable Venn and Euler diagrams in R.* BMC Bioinformatics, 2011. **12**: p. 35.
44. Subramanian, A., et al., *Gene set enrichment analysis: a knowledge-based approach for interpreting genome-wide expression profiles.* Proc Natl Acad Sci U S A, 2005. **102**(43): p. 15545-50.
45. Ashburner, M., et al., *Gene ontology: tool for the unification of biology. The Gene Ontology Consortium.* Nat Genet, 2000. **25**(1): p. 25-9.
46. Wu, T., et al., *clusterProfiler 4.0: A universal enrichment tool for interpreting omics data.* Innovation (Camb), 2021. **2**(3): p. 100141.
47. Liberzon, A., et al., *The Molecular Signatures Database (MSigDB) hallmark gene set collection.* Cell Syst, 2015. **1**(6): p. 417-425.

48. Street, K., et al., *Slingshot: cell lineage and pseudotime inference for single-cell transcriptomics.* BMC Genomics, 2018. **19**(1): p. 477.
49. Van den Berge, K., et al., *Trajectory-based differential expression analysis for single-cell sequencing data.* Nat Commun, 2020. **11**(1): p. 1201.
50. Selewa, A., et al., *Systematic Comparison of High-throughput Single-Cell and Single-Nucleus Transcriptomes during Cardiomyocyte Differentiation.* Sci Rep, 2020. **10**(1): p. 1535.
51. Elorbany, R., et al., *Single-cell sequencing reveals lineage-specific dynamic genetic regulation of gene expression during human cardiomyocyte differentiation.* PLoS Genet, 2022. **18**(1): p. e1009666.
52. 10X Genomics. *Quality Assessment Using the Cell Ranger Web Summary at* www.10xgenomics.com/resources/analysis-guides/quality-assessment-using-the-cell-ranger-web-summary. Analysis Guides 2022.
53. Pollen, A.A., et al., *Low-coverage single-cell mRNA sequencing reveals cellular heterogeneity and activated signaling pathways in developing cerebral cortex.* Nat Biotechnol, 2014. **32**(10): p. 1053-8.
