# Supplementary material for: LMNA-Related Dilated Cardiomyopathy: Single-Cell Transcriptomics during Patient-Derived iPSC Differentiation Support Cell Type and Lineage-Specific Dysregulation of Gene Expression and Development for Cardiomyocytes and Epicardium-Derived Cells with Lamin A/C Haploinsufficiency
Source: Cells. 2024 Sep 3;13(17):1479. doi: 10.3390/cells13171479 (PMC11394257; doi:10.3390/cells13171479)
Supplement: Supplementary file 1 [file cells-13-01479-s001.zip › Zaragoza.Cells.SUPPL.Tables_06.10.2024.pdf]

LMNA-Related Dilated Cardiomyopathy: Single-Cell Transcriptomics during Patient-derived iPSC Differentiation Support Cell type and Lineage-specific Dysregulation of Gene Expression and Development for Cardiomyocytes and Epicardium-Derived Cells with Lamin A/C Haploinsufficiency. M. Zaragoza, T-A. Bui, H. Widyastuti, M. Mehrabi, Z. Cang, Y. Sha, A. Grosberg, Q. Nie; Univ. of California, Irvine, Irvine, CA

Supplemental Tables

		Page
**Table S1.**	**Human subjects and results for fibroblast _LMNA_ sequencing and iPSC validation.**	**2**
**Table S2.**	**Methods: Primary and secondary antibodies.**	**3**
**Table S3.**	**Methods: Main software packages.**	**4**
**Table S4.**	**scRNA-seq Summary Metrics using Cell Ranger.**	**5**
**Table S5.**	**Single Sample Data: QC data processing using SoupX, Seurat, and DoubletFinder.**	**6**
**Table S6.**	**Single Sample and Combined Data: QC processing of Raw to Singlet Data Matrices.**	**7**
**Table S7.**	**Single Sample Data: Individual and Subcluster Analyses for Cell Annotation.**	**8 - 10**
**Table S8.**	**Gene marker panels for cell annotation.**	**11 - 12**
**Table S9.**	**Paired Sample Data: Individual and Subcluster Analyses.**	**13 - 14**
**Table S10.**	**Single Subset Data: Trajectory Inference, Lineage DEG, and Enrichment.**	**15**
**Table S11.**	**Paired Subset Data: Integration, Trajectory Inference, Lineage DEG, and Enrichment.**	**16**
**Table S12.**	**Lamin A/C Western Blot Quantification Data and Statistical Analyses.**	**17 - 19**
	**References:**	**20 - 22**

Table S1. Human subjects and results for fibroblast *LMNA* sequencing and iPSC validation.

Human Subjects			*Fibroblast*		*iPSC*		
**Identification[a]**	**Sex**	**Age At Bx**	***LMNA* Mutation Genotype[b]**	***LMNA* Coding SNV[c]**	**Clone**	**Karyotype (Passage #)[d]**	**ICC-DIFF[e]**
**CONTROLS**							
Control A1 (CA1): IV-2	F	49	+/+	None	CA1-A	46,XX (P13)	Validated
					CA1-B	46,XX (P13)	Validated
Control A2 (CA2): III-2	M	69	+/+	None	CA2[f]	46,XY (P09)	Validated
Control A3 (CA3): III-3	F	68	+/+	None	CA3	46,XX (P13)	Validated
Unrelated Control (U2)[f]	M	51	+/+	rs538089 Exon 5: T>C<br>rs505058 Exon 7: T>C	U2	46,XY (P12)	Validated
**PATIENTS**							
Patient A1 (PA1): IV-5	F	38	-/+	rs4641 Exon 10: C>T	PA1	46,XX (P19)	Validated
Patient A2 (PA2): III-5	M	62	-/+	rs4641 Exon 10: C>T	PA2	46,XY (P11)	Validated
Patient A3 (PA3): III-1	F	70	-/+	rs4641 Exon 10: C>T	PA3	46,XX (P10)	Validated

[a]Identification: Study family pedigree [1]
[b]*LMNA* Genotype: +/+ homozygous normal allele; +/- heterozygous *LMNA* c.357-2A>G mutation
[c]*LMNA* Coding SNV: detected in fibroblast gDNA and cDNA [1] and used for allelic expression.
[d]Karyotype: G-banded metaphase chromosome constitution [2]
[e]ICC-DIFF: Differentiation capability by immunocytochemistry staining for germ layer markers in embryoid bodies (EBs).
[f]CA2 had poor viability and failed CM differentiation; therefore, U2 was used instead.
Abbreviations: iPSC, induced pluripotent stem cell; Bx, biopsy; SNV, single nucleotide variant; F, female; M, male; P, Passage

Table S2. Methods: Primary and secondary antibodies.

Antigen (host)	Company, Catalog No.	Dilution	Antigen (host)	Company, Catalog No.	Dilution	Assay
**Primary antibodies**			**Secondary antibodies**			
AFP/alpha-fetoprotein (mouse)	Thermo Fisher Sci, A25530*	1:500	Anti-mouse (goat) AF-488	Thermo Fisher Sci, A25536*	1:250	ICC-DIFF ENDO
FOXA2/Forkhead Box A2 (mouse)	Thermo Fisher Sci, BDB561580	1:100	Anti-mouse (goat) AF-488	Abcam, ab150113	1:250	ICC-DIFF ENDO
SMA/smooth muscle actin (mouse)	Thermo Fisher Sci, A25531*	1:100	Anti-mouse (goat) AF-594	Thermo Fisher Sci, A25534*	1:250	ICC-DIFF MESO
Beta-III tubulin (rabbit)	Thermo Fisher Sci, A25532*	1:500	Anti-rabbit (donkey) AF-647	Thermo Fisher Sci, A25537*	1:250	ICC-DIFF ECTO
Lamin A/C N-term (E-1) monoclonal (mouse)	SC Biotech, sc-376248	1:500	Anti-mouse (goat) AF-680	Thermo Fisher Sci, A21057	1:2000	WB
Beta-Actin (13E5) monoclonal (rabbit)	Cell Signaling, 4970S	1:1000	Anti-rabbit (goat) AF-790	Thermo Fisher Sci, A11369	1:2000	WB-control

*3-Germ Layer Immunocytochemistry Kit (A25538, Life Technologies)

Abbreviations: AF, Alexa Fluor; ICC-DIFF, differentiation capability by immunocytochemistry; ENDO, endoderm; MESO, mesoderm; ECTO, ectoderm

Table S3. Methods: Main software packages.

Name	Version	Source	Reference
AzureSpot	2.1	azurebiosystems.com	
bcl2fastq2	2.20	support.illumina.com	
BioRender		www.biorender.com	
Cell Ranger	3.0.2	www.10xgenomics.com	[3]
Cluster Profiler	4.6.2	bioconductor.org	[4]
Condiments	1.6	bioconductor.org	[5]
DoubletFinder	2.0	github.com	[6]
EnhancedVolcano	1.16.0	github.com	[7]
Integrative Genomics Viewer	2.9.2	igv.org	[8]
Molecular Signatures Database R	7.5.1	cran.r-project.org	[9]
Pretty heatmaps (pheatmap)	1.0.12	cran.r-project.org	
R	4.2.2	www.r-project.org	
RStudio	2023.06.1+524	posit.co	
Seurat	4.3.0	cran.r-project.org	[10, 11]
Slingshot	2.6.0	bioconductor.org	[12]
SoupX	1.6.2	cran.r-project.org	[13]
Tidyverse includes ggplot2	1.3.2	cran.r-project.org	
TradeSeq	1.12.0	bioconductor.org	[14]
VennDiagram	1.7.3	cran.r-project.org	[15]

Table S4. scRNA-seq Summary Metrics using Cell Ranger [3].

Sample	Set	Shallow Sequencing			Deep Sequencing			
		Estimated No. Cells Captured	Mean Reads Per Cell	Median Genes Per Cell	Estimated No. Cells Captured	Mean Reads Per Cell	Median Genes Per Cell	% Reads Mapped Confidently to Genome
Control A1 D00	B	11,825	3,072	820	14,080	40,657	4,482	93.0
Control A1 D02	A	5,684	6,015	1,298	6,360	44,140	4,396	94.2
Control A1 D04	A	4,899	5,724	1,166	5,570	36,259	3,698	93.3
Control A1 D09	A	6,517	5,913	1,057	7,306	47,307	3,908	93.6
Control A1 D09	B	10,311	4,200	853	13,727	29,371	3,236	92.0
Control A1 D16	B	8,054	7,580	1,383	9,822	46,790	4,068	92.9
Control A1 D19	B	10,077	5,571	1,076	12,601	41,416	3,550	93.9
Control A1 D30	A	4,195	9,957	1,502	4,631	48,000	3,682	94.0
Patient A1 D00	B	10,764	3,386	856	13,471	39,451	4,410	93.1
Patient A1 D09	B	10,367	4,441	846	14,116	30,951	3,201	91.6
Patient A1 D16	B	8,806	6,519	1,269	10,836	43,074	4,040	92.9
Patient A1 D19	B	10,401	5,975	1,070	13,034	44,325	3,588	93.6
All Samples (n=12)	AB	101,900	5,696		125,554	40,978		93.2
Control Samples (n=8)	AB	60,501	6,043		74,097	41,743		93.4
Patient Samples (n=4)	B	41,399	5,002		51,457	39,450		92.8
Paired Samples (n=8)	B	80,605	5,093		101,687	39,504		92.9

Table S5. Single Sample Data: QC data processing using SoupX, Seurat, and DoubletFinder.

Single Sample Data (n=12)	Set	SoupX[a]			Seurat[b]			DoubletFinder[c]		
		Clustering Dims and Resolution	Method: Contamination Fraction (rho)	Non-expressed Genes for rho Calculation	nFeature	nCount	%Mt	pK	Exp	nExp, nExp-adj
Control A1 D00	B	1:30, 0.15	Auto: 0.01[a]<br>Manual: Not done	N/A	< 500	> 40000	N/A	0.23	0.10	1318, 489
Control A1 D02	A	1:30, 0.25	Auto: 0.16<br>Manual: 0.45	*SOX17, FOXA2*	< 900	> 30000	N/A	0.04	0.05	285, 206
Control A1 D04	A	1:30, 0.25	Auto: 0.03<br>Manual: 0.37	*SOX17, FOXA2*	< 900	> 30000	N/A	0.04	0.05	241, 178
Control A1 D09	A	1:30, 0.25	Auto: 0.04<br>Manual: 0.13	*TTN, MYL7, MYL2*	< 300	> 60000	N/A	0.04	0.06	423, 359
Control A1 D09	B	1:30, 0.25	Auto: 0.12<br>Manual: 0.33	*TTN, MYL7, MYL2*	< 100	> 30000	N/A	0.21	0.10	1312, 1073
Control A1 D16	B	1:30, 0.15	Auto: 0.04<br>Manual: 0.53	*TTN, MYL7, MYL2*	< 100	> 30000	N/A	0.17	0.08	740, 537
Control A1 D19	B	1:30, 0.05	Auto: 0.04<br>Manual: 0.47	*TTN, MYL7, MYL2*	< 100	> 40000	N/A	0.16	0.10	1140, 784
Control A1 D30	A	1:30, 0.05	Auto 0.05<br>Manual: 0.19	*TTN, MYL7, MYL2*	< 200	> 70000	N/A	0.30	0.04	182, 134
Patient A1 D00	B	1:30, 0.15	Auto: 0.13<br>Manual: Not done	N/A	< 500	> 40000	N/A	0.16	0.10	1252, 495
Patient A1 D09	B	1:30, 0.25	Auto: 0.01<br>Manual: 0.30	*TTN, MYL7, MYL2*	< 100	> 30000	N/A	0.17	0.10	1403, 1127
Patient A1 D16	B	1:30, 0.20	Auto: 0.06<br>Manual: 0.38	*TTN, MYL7, MYL2*	< 100	> 30000	N/A	0.10	0.08	823, 611
Patient A1 D19	B	1:30, 0.07	Auto: 0.04<br>Manual: 0.42	*TTN, MYL7, MYL2*	< 100	> 40000	N/A	0.005	0.10	1136, 768

[a]SoupX [13]: manually calculated rho values were used except for Day 0 due to lack of negative gene markers. For Day 0, automatically estimated global rho values were used. For CA1, this value was low (0.01); therefore, the greater rho value (0.13) estimated for PA1 was used instead.
[b]Seurat [10, 11]: QC covariate thresholds used to identify low-quality cells.
High %Mt thresholds were used after clustering/annotation for non-CM cells.
[c]DoubletFinder [6]: values for pK were obtained with 'paramSweep_v3' (PCs = 1:30). Expected proportion of doublets (Exp) was obtained from the 10x Chromium User Guide. Expected number of doublets (nExp) was adjusted (nExp-adj) using an estimated proportion of homotypic doublets.
Abbreviations: D, day; dims, dimensions; Auto, automatic; N/A, Not Applicable; Mt, Mitochondrial DNA genes; CM, cardiomyocyte

Table S6. Single Sample and Combined Data: QC processing of Raw to Singlet Data Matrices.

Single Sample Data (n=12)	Set	Total Number of Cells in Processed Data (gene-barcode matrix)					
		Raw[a]	Corrected[b] (%Raw)	Filtered: LQ Removed[c] (%Corrected)	Filtered: HQ Retained[c] (%Corrected)	Doublet Cells Removed[d] (%Filtered-HQ)	Singlet Cells Retained[d] (%Filtered-HQ)
Control A1 D00	B	14,080	14,080 (100%)	901 (6%)	13,179 (94%)	489 (4%)	12,690 (96%)
Control A1 D02	A	6,360	6,360 (100%)	664 (10%)	5,696 (90%)	206 (4%)	5,490 (96%)
Control A1 D04	A	5,570	5,570 (100%)	741 (13%)	4,829 (87%)	178 (4%)	4,651 (96%)
Control A1 D09	A	7,306	7,306 (100%)	253 (3%)	7,053 (97%)	359 (5%)	6,694 (95%)
Control A1 D09	B	13,727	13,727 (100%)	600 (4%)	13,127 (96%)	1,073 (8%)	12,054 (92%)
Control A1 D16	B	9,822	9,822 (100%)	573 (6%)	9,249 (94%)	537 (6%)	8,712 (94%)
Control A1 D19	B	12,601	12,601 (100%)	1,201 (10%)	11,400 (90%)	784 (7%)	10,616 (93%)
Control A1 D30	A	4,631	4,631 (100%)	80 (2%)	4,551 (98%)	134 (3%)	4,417 (97%)
Patient A1 D00	B	13,471	13,471 (100%)	953 (7%)	12,518 (93%)	495 (4%)	12,023 (96%)
Patient A1 D09	B	14,116	14,116 (100%)	90 (1%)	14,026 (99%)	1,127 (8%)	12,899 (92%)
Patient A1 D16	B	10,836	10,836 (100%)	547 (5%)	10,289 (95%)	611 (6%)	9,678 (94%)
Patient A1 D19	B	13,034	13,034 (100%)	1,669 (13%)	11,365 (87%)	768 (7%)	10,597 (93%)
**Combined Data**							
All 12 Samples- Merged	AB	125,554	125,554 (100%)	8,272 (7%)	117,282 (93%)	6,761 (6%)	110,521 (94%)
Eight Controls- Merged	AB	74,097	74,097 (100%)	5,013 (7%)	69,084 (93%)	3,760 (5%)	65,324 (95%)
Four Patients- Merged	B	51,457	51,457 (100%)	3,259 (6%)	48,198 (94%)	3,001 (6%)	45,197 (94%)
Eight Paired- Integrated	B	101,687	101,687 (100%)	6,534 (6%)	95,153 (94%)	5,884 (6%)	89,269 (94%)

[a]Raw Data Matrix: total cells from Cell Ranger filtered gene-barcode matrix

[b]Corrected Data Matrix: total cells from SoupX corrected gene-barcode matrix

[c]Filtered Data Matrix: total high-quality cells after low-quality cell removal using QC covariate thresholds for nFeature and nCounts in Seurat

[d]Singlet Data Matrix: total DoubletFinder predicted singlet cells after removal of predicted heterotypic doublet cells

Table S7. Single Sample Data: Individual and Subcluster Analyses for Cell Annotation

A. Individual Analyses of Singlet Data for Main Cell Types						B. Subcluster Analyses of Subset Data for Possible Cell Subtypes									
Single Sample Data (Singlet): n=12	Total Cells	Res	Vars.to regress	Main Cell Types: n=10	Cells (%Total)	Subset Data: n=32	QC Thresholds (LQ Cells) %Mt	nFeature	nCount	LQ Cells Removed (%Pre-filter)	Post-filter HQ Cells (%Pre-filter)	Res	Vars.to. regress	All Possible Subtypes: n=30*	Cells (%Post-filter)
Control A1 D00	12,690	0.15	%Mt & CC	PP	11,241 (89%)	A	>25%	N/A	N/A	185 (2%)	11,056 (98%)	0.15	CC	PP-A<br>PP-B<br>PP-C<br>PP-D	9670 (88%)<br>318 (3%)<br>722 (6%)<br>346 (3%)
				UNK	1,449 (11%)	B	N/A	N/A	N/A	N/A	1,449 (100%)	0.15	CC	UNK-A<br>UNK-B<br>UNK-C	566 (39%)<br>679 (47%)<br>204 (14%)
Control A1 D02	5,490	0.25	%Mt & CC	ME<br>CMESO<br>ENDO	5,292 (96%)	A	>20%	N/A	N/A	8 (0.2%)	5,284 (99.8%)	0.25	CC	ME-A<br>ME-B<br>ME-C<br>CMESO<br>ENDO	1,954 (37%)<br>185 (3%)<br>1,356 (26%)<br>1,609 (31%)<br>180 (3%)
				UNK	198 (4%)	B	N/A	N/A	N/A	N/A	198 (100%)	0.25	CC	UNK-A1<br>UNK-A2<br>UNK-B	90 (46%)<br>82 (41%)<br>26 (13%)
Control A1 D04	4,651	0.25	%Mt & CC	PP<br>CMESO<br>ENDO<br>CP	4,572 (98%)	A	>20%	N/A	N/A	40 (1%)	4,532 (99%)	0.25	CC	CMESO<br>CP<br>ENDO-A<br>ENDO-B<br>PP	1,175 (26%)<br>1,054 (23%)<br>1,515 (33.5%)<br>23 (0.5%)<br>765 (17%)
				UNK	79 (2%)	B	N/A	N/A	N/A	N/A	79 (100%)	0.25	CC	UNK-A	79 (100%)
Control A1 D09A	6,694	0.25	%Mt & CC	CP<br>CM<br>EPDC	4,568 (69%)	A	N/A	N/A	N/A	N/A	4,568 (100%)	0.25	CC	CP-A<br>CM-A<br>CM/UNK-B<br>CP-B<br>EPDC	421 (9%)<br>1,250 (28%)<br>334 (7%)<br>1,090 (24%)<br>1,473 (32%)
				ENDO<br>ENDOTH<br>UNK	1,823 (27%)	B	>20%	N/A	N/A	20 (1%)	1,803 (99%)	0.25	CC	ENDO-A1<br>ENDO-A2<br>ENDO-A3<br>ENDO-A4<br>ENDO-B<br>ENDOTH<br>UNK-C1<br>UNK-C2	658 (37%)<br>510 (28%)<br>238 (13%)<br>178 (10%)<br>42 (2%)<br>98 (5%)<br>62 (3%)<br>17 (1%)
				UNK	303 (4%)	C	N/A	N/A	N/A	N/A	303 (100%)	0.25	CC	UNK-A	303 (100%)
Control A1 D09B	12,054	0.15	%Mt & CC	CP<br>CM<br>EPDC	10,612 (88%)	A	N/A	N/A	N/A	N/A	10,612 (100%)	0.25	CC	CP-A<br>CM-A<br>CP-B<br>EPDC<br>CM/UNK-B1<br>CM/UNK-B2	3,077 (29%)<br>2,216 (21%)<br>396 (4%)<br>3,033 (28%)<br>1,258 (12%)<br>632 (6%)
				ENDO<br>ENDOTH<br>ECTO<br>UNK	532 (5%)	B	>20%	N/A	N/A	13 (2%)	519 (98%)	0.25	CC	ECTO-A<br>ECTO-B<br>ENDOTH<br>ENDO	159 (31%)<br>131 (25%)<br>97 (19%)<br>87 (17%)

														UNK-C	45 (8%)
				UNK	910 (7%)	C	N/A	N/A	N/A	N/A	910 (100%)	0.25	CC	UNK-A1<br>UNK-A2<br>UNK-A3	700 (77%)<br>118 (13%)<br>92 (10%)
Control A1 D16	8,712	0.15	%Mt & CC	CM	6,508 (75%)	A	N/A	N/A	N/A	N/A	6,508 (100%)	0.15	CC	CM-A1<br>CM-A2<br>CM-A3<br>CM/UNK-B	4,656 (72%)<br>240 (4%)<br>218 (3%)<br>1,394 (21%)
				EPDC	1,172 (13%)	B	>15%	<1500	>15000	154 (13%)	1,018 (87%)	0.15	CC	EPDC-A<br>EPDC-B	867 (85%)<br>151 (15%)
				UNK	1,032 (12%)	C	N/A	N/A	N/A	N/A	1,032 (100%)	0.15	CC	UNK-A1<br>UNK-A2<br>UNK-B	522 (51%)<br>328 (32%)<br>182 (17%)
Control A1 D19	10,616	0.05	%Mt & CC	CM	7,831 (74%)	A	N/A	N/A	N/A	N/A	7,831 (100%)	0.10	CC	CM-A1<br>CM-A2<br>CM/UNK-B	4,914 (63%)<br>526 (7%)<br>2,391 (30%)
				EPDC	1,359 (13%)	B	>15%	<1500	>30000	234 (17%)	1,125 (83%)	0.10	CC	EPDC	1,125 (100%)
				UNK	1,426 (13%)	C	N/A	N/A	N/A	N/A	1,426 (100%)	0.10	CC	UNK-A<br>UNK-B	904 (63%)<br>522 (37%)
Control A1 D30	4,417	0.05	%Mt & CC	CM	1,675 (38%)	A	N/A	N/A	N/A	N/A	1,675 (100%)	0.15	CC	VENTR-CM<br>ATRIAL-CM	1,070 (64%)<br>605 (36%)
				EPDC	1,960 (44%)	B	>15%	<1500	>40000	243 (12%)	1,717 (88%)	0.15	CC	CFIBRO<br>EPROG<br>EPDC-UNSP<br>VSM	797 (46%)<br>460 (27%)<br>382 (22%)<br>78 (5%)
				UNK	782 (18%)	C	N/A	N/A	N/A	N/A	782 (100%)	0.15	CC	UNK-A<br>UNK-B<br>UNK-C	444 (57%)<br>245 (31%)<br>93 (12%)
Patient A1 D00	12,023	0.15	%Mt & CC	PP	9,878 (82%)	A	>25%	N/A	N/A	118 (1%)	9,760 (99%)	0.15	CC	PP-A<br>PP-B<br>PP-C<br>PP-D	8,976 (92%)<br>382 (4%)<br>338 (3%)<br>64 (1%)
				UNK	2,145 (18%)	B	N/A	N/A	N/A	N/A	2,145 (100%)	0.15	CC	UNK-A<br>UNK-B<br>UNK-C	764 (36%)<br>1,004 (47%)<br>377 (17%)
Patient A1 D09B	12,899	0.25	%Mt & CC	CP<br>CM<br>EPDC	10,588 (82%)	A	N/A	N/A	N/A	N/A	10,588 (100%)	0.25	CC	CP-A<br>CM-A<br>CP-B<br>CP-C<br>EPDC<br>CM/UNK-B	819 (8%)<br>3,272 (31%)<br>2,160 (20%)<br>801 (8%)<br>1,314 (12%)<br>2,222 (21%)
				ENDO<br>ENDOTH<br>ECTO<br>UNK	1,066 (8%)	B	>20%	N/A	N/A	N/A	1,012 (95%)	0.25	CC	ECTO-A<br>ECTO-B<br>ECTO-C<br>ECTO-D<br>ENDO<br>ENDOTH<br>UNK-A	245 (24%)<br>247 (24%)<br>159 (16%)<br>188 (19%)<br>85 (8%)<br>31 (3%)<br>57 (6%)

				UNK	1,245 (10%)	C	N/A	N/A	N/A	N/A	1,245 (100%)	0.25	CC	UNK-A1<br>UNK-A2<br>UNK-A3<br>UNK-C	746 (60%)<br>173 (14%)<br>47 (4%)<br>279 (22%)
Patient A1 D16	9,678	0.20	%Mt & CC	CM	6,192 (64%)	A	N/A	N/A	N/A	N/A	6,192 (100%)	0.20	CC	CM-A1<br>CM-A2<br>CM-A3<br>CM-A4<br>CM/UNK-B<br>UNK-B	3,425 (55%)<br>1,222 (20%)<br>216 (3.5%)<br>117 (2%)<br>991 (16%)<br>221 (3.5%)
				EPDC	2,175 (22%)	B	>15%	<1500	>20000	60 (3%)	2,115 (97%)	0.20	CC	EPDC-A1<br>EPDC-A2<br>EPDC-B<br>EPDC-C	1,040 (49%)<br>889 (42%)<br>141 (7%)<br>45 (2%)
				UNK	1,311 (14%)	C	N/A	N/A	N/A	N/A	1,311 (100%)	0.20	CC	UNK-A1<br>UNK-A2<br>UNK-C1<br>UNK-C2<br>UNK-C3<br>UNK-C4	643 (49%)<br>284 (22%)<br>219 (17%)<br>95 (7%)<br>37 (3%)<br>33 (2%)
Patient A1 D19	10,597	0.07	%Mt & CC	CM	7,392 (70%)	A	N/A	N/A	N/A	N/A	7,392 (100%)	0.10	CC	CM-A<br>CM/UNK-B<br>UNK-B	5,129 (69%)<br>2,201 (30%)<br>62 (1%)
				EPDC	1,164 (11%)	B	>15%	<2000	>40000	31 (3%)	1,133 (97%)	0.10	CC	EPDC	1,133 (100%)
				UNK	2,041 (19%)	C	N/A	N/A	N/A	N/A	2,041 (100%)	0.10	CC	UNK-A1<br>UNK-A2<br>UNK-B<br>UNK-C	1,326 (65%)<br>164 (8%)<br>382 (19%)<br>169 (8%)

[*]Possible Cell subtypes (n=18 of 30 total, highlighted in blue) re-subsetted, integrated, and used for trajectory inference.

Abbreviations: D, day; Res, resolution; Mt, MtDNA genes; CC, cell cycle; PP, pluripotent; UNK, unknown; CM, cardiomyocyte; EPDC, Epicardium-derived cells; N/A, Not Applicable; LQ, low-quality; ME, Mesendoderm; CMESO, Cardiogenic Mesoderm; ENDO, Endoderm; CP, Cardiac Progenitors; ENDOTH, Endothelium; ECTO, Ectoderm; VENTR-CM, Ventricular-Cardiomyocytes; ATRIAL-CM, Atrial Cardiomyocytes; CFIBRO, Cardiac Fibroblasts; EPROG, Epicardial Progenitors; EPDC-UNSP, EPDC-Unspecified; VSM, Vascular Smooth Muscle

Table S8. Gene marker panels for cluster annotation.

Panel	No.	Gene Names	References
**Primary Marker Panel**	25	*POU5F1, SOX2, NANOG, CNMD, EOMES, MESP1, SOX17, FOXA2, PAX6, EGFL7, HAND1, HAPLN1, TMEM88, MYL7, TNNI1, NKX2-5, TTN, MYH7, MYL2, TBX18, TCF21, WT1, COL3A1, LUM, FBN1*	
Pluripotent cells (PP)	3	*POU5F1, SOX2, NANOG*	[16, 17]
Undifferentiated cells	1	*CNMD*	[18]
Early Differentiated cells	6	Mesendoderm: *EOMES*<br>Cardiogenic Mesoderm: *MESP1*<br>Endoderm: *SOX17, FOXA2*<br>Ectoderm: *PAX6*<br>Endothelium: *EGFL7*	[19-22]
Cardiac Progenitors (CP)	3	*HAND1, HAPLN1, TMEM88*	[20, 23]
Cardiomyocyte (CM)	6	Early CM: *MYL7, TNNI1, NKX2-5*<br>CM: *TTN, MYH7, MYL2*	[16, 19, 24, 25]
Epicardium-derived cell (EPDC)	6	Early EPDC/PEO: *TBX18, TCF21, WT1*<br>EPDC: *COL3A1, LUM, FBN1*	[19, 26, 27]
**Expanded Marker Panel**	38	*POU5F1, SOX2, NANOG, DNMT3B, NODAL, UTF1, LIN28A, LEFTY1, GDF3, SDC2, CNMD, EOMES, MIXL1, TBXT, SOX17, FOXA2, AFP, FOXA3, HHEX, IHH, APOA1, GATA6, HAND1, HAPLN1, TMEM88, GATA4, HCN4, TBX5, ISL1, TBX1, HAND2, PAX6, SOX1, EGFL7, SELE, PECAM1, VWF, CD34*	
Pluripotent cells	10	*POU5F1, SOX2, NANOG, DNMT3B, NODAL, UTF1, LIN28A, LEFTY1, GDF3, SDC2*	[17]
Undifferentiated cells	1	*CNMD*	[18]

Mesendoderm (ME)	3	*EOMES*, *MIXL1, TBXT*	[20]
Endoderm (ENDO)	8	*SOX17, FOXA2*, *AFP, FOXA3, HHEX, IHH, APOA1, GATA6*	[21, 28, 29]
Cardiac Progenitor (CP)	9	*HAND1, HAPLN1, TMEM88, GATA4, HCN4, TBX5, ISL1, TBX1, HAND2*	[19, 20, 23]
Ectoderm (ECTO)	2	*PAX6, SOX1*	[21]
Endothelium (ENDOTH)	5	*EGFL7*, *SELE, PECAM1, VWF, CD34*	[27]
**Cardiomyocyte Subtype Marker Panel**	27	*NKX2-5, TBX5, NPPA, GATA4, NR2F2, MYH6, MYL7, MYL4, PITX2, HAND1, IRX4, IRX5, MYL2, MYH7, MYL3, ACTN2, VDR, HAND2, ISL1, MYH3, TBX3, HCN4, IRX3, ID2, GJC1, GJD3, TBX18*	[21, 30, 31]
Cardiomyocyte-shared	3	*NKX2-5, TBX5, NPPA*	
Atrial CM (ATR-CM)	6	*GATA4, NR2F2, MYH6, MYL7, MYL4, PITX2,*	
Ventricular CM (VENTR-CM)	11	*HAND1, IRX4, IRX5, MYL2, MYH7, MYL3, ACTN2, VDR, HAND2, ISL1, MYH3*	
Nodal-shared	2	*TBX3, HCN4*	
Atrioventricular node (AVN)	4	*IRX3, ID2, GJC1, GJD3*	
Sinoatrial node (SAN)	1	*TBX18*	
**EPDC Subtype Marker Panel**	27	*TBX18, TCF21, WT1, TBX5, ETS1, VCAM1, BVES, GJA1, ALDH1A2, ITGA4, PDGFRB, PLAU, ISL1, ITLN1, EFEMP1, UPK3B, PDGFRA, VIM, SNAI2, THY1, S100A4, DDR2, PECAM1, ACTA2, SRF, KDR, NFATC1*	[21, 26, 32]
PEO/EPI-shared	6	*TBX18, TCF21, WT1, TBX5, ETS1, VCAM1*	
Proepicardial Progenitor (PEO)	2	*BVES, GJA1*	
Epicardial Progenitor (EPI)	8	*ALDH1A2, ITGA4, PDGFRB, PLAU, ISL1, ITLN1, EFEMP1, UPK3B*	
Mesenchymal/ EPDC-shared	3	*PDGFRA, VIM, SNAI2*	
Cardiac Fibroblast (CFIBRO)	4	*THY1, S100A4, DDR2, PECAM1*	
Vascular Smooth Muscle (VSM)	2	*ACTA2, SRF*	
Angioblasts/ Endothelial cells	2	*KDR, NFATC1*	

Table S9. Paired Sample Data: Individual and Subcluster Analyses.

A. Individual Analyses of Paired Singlet Data for Shared Cell Types: 89,269 total cells

Paired Sample Data (Singlet): n=4 pairs	Cells (%Total)	Integrated Data: n=4	Total Cells	Res	Vars.to. regress	Shared Cell Types: n=10	Cells (%Total)	CA1 Cells	PA1 Cells	CA1%	PA1%	Balanced Cell Type
CA1 D00<br>PA1 D00	12,690 (51%)<br>12,023 (49%)	CA1PA1 D00	24,713	0.15	%Mt & CC	PP<br>UNK	21,150 (86%)<br>3,563 (14%)	11,149<br>1,541	10,001<br>2,022	53%<br>43%	47%<br>57%	No<br>No
CA1 D09B<br>PA1 D09B	12,054 (48%)<br>12,899 (52%)	CA1PA1 D09	24,953	0.25	%Mt & CC	CM<br>CP<br>EPDC<br>UNK<br>ECTO<br>ENDO<br>ENDOTH	7,419 (30%)<br>7,037 (28%)<br>6,449 (26%)<br>2,567 (10%)<br>1,148 (4%)<br>197 (1%)<br>136 (1%)	3,878<br>3,512<br>2,998<br>1,179<br>291<br>94<br>102	3,541<br>3,525<br>3,451<br>1,388<br>857<br>103<br>34	52%<br>50%<br>46%<br>46%<br>25%<br>48%<br>75%	48%<br>50%<br>54%<br>54%<br>75%<br>52%<br>25%	Yes<br>Yes<br>Yes<br>No<br>No<br>No<br>Yes
CA1 D16<br>PA1 D16	8,712 (47%)<br>9,678 (53%)	CA1PA1 D16	18,390	0.15	%Mt & CC	CM<br>EPDC<br>UNK	12,555 (68%)<br>3,419 (19%)<br>2,416 (13%)	6,510<br>1,149<br>1,053	6,045<br>2,270<br>1,363	52%<br>34%<br>44%	48%<br>66%<br>56%	Yes<br>Yes<br>No
CA1 D19<br>PA1 D19	10,616 (50%)<br>10,597 (50%)	CA1PA1 D19	21,213	0.05	%Mt & CC	CM<br>EPDC<br>UNK	15,188 (72%)<br>2,893 (13%)<br>3,132 (15%)	7,828<br>1,364<br>1,424	7,360<br>1,529<br>1,708	52%<br>47%<br>45%	48%<br>53%<br>55%	Yes<br>Yes<br>No

B. Subcluster Analyses of Paired Subset Data for Possible Shared Cell Subtypes: 88,420 total cells

Paired Subset Data: n=11 pairs	Cells (%Total)	Integrated Subset Data: n=11*	Total Cells	Res	Vars.to. regress	Shared Subtypes: n=19*	Cells (%Total)	CA1 Cells	PA1 Cells	CA1%	PA1%	Balanced Subtype
CA1 D00 Subset-A (PP)<br>PA1 D00 Subset-A (PP)	11,056 (53%)<br>9,760 (47%)	CA1PA1 D00 Subset-A (PP)	20,816	0.10	CC	PP-A1<br>PP-A2<br>PP-B<br>PP-C<br>PP-D	17,677 (85%)<br>877 (4%)<br>716 (3%)<br>1,184 (6%)<br>362 (2%)	9,333<br>296<br>326<br>751<br>350	8,344<br>581<br>390<br>433<br>12	53%<br>34%<br>46%<br>63%<br>97%	47%<br>66%<br>54%<br>37%<br>3%	Yes<br>Yes<br>Yes<br>No<br>No
CA1 D00 Subset-B (UNK)<br>PA1 D00 Subset-B (UNK)	1,449 (40%)<br>2,145 (60%)	CA1PA1 D00 Subset-B (UNK)	3,594	0.15	CC	UNK-A<br>UNK-B<br>UNK-C	1,353 (38%)<br>1,776 (49%)<br>465 (13%)	487<br>762<br>200	866<br>1,014<br>265	36%<br>43%<br>43%	64%<br>57%<br>57%	No<br>No<br>No
CA1 D09B Subset-A<br>PA1 D09B Subset-A	10,612 (50%)<br>10,588 (50%)	CA1PA1 D09 Subset-A (CP/CM /EPDC)	21,200	0.25	CC	CP-A<br>CP-B<br>CP-C<br>CM-A<br>CM/UNK-B1<br>CM/UNK-B2<br>EPDC	5,946 (28%)<br>4,750 (23%)<br>1,216 (6%)<br>3,433 (16%)<br>2,191 (10%)<br>1,936 (9%)<br>1,728 (8%)	3,053<br>2,252<br>437<br>2,219<br>1,244<br>641<br>766	2,893<br>2,498<br>779<br>1,214<br>947<br>1,295<br>962	51%<br>47%<br>36%<br>65%<br>57%<br>33%<br>44%	49%<br>53%<br>64%<br>35%<br>43%<br>67%<br>56%	Yes<br>Yes<br>Yes<br>Yes<br>Yes<br>Yes<br>Yes
CA1 D09B Subset-B<br>PA1 D09B Subset-B	519 (34%)<br>1,012 (66%)	CA1PA1 D09 Subset-B (ENDO/ ENDOTH/ ECTO/UNK)	1,531	0.25	CC	ECTO-A<br>ECTO-B<br>ECTO-C<br>ECTO-D<br>ENDO<br>ENDOTH<br>UNK-A	286 (19%)<br>289 (19%)<br>205 (13%)<br>191 (12%)<br>166 (11%)<br>132 (9%)<br>93 (6%)	83<br>88<br>47<br>13<br>81<br>101<br>19	203<br>201<br>158<br>178<br>85<br>31<br>74	29%<br>30%<br>23%<br>7%<br>49%<br>77%<br>20%	71%<br>70%<br>77%<br>93%<br>51%<br>23%<br>80%	Yes<br>Yes<br>No<br>No<br>No<br>No<br>No

						UNK-C1	116 (8%)	67	49	58%	42%	No
						UNK-C2	53 (3%)	20	33	38%	62%	No
CA1 D09B Subset-C (UNK)<br>PA1 D09B Subset-C (UNK)	910 (42%)<br>1,245 (58%)	CA1PA1 D09 Subset-C (UNK)	2,155	0.25	CC	UNK-A1a	806 (37%)	145	661	18%	82%	No
						UNK-A1b	623 (29%)	505	118	81%	19%	No
						UNK-A2	195 (9%)	100	95	51%	49%	No
						UNK-A3	165 (8%)	98	67	59%	41%	No
						UNK-A4	91 (4%)	44	47	48%	52%	Yes
						UNK-C	275 (13%)	18	257	7%	93%	No
CA1 D16 Subset-A (CM)<br>PA1 D16 Subset-A (CM)	6,508 (51%)<br>6,192 (49%)	CA1PA1 D16 Subset-A (CM)	12,700	0.15	CC	CM-A1	5,198 (41%)	2,601	2,597	50%	50%	Yes
						CM-A2	3,441 (27%)	1,794	1,647	52%	48%	Yes
						CM-A3	1,368 (11%)	712	656	52%	48%	No
						CM/UNK-B	2,472 (19%)	1,393	1,079	56%	44%	Yes
						UNK-B	221 (2%)	8	213	4%	96%	No
CA1 D16 Subset-B (EPDC)<br>PA1 D16 Subset-B (EPDC)	1,018 (32%)<br>2,115 (68%)	CA1PA1 D16 Subset-B (EPDC)	3,133	0.15	CC	EPDC-A1	2,126 (68%)	672	1454	32%	68%	No
						EPDC-A2	723 (23%)	227	496	31%	69%	No
						EPDC-B	263 (8.5%)	98	165	37%	63%	No
						EPDC-C	21 (0.5%)	21	0	100%	0%	No
CA1 D16 Subset-C (UNK)<br>PA1 D16 Subset-C (UNK)	1,032 (44%)<br>1,311 (56%)	CA1PA1 D16 Subset-C (UNK)	2,343	0.15	CC	UNK-A1	889 (38%)	386	503	43%	57%	No
						UNK-A2	569 (24%)	258	311	45%	55%	Yes
						UNK-A3	243 (10%)	126	117	52%	48%	No
						UNK-A4	84 (4%)	63	21	75%	25%	No
						UNK-B	280 (12%)	197	83	70%	30%	No
						UNK-C1	216 (9%)	2	214	1%	99%	No
						UNK-C2	62 (3%)	0	62	0%	100%	No
CA1 D19 Subset-A (CM)<br>PA1 D19 Subset-A (CM)	7,831 (51%)<br>7,392 (49%)	CA1PA1 D19 Subset-A (CM)	15,223	0.10	CC	CM-A1	9,978 (65%)	4,927	5,051	49%	51%	Yes
						CM-A2	590 (4%)	507	83	86%	14%	No
						CM/UNK-B	4,591 (30%)	2,389	2,202	52%	48%	Yes
						UNK-B	64 (0.4%)	8	56	13%	88%	No
CA1 D19 Subset-B (EPDC)<br>PA1 D19 Subset-B (EPDC)	1,125 (50%)<br>1,133 (50%)	CA1PA1 D19 Subset-B (EPDC)	2,258	0.10	CC	EPDC	2,258 (100%)	1,125	1,133	50%	50%	Yes
CA1 D19 Subset-C (UNK)<br>PA1 D19 Subset-C (UNK)	1,426 (41%)<br>2,041 (59%)	CA1PA1 D19 Subset-C (UNK)	3,467	0.10	CC	UNK-A1	2,005 (58%)	740	1,265	37%	63%	No
						UNK-A2	360 (10%)	154	206	43%	57%	No
						UNK-B	935 (27%)	531	404	57%	43%	No
						UNK-C	167 (5%)	1	166	1%	99%	No

*Integrated Subset Data with primarily ‘balanced” cell types (n=6) and ‘balanced’ cell subtypes (n=14): highlighted in yellow used for cell type analyses of differential expression.

Abbreviations: D, day; PP, pluripotent; UNK, unknown; CM, cardiomyocyte; EPDC, Epicardium-derived cells; Res, resolution; Mt, MtDNA genes; CC, cell cycle; CP, Cardiac Progenitors; ECTO, Ectoderm; ENDO, Endoderm; ENDOTH, Endothelium

Table S10. Single Subset Data: Trajectory Inference, Lineage DEG, and Enrichment.

Single Subset Data (n=7)	Total Cells	Res	k	Lineage Topology using Slingshot [12]	Cell Lineage(s)	Lineage DEG[a]		ORA GO BP GS[b]	
						AT	SET	AT	SET
CA1 D00 PP-AB	9,988	0.05	6	Single Trajectory	PP-A → PP-B	367	83	36	45
CA1 D02 ME/CMESO/ENDO	3,145	0.15	6	Bifurcating Trajectory	1. ME → CMESO<br>2. ME → ENDO	907	469	27	187
CA1 D04 CMESO/CP	2,229	0.15	6	Single Trajectory	CMESO → CP	1,683	711	0	114
CA1 D09A CP-A/CM-A	1,671	0.05	6	Single Trajectory	CP-A → CM-A	1,835	1,003	139	239
CA1 D09B CP-A/CM-A	5,293	0.05	6	Single Trajectory	CP-A → CM-A	1,806	483	153	305
PA1 D00 PP-AB	9,358	0.05	6	Single Trajectory	PP-A → PP-B	301	99	174	246
PA1 D09B CP-A/CM-A	4,091	0.10	6	Single Trajectory	CP-A → CM-A	1,538	247	117	195

[a]Lineage DEG using TradeSeq [14]: Threshold = FDR <0.05 & Fold Change > 2x;
For bifurcating trajectories, global lineage DEG were identified across both lineages.
[b]ORA Parameters: DEG = top 100 DEG ranked by Wald statistic, minimum #DEG = 3, adjusted p-value < 0.05, q-value cutoff = 0.20
Abbreviations: PP, pluripotent; ME, mesendoderm; CMESO, cardiogenic mesoderm; ENDO, endoderm; CP, Cardiac Progenitors; CM, cardiomyocyte; Res, resolution; DEG, differentially expressed gene; AT, Association Test; SET, Start-End Test; ORA, Over-Representation Analysis; GO BP, Gene Ontology Biological Processes; GS, gene set

Table S11. Paired Subset Data: Integration, Trajectory Inference, Lineage DEG and Enrichment.

Paired Subset Data (n=2)	Cells (%Total)	Integrated Subset Data (n=2)	Total Cells	Patient (%Total)	Control (%Total)	Res	Vars.to. regress	k*	Lineage Topology [12]	Cell Lineage(s)
CA1 D00 PP-AB<br>PA1 D00 PP-AB	9,988 (52%)<br>9,358 (48%)	CA1PA1 D00 PP-AB	19,346	9,988 (52%)	9,358 (48%)	0.05	CC	6	Single-Trajectories	PP-A→PP-B
CA1 D09B CP-AB/CM-A/EPDC<br>CA1 D16 CM-A123<br>CA1 D16 EPDC-AB<br>CA1 D19 CM-A12<br>CA1 D19 EPDC<br>PA1 D09B CP-ABC/CM-A/EPDC<br>PA1 D16 CM-A1234<br>PA1 D16 EPDC-A12BC<br>PA1 D19 CM-A<br>PA1 D19 EPDC	8,722 (20%)<br>5,114 (12%)<br>1,018 (2%)<br>5,440 (13%)<br>1,125 (3%)<br>8,366 (19%)<br>4,980 (11%)<br>2,115 (5%)<br>5,129 (12%)<br>1,133 (3%)	CA1PA1 D09BD16D19 CP/CM-A/EPDC	43,142	21,419 (50%)	21,723 (50%)	0.20	CC	6	Bifurcating-Trajectories	1. CP1→CP2→CM1→CM2<br>2. CP1→CP2→EPDC
Totals	62,488		62,488	31,407 (50%)	31,081 (50%)					

*Tradeseq [14] parameters: number of knots (k) determined using ‘evaluateK (k = 3:10, nGenes = 200).

Abbreviations: PP, pluripotent; CP, Cardiac Progenitors; CM, cardiomyocyte; EPDC, Epicardium-derived cells; Res, resolution; CC, cell cycle

Table S12. Lamin A/C Western Blot Quantification Data and Statistical Analyses [33].

A. Technical Replicate (TR) Blots: Raw Band Volumes and Normalized Ratio (NR)

	Raw Band Volume (BV)								
Cell Type	*Fibroblast*	*Day 19 Differentiated Cells*							
Sample ID	CA1	CA1	PA1	CA3	PA3	U2	PA2	CA1	
Blot 1									
Lamin A	9048601.8	483888.56	173706.08	516436.6	449955.8	278777	153263.4		
Lamin C	8877858.2	2529873	746270.23	2318767	1937147	2856458	1054945.5		
B-Actin	13319695	3442580	7558977	7507949	6711578	8815425	7482798.55		
Blot 2									
Lamin A	439561.51	88750	33436	85352.5	60939	124498.31	30014.9	119215.5	
Lamin C	491520.49	343447	98663.63	242385.43	197033.5	249367.69	127044.24	250459	
B-Actin	792273	741697	684119	560653.5	538305.5	744770	717710	361561	
Blot 3									
Lamin A	606177	116682	44697	87484	65595	155078	36141		
Lamin C	426928	137338.5	112898	230149	168734	305713	161105		
B-Actin	585176.5	127469.3	386324.5	410290.5	246465.4	585631.5	422438		
	Normalized Ratio (NR) = Lamin BV / B-Actin BV								
Cell Type	*Fibroblast*	*Day 19 Differentiated Cells*							
Sample ID	CA1	CA1 (#1)*	PA1	CA3	PA3	U2	PA2	CA1 (#2)*	
Blot 1									
Lamin A/B-Actin	0.67934	0.1405599	0.0229801	0.068785	0.067042	0.031624	0.0204821		
Lamin C/B-Actin	0.6665211	0.734877	0.0987264	0.308842	0.288628	0.324029	0.14098275		
Lamin A+C/B-Actin	1.3458611	0.8754369	0.1217065	0.377627	0.355669	0.355653	0.16146484		
Blot 2									Mean CA1*
Lamin A/B-Actin	0.224691	0.119658027	0.048874538	0.152237523	0.113205234	0.167163433	0.041820373	0.329724	0.224691
Lamin C/B-Actin	0.57788571	0.463055668	0.144219982	0.432326615	0.366025426	0.3348251	0.177013334	0.692716	0.57788571
Lamin A+C/B-Actin	0.802576946	0.582713696	0.19309452	0.584564138	0.47923066	0.501988533	0.218833707	1.02244	0.802576946
Blot 3									
Lamin A/B-Actin	1.035887	0.915373	0.115698	0.213225	0.266143	0.264805	0.085553		
Lamin C/B-Actin	0.729571	1.077424	0.292236	0.560942	0.684615	0.522023	0.38137		
Lamin A+C/B-Actin	1.765459	1.992797	0.407934	0.774166	0.950758	0.786828	0.466923		

*Mean CA1 NR calculated using CA1 (#1) NR and CA1 (#2) NR for Blot 2

B. Patient vs. Control Samples: Mean Normalized Ratio (NR) of Biological Replicates

	NR Lamin A					
	Day 19 Differentiated Cells					
	Control			Patient		
**Technical Replicate (TR) / Sample ID**	**CA1**	**U2**	**CA3**	**PA1**	**PA2**	**PA3**
**Blot 1**	0.1405599	0.031624	0.068785	0.0229801	0.0204821	0.067042
**Blot 2**	0.224691*	0.167163433	0.152237523	0.048874538	0.041820373	0.113205234
**Blot 3**	0.915373205	0.264805	0.213225	0.115698	0.085553	0.266143
**Mean NR of TR**	0.426874767	0.15453065	0.14474912	0.062517572	0.049285284	0.148796616
**SD of TR**	0.42513829	0.072510204	0.117102655	0.047840931	0.104213127	0.033171701
**Mean NR ± SD of BR (n=3)**	0.242051512 ± 0.160136336			0.08686649 ± 0.054039603		
	**NR Lamin C**					
	Day 19 Differentiated Cells					
	Control			Patient		
**Technical Replicate (TR) / Sample ID**	**CA1**	**U2**	**CA3**	**PA1**	**PA2**	**PA3**
**Blot 1**	0.73487704	0.324029471	0.308841661	0.098726353	0.140982748	0.288627604
**Blot 2**	0.57788571*	0.3348251	0.432326615	0.144219982	0.177013334	0.366025426
**Blot 3**	1.077423964	0.522022808	0.560941577	0.29223619	0.381369574	0.177013334
**Mean NR of TR**	0.796728905	0.393625793	0.434036618	0.178394175	0.446422836	0.233121885
**SD of TR**	0.255448344	0.111326014	0.126058657	0.101180141	0.209879505	0.129644065
**Mean NR ± SD of BR (n=3)**	0.541463772 ± 0.221987555			0.285979632 ± 0.141616725		
	**NR Lamin A+C**					
	Day 19 Differentiated Cells					
	Control			Patient		
**Technical Replicate (TR) / Sample ID**	**CA1**	**U2**	**CA3**	**PA1**	**PA2**	**PA3**
**Blot 1**	0.875436899	0.355653244	0.377626966	0.121706457	0.161464844	0.35566934
**Blot 2**	0.802576946*	0.501988533	0.584564138	0.19309452	0.218833707	0.47923066
**Blot 3**	1.992797169	0.786827553	0.774166109	0.407934263	0.466922957	0.950758354
**Mean NR of TR**	1.223603671	0.548156443	0.578785738	0.240911747	0.282407169	0.595219451
**SD of TR**	0.667136508	0.219263376	0.198332714	0.148984746	0.162349479	0.314042646
**Mean NR ± SD of BR (n=3)**	0.783515284 ± 0.381435289			0.372846122 ± 0.193695355		

*NR value = Mean CA1 NR for Blot 2
Abbreviations: SD, Standard Deviation

C. Pairwise Comparisons: Relative Normalized Ratio (RNR) and Mean Fold Change (FC)

	CA1 vs PA1			
	**NR Lamin A+C**		**RNR Lamin A+C**	
**Technical Replicate / Sample ID**	**CA1**	**PA1**	**CA1/CA1**	**PA1/CA1**
**Blot 1**	0.875436899	0.121706457	1	0.139023677
**Blot 2**	0.802576946	0.19309452	1	0.240593156
**Blot 3**	1.992797169	0.407934263	1	0.204704357
			**Mean FC of TR**	0.19477373
			**SD of TR**	0.051507793
			**% Change**	-80.52262701
			**CV (%)**	26.44493842
			**% Change/CV**	-3.044916412
	U2 vs PA2			
	**NR Lamin A+C**		**RNR Lamin A+C**	
**Technical Replicate / Sample ID**	**U2**	**PA2**	**U2/U2**	**PA2/U2**
**Blot 1**	0.355653244	0.161464844	1	0.453995139
**Blot 2**	0.501988533	0.218833707	1	0.435933677
**Blot 3**	0.786827553	0.466922957	1	0.593424766
			**Mean FC of TR**	0.494451194
			**SD of TR**	0.086188051
			**% Change**	-50.55488059
			**CV (%)**	17.43105324
			**% Change/CV**	-2.900276873
	CA3 vs PA3			
	**NR Lamin A+C**		**RNR Lamin A+C**	
**Technical Replicate / Sample ID**	**CA3**	**PA3**	**CA3/CA3**	**PA3/CA3**
**Blot 1**	0.377626966	0.35566934	1	0.941853661
**Blot 2**	0.584564138	0.47923066	1	0.819808518
**Blot 3**	0.774166109	0.950758354	1	1.228106401
			**Mean FC of TR**	0.996589527
			**SD of TR**	0.20958006
			**% Change**	-0.341047311
			**CV (%)**	21.0297273
			**% Change/CV**	-0.016217391

Abbreviations: CV, coefficient of variation

REFERENCES

1. Zaragoza, M.V., et al., *Exome Sequencing Identifies a Novel LMNA Splice-Site Mutation and Multigenic Heterozygosity of Potential Modifiers in a Family with Sick Sinus Syndrome, Dilated Cardiomyopathy, and Sudden Cardiac Death.* PLoS One, 2016. **11**(5): p. e0155421.
2. Morival, J.L.P., et al., *DNA methylation analysis reveals epimutation hotspots in patients with dilated cardiomyopathy-associated laminopathies.* Clin Epigenetics, 2021. **13**(1): p. 139.
3. 10X Genomics. *Quality Assessment Using the Cell Ranger Web Summary at www.10xgenomics.com/resources/analysis-guides/quality-assessment-using-the-cell-ranger-web-summary*. Analysis Guides 2022.
4. Wu, T., et al., *clusterProfiler 4.0: A universal enrichment tool for interpreting omics data.* Innovation (Camb), 2021. **2**(3): p. 100141.
5. Roux de Bézieux, H., et al., *Trajectory inference across multiple conditions with condiments.* Nat Commun, 2024. **15**(1): p. 833.
6. McGinnis, C.S., L.M. Murrow, and Z.J. Gartner, *DoubletFinder: Doublet Detection in Single-Cell RNA Sequencing Data Using Artificial Nearest Neighbors.* Cell Syst, 2019. **8**(4): p. 329-337.e4.
7. Blighe, K., S. Rana, and M. Lewis. *EnhancedVolcano: Publication-ready volcano plots with enhanced colouring and labeling. R package version 1.18.0, //github.com/kevinblighe/EnhancedVolcano.* 2023.
8. Robinson, J.T., et al., *Integrative genomics viewer.* Nat Biotechnol, 2011. **29**(1): p. 24-6.
9. Subramanian, A., et al., *Gene set enrichment analysis: a knowledge-based approach for interpreting genome-wide expression profiles.* Proc Natl Acad Sci U S A, 2005. **102**(43): p. 15545-50.
10. Stuart, T., et al., *Comprehensive Integration of Single-Cell Data.* Cell, 2019. **177**(7): p. 1888-1902.e21.
11. Butler, A., et al., *Integrating single-cell transcriptomic data across different conditions, technologies, and species.* Nat Biotechnol, 2018. **36**(5): p. 411-420.
12. Street, K., et al., *Slingshot: cell lineage and pseudotime inference for single-cell transcriptomics.* BMC Genomics, 2018. **19**(1): p. 477.
13. Young, M.D. and S. Behjati, *SoupX removes ambient RNA contamination from droplet-based single-cell RNA sequencing data.* Gigascience, 2020. **9**(12).

14. Van den Berge, K., et al., *Trajectory-based differential expression analysis for single-cell sequencing data.* Nat Commun, 2020. **11**(1): p. 1201.
15. Chen, H. and P.C. Boutros, *VennDiagram: a package for the generation of highly-customizable Venn and Euler diagrams in R.* BMC Bioinformatics, 2011. **12**: p. 35.
16. Lian, X., et al., *Robust cardiomyocyte differentiation from human pluripotent stem cells via temporal modulation of canonical Wnt signaling.* Proc Natl Acad Sci U S A, 2012. **109**(27): p. E1848-57.
17. Nguyen, Q.H., et al., *Single-cell RNA-seq of human induced pluripotent stem cells reveals cellular heterogeneity and cell state transitions between subpopulations.* Genome Res, 2018. **28**(7): p. 1053-1066.
18. Sekine, K., et al., *Robust detection of undifferentiated iPSC among differentiated cells.* Sci Rep, 2020. **10**(1): p. 10293.
19. Friedman, C.E., et al., *Single-Cell Transcriptomic Analysis of Cardiac Differentiation from Human PSCs Reveals HOPX-Dependent Cardiomyocyte Maturation.* Cell Stem Cell, 2018. **23**(4): p. 586-598.e8.
20. Ruan, H., et al., *Single-cell reconstruction of differentiation trajectory reveals a critical role of ETS1 in human cardiac lineage commitment.* BMC Biol, 2019. **17**(1): p. 89.
21. Edgar, R., et al., *LifeMap Discovery™: the embryonic development, stem cells, and regenerative medicine research portal.* PLoS One, 2013. **8**(7): p. e66629.
22. Grancharova, T., et al., *A comprehensive analysis of gene expression changes in a high replicate and open-source dataset of differentiating hiPSC-derived cardiomyocytes.* Sci Rep, 2021. **11**(1): p. 15845.
23. Später, D., et al., *How to make a cardiomyocyte.* Development, 2014. **141**(23): p. 4418-31.
24. Yang, X., L. Pabon, and C.E. Murry, *Engineering adolescence: maturation of human pluripotent stem cell-derived cardiomyocytes.* Circ Res, 2014. **114**(3): p. 511-23.
25. Lian, X., et al., *Directed cardiomyocyte differentiation from human pluripotent stem cells by modulating Wnt/β-catenin signaling under fully defined conditions.* Nat Protoc, 2013. **8**(1): p. 162-75.
26. Floy, M.E., et al., *Direct coculture of human pluripotent stem cell-derived cardiac progenitor cells with epicardial cells induces cardiomyocyte proliferation and reduces sarcomere organization.* J Mol Cell Cardiol, 2022. **162**: p. 144-157.
27. Karlsson, M., et al., *A single-cell type transcriptomics map of human tissues.* Sci Adv, 2021. **7**(31).
28. Biddy, B.A., et al., *Single-cell mapping of lineage and identity in direct reprogramming.* Nature, 2018. **564**(7735): p. 219-224.

29. Cuomo, A.S.E., et al., *Single-cell RNA-sequencing of differentiating iPS cells reveals dynamic genetic effects on gene expression.* Nat Commun, 2020. **11**(1): p. 810.
30. Churko, J.M., et al., *Defining human cardiac transcription factor hierarchies using integrated single-cell heterogeneity analysis.* Nat Commun, 2018. **9**(1): p. 4906.
31. Zhao, M.T., N.Y. Shao, and V. Garg, *Subtype-specific cardiomyocytes for precision medicine: Where are we now?* Stem Cells, 2020. **38**(7): p. 822-833.
32. D'Antonio-Chronowska, A., et al., *Association of Human iPSC Gene Signatures and X Chromosome Dosage with Two Distinct Cardiac Differentiation Trajectories.* Stem Cell Reports, 2019. **13**(5): p. 924-938.
33. Pillai-Kastoori, L., A.R. Schutz-Geschwender, and J.A. Harford, *A systematic approach to quantitative Western blot analysis.* Anal Biochem, 2020. **593**: p. 113608.
